# The Aging Landscape by scRNAseq of Mesenchymal Lineage Cells in Mouse Bone

**DOI:** 10.1111/acel.70256

**Published:** 2025-10-13

**Authors:** Melda Onal, Intawat Nookaew, Ana Resende‐Coelho, Olivia Reyes‐Castro, Adriana Marques‐Carvalho, Landon Gatrell, Alongkorn Kurilung, Alicen James, Aaron Warren, Ha‐Neui Kim, Jinhu Xiong, Charles A. O'Brien, Maria Almeida

**Affiliations:** ^1^ Center for Musculoskeletal Disease Research University of Arkansas for Medical Sciences Little Rock Arkansas USA; ^2^ Department of Physiology and Cell Biology University of Arkansas for Medical Sciences Little Rock Arkansas USA; ^3^ Department of Biomedical Informatics University of Arkansas for Medical Sciences Little Rock Arkansas USA; ^4^ Division of Endocrinology and Metabolism University of Arkansas for Medical Sciences Little Rock Arkansas USA; ^5^ Department of Orthopedic Surgery University of Arkansas for Medical Sciences Little Rock Arkansas USA

## Abstract

A decrease in osteoblast number and bone formation are seminal contributors to age‐related osteoporosis. However, the aging‐associated molecular mechanisms that impact osteoblast precursors, osteoblasts, osteocytes, and other bone mesenchymal cell types remain unclear. We performed single‐cell RNA‐sequencing of mesenchymal cells present at the endosteum and periosteum of young and old C57BL/6 mice of both sexes. Osteoblast precursors and osteoblasts from female endosteum exhibited the greatest changes with aging. Transcriptional changes revealed decreased matrix protein production and autophagy, as well as increased senescence, phosphorylation, and hypoxia. Because deficient macroautophagy in osteoblast lineage cells decreases bone formation, we contrasted the transcriptional changes caused by autophagy inactivation in Atg7^f/f^; Osx1‐Cre mice with those caused by aging and found a causal link between autophagy deficiency and increased senescence in osteoblastic cells. Overall, these findings reveal distinct features of aging in males and females and molecular pathways that might be implicated in the development of intracortical porosity in the female skeleton. The transcriptional changes in periosteal cells indicate mechanisms that might contribute to the decreased response to mechanical loading and delayed fracture healing in the old skeleton. Our data provides a comprehensive resource and serves as a reference for understanding how future genetic and pharmacological interventions impact molecular mechanisms of aging in osteoblasts and other mesenchymal cells in the skeleton.

## Graphical abstract

scRNA‐seq analysis reveals age‐related changes in bone mesenchymal cells in male and female mice. Moreover, the comparison of aging and autophagy deficiency in bone mesenchymal cells shows overlap and suggests that age‐related decline in autophagy contributes to skeletal aging.

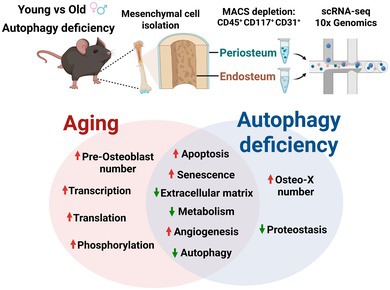

## 1 Introduction

Aging is the greatest risk factor for osteoporosis and bone fractures (Almeida et al. 2017; Manolagas 2018). In both humans and mice, loss of bone mass is due to an imbalance between the bone removed by osteoclasts and the new bone produced by osteoblasts. During aging, bones also undergo microarchitectural changes, including the loss of trabeculae, enlargement of the marrow space due to loss of bone at the endocortical surface, as well as enlargement of the outer surfaces due to periosteal bone formation (Seeman 2013; Zebaze and Seeman 2015). Throughout the skeleton, the number of osteoblasts decreases with aging, while at some skeletal sites, such as the endosteal surface of long bones, osteoclast number increases (Almeida et al. 2007; Parfitt et al. 1995; Piemontese et al. 2017). Osteoblasts secrete collagen I and other bone matrix proteins and are derived from mesenchymal precursors present in the bone marrow and in the periosteum (Mizoguchi and Ono 2021). Thus, osteoblasts and their precursors are found both at the inner and outer surfaces of bone but in very different niches. Indeed, endosteal osteoblasts are in direct contact with marrow cells, while periosteal osteoblasts are part of a fibrous layer that is in direct contact with tendons and muscle. A subset of osteoblasts becomes buried in the bone matrix and differentiates into osteocytes (Jilka and O'Brien 2016). While osteocytes are long‐lived terminally differentiated cells, osteoblasts are short‐lived and need to be continuously replaced via the proliferation and differentiation of precursors.

In humans, there are striking differences in female and male skeletal aging, with females losing more bone mass than males (Chavassieux and Meunier 2001). This is, at least in part, due to the loss of estrogens at menopause (Almeida et al. 2017). Similar sex differences occur in the mouse skeleton, even though female mice do not undergo menopause (Piemontese et al. 2017; Ucer et al. 2017). Consistent with this, sex differences in lifespan and health span have been documented in both humans and mice (Mitchell et al. 2016). Multiple mechanisms have been proposed to contribute to the loss of health span with aging (Lopez‐Otin et al. 2023). Many of these mechanisms are thought to play a role in the decline of multiple tissues. Moreover, mechanisms of aging are not independent of each other, as the attenuation or exacerbation of a specific mechanism frequently affects other mechanisms. Work using candidate approaches has revealed that an increase in mitochondrial reactive oxygen species (ROS) (Almeida et al. 2007; Ucer et al. 2017), cellular senescence (Farr et al. 2016; Farr et al. 2017), and a decrease in nicotinamide adenine dinucleotide (NAD^+^) (Kim et al. 2021) contribute to the loss of bone mass with aging. However, the effects of increasing NAD^+^ or eliminating mitochondrial ROS or senescent cells on skeletal aging are modest, suggesting that additional mechanisms are involved. A decline in autophagy is another aging mechanism common to many tissues (Lopez‐Otin et al. 2023). Young mice lacking *Atg7* (autophagy‐related 7), a protein critical for autophagy, in cells of the osteoblast lineage have low bone mass with spontaneous fractures, and a decrease in autophagy has been proposed to contribute to skeletal aging (Chen et al. 2014; Ma et al. 2018; Onal et al. 2013; Piemontese et al. 2016). However, the full spectrum of aging mechanisms that impact bone cells and the cell types affected remain unclear.

Recent studies using single‐cell RNA‐sequencing (scRNA‐seq) have expanded our understanding of the diverse mesenchymal cell types present in bone (Nookaew et al. 2024). In this study, we examined the effects of aging at a single‐cell level in mesenchymal bone cells from the endosteum and periosteum of mice and contrasted our findings between males and females. In addition, using mice deficient in Atg7, we examined whether lack of autophagy could replicate some of the effects of aging. Our findings provide a comprehensive molecular framework for understanding the effects of aging in mesenchymal cells of bone.

## 2 Methods

### 2.1 Animal Housing and Care

Wild‐type C57BL/6J mice were obtained from the NIA Rodent Aging Colony. Atg7^f/f^; Osx1‐Cre mice were generated by crossing Atg7^f/+^ (Komatsu et al. 2005) with Osx1‐Cre mice (Rodda and McMahon 2006), as previously described (Piemontese et al. 2016). Mice were socially housed at 2–5 animals per cage using a blend of 1/4″ corncob bedding and white enrichment paper both produced by Andersons Incorporated. Mice were provided ad libitum water and an irradiated Purina diet of 5V5R. The temperature range in the room was 68°F–79°F with a set point of 71°±2°, additionally, the humidity range was 30%–70%. The room was on a 12:12 h light cycle and the illumination was 364 lx measured 1 m from the floor. The following primer sets were used for genotyping mice for *Atg7* conditional allele and Osx1‐Cre transgene: *Atg7*‐flox primer#1 5′‐TGG CTG CTA CTT CTG CAA TGA TGT‐3′, primer #2 5′‐TCT CCC AAG ACA AGA CAG GGT GAA‐3′, primer #3 5′‐CAG GAC AGA GAC CAT CAG CTC CAC‐3′; Cre‐F 5′‐GAG AAT AGG AAC TTC GGA ATA GTA AC‐3′ and Cre‐R 5′‐CCC TGG AAG TGA CTA GCA TTG‐3′; IntCon‐F 5′‐AGA GAG CTC CCC TCA ATT ATG T‐3′ and IntCon‐R 5′‐AGC CAC TTC TAG CAC AAA GAA CT‐3′.

### 2.2 Cell Isolation

Separate preparations of cells from either the periosteum or the endosteum and trabecular bone were performed using 2–4 mice per group, as previously described (Nookaew et al. 2024). Briefly, to isolate endosteal and trabecular cells (hereafter referred to as endosteal preparations), femurs and tibias from two mice per group were cleaned of soft tissues, and the periosteum was removed by scraping with a scalpel and discarded. Epiphyses were removed and bone marrow was flushed away with phosphate‐buffered saline (PBS) containing 1% bovine serum albumin (BSA). The remaining bone fragments were then cut into smaller pieces and placed into Hank's Balanced Salt Solution (HBSS) containing 2 Wunsch units of LiberaseTM (Millipore Sigma, Catalog # 5401119001). The cells were incubated for 20 min at 37°C with shaking; the released cells were collected by pipetting and incubated on ice. Following this first digestion, bone fragments were digested (20 min with LiberaseTM at 37°C with shaking) and then decalcified (20 min in PBS with 5 mM ethylenediaminetetraacetic acid [EDTA] and 0.1% BSA at 37°C with shaking) sequentially to yield a total of four LiberaseTM digestions and three EDTA incubations. Cells were collected after each digestion/incubation and saved as above to produce a total of 7 fractions. Fractions were pooled, filtered through a 70 μm filter, collected by centrifugation (300×*g* for 10 min), and resuspended in less than 1 mL of PBS with 0.5% BSA and 2 mM EDTA.

To isolate periosteal cells, femurs from mice (distinct from the ones used to collect endosteal cells) were cleaned of muscle without damaging the periosteum. Intact femurs were next placed in a 50 mL tube and digested with 2 Wunsch units of LiberaseTM in HBSS for 20 min at 37°C with shaking. The cells released by the digestion were collected in a tube by pipetting and placed on ice. The bones were next incubated with 5 mM EDTA and 0.1% BSA for 20 min at 37°C with shaking. The released cells were collected and placed on ice. This process was repeated sequentially to yield a total of three LiberaseTM digestions and three EDTA incubations. After all incubations, cells were pooled, filtered through a 70 μm filter, pelleted (300×*g* for 10 min), and resuspended in 200 μL PBS with 0.5% BSA and 2 mM EDTA.

Endosteal or periosteal cell preparations were further enriched in mesenchymal cells by depletion of hematopoietic and endothelial cells using a lineage depletion kit (Miltenyi Biotec, Catalog # 130‐090‐858) followed by CD45, CD117, and CD31 microbeads (Miltenyi Biotec, Catalog # 130‐0520301, 130‐091‐224, and 130‐097‐418) according to the manufacturer's instructions.

### 2.3 10X Genomics Sequencing

Single cells isolated from digested bones were encapsulated using a Chromium Controller (10X Genomics), and libraries were constructed using a Chromium Single Cell 3′ Reagent Kit (10X Genomics). The libraries were then sequenced using the Illumina NovaSeq 6000 machine to generate fastq files.

### 2.4 Bioinformatic Analysis of Single‐Cell RNA‐Sequencing

**FIGURE 1:**
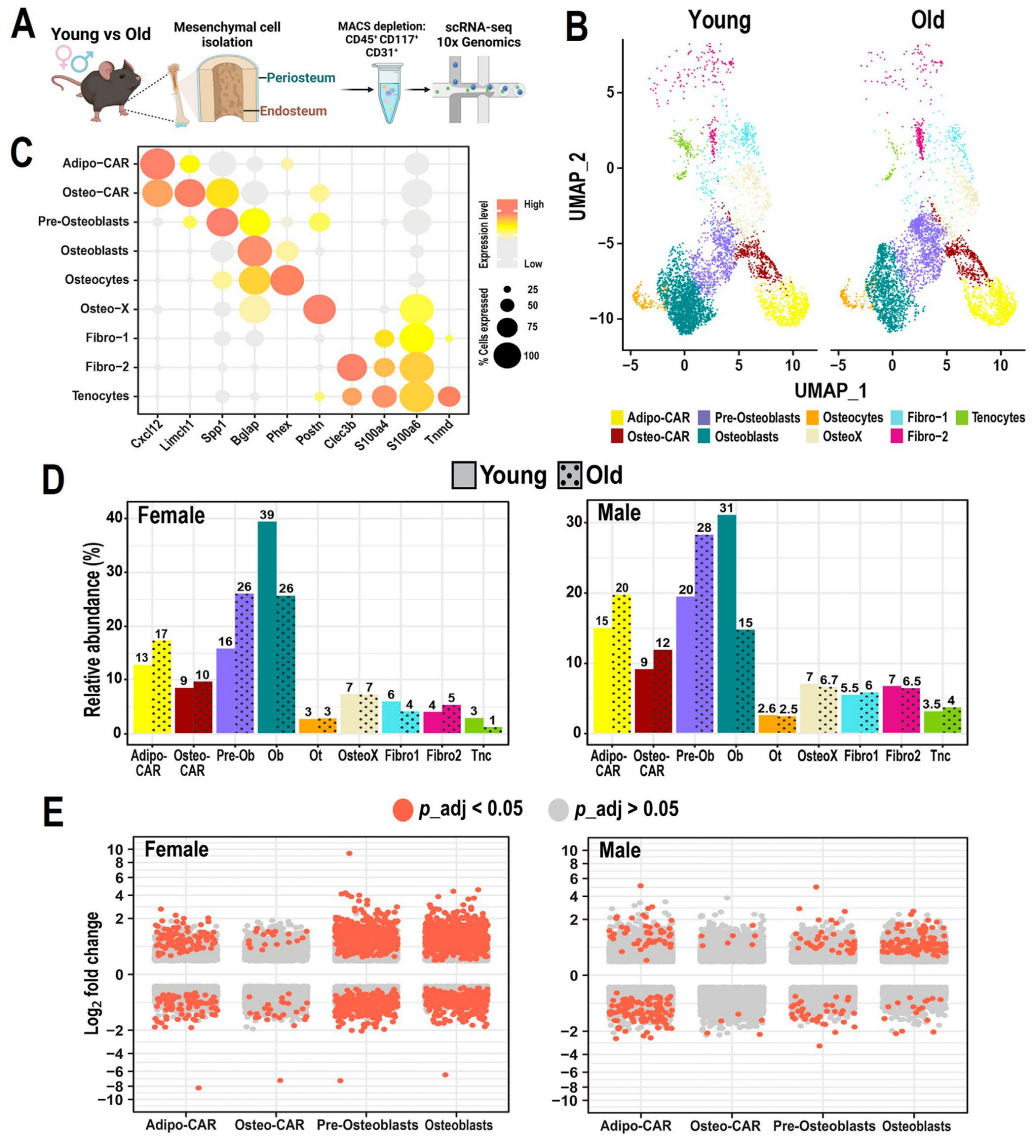
Aging affects the bone mesenchymal transcriptome of females more profoundly than males. (A) Endosteal or periosteal cells were obtained from the bones of 6 or 24 months C57Bl/6J female and male mice, enriched in mesenchymal lineage cells, and subjected to single‐cell RNA‐seq using the 10X Chromium platform. (B) UMAP visualization of mesenchymal cell clusters obtained from endosteum and periosteum. (C) Dot plot of representative marker genes for each mesenchymal cell cluster. Dot size indicates the percentage of cells expressing each gene, and the color gradient indicates expression levels. (D) Relative abundance of cells in endosteal preparations from young and old wild‐type females and males. Exact percentages are indicated above each bar. Solid bars = young, dotted bars = old. (E) Volcano plots of genes significantly up‐ or down‐regulated in each cluster, using the MAST with a random effect for the sample of origin. UMAP, uniform manifold approximation and projection; MAST, model‐based analysis of single‐cell transcriptomics.

The fastq files were preprocessed using Cell Ranger software version 7.1 (10X Genomics) to produce feature‐barcode matrices. The alignments were performed using the mouse reference genome mm10. The feature‐barcode matrices were imported for further analysis in R suite software using the Surat package version 4.2.0 (Hao et al. 2021). The harmonization between samples was performed using a reciprocal Principal Component Analysis method based on the top 50 principal components and 6000 most variable features to minimize the batch effect. The harmonized results were used for clustering using the Louvain algorithm with multilevel refinement and Uniform Manifold Approximation and Projection (UMAP) for dimension reduction. The gene‐specific markers of individual clusters and differentially expressed genes were identified for different cell types using the Model‐based Analysis of Single‐cell Transcriptomics (MAST) algorithm for cell type identification (Finak et al. 2015). Chondrocytes, most likely originating from remnants of the growth plate, were excluded from our analysis. The other nine mesenchymal cell types analyzed adipo‐CAR, osteo‐CAR, pre‐osteoblasts, osteoblasts, osteocytes, osteo‐X, fibro‐1, fibro‐2, and tenocytes are illustrated in Figure 1B,C. Gene Set Enrichment Analysis (GSEA) analyses were performed using the piano package (Varemo et al. 2013). The transcriptional activation analysis was performed using Single‐Cell Regulatory Network Inference and Clustering (SENIC) pipeline software (Aibar et al. 2017).

### 2.5 In Situ Hybridization

Sample preparation for in situ hybridization using RNAscope technology was performed under RNase‐free conditions. Right femurs from old and young wild‐type C57BL/6J mice were quickly excised and fixed in Millonig's buffer for 36 h at 4°C, followed by decalcification in 14% EDTA (pH 7.4) for 1 week at 4°C. Dehydrated femurs were embedded in paraffin, and 8 μm sagittal sections were probed for *Limch1* (Catalog # 591801), *Cdkn2a* (Catalog # 411011), *Angpt4* (Catalog # 476671), and *Postn* (Catalog # 418581) using the RNAscope 2.5 HD Reagent Red (Catalog # 322360) kit according to the manufacturer's instructions (Advanced Cell Diagnostics, Newark, CA). These genes, with the exception of *Cdkn2a*, were selected for in situ hybridization based on their specific as well as high expression in cells of the mesenchymal lineage. Because *Cdkn2a* is expressed by hematopoietic cells, we examined *Cdkn2a* in osteocytes due to the localization of these cells within the bone matrix.

### 2.6 Ex Vivo Osteoblastic Cell Culture

Bone marrow stromal cells (BMSCs) were flushed from the tibias and femurs of 6‐month‐old or 24‐month‐old C57BL/6 wild‐type mice using an isolation medium (HBSS supplemented with 10% fetal bovine serum [FBS] and 1% penicillin–streptomycin‐glutamine [PSG]). Cells from four mice per group were pooled and cultured in alpha minimum essential medium (α‐MEM) (Sigma) supplemented with 20% FBS (Sigma), 1% PSG (Sigma), and 50 μg/mL ascorbic acid (Sigma) in 10 cm culture dishes for 5–7 days. Half of the medium was replaced every 3 days. Adherent BMSCs were then re‐plated in triplicate into 12‐well plates at a density of 2 × 10^5^ cells per well. Cells were cultured in a medium containing ascorbic acid and 10 mM β‐glycerophosphate (Sigma) for 3 days to prepare samples for western blot assays.

### 2.7 Western Blot Analysis

Cultured cells were washed twice with ice‐cold PBS and lysed in a buffer containing 20 mM Tris–HCl, 150 mM NaCl, 1% Triton X‐100, a protease inhibitor mixture, and a phosphatase inhibitor cocktail (Sigma‐Aldrich) on ice for 30 min. The cell lysates were then centrifuged at 13,000 rpm for 10 min at 4°C, and the supernatants were collected into fresh tubes. The protein concentration in the lysates was determined using the Bio‐Rad DC protein assay kit. For western blotting, 20 μg of protein per sample was separated by 10% SDS‐PAGE and transferred electrophoretically to polyvinylidene fluoride membranes (Merck Millipore). The membranes were blocked in 5% fat‐free milk/Tris‐buffered saline for 120 min, followed by incubation with primary antibodies, and subsequently with horseradish peroxidase‐conjugated secondary antibodies. MAPKs and Akt signaling levels in the cell lysates were determined using the following antibodies: mouse monoclonal antibodies for p‐Erk (Santa Cruz Biotechnology sc‐7383, 1:500) and p‐Jnk (Cell Signaling no. 9255, 1:1000), rabbit monoclonal antibodies for p‐p38 (Cell Signaling no. 9215, 1:1000) and p‐Akt (Cell Signaling no. 4058, 1:1000), rabbit polyclonal antibodies for Erk (Santa Cruz Biotechnology sc‐94, 1:500), p38 (Cell Signaling no. 9212, 1:1000), and Akt (Cell Signaling no. 9272, 1:1000), and mouse monoclonal antibody for Jnk (Santa Cruz Biotechnology sc‐1648, 1:500). The membranes were subjected to western blot analysis using enhanced chemiluminescence reagents (Millipore), and images were captured with a VersaDoc imaging system (Bio‐Rad). All blots shown in each figure were derived from the same experiment and processed in parallel.

### 2.8 Senescence‐Associated β‐Galactosidase Staining

BMSCs were flushed from the tibias and femurs of mice using complete media (α‐MEM, 10% FBS, 1% PSG). Red blood cells were removed using ammonium‐chloride‐potassium buffer (0.01 mM EDTA, 0.011 M KHCO3, and 0.155 M NH4Cl, pH 7.3). The remaining cells were cultured with osteogenic media (α‐MEM, 10% FBS, 1% PSG, and 50 μg/mL ascorbic acid) and incubated at 37°C and 5% CO_2_. After cells reached 80% confluency (5–10 days), cells were re‐plated in 6‐well plates at 2.5 × 104 cells/cm^2^. When cells reached 60%–80% confluency, senescent cells were detected via senescence‐associated β‐Galactosidase (SA‐β‐gal) staining as described (Itahana et al. 2013). Briefly, cells were fixed with 4% paraformaldehyde for 5 min at room temperature. Then, cells were washed twice with PBS and incubated with SA‐β‐gal staining buffer (Citric acid/sodium phosphate buffer [pH 6.0], 5 mM potassium ferricyanide, 5 mM potassium ferrocyanide, 150 mM NaCl, 2 mM MgCl2, 1 mg/mL X‐Gal) overnight at 37°C in a dry incubator. Finally, cells were washed twice with PBS and imaged with a bright‐field microscope.

### 2.9 RNA Isolation and Gene Expression Analysis

Femur shafts of 4‐month‐old Osx1‐Cre and Atg7^f/f^; Osx1‐Cre mice were dissected and cleaned of soft tissue. The epiphyses were removed, and the marrow was flushed. The cleaned femur cortical bones were snap‐frozen in liquid nitrogen and stored at −80°C. For RNA isolation, bones were homogenized in Trizol Reagent (Life Technologies, Catalog # 15596018), and RNA was isolated using the RNAeasy Plus Mini Kit (Qiagen, Catalog # 74136) according to the manufacturer's instructions. 1 μg RNA was used to synthesize cDNA with a High‐Capacity cDNA Reverse Transcription Kit (Applied Biosystems, Catalog #4368814). Relative mRNA levels were measured using multiplex quantitative real‐time PCR (qRT‐PCR) with TaqMan Fast Advanced Master Mix (Applied Biosystems, Catalog # 4444964), FAM‐labeled TaqMan gene expression assays indicated below (Life Technologies), and VIC‐labeled mouse Actb (β‐actin) (Applied Biosystems, Catalog # 4352341E). The following FAM‐labeled assays were used in the gene expression analysis: Bnip3 (Mm01275600_g1), Ccnd1 (Mm00432359_m1), Map1lc3b (Mm00782868_sH), Timp2 (Mm00441825_m1), Tgfb2 (Mm00436955_m1), Serpine2 (Mm00436753_m1), Plat (Mm00476931_m1), Fn1 (Mm01256744_m1), Mmp2 (Mm00439498_m1), Mmp13 (Mm00439491_m1), Igfbp4 (Mm00494922_m1), Igfbp5 (Mm00516037_m1), Igfbp7 (Mm03807886_m1). The relative mRNA levels were calculated using the comparative cycle threshold (ΔCt) method (Livak and Schmittgen 2001).

### 2.10 Mitochondrial DNA Isolation and Analysis

For DNA isolation, cortical bone was cleaned as above, and bones devoid of marrow were cut into pieces and decalcified in 14% EDTA for 1 week. Decalcified cortical bone fragments were digested with proteinase K (0.5 mg/mL in 10 mm Tris, pH 8.0, 100 mm NaCl2, 20 mm EDTA, and 1% SDS) at 55°C overnight. DNA was isolated by phenol/chloroform extraction and ethanol precipitation. For mitochondrial DNA quantification, the following custom Taqman assay for the mitochondrial gene ND2 was utilized: forward, 5′‐CATGACAAAAAATTGCTCCCCTATCAA‐3′, reverse, 5′‐ATGCCCCTATGAAAATAGAAGTAATTGCT‐3′, probe, 5′‐FAM‐CCCGCTACTCAACTCT‐NFQ‐3′. The amount of mitochondrial DNA was calculated using results from the ND2 and Tfrc (Applied Biosystems, Cat # 4458367) copy number reference assays and the (ΔCt) method (Livak and Schmittgen 2001).

### 2.11 Statistical Analysis

All values are reported as mean ± standard deviation (STD). Differences between the two groups in Figures 5 and 6 were evaluated using Graph Pad Prism 7.05 software (GraphPad Software Inc., La Jolla, CA, USA). Data were analyzed by student's *t*‐test.

## 3 Results

### 3.1 An Aging Mouse Bone Mesenchymal Cell Atlas by scRNA‐Seq

To examine the transcriptional changes induced by aging in the mesenchymal cells of bone, we isolated cells from male and female 6‐ and 24‐month‐old C57BL/6J mice using a previously established protocol (Nookaew et al. 2024). Separate mice were used to isolate endosteal versus periosteal cells by serial digestions. Isolated cells were enriched for mesenchymal lineage cells by removal of hematopoietic and endothelial cells and subjected to scRNA‐seq using the 10X Chromium platform (Figure 1A, Figure S1). Each scRNA‐seq experiment was performed with pooled cells isolated from 2 to 4 mice per group (Table S1). Periosteal cells were analyzed separately and are described in Figure 7.

### 3.2 The Proportion of Pre‐Osteoblasts in the Endosteal Surface Does Not Decrease With Aging

Clustering analysis with the Louvain algorithm revealed distinct clusters representing the major mesenchymal cell types at the endosteum, illustrated in uniform manifold approximation and projection (UMAP), identified based on the expression of established markers (Figure 1B,C). The most abundant cell types in the endocortical samples from both young and old mice were osteoblasts, pre‐osteoblasts, osteo‐CAR cells, and adipo‐CAR cells (Figure 1D, Table S1). As previously described, osteoblasts were defined by high expression of *Bglap* and *Col1a1*, pre‐osteoblasts by *Spp1* and *Mmp13*, osteo‐CAR by *Limch1* and *Angpt4* expression, and Adipo‐CAR cells by *Cxcl12* and *Adipoq* (Figure 1C) (Nookaew et al. 2024). These cell preparations also included a small number of osteocytes, identified by expression of *Phex* and *Sost*. Despite osteocytes being abundant in bone, they were not obtained in large numbers (Table S1), likely due to their inefficient release from the calcified bone matrix. Thus, examination of the osteocyte transcriptome was performed after pooling all these cells from the female and male experiments. The presence of low numbers of fibro‐1, fibro‐2, and tenocytes in the endosteal preparations most likely resulted from remnants of the periosteum. Indeed, we have previously used in situ hybridization to show that fibro‐1, fibro‐2, and tenocytes reside mostly, if not exclusively, on the periosteum (Nookaew et al. 2024).

We first examined whether aging was associated with changes in the relative abundance of any of the endosteal clusters. In both sexes, osteoblasts exhibited the most significant reduction in abundance with age (Figure 1D, Figure S1). This reduction is consistent with previous histomorphometric findings demonstrating that osteoblast number and bone formation rate decrease with age in remodeling trabecular and endocortical bone (Almeida et al. 2007; Ferguson et al. 2003; Ucer et al. 2017). Unexpectedly, the proportion of pre‐osteoblasts showed a consistent increase with aging, suggesting that the differentiation of these cells is compromised, or that the factors that promote their differentiation are diminished with aging. In both young and old mice, pre‐osteoblasts localize to the endosteal and trabecular bone surfaces (Figure S2) (Nookaew et al. 2024).

We next investigated the changes in gene expression that occur with age in the major cell types of the endosteum of female and male mice by performing differential expression analysis using the model‐based analysis of single‐cell transcriptomics (MAST), with a random effect for the sample of origin. Female pre‐osteoblasts and osteoblasts showed a much greater number of differentially expressed genes (DEGs) with aging than those from males (Figure 1E, Tables S2 and S3). In both males and females, osteo‐CAR cells showed the least change in gene expression. Likewise, osteocytes showed few changes in gene expression (Figure S3).

### 3.3 Aging Decreases the Osteoblastic Gene Signature and Osteoblast Differentiation Pathways

**FIGURE 2:**
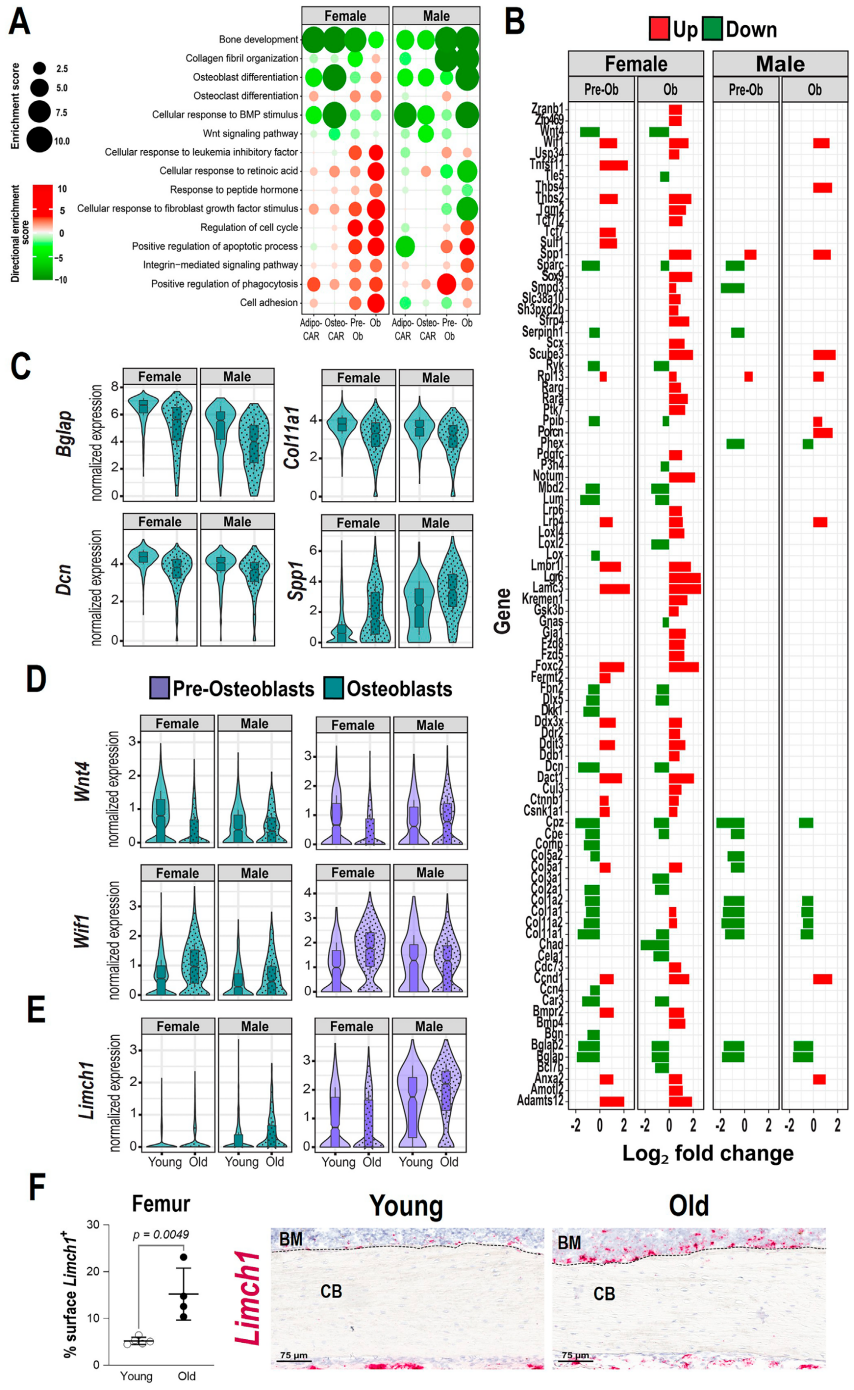
Extracellular matrix and osteoblast differentiation genes decrease with aging. (A) Biological processes determined by Gene Ontology (GO) analysis up‐regulated (red) or down‐regulated (green) with aging in endosteal mesenchymal cell clusters of females and males. Larger circle sizes and darker colors indicate higher significance. (B) Increased (red) or decreased (green) differentially expressed genes with aging (*p*_adj_ < 0.05) in Pre‐Osteoblasts and Osteoblasts related to bone development, cellular response to BMP, collagen fibril organization, and Wnt signaling pathway. (C–E) Violin plots of the expression of indicated genes in Osteoblasts (C) and in Osteoblasts and Pre‐Osteoblasts (D) from endosteum of female and male mice. (F) Quantification of the bone surface positive for *Limch1* normalized to total bone surface (left) and representative images of *Limch1* expression (red) on the endosteal surface of femur (right) from 6 and 24‐month‐old female mice, by in situ hybridization. *n* = 4–5 mice/group (images from all mice are shown in Figure S3); BM = bone marrow, CB = cortical bone. Bars represent mean ± SD.

To examine the biological processes that are affected by aging in the different cell types, we performed gene set enrichment analysis (GSEA) within each gene set. Because there were multiple biological processes altered with aging, we presented these biological pathways by major themes, in Figures 2 through 5, for simplicity.

Many gene ontology (GO) terms related to the osteoblast phenotype were down‐regulated with age in both female and male mice (Figure 2A). Expression of markers of osteoblast differentiation such as *Bglap*, *Bglap2*, *Car3*, *Col1a1*, and *Col1a2* were decreased, as were other extracellular matrix (ECM) genes such as *Coll11a1*, *Sparc*, *Lum*, *Dcn*, and *Fbn2* (Figure 2B,C). These changes occurred within the osteoblast and pre‐osteoblast clusters (Figure 2B,C). In contrast, other ECM genes were increased with aging including *Spp1*, *Lamc3*, *Thbs2*, and *Thbs4* (Figure 2B,C). These changes suggest that matrix composition could be altered in old mice and contribute to the age‐related decrease in bone strength observed in both females and males (Almeida et al. 2007).

Moreover, genes that regulate the cell cycle and the apoptotic process were also increased in osteoblasts and pre‐osteoblasts in both males and females (Figure 2A). An increase in TUNEL‐positive osteoblast with aging was previously described (Jilka et al. 2010). Wnt and BMP signaling is indispensable for osteoblastogenesis (Baron and Kneissel 2013; Lowery and Rosen 2018). Several inhibitors of the Wnt pathway, such as *Lrp4*, *Wif*, *Kremen1*, *Notum*, and *Sfrp4*, were increased with aging, particularly in females. A decrease in *Wnt4* was also seen in females (Figure 2B,D). These changes may contribute to the decrease in osteoblasts and the accumulation of pre‐osteoblasts with age.

We also noted that several of the cell identity marker genes of the different clusters were altered with age. Besides the decrease in *Bglap*, *Car3*, and *Col1a1* in osteoblasts, marker genes of pre‐osteoblasts such as *Spp1*, *Mmp13*, and *Vdr* (Nookaew et al. 2024) were increased in the osteoblast cluster (Table S2). Similarly, both *Wif1* and *Limch1*, markers of the osteo‐CAR cluster, were increased in pre‐osteoblast and osteoblast clusters (Figure 2D,E). An increase in *Limch1* expression at the endosteal surface of the femur was confirmed by RNA in situ hybridization (Figure 2F, Figure S3). This aging‐associated identity noise has also been observed in old skin fibroblasts and is associated with pro‐adipogenic traits (Salzer et al. 2018).

Osteocytes produce factors that modulate both bone formation and resorption, such as Sost, Dkk1, and Rankl, and some of these factors change with age in whole bone extracts (Chermside‐Scabbo et al. 2024; Piemontese et al. 2017). However, we did not detect changes in mRNA levels of these factors in osteocytes from aged mice (Figure S2).

### 3.4 Endosteal Cells From Old Mice Exhibit Cellular Senescence and Markers of Inflammatory Response

**FIGURE 3:**
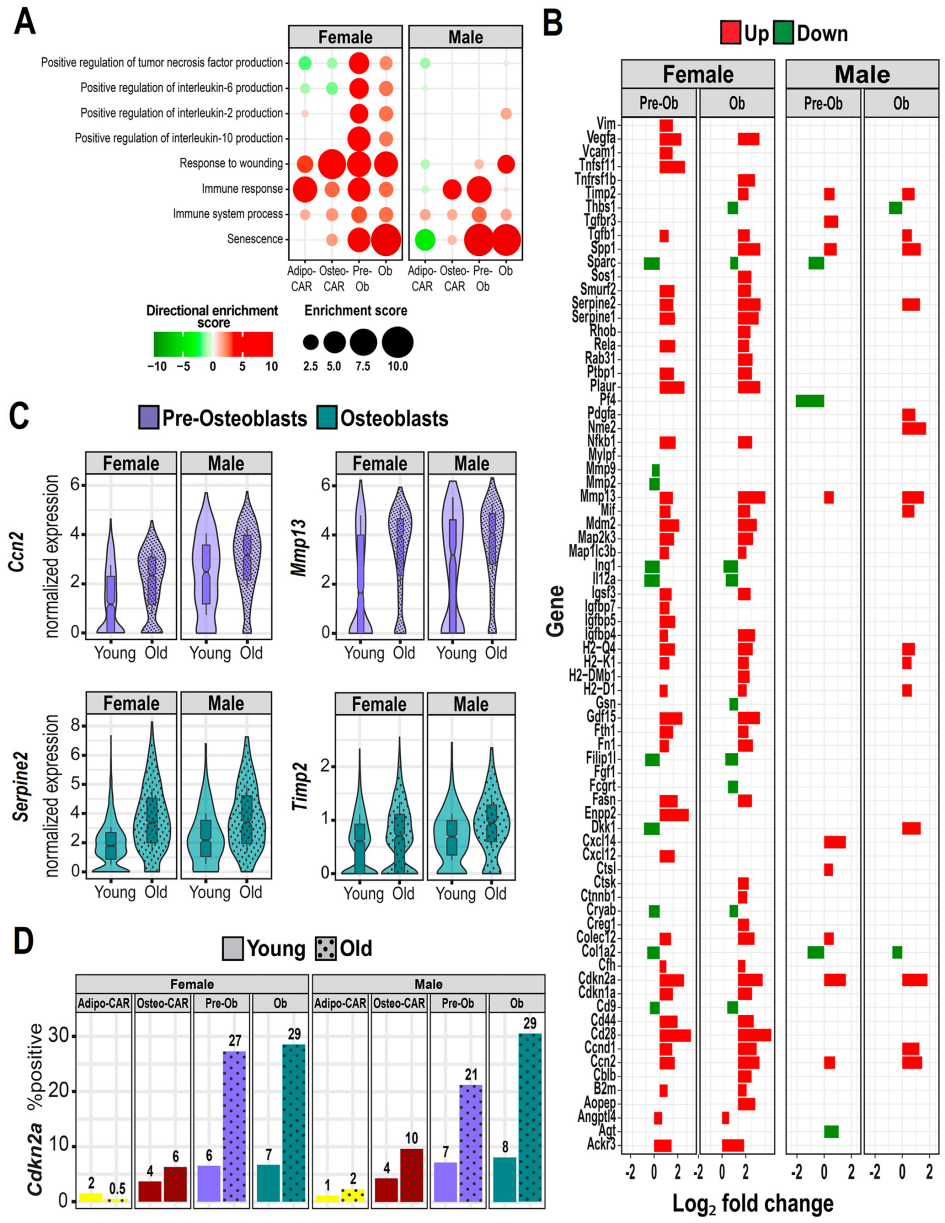
Markers of inflammation and senescence increase with aging in endosteal mesenchymal cells. (A) Biological processes determined by Gene Ontology (GO) analysis up‐regulated (red) or down‐regulated (green) with aging in endosteal mesenchymal cell clusters of females and males. Larger circle sizes and darker colors indicate higher significance. (B) Increased (red) or decreased (green) differentially expressed genes with aging (*p*_adj_ < 0.05) in Pre‐Osteoblasts and Osteoblasts related to senescence and immune response. (C) Violin plots of the expression of the indicated genes in Osteoblasts and Pre‐Osteoblasts from endosteum of female and male mice. (D) Bar graphs depicting the percentage of *Cdkn2a* positive cells within the indicated cell clusters. Exact percentages are indicated above each bar.

Cellular senescence is a process that is triggered in response to stress or damage and is characterized by a stable cell cycle arrest and multiple intracellular phenotypic changes, including the expression of a senescence‐associated secretory phenotype (SASP), which comprises cytokines, chemokines, and other pro‐inflammatory factors (Suryadevara et al. 2024). Previous findings obtained with isolated cells and in situ analysis show that senescence markers increase in osteoblastic cells from aged mice (Farr et al. 2016; Kim et al. 2017). We constructed a GO term for senescence that includes the transcripts previously described in the Fridman, SenMayo, and SenNet gene sets (Fridman and Tainsky 2008; Saul et al. 2022; Suryadevara et al. 2024). An increase in biological processes related to inflammatory response and senescence was seen in cells from both females and males with aging (Figure 3A). Pre‐osteoblasts and osteoblasts exhibited the most significant increase in senescence‐related genes including *Cdkn2a*, *Ccnd1*, *Ccn2*, *Mmp13*, *Serpine2*, *Spp1*, *Mif*, *Timp2*, and *Tgfb1* (Figure 3B,C). In line with these findings, the proportion of cells expressing *Cdkn2a* (also known as p16^INK4A^) was increased 3–4 fold in pre‐osteoblasts and osteoblasts (Figure 3D). Single‐cell CyTOF studies using a mixture of mesenchymal cell populations isolated from combined male and female mouse bone also showed a 3‐fold expansion in p16 positive cells with aging (Doolittle et al. 2023). RNA in situ hybridization revealed an increase in the number of osteocytes expressing *Cdkn2a* in femur cortical bone from old mice (Figure S5). In addition, many *Cdkn2a*‐positive cells were present on the bone surface within intracortical pores (Figure S5). Females, but not males, had increased expression of other senescence‐related genes such as *Cdkn1a*, *Cd44*, *Gdf15*, and *Igfbp4* (Figure 3B).

Pre‐osteoblasts and osteoblasts from females also exhibited upregulation of pro‐inflammatory transcription factors, including *Rela* and *Nfkb*, as well as the scavenger receptor *Colec12* and chemokine receptor *Ackr3* (*Cxcr7*) (Figure 3B). Stromal cells and adipocytes are thought to produce inflammatory cytokines such as IL1α, IL1β, CCL2, and TNFα, thereby contributing to the aging of the bone marrow niche (Cain et al. 2024). Nonetheless, we found no age‐related changes in the expression of these inflammatory cytokines in any of the mesenchymal cell clusters (Tables S2 and S3). In contrast to pre‐osteoblasts and osteoblasts, adipo‐CAR and osteo‐CAR cells did not show elevated *Cdkn2a*, *Cdkn1a*, or SASP (Tables S2 and S3), suggesting that these cells are better protected from the stresses that cause senescence.

### 3.5 Aging Affects Cellular Metabolism and Upregulates the Response to Hypoxia in Female Pre‐Osteoblasts and Osteoblasts

**FIGURE 4:**
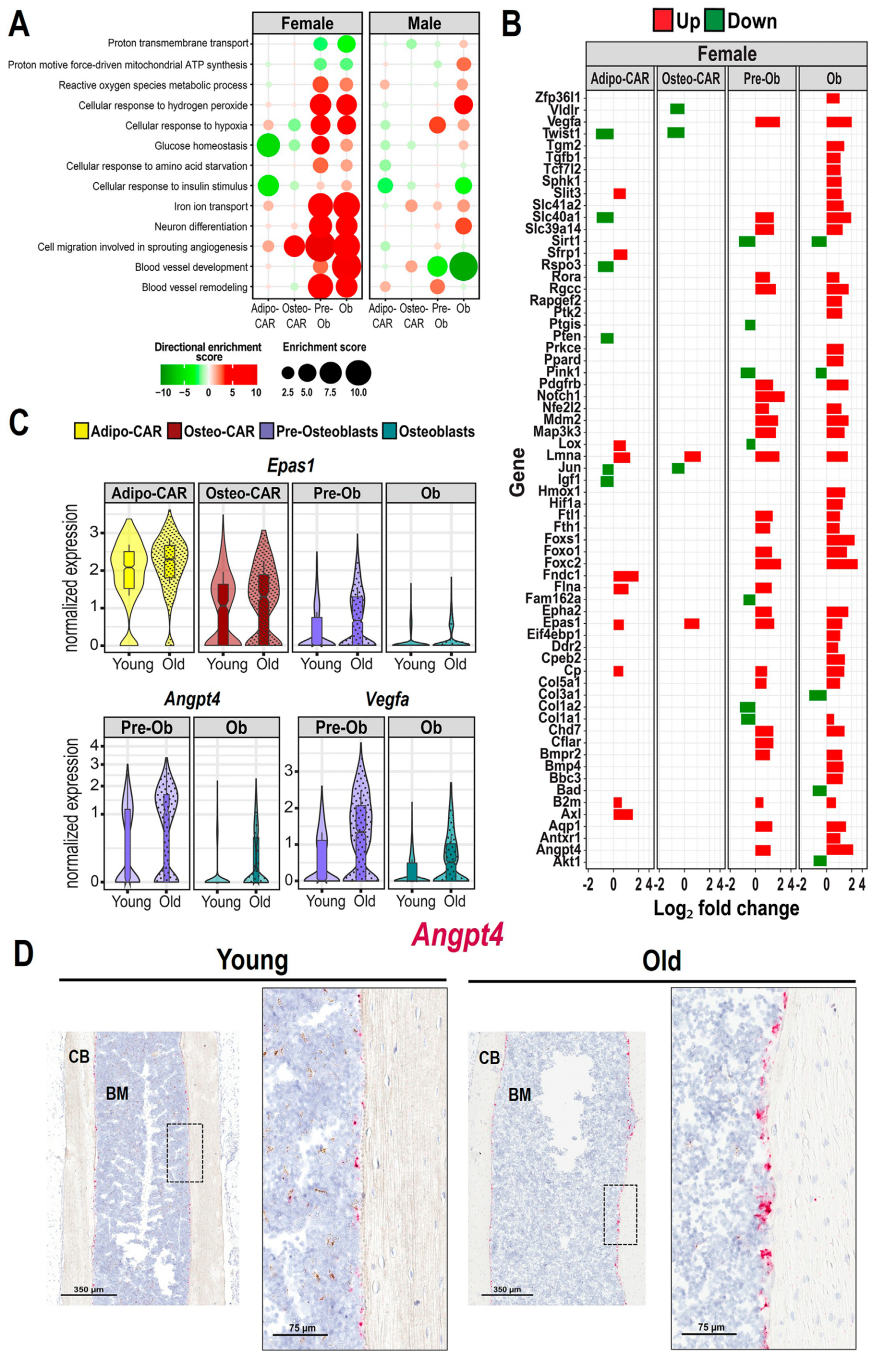
Aging increases the expression of iron metabolism‐ and angiogenesis‐related genes particularly in females. (A) Biological processes determined by Gene Ontology (GO) analysis up‐regulated (red) or down‐regulated (green) with aging in endosteal mesenchymal cell clusters of females and males. Larger circle sizes and darker colors indicate higher significance. (B) Increased (red) or decreased (green) differentially expressed genes with aging (*p*_adj_ < 0.05) in the indicated cell clusters related to blood vessel development and remodeling, cellular response to hypoxia, and iron ion transport. (C) Violin plots of the expression of the indicated genes in Osteoblasts and Pre‐Osteoblasts from endosteum of female mice. (D) Representative image of *Angpt4* expression (red) on the endosteal surface of femur from 6 and 24‐month‐old female mice by in situ hybridization. BM = bone marrow, CB = cortical bone.

Our analysis revealed that aging deregulated gene expression patterns related to nutrient‐sensing and metabolism and mitochondrial dysfunction, particularly in females (Figure 4A). Specifically, markers for cellular response to insulin signaling and glucose homeostasis were decreased in adipo‐CAR cells (Figure 4A). The cellular response to hydrogen peroxide (H_2_O_2_) was increased in pre‐osteoblasts and osteoblasts along with decreased expression of genes involved in the proton motive force‐driven mitochondrial ATP synthesis (Figure 4A). These findings support the notion that mitochondria become dysfunctional in bone cells with age. Pre‐osteoblasts and osteoblasts also exhibited an upregulated response to amino‐acid starvation, hypoxia, angiogenesis, and iron ion transport, particularly in female mice (Figure 4A and Figure S5). Genes related to hypoxia and angiogenesis included *Vegfa*, *Angpt4*, *Hif1*α, *Epas1* (*Hif2α*), *Epha2*, and *Slit3* (Figure 4B,C). RNA in situ hybridization revealed elevated expression of *Angpt4* in the endosteal bone surface of aged female mice, in samples obtained from independent biological replicates (Figure 4D). As blood vessels are essential components of bone remodeling units, the increase in pro‐angiogenic factors may contribute to the intracortical remodeling observed in old female mice (Piemontese et al. 2017).

### 3.6 Transcription and Phosphorylation Are Increased While Proteostasis Is Decreased With Aging

**FIGURE 5:**
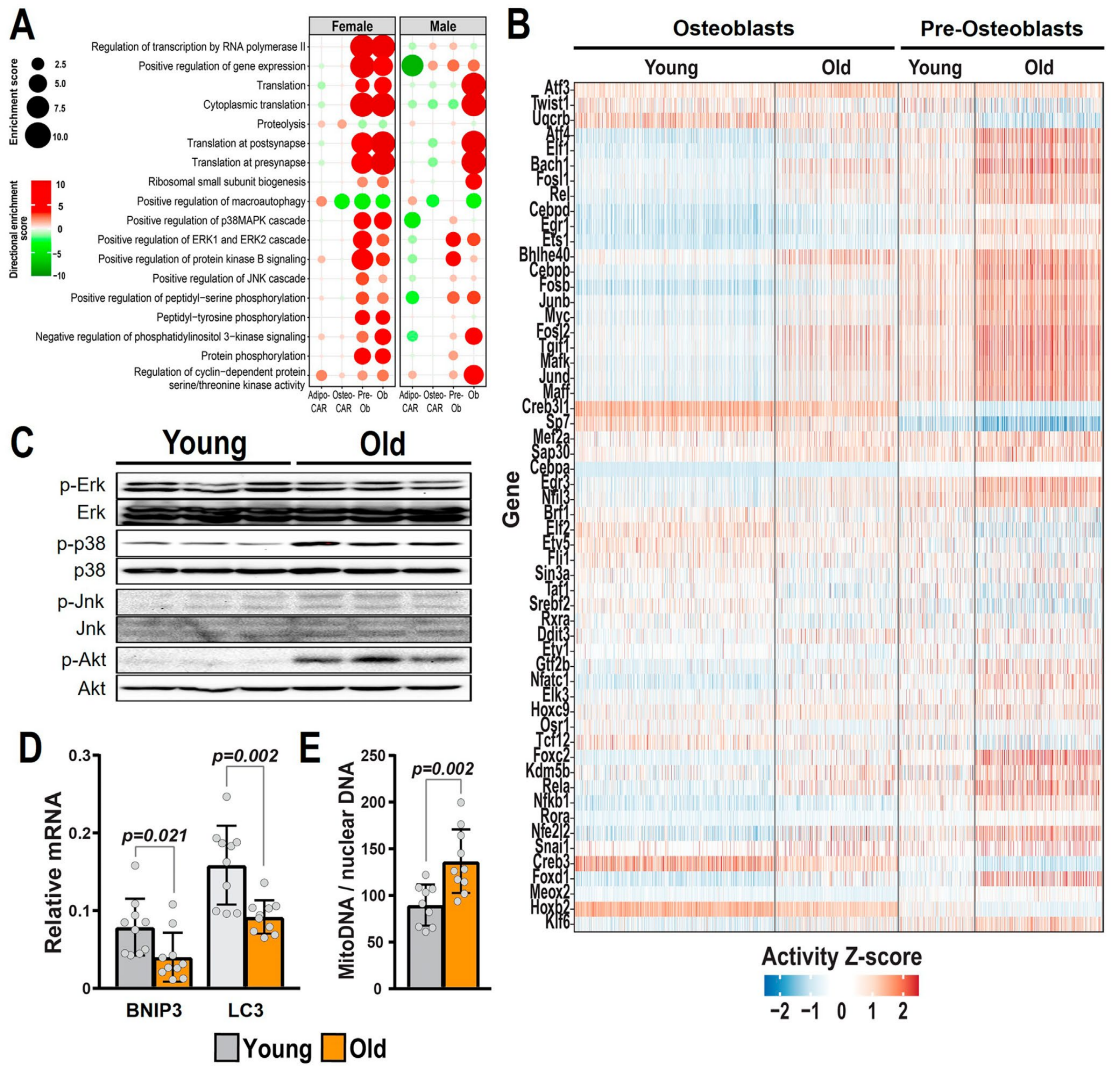
Transcriptional activity and MAPK phosphorylation are increased. (A) Biological processes determined by Gene Ontology (GO) analysis up‐regulated (red) or down‐regulated (green) with aging in endosteal mesenchymal cell clusters of females and males. Larger circle sizes and darker colors indicate higher significance. (B) SCENIC analysis of transcription factor activity in Osteoblasts and Pre‐Osteoblasts from females. (C) Immunoblot analysis of Erk, p38, Jnk, and Akt phosphorylation in osteoblastic cell cultures from 6 to 24‐month‐old females (*n* = 3 mice/group). (D) Autophagy‐related genes measured by quantitative real‐time PCR. (E) Mitochondrial DNA normalized to genomic DNA in cortical bone from female mice (*n* = 5–10 mice/group). Bars represent mean ± SD, *p*‐values by Student's *t*‐test. SCENIC, single‐cell regulatory network inference and clustering.

Our comparative transcriptome results also indicated that pre‐osteoblasts and osteoblasts from aged females had increased biological processes related to transcription (Figure 5A). Expression of many transcription factors implicated in stress responses such as *Atf4*, *Egr3*, *JunD*, *Fosb*, *Fosl2*, *Rela*, and *Nfkb1* was upregulated in osteoblasts and pre‐osteoblasts from old female mice (Figure S6). We used the single‐cell regulatory network inference and clustering (SENIC) approach to infer the transcription factor activity of individual cells across the cell clusters. This analysis suggested that the activity of the transcription factors above was increased while the activity of *Sp7*, *Creb3*, *Creb3l1*, and *Hoxb2*, among others, was decreased with aging (Figure 5B).

Strikingly, protein phosphorylation GO terms were strongly increased in pre‐osteoblasts and osteoblasts (Figure 5A). We used protein lysates from cultured bone marrow stromal cells from young and old female mice to examine the phosphorylation status of mitogen‐activated protein kinases (MAPKs), extracellular signal‐regulated kinases (Erks), c‐Jun N‐terminal kinases (JNKs), p38, and protein kinase B (PKB/Akt). Phosphorylation of p38 and Akt was greatly increased in samples from old mice (Figure 5C), an effect that is in line with the activation of the transcription factors described above. Accordingly, the expression of many genes encoding large and small ribosomal protein subunits implicated in translation was upregulated in cells from both female and male mice (Figure 5A, Figure S7). These findings suggest that exacerbated stimulation of Akt, p38 MAPK, and other kinases might be responsible for the dysregulated transcriptional program in female osteoblasts.

Another common mechanism of aging is the loss of protein homeostasis (proteostasis). Protein homeostasis is maintained by protein quality control mechanisms, which ensure the degradation of damaged and misfolded proteins. The autophagy‐lysosome system plays a key role in the elimination of damaged proteins and organelles, including mitochondria. Genes related to autophagy were decreased in most cell clusters with aging (Figure 5A). We also examined the expression of *Bnip3* (an autophagy receptor) and *Map1lc3* (*LC3*, an essential component of autophagosomes) (Hanna et al. 2012) in cortical bone shafts of long bones and found that the expression of both genes was decreased in old mice (Figure 5D). In line with a decrease in the turnover of mitochondria by autophagy, the amount of mitochondrial DNA relative to nuclear DNA, an indicator of the number of mitochondria in the tissue, was increased in bone from old mice (Figure 5E).

### 3.7 Attenuation of Autophagy Promotes Senescence in Mesenchymal Lineage Cells

**FIGURE 6:**
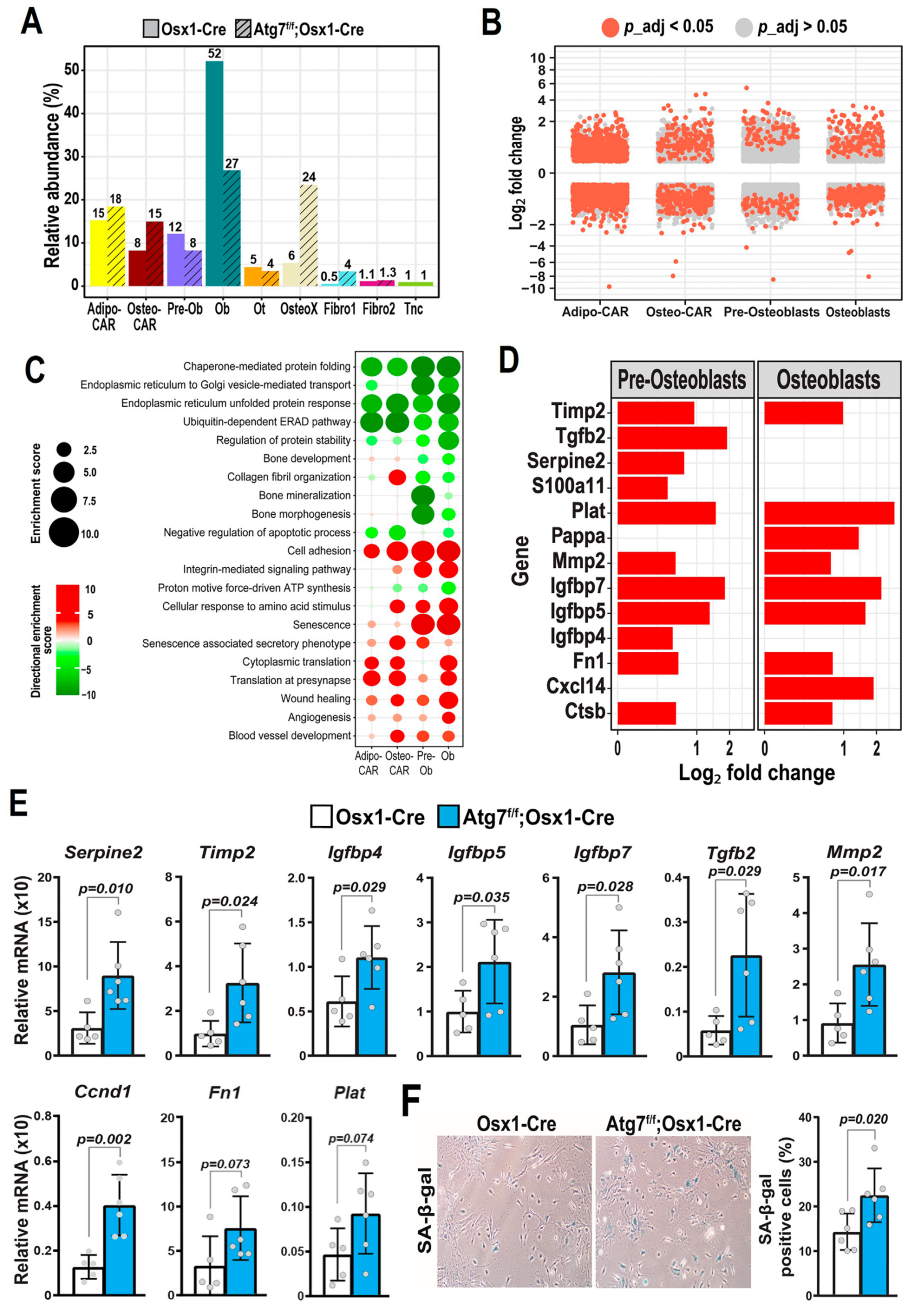
Deletion of Atg7 in osteoblast lineage cells disrupts protein processing and increases senescence. (A–D) Endosteal cells from long bones were isolated from 3‐month‐old male Atg7^f/f^; Osx1‐Cre mice or their Osx1‐Cre littermate controls, and subjected to single‐cell RNA‐seq. (A) Relative abundance of mesenchymal cell cluster obtained from Atg7^f/f^; Osx1‐Cre versus Osx1‐Cre mice. Exact percentages are indicated above each bar. (B) Volcano plots of up‐ and down‐regulated genes in each cluster using MAST with a random effect for the sample of origin. (C) Biological processes determined by Gene Ontology (GO) analysis up‐regulated (red) or down‐regulated (green) in response to *Atg7* deficiency in endosteal mesenchymal cells. Larger circle sizes and darker colors indicate higher significance. (D) Increased differentially expressed genes (*p*_adj_ < 0.05) in Pre‐Osteoblasts and Osteoblasts from Atg7^f/f^; Osx1‐Cre mice. (E) mRNA levels of senescence‐marker genes in cortical bone from 5‐month‐old Atg7^f/f^; Osx1‐Cre or Osx1‐Cre control mice as measured by qRT‐PCR. (F) Senescence‐associated β‐Galactosidase (SA‐β‐gal) activity measured in Atg7^f/f^; Osx1‐Cre and Osx1‐Cre stromal osteoblastic cultures (*n* = 6 wells/group). Bars represent mean ± SD, *p*‐values by student's *t*‐test. MAST, model‐based analysis of single‐cell transcriptomics.

**FIGURE 7:**
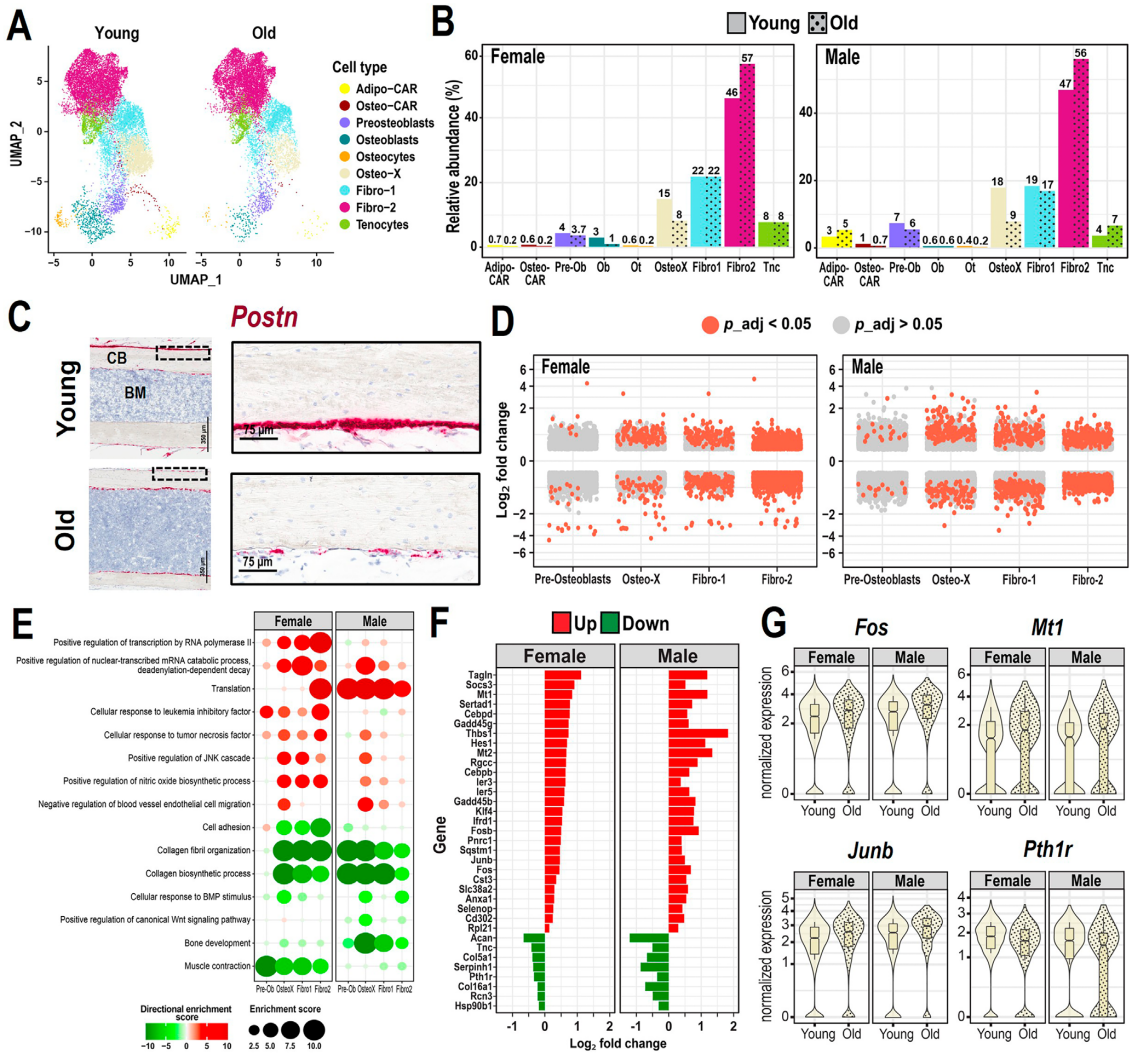
The proportion of Osteo‐X cells decreases with aging in the periosteum and is associated with increased stress response genes. (A) Relative abundance of cells in periosteal preparations from young and old wild‐type females and males. Exact percentages are indicated above each bar. (B) Volcano plots of up‐ and down‐regulated genes in each cluster using MAST with a random effect for the sample of origin. (C) Biological processes determined by Gene Ontology (GO) analysis up‐regulated (red) or down‐regulated (green) with aging in periosteal mesenchymal cell clusters of females and males. Larger circle sizes and darker colors indicate higher significance. (D) Bar graph and (E) violin plots of commonly regulated genes in female and male Osteo‐X cells with aging (*p*_adj_ < 0.05). (F) Representative images of *Postn* expression in bone from 6‐ and 24‐month‐old females by in situ hybridization. BM = bone marrow, CB = cortical bone. MAST, model‐based analysis of single‐cell transcriptomics.

The results thus far indicate that osteoblast lineage cells exhibit markers of many distinct mechanisms of aging, including a decline in macroautophagy. Aging mechanisms are often interconnected, but how these interact might be different depending on the cell type. We and others have previously shown that genetic inactivation of autophagy in the osteoblast lineage severely decreases osteoblast number and bone mass (Li et al. 2018; Liu et al. 2013; Nollet et al. 2014; Onal et al. 2013; Piemontese et al. 2016), resembling changes that occur with aging. To examine whether a decline in autophagy could be the cause of other aging‐associated mechanisms, we analyzed the transcriptomic changes caused by autophagy deficiency alone using endosteal cells from 3‐month‐old male Atg7^f/f^; Osx1‐Cre and Osx1‐Cre (control) mice. Because we observed evidence of decreased autophagy in endosteal cells from aged mice of both sexes (Figure 5A), but no such evidence in periosteal cells (Figure 7E), we limited our analyses to endosteal cells. The proportion of osteoblasts was significantly reduced in mice with *Atg7* deficiency when compared to Osx1‐Cre littermates (Figure 6A and Figure S8), in line with previous histological findings showing low osteoblast number and bone formation in these mice (Piemontese et al. 2016). In contrast, the proportion of osteo‐X was increased. Adipo‐CAR showed the greatest number of DEGs (Figure 6B). *Atg7* deletion decreased transcripts related to chaperone‐mediated protein folding, the endoplasmic reticulum (ER) unfolded protein response, and the ubiquitin‐dependent ER‐associated degradation pathway (ERAD) in all major cell clusters (Figure 6C). These results indicate dysregulation of protein homeostasis in these cell types. *Atg7* deletion also increased the expression of genes involved in translation, senescence, and angiogenesis (Figure 6C). The markers of senescence included the upregulation of *Ccnd1* and many SASP components, but not *Cdkn1a* and *Cdkn2a* (Figure 6D,E). Using bulk RNA from the cortical bone of Atg7^f/f^; Osx1‐Cre mice, we confirmed the upregulation of many senescence markers (Figure 6E). In addition, the number of senescence‐associated β‐galactosidase (SA‐β‐Gal) positive cells was higher in osteoblastic cell cultures from the same mice, lending support to the idea that autophagy deficiency promotes senescence (Figure 6F). Suppression of autophagy did not recapitulate all the effects of aging, such as, for example, an increase in pre‐osteoblasts. This was unsurprising as a decline in autophagy is just one of several mechanisms of aging. In addition, suppression of autophagy led to an increase in the proportion of osteo‐X cells and an exacerbated number of DEGs in Adipo‐CAR cells. These changes were not observed in aged mice, most likely because autophagy was not decreased with aging in these populations.

### 3.8 The Proportion of Osteo‐X Cells Decreases With Aging in the Periosteum Associated With Increased Stress Response Genes

The periosteum is a fibrous tissue that covers the outer surface of bones and contains a population of stem and progenitor cells that are indispensable for fracture healing. Osteoblasts at the periosteum are responsible for the enlargement of bone, which proceeds at a very slow rate after peak bone mass is attained. Several fibroblast clusters represent the majority of cells in the periosteum (Figure 7A,B, Figure S9), namely osteo‐X defined by high expression of *Postn* (periostin) and *Aspn*, fibro‐1 defined by *S100a6* and *Dcn*, fibro‐2 defined by *Mfap5* and *Clec3b*, and tenocytes characterized by expression of *Tnmd* and *Mfap4* (Figure 1C) (Nookaew et al. 2024). Less abundant cell types include osteoblasts, pre‐osteoblasts, osteo‐CAR, and adipo‐CAR. Previous studies have examined periosteal cells targeted by Prx1‐Cre or αSMA‐CreERT2 using scRNAseq and describe some of the same cluster markers, including one expressing Bglap for osteoblasts, one expressing Ly6a and CD34 for Fibro‐2, and one expressing Tnmd for tenocytes (Julien et al. 2022; Matthews et al. 2021; Xing et al. 2024).

In both females and males, the proportion of osteo‐X cells was reduced with aging (Figure 7A,B). Lower expression of *Postn* in the periosteum of old mice was confirmed by RNA in situ hybridization (Figure 7C). We also found a modest increase in the proportion of fibro‐2 cells with aging. Most cells in the fibro‐2 cluster express *Ly6a* and *Cd34* and may represent mesenchymal stem cells or other mesenchymal progenitors important for bone repair (Baryawno et al. 2019; Sidney et al. 2014).

The total number of DEGs within pre‐osteoblasts, osteo‐X, fibro‐1, and fibro‐2 clusters was similar between the female and male experiments (Figure 7D). GO analysis revealed that biological processes related to bone development, collagen production, and response to BMP were decreased in the majority of periosteal cell clusters with aging (Figure 7E). In contrast, processes related to mRNA catabolism, translation, response to TNF, JNK signaling, and nitric oxide (NO) biosynthesis were up‐regulated, suggesting increased stress responses. Indeed, in both females and males, osteo‐X exhibited up‐regulation of stress genes such as *Mt1*, *Mt2*, *Gadd45b*, *Gadd45g*, *Ier3*, *Ier5*, and many transcription factors including *Cebpd*, *Cebpb*, *Klf4*, *Fos*, *Fosb*, and *Junb* (Figure 7E,F). The receptor for parathyroid hormone (*Pth1r*), several collagens, and other ECM genes, such as *Postn*, were downregulated in osteo‐X and other periosteal clusters (Figure 7E,F, Tables S4 and S5).

## 4 Discussion

We have previously examined in detail the major mesenchymal cell clusters associated with endosteal versus periosteal bone surfaces in young mice and elucidated the location of these cells using in situ hybridization (Nookaew et al. 2024). The findings presented here provide a comprehensive description of the aging‐associated transcriptional changes that occur in these different mesenchymal cell types. Others have examined old mice when describing mesenchymal cells targeted by Col2‐Cre (Zhong et al. 2020) or LepR‐Cre (Mo et al. 2022) using scRNAseq. However, no detailed description of the effects of aging was included in these studies. A comparison of FACS‐isolated mesenchymal cells from juvenile and old male mice has also been performed (Ambrosi et al. 2021). These cells contained chondrocytes, CAR cells, and osteoblasts, but the age‐related analysis did not discriminate among the different clusters or origin of the cells, that is, endosteum versus periosteum. Thus, a direct comparison between this and our current studies is not possible. To our knowledge, our current study is the first to compare aging‐associated changes in individual bone mesenchymal cell clusters. Our findings that pre‐osteoblasts and osteoblasts exhibited the most transcriptional changes were surprising, particularly when compared to long‐lived cells such as osteocytes. Among others, we identified changes in pathways related to ECM, phosphorylation, translation, and stimulation of angiogenesis. In addition, our findings revealed unanticipated differences between male and female aging transcriptomes and between cells of the endosteum versus the periosteum.

One of the novel findings from our analysis was the increase in the proportion of pre‐osteoblasts with aging, seen in both female and male mice. This is in contrast with the current paradigm that aging of skeletal stem cells and a decrease in osteoblast progenitors contribute to the decrease in bone formation and bone loss (Butler et al. 2022; Melis et al. 2025). The studies on which this paradigm is based used Cre‐driver strains that were not specific for stem cells or used cocktails of antibodies to isolate stem cells. However, the contribution of cells identified in these previous studies to physiological adult bone remodeling remains unclear. Our studies suggest that in old mice the number of pre‐osteoblasts is not limiting, but their differentiation to osteoblasts might be compromised. A decline in Wnt signaling with age due to the up‐regulation of Lrp4, Wif, and other Wnt inhibitors may reduce the differentiation of pre‐osteoblasts and thereby reduce bone formation.

The findings that an increase in markers of senescence with aging was limited to the pre‐osteoblast and osteoblast clusters at the endosteum were unexpected. Adipo‐CAR and osteo‐CAR cells from endosteal preparations and periosteal cells, including pre‐osteoblasts, showed no changes in markers of senescence. Based on these differences, we speculate that bone mesenchymal cell senescence does not result from circulating factors, but from intrinsic cellular stresses occurring in particular cell populations. While the source of the stress that causes osteoblast senescence remains unclear, a clue might be provided by our findings that autophagy decreases with aging and suppression of autophagy increases markers of senescence in pre‐osteoblasts and osteoblasts. These cells produce large amounts of collagen I, which is prone to misfolding. Thus, a decline in autophagy with aging might contribute to the accumulation of misfolded proteins, a source of stress that contributes to senescence. In osteoblastic cells lacking *Atg7*, several SASP components were increased, although *Cdkn1a* and *Cdkn2a* were not. In cells other than bone, autophagy suppresses SASP independent of p16 and p53/p21 by promoting the degradation of the transcription factor GATA4 (Kang et al. 2015). On the other hand, autophagy can promote senescence in some cell types (Kwon et al. 2017; Slobodnyuk et al. 2019). In addition to increasing cellular senescence, and similar to aging, depletion of autophagy increased expression of genes related to translation and angiogenesis. Overall, the contrast between transcriptomic changes that occur with natural aging and autophagy deficiency provides some clues on potential hierarchies among mechanisms of aging in osteoblastic cells and warrants future studies to examine whether stimulation of autophagy in osteoblastic cells attenuates some of the effects of aging.

Our studies also revealed that the mechanisms of aging operating in endosteal versus periosteal cells are quite distinct. Specifically, we found no markers of senescence, inflammation, metabolism, hypoxia, and angiogenesis in periosteal cells from old mice. In addition, we found no obvious differences in the number of DEGs with aging in periosteal cells from female versus male mice. Perhaps more important, our findings indicate that the abundance of osteo‐X cells, characterized by high expression of *Postn*, decreases with aging in male and female mice. Osteo‐X cells, also identified as injury‐induced fibrogenic cells (Perrin et al. 2024) or Postn^+^ periosteal progenitor cells (Yin et al. 2024), undergo an early expansion following a fracture and engage in osteogenic and chondrogenic differentiation. Stem/precursor cells and osteoblasts in the periosteum also contribute to bone formation in response to mechanical loading (Matthews et al. 2021). Studies with mice lacking periostin have shown that this protein promotes fracture repair and the response to loading (Clark et al. 2023; Gerbaix et al. 2015; Wang et al. 2021). Both of these processes become less effective in aged individuals, but the mechanisms responsible remain unclear (Clark et al. 2017; Holguin et al. 2014; Resende‐Coelho et al. 2025). Osteo‐X from old mice exhibited an increase in early stress response and NO synthesis genes. A transient increase in these genes characterizes the early response of bone cells to mechanical loading (Mantila Roosa et al. 2011; Robling and Turner 2009). This early response to loading is compromised in old mice (Chermside‐Scabbo et al. 2020). We speculate that a lower number of osteo‐X and a continuous up‐regulation of stress response genes in aged individuals delays fracture repair and disrupts the response to mechanical signals.

Another novel finding from this work is the much greater change in gene expression with age in pre‐osteoblasts and osteoblasts from the endosteum of female versus male mice. Specifically, transcripts linked to the positive regulation of TNF and interleukins, transcription, and angiogenesis were altered in females but not in males. While the reasons for these differences are unknown, sex influences many of the changes seen with aging, resulting in divergent responses to infection, autoimmunity, cancer, and many other disease outcomes (Austad and Fischer 2016). Human skeletal stem cells (SSCs) collected from fracture sites of elderly females exhibit distinct phenotypes compared to SSCs from males (Ambrosi et al. 2020). A major sex difference in skeletal aging is the development of intracortical porosity, which is observed predominantly in females (Nirody et al. 2015; Piemontese et al. 2017; Tiede‐Lewis et al. 2017). Cortical porosity is caused by excessive and unbalanced bone remodeling and likely contributes to the high bone fragility of the aged female population. Intracortical pores exhibit numerous capillaries which provide oxygen and nutrients to support all cells implicated in bone remodeling (Mohanakrishnan et al. 2024; Piemontese et al. 2017). The mechanisms that contribute to cortical porosity and the recruitment of blood vessels to these sites remain unclear. We have shown that osteoblast lineage cells contribute to the development of cortical porosity (Jilka et al. 2014; Kim et al. 2020). During development and growth, the expression of *Hif1a, Hif2a, Vegfa, Slit3*, and *Pdgfrb* by cells of the osteoblast lineage contribute to bone angiogenesis (Bohm et al. 2019; Maes et al. 2010; Merceron et al. 2019; Wang et al. 2007; Xu et al. 2018). The upregulation of genes related to hypoxia and angiogenesis in osteoblastic cells from old females suggests that these cells might actively contribute to the recruitment or maintenance of capillaries in intracortical pores.

In summary, our analysis identified major transcriptomic changes caused by aging within different bone mesenchymal cell populations. Some of the age‐associated changes were expected based on previous work, but many of the altered pathways noted here have not been previously described. These findings are important because even though many aging processes are shared across organs, such as a decrease in extracellular matrix, loss of mitochondria function, and increased protein misfolding and immune response, there is little overlap of transcription factor regulatory networks (Schaum et al. 2020). Our data provide a comprehensive resource for understanding aging‐related molecular changes in osteoblasts and other mesenchymal cells in bone and may help design future genetic and pharmacological interventions aimed at attenuating osteoporosis.

## 5 Limitation of the Studies

These studies have several limitations, including the lack of sensitivity of scRNAseq to low‐expressed transcripts and the small number of mice represented in each analysis. Nonetheless, throughout the manuscript, we highlighted the instances in which the single‐cell results confirmed age‐associated changes that were observed using other experimental approaches, such as histology and flow cytometry‐based isolation of cell types. For example, the decrease in the proportion of osteoblasts was observed in both sexes and is consistent with abundant histological evidence from numerous published reports. We also found increased markers of cellular senescence, similar to other studies aimed at characterizing senescent cells in bone. This consistency increases confidence that the observed changes are an accurate reflection of reality. It is possible that sub‐populations exist within each of the major clusters described here and that aging might particularly impact these sub‐populations. The Western blot results showing phosphorylation of signaling proteins are not directly linked to the transcriptomic results, as these were not performed in situ. Lastly, we provide no evidence that any of the transcriptomic changes seen are functionally connected to the loss of bone mass with aging.

## Author Contributions

Maria Almeida, Melda Onal, Charles A. O'Brien, Jinhu Xiong, and Intawat Nookaew conceived and designed the experiments. Aaron Warren and Melda Onal performed all animal experimentation. Melda Onal, Alicen James, Adriana Marques‐Carvalho, Landon Gatrell, and Ha‐Neui Kim generated and analyzed the in vitro data. Olivia Reyes‐Castro performed RNA in situ histological analysis. Melda Onal, Charles A. O'Brien, and Jinhu Xiong collected and processed samples for scRNAseq. Intawat Nookaew, Alongkorn Kurilung, Melda Onal, Ana Resende‐Coelho, and Maria Almeida analyzed scRNAseq data. Maria Almeida, Melda Onal, Intawat Nookaew, Ana Resende‐Coelho prepared figures. Maria Almeida wrote the manuscript. All authors reviewed the manuscript.

## Conflicts of Interest

The authors declare no conflicts of interest.

## Supporting information


**Figure S1:** Endosteal mesenchymal clusters isolated from young and old male mice. Uniform manifold approximation and projection (UMAP) visualization of mesenchymal cells from endosteal bone preparations of young (6 months) or old (24 months) wild‐type male mice. Cell names and color codes are indicated at the right.

**Figure S2:** acel70256‐sup‐0002‐FigureS2.pptx. *Spp1* expression in endocortical bone surface. In situ hybridization of *Spp1* (red) was performed on femoral bone sections from old (24 months) wild‐type female mice. Images at the right are higher magnifications of the boxed areas in the left panel. BM = bone marrow, CB = cortical bone.

**Figure S3:** Transcriptional changes with age in osteocytes. (A‐C) Osteocytes were obtained from endosteal and periosteal cells isolated from young (6 months) or old (24 months) wild‐type female and male mice and combined for analysis. (A) Total number of osteocytes obtained from endosteal and periosteal isolations in young or old wild‐type females and males. (B) All differentially expressed genes significantly up‐(red) or down‐(green) regulated with age in osteocytes. (C) Gene ontology terms increasing (red) and decreasing (green) in response to aging in osteocytes. Larger circle sizes and darker colors indicate higher significance.

**Figure S4:** acel70256‐sup‐0004‐FigureS4.pptx. *Limch1* expression in the endosteum increases with age. In situ hybridization of *Limch1* (red) was performed on femoral bone sections from young (6 months) or old (24 months) wild‐type female mice. Images at the right are higher magnifications of the boxed areas in the left panels. *n* = 4–5 mice/group BM = bone marrow, CB = cortical bone.

**Figure S5:** The number of *Cdkn2a*‐*positive* cells increases with age. In situ hybridization of *Cdkn2a* was performed on femoral bone sections from young (6 months) or old (24 months) wild‐type female mice. (A) Representative images of *Cdkn2a* expression (red) on the endosteal surface of femur and quantification of the bone surface positive for *Cdkn2a* normalized to total bone surface. *n* = 4–5 mice/group; BM = bone marrow, CB = cortical bone. Bars represent mean ± SD, *p*‐values by Student's *t*‐test.

**Figure S6:** Aging does not impact hypoxia and angiogenesis in male mice. Differentially expressed genes related to blood vessel development, blood vessel remodeling, cellular response to hypoxia, and iron ion transport, significantly up‐(red) or down‐(green) regulated with age (6 vs. 24 months) in Adipo‐CAR, Osteo‐CAR, pre‐osteoblasts (Pre‐Ob), and osteoblasts (Ob) from wild‐type male mice.

**Figure S7:** Age‐related changes in genes encoding transcription factors. Differentially expressed genes related to transcription and stress responses, significantly up‐(red) or down‐(green) regulated with age (6 vs. 24 months) in Pre‐osteoblasts (Pre‐Ob) and Osteoblasts (Ob) from wild‐type female and male mice.

**Figure S8:** Age‐related changes in genes encoding ribosomal proteins. Differentially expressed genes related to ribosomal processes, significantly up‐(red) or down‐(green) regulated with age (6 months vs. 24 months) in adipo‐CAR, Osteo‐CAR, pre‐osteoblasts (Pre‐Ob), and osteoblasts (Ob) from wild‐type female and male mice.

**Figure S9:** Mesenchymal clusters isolated from Osx1‐Cre or Atg7^f/f^ mice. Uniform manifold approximation and projection (UMAP) visualization of mesenchymal cells from endosteal bone preparations of 3‐month‐old control (Osx1‐Cre) and autophagy‐deficient (Atg7^f/f^; Osx1‐Cre) male mice. Cell names and color codes are indicated at the right.

**Figure S10:** Periosteal mesenchymal clusters isolated from young and old male mice. Uniform manifold approximation and projection (UMAP) visualization of mesenchymal cells from periosteal bone preparations of young (6 months) or old (24 months) wild‐type male mice. Cell names and color codes are indicated at the right.

**Table S1:** acel70256‐sup‐0011‐TableS1.pdf.

**Table S2:** acel70256‐sup‐0012‐TableS2.pdf.

**Table S3:** acel70256‐sup‐0013‐TableS3.pdf.

**Table S4:** acel70256‐sup‐0014‐TableS4.pdf.

**Table S5:** acel70256‐sup‐0015‐TableS5.pdf.

## Data Availability

The raw sequencing data are accessible in the NCBI's Sequence Read Archive (SRA) under bioproject PRJNA1192583. Other data that support the findings of this study are available from the corresponding author upon reasonable request.
