# Supplementary material for: The Aging Landscape by scRNAseq of Mesenchymal Lineage Cells in Mouse Bone
Source: Aging Cell. 2025 Oct 13;24(12):e70256. doi: 10.1111/acel.70256 (PMC12686594; doi:10.1111/acel.70256)
Supplement: Supplementary file 1 — Figure S1: Endosteal mesenchymal clusters isolated from young and old male mice. Uniform manifold approximation and projection (UMAP) visualization of mesenchymal cells from endosteal bone preparations of young (6 months) or old (24 months) wild‐type male mice. Cell names and color codes are indicated at the right. [file ACEL-24-e70256-s011.pptx]

## Slide 1
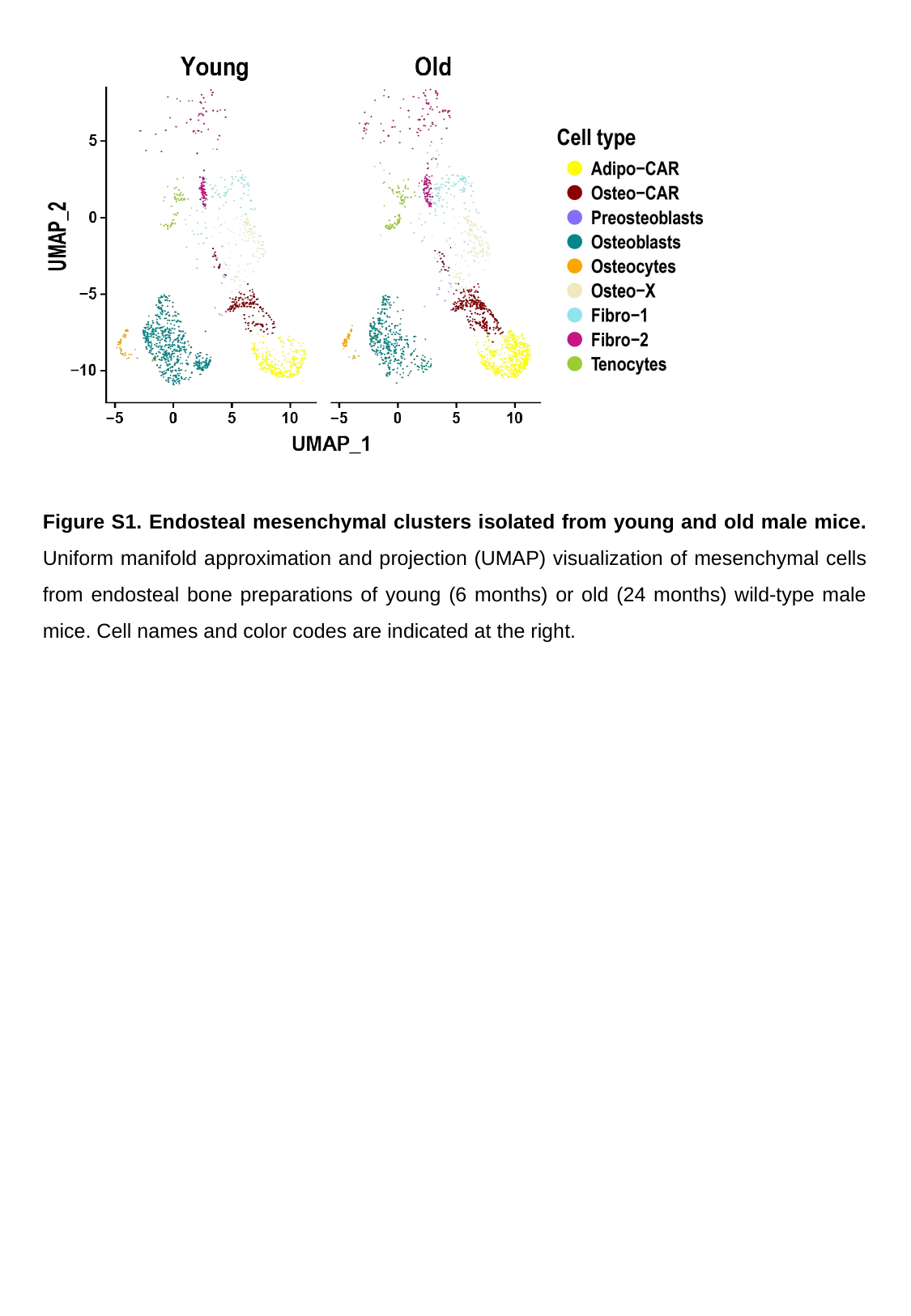

Figure S1. Endosteal mesenchymal clusters isolated from young and old male mice. Uniform manifold approximation and projection (UMAP) visualization of mesenchymal cells from endosteal bone preparations of young (6 months) or old (24 months) wild-type male mice. Cell names and color codes are indicated at the right.
