# Supplementary material for: The Aging Landscape by scRNAseq of Mesenchymal Lineage Cells in Mouse Bone
Source: Aging Cell. 2025 Oct 13;24(12):e70256. doi: 10.1111/acel.70256 (PMC12686594; doi:10.1111/acel.70256)
Supplement: Supplementary file 2 — Figure S2: acel70256‐sup‐0002‐FigureS2.pptx. Spp1 expression in endocortical bone surface. In situ hybridization of Spp1 (red) was performed on femoral bone sections from old (24 months) wild‐type female mice. Images at the right are higher magnifications of the boxed areas in the left panel. BM = bone marrow, CB = cortical bone. [file ACEL-24-e70256-s012.pptx]

## Slide 1
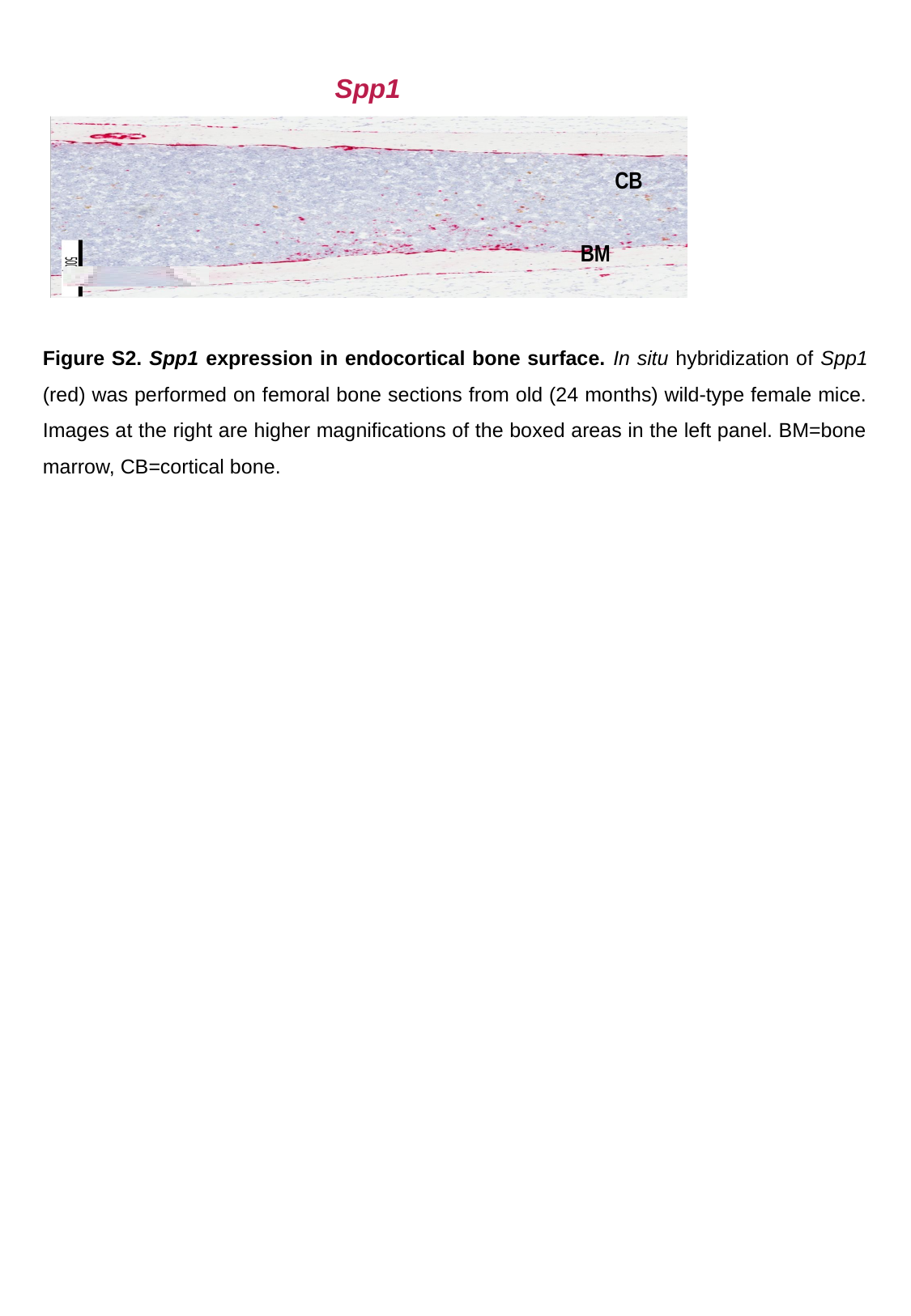

Spp1
CB
BM
Figure S2. Spp1 expression in endocortical bone surface. In situ hybridization of Spp1 (red) was performed on femoral bone sections from old (24 months) wild-type female mice. Images at the right are higher magnifications of the boxed areas in the left panel. BM=bone marrow, CB=cortical bone.
