# Supplementary material for: The Aging Landscape by scRNAseq of Mesenchymal Lineage Cells in Mouse Bone
Source: Aging Cell. 2025 Oct 13;24(12):e70256. doi: 10.1111/acel.70256 (PMC12686594; doi:10.1111/acel.70256)
Supplement: Supplementary file 3 — Figure S3: Transcriptional changes with age in osteocytes. (A‐C) Osteocytes were obtained from endosteal and periosteal cells isolated from young (6 months) or old (24 months) wild‐type female and male mice and combined for analysis. (A) Total number of osteocytes obtained from endosteal and periosteal isolations in young or old wild‐type females and males. (B) All differentially expressed genes significantly up‐(red) or down‐(green) regulated with age in osteocytes. (C) Gene ontology terms increasing (red) and decreasing (green) in response to aging in osteocytes. Larger circle sizes and darker colors indicate higher significance. [file ACEL-24-e70256-s014.pptx]

## Slide 1
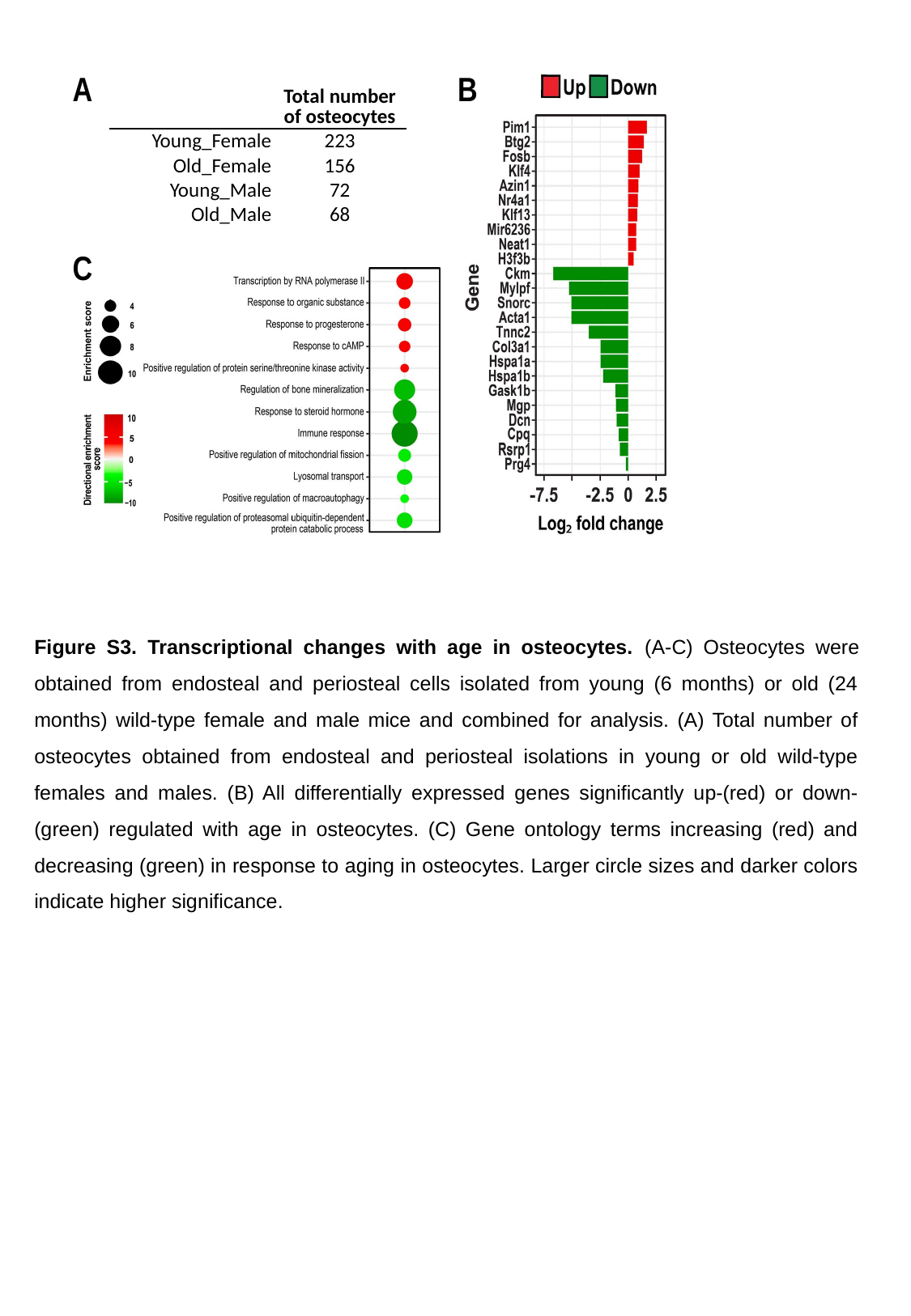

B
A
| | Total number of osteocytes |
| --- | --- |
| Young\_Female | 223 |
| Old\_Female | 156 |
| Young\_Male | 72 |
| Old\_Male | 68 |
C
Figure S3. Transcriptional changes with age in osteocytes. (A-C) Osteocytes were obtained from endosteal and periosteal cells isolated from young (6 months) or old (24 months) wild-type female and male mice and combined for analysis. (A) Total number of osteocytes obtained from endosteal and periosteal isolations in young or old wild-type females and males. (B) All differentially expressed genes significantly up-(red) or down-(green) regulated with age in osteocytes. (C) Gene ontology terms increasing (red) and decreasing (green) in response to aging in osteocytes. Larger circle sizes and darker colors indicate higher significance.
