# Supplementary material for: The Aging Landscape by scRNAseq of Mesenchymal Lineage Cells in Mouse Bone
Source: Aging Cell. 2025 Oct 13;24(12):e70256. doi: 10.1111/acel.70256 (PMC12686594; doi:10.1111/acel.70256)
Supplement: Supplementary file 5 — Figure S5: The number of Cdkn2a‐positive cells increases with age. In situ hybridization of Cdkn2a was performed on femoral bone sections from young (6 months) or old (24 months) wild‐type female mice. (A) Representative images of Cdkn2a expression (red) on the endosteal surface of femur and quantification of the bone surface positive for Cdkn2a normalized to total bone surface. n = 4–5 mice/group; BM = bone marrow, CB = cortical bone. Bars represent mean ± SD, p‐values by Student's t‐test. [file ACEL-24-e70256-s002.pptx]

## Slide 1
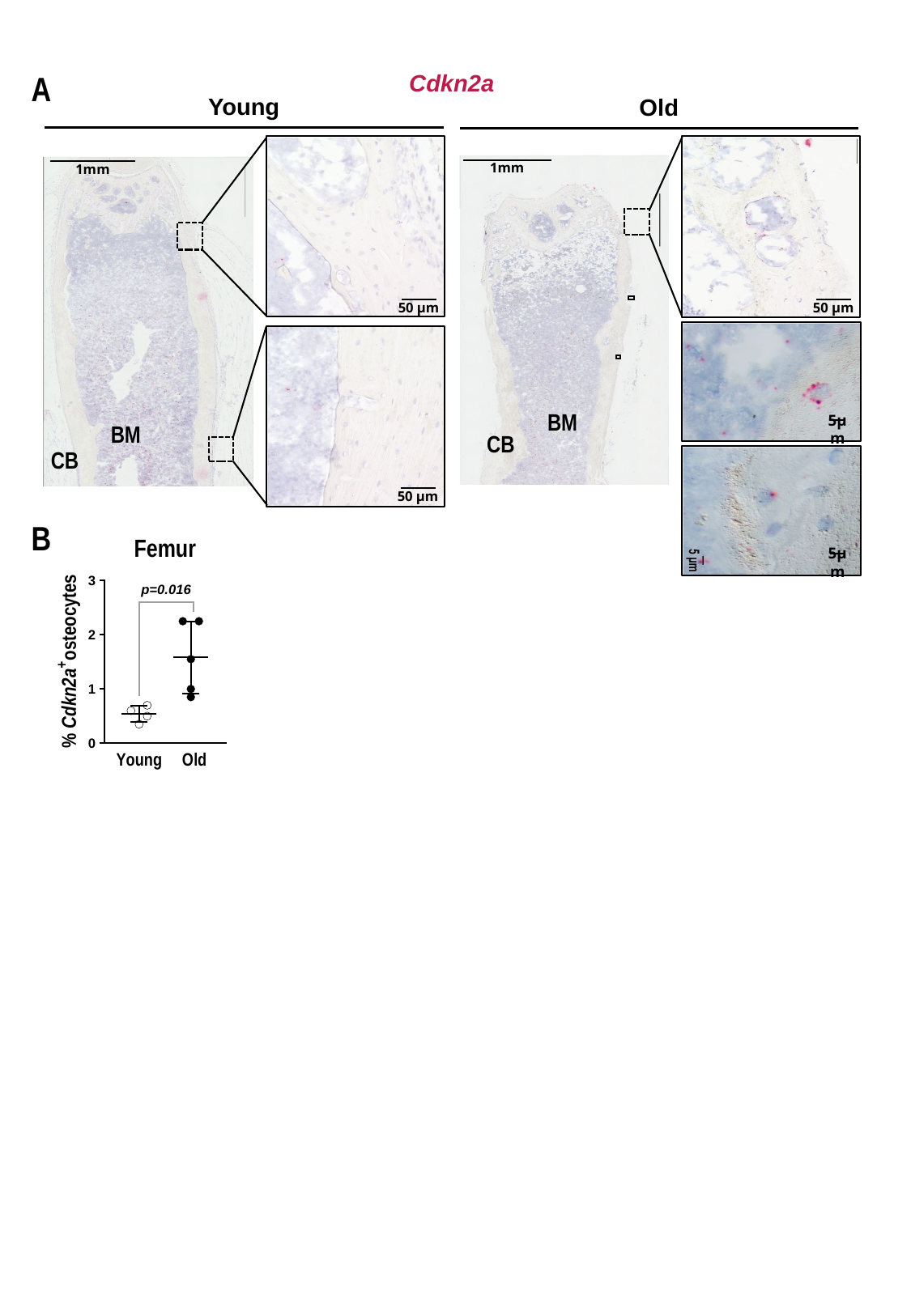

A
Cdkn2a
Young
50 µm
1mm
BM
CB
50 µm
Old
1mm
BM
CB
50 µm
5µm
5µm
B

## Slide 2
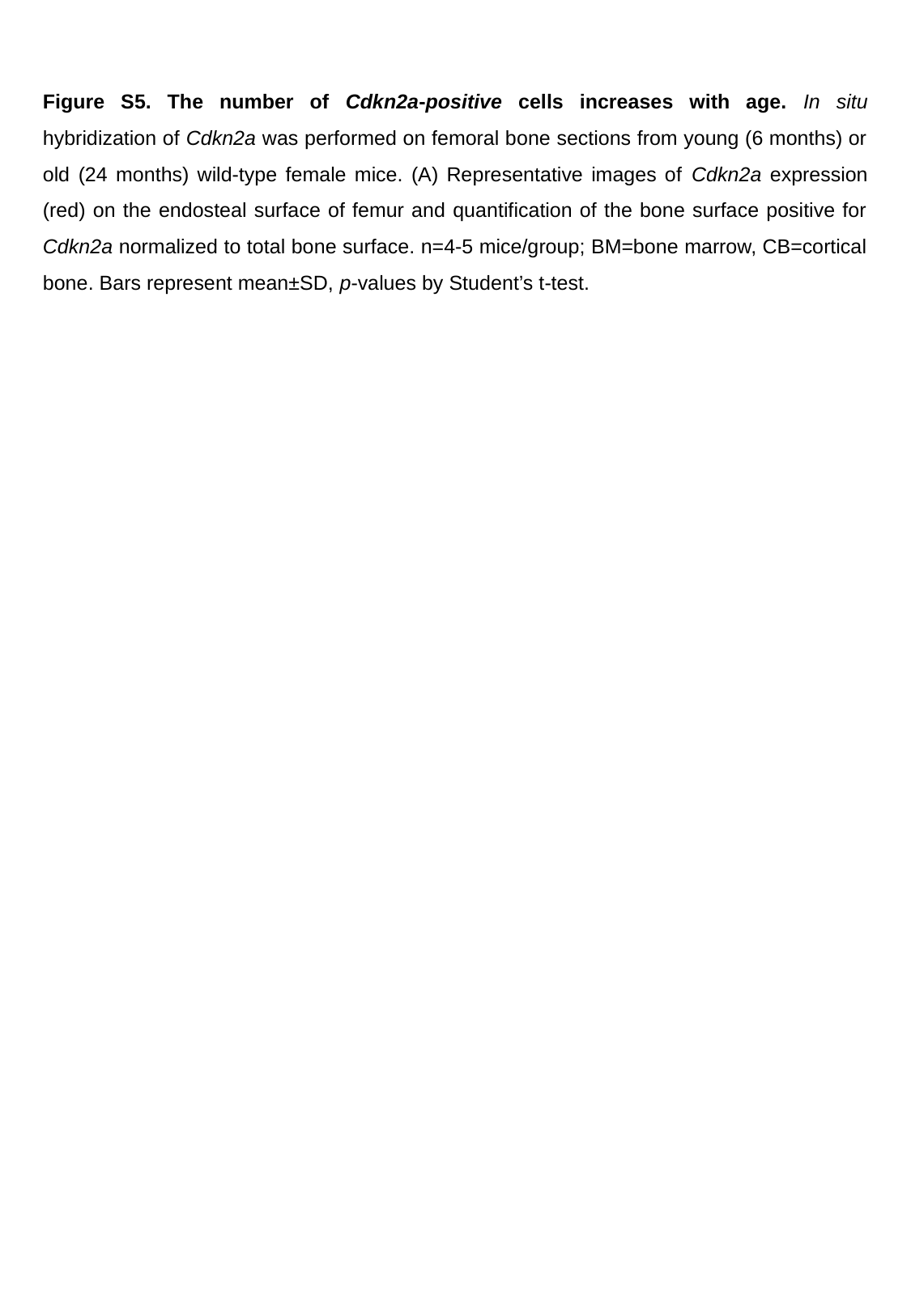

Figure S5. The number of Cdkn2a-positive cells increases with age. In situ hybridization of Cdkn2a was performed on femoral bone sections from young (6 months) or old (24 months) wild-type female mice. (A) Representative images of Cdkn2a expression (red) on the endosteal surface of femur and quantification of the bone surface positive for Cdkn2a normalized to total bone surface. n=4-5 mice/group; BM=bone marrow, CB=cortical bone. Bars represent mean±SD, p-values by Student’s t-test.
