# Supplementary material for: The Aging Landscape by scRNAseq of Mesenchymal Lineage Cells in Mouse Bone
Source: Aging Cell. 2025 Oct 13;24(12):e70256. doi: 10.1111/acel.70256 (PMC12686594; doi:10.1111/acel.70256)
Supplement: Supplementary file 6 — Figure S6: Aging does not impact hypoxia and angiogenesis in male mice. Differentially expressed genes related to blood vessel development, blood vessel remodeling, cellular response to hypoxia, and iron ion transport, significantly up‐(red) or down‐(green) regulated with age (6 vs. 24 months) in Adipo‐CAR, Osteo‐CAR, pre‐osteoblasts (Pre‐Ob), and osteoblasts (Ob) from wild‐type male mice. [file ACEL-24-e70256-s009.pptx]

## Slide 1
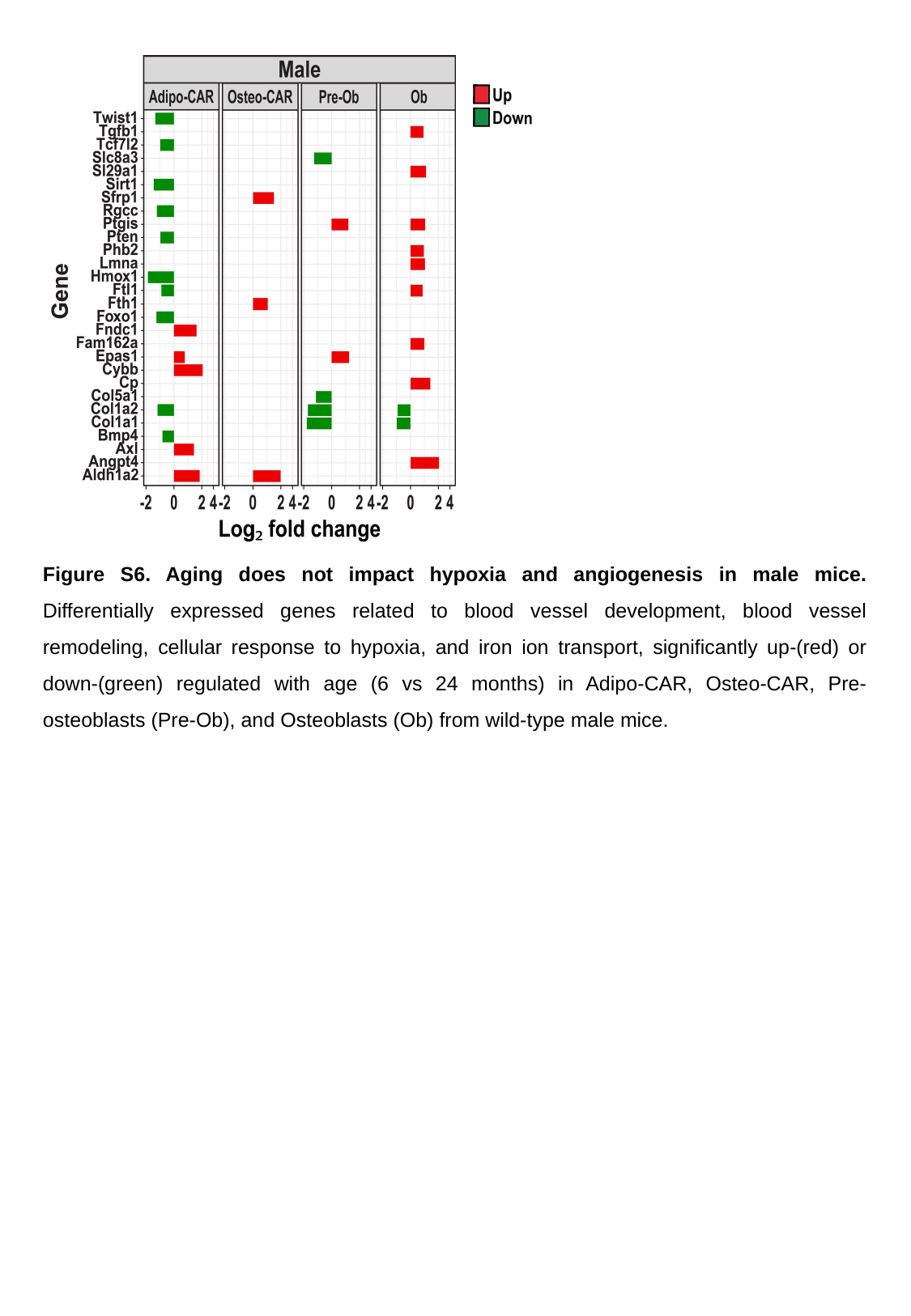

Figure S6. Aging does not impact hypoxia and angiogenesis in male mice. Differentially expressed genes related to blood vessel development, blood vessel remodeling, cellular response to hypoxia, and iron ion transport, significantly up-(red) or down-(green) regulated with age (6 vs 24 months) in Adipo-CAR, Osteo-CAR, Pre-osteoblasts (Pre-Ob), and Osteoblasts (Ob) from wild-type male mice.
