# Supplementary material for: The Aging Landscape by scRNAseq of Mesenchymal Lineage Cells in Mouse Bone
Source: Aging Cell. 2025 Oct 13;24(12):e70256. doi: 10.1111/acel.70256 (PMC12686594; doi:10.1111/acel.70256)
Supplement: Supplementary file 7 — Figure S7: Age‐related changes in genes encoding transcription factors. Differentially expressed genes related to transcription and stress responses, significantly up‐(red) or down‐(green) regulated with age (6 vs. 24 months) in Pre‐osteoblasts (Pre‐Ob) and Osteoblasts (Ob) from wild‐type female and male mice. [file ACEL-24-e70256-s001.pptx]

## Slide 1
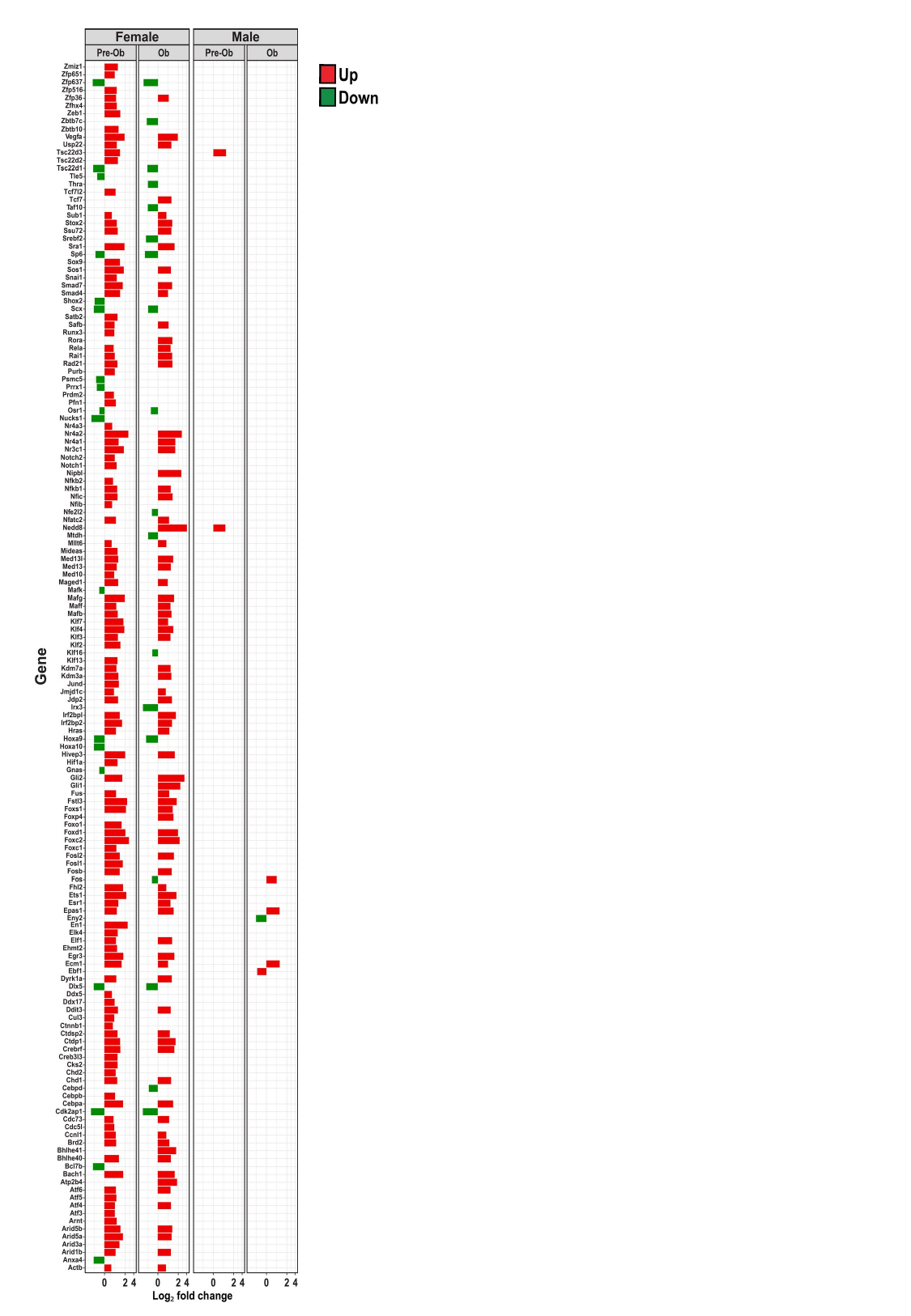

## Slide 2
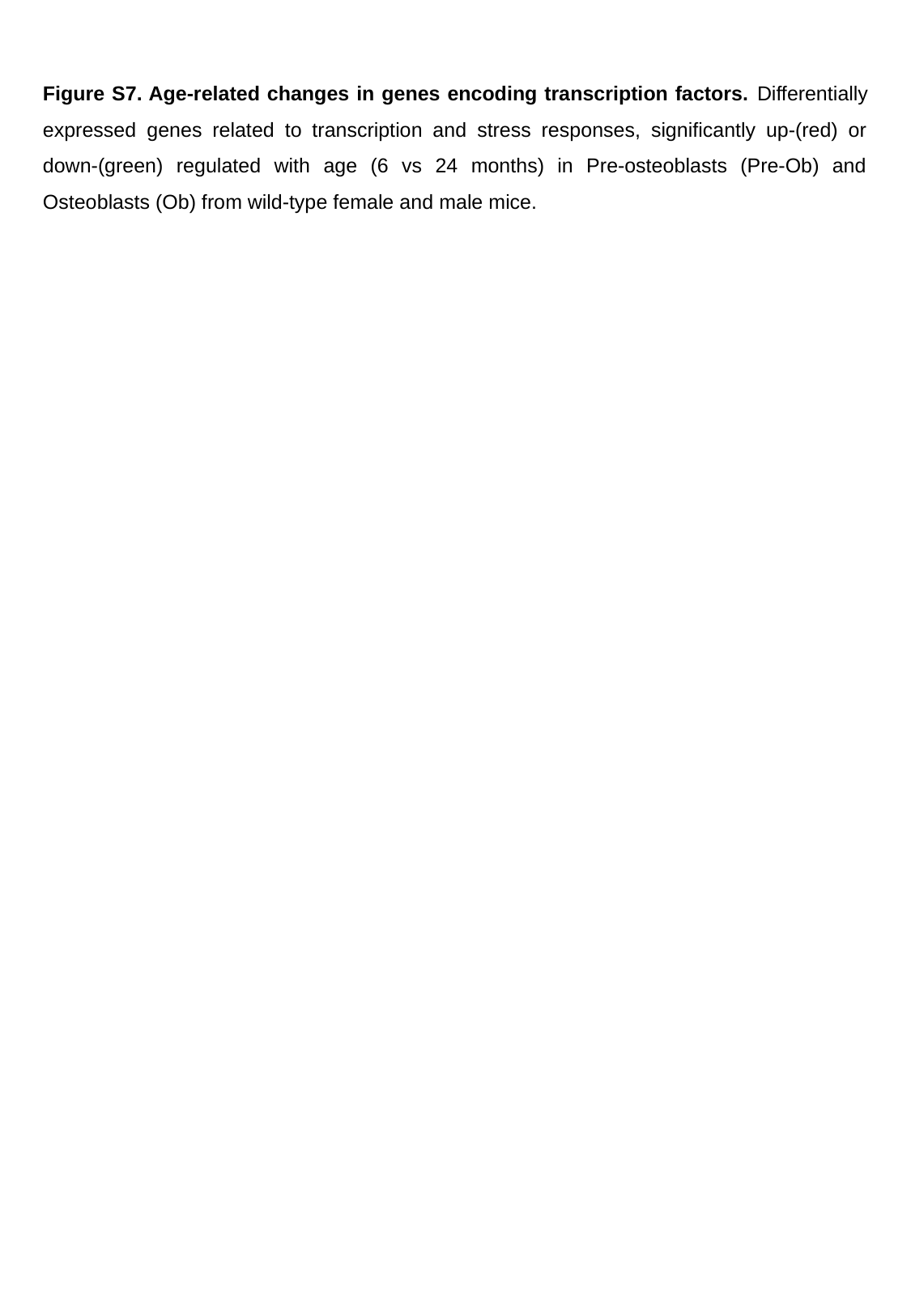

Figure S7. Age-related changes in genes encoding transcription factors. Differentially expressed genes related to transcription and stress responses, significantly up-(red) or down-(green) regulated with age (6 vs 24 months) in Pre-osteoblasts (Pre-Ob) and Osteoblasts (Ob) from wild-type female and male mice.
