# Supplementary material for: The Aging Landscape by scRNAseq of Mesenchymal Lineage Cells in Mouse Bone
Source: Aging Cell. 2025 Oct 13;24(12):e70256. doi: 10.1111/acel.70256 (PMC12686594; doi:10.1111/acel.70256)
Supplement: Supplementary file 8 — Figure S8: Age‐related changes in genes encoding ribosomal proteins. Differentially expressed genes related to ribosomal processes, significantly up‐(red) or down‐(green) regulated with age (6 months vs. 24 months) in adipo‐CAR, Osteo‐CAR, pre‐osteoblasts (Pre‐Ob), and osteoblasts (Ob) from wild‐type female and male mice. [file ACEL-24-e70256-s015.pptx]

## Slide 1
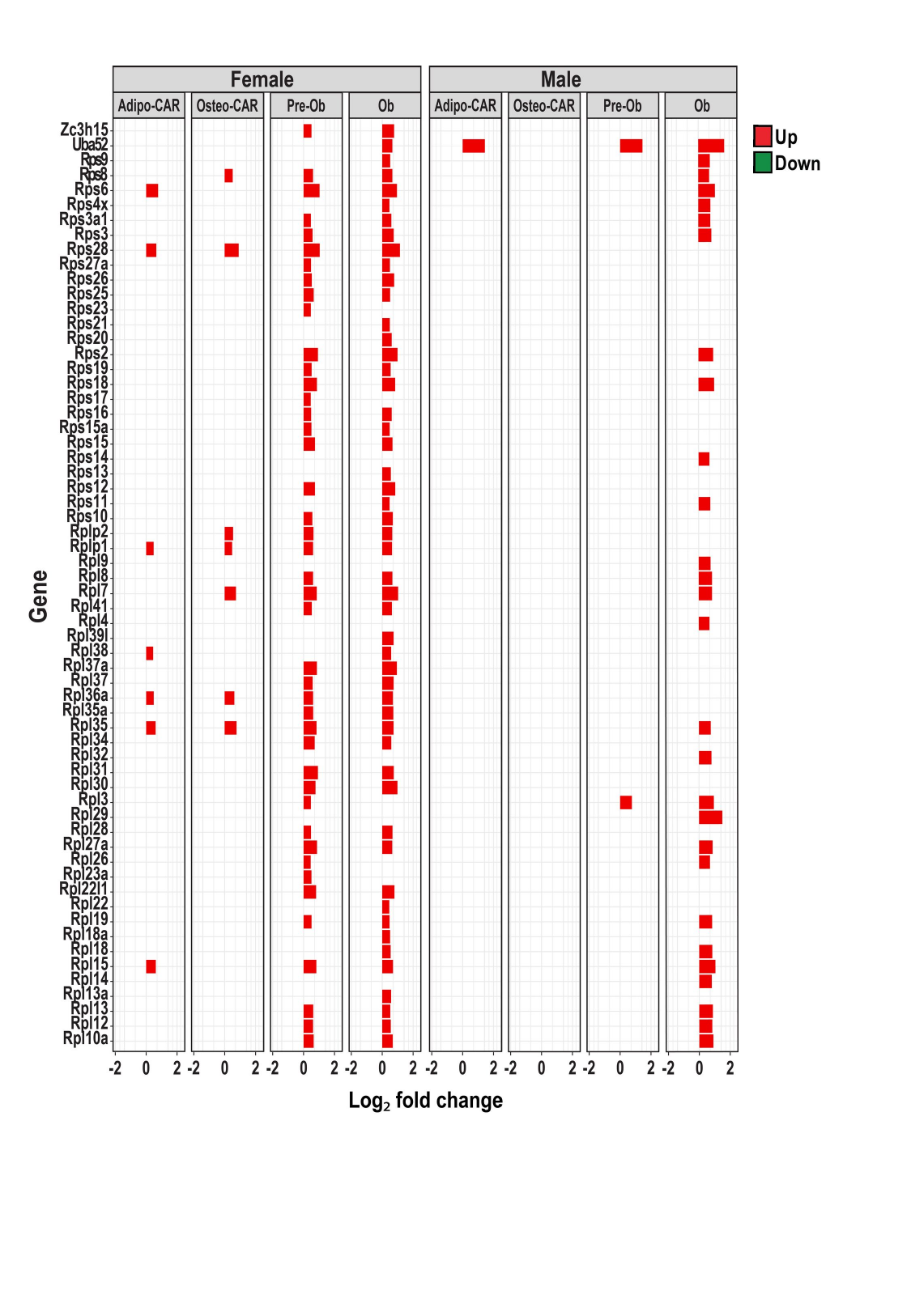

## Slide 2
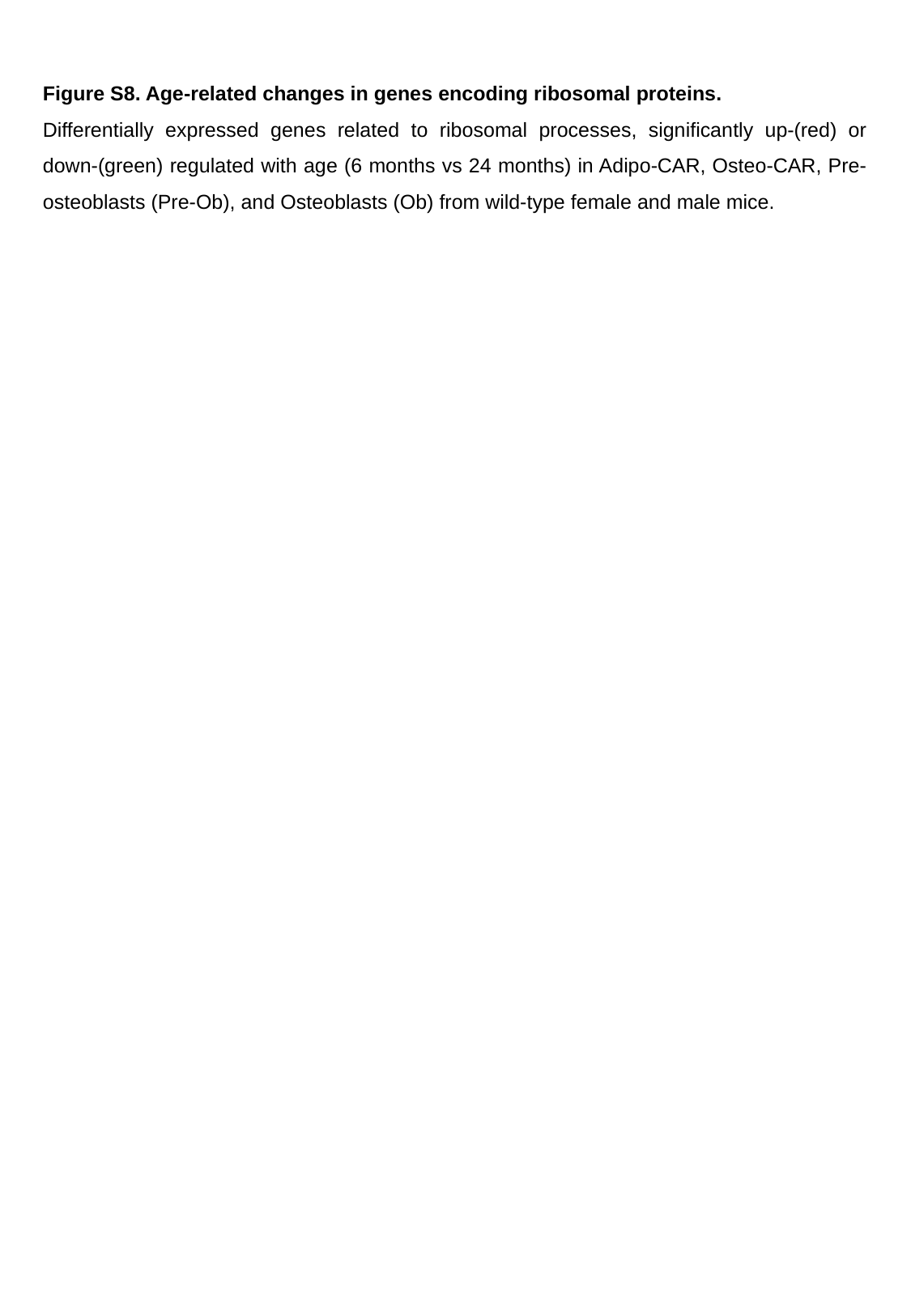

Figure S8. Age-related changes in genes encoding ribosomal proteins.
Differentially expressed genes related to ribosomal processes, significantly up-(red) or down-(green) regulated with age (6 months vs 24 months) in Adipo-CAR, Osteo-CAR, Pre-osteoblasts (Pre-Ob), and Osteoblasts (Ob) from wild-type female and male mice.
