# Supplementary material for: The Aging Landscape by scRNAseq of Mesenchymal Lineage Cells in Mouse Bone
Source: Aging Cell. 2025 Oct 13;24(12):e70256. doi: 10.1111/acel.70256 (PMC12686594; doi:10.1111/acel.70256)
Supplement: Supplementary file 9 — Figure S9: Mesenchymal clusters isolated from Osx1‐Cre or Atg7f/f mice. Uniform manifold approximation and projection (UMAP) visualization of mesenchymal cells from endosteal bone preparations of 3‐month‐old control (Osx1‐Cre) and autophagy‐deficient (Atg7f/f; Osx1‐Cre) male mice. Cell names and color codes are indicated at the right. [file ACEL-24-e70256-s010.pptx]

## Slide 1
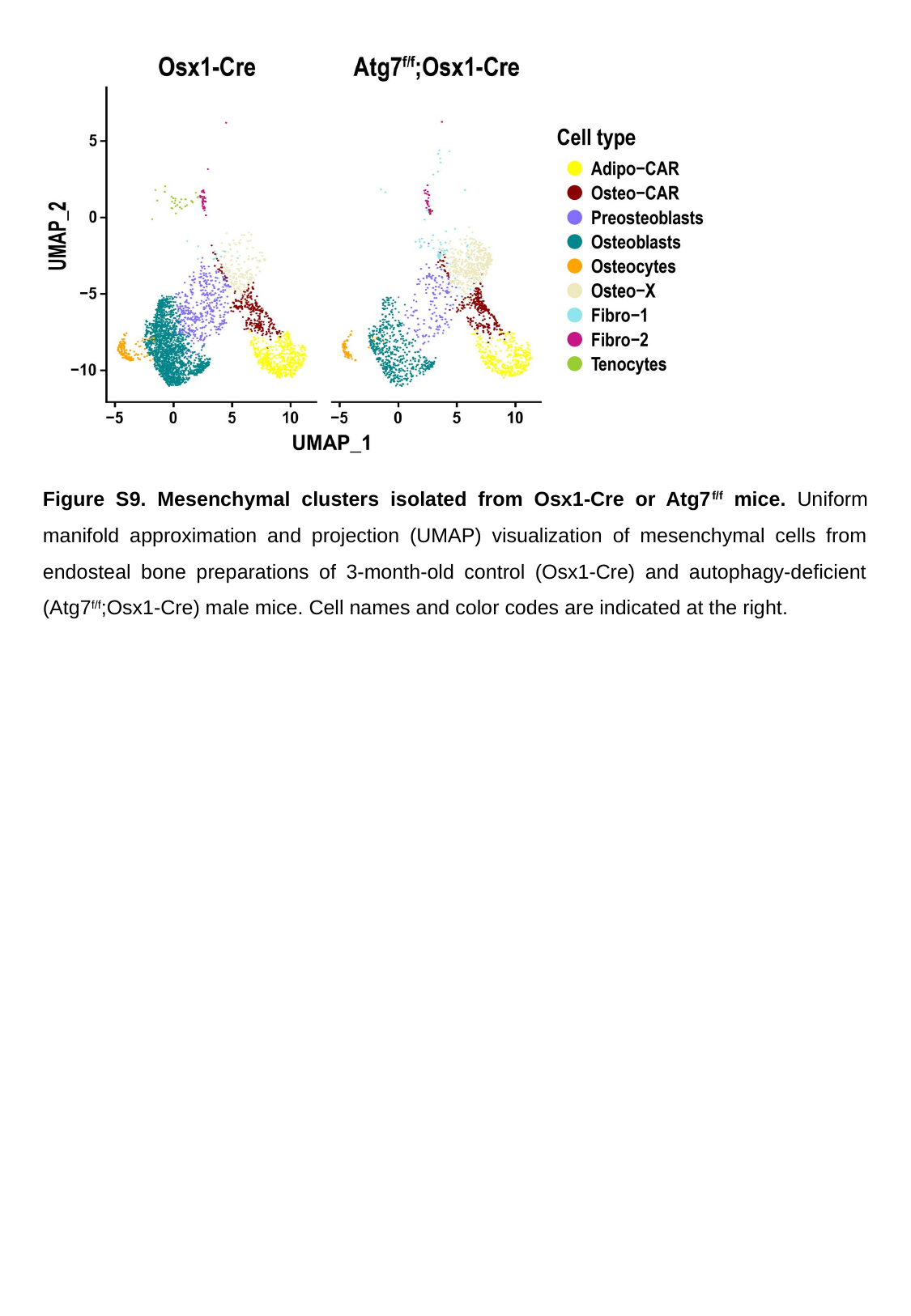

Figure S9. Mesenchymal clusters isolated from Osx1-Cre or Atg7f/f mice. Uniform manifold approximation and projection (UMAP) visualization of mesenchymal cells from endosteal bone preparations of 3-month-old control (Osx1-Cre) and autophagy-deficient (Atg7f/f;Osx1-Cre) male mice. Cell names and color codes are indicated at the right.
