# Supplementary material for: The Aging Landscape by scRNAseq of Mesenchymal Lineage Cells in Mouse Bone
Source: Aging Cell. 2025 Oct 13;24(12):e70256. doi: 10.1111/acel.70256 (PMC12686594; doi:10.1111/acel.70256)
Supplement: Supplementary file 10 — Figure S10: Periosteal mesenchymal clusters isolated from young and old male mice. Uniform manifold approximation and projection (UMAP) visualization of mesenchymal cells from periosteal bone preparations of young (6 months) or old (24 months) wild‐type male mice. Cell names and color codes are indicated at the right. [file ACEL-24-e70256-s006.pptx]

## Slide 1
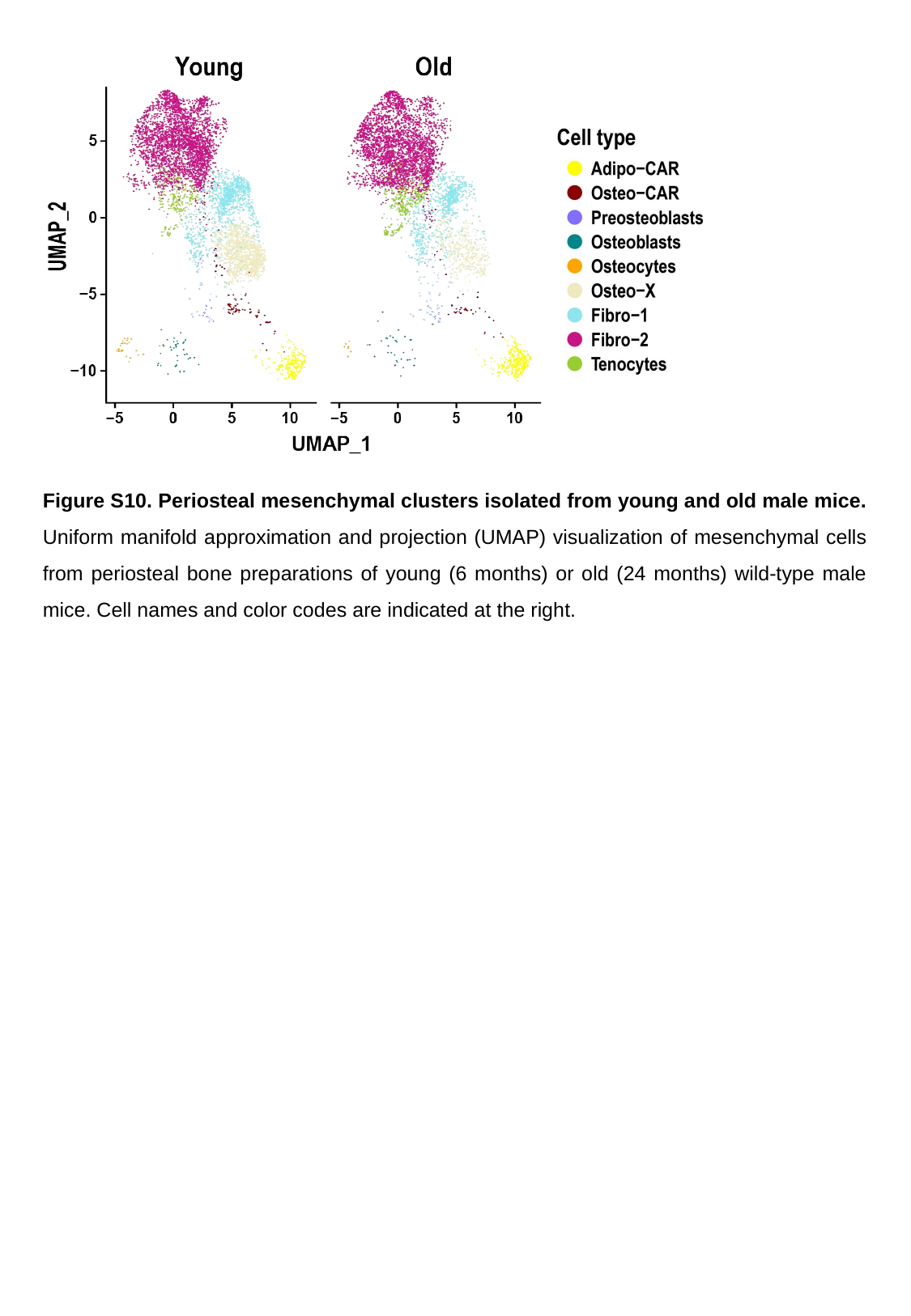

Figure S10. Periosteal mesenchymal clusters isolated from young and old male mice. Uniform manifold approximation and projection (UMAP) visualization of mesenchymal cells from periosteal bone preparations of young (6 months) or old (24 months) wild-type male mice. Cell names and color codes are indicated at the right.
