# Supplementary material for: The Aging Landscape by scRNAseq of Mesenchymal Lineage Cells in Mouse Bone
Source: Aging Cell. 2025 Oct 13;24(12):e70256. doi: 10.1111/acel.70256 (PMC12686594; doi:10.1111/acel.70256)
Supplement: Supplementary file 11 — Table S1: acel70256‐sup‐0011‐TableS1.pdf. [file ACEL-24-e70256-s005.pdf]

TABLE S1 - Experimental Summary

ID	n of mice	sex	age	bones	source	scRNA-seq	Clusters	cell number	percentage	n of total cells	median read/cell	median gene/cell	% maped on reference genome
Young wild type	2	Female	6 mo	femurs and tibias	endosteal cells	1	Adipo-CAR	608	12.84	11805	11805	2099	95.2
							Osteo-CAR	406	8.57				
							Preosteoblasts	751	15.86				
							Osteoblasts	1867	39.42				
							Osteocytes	132	2.79				
							Osteo-X	350	7.39				
							Fibro-2	195	4.12				
							Fibro-1	287	6.06				
							Tenocytes	140	2.96				
Old wild type	2	Female	24 mo	femurs and tibias	endosteal cells	1	Adipo-CAR	791	17.38	11892	20749	2064	95.7
							Osteo-CAR	444	9.75				
							Preosteoblasts	1187	26.08				
							Osteoblasts	1167	25.64				
							Osteocytes	135	2.97				
							Osteo-X	333	7.32				
							Fibro-2	245	5.38				
							Fibro-1	192	4.22				
							Tenocytes	58	1.27				

ID	n of mice	sex	age	bones	source	scRNA-seq	Clusters	cell number	percentage	n of total cells	median read/cell	median gene/cell	% maped on reference genome
Young wild type	2	Female	6 mo	femurs	periosteal cells	1	Adipo-CAR	97	0.68	27104	25537	2706	96.1
							Osteo-CAR	82	0.57				
							Preosteoblasts	632	4.41				
							Osteoblasts	438	3.06				
							Osteocytes	91	0.64				
							Osteo-X	2137	14.92				
							Fibro-2	6593	46.04				
							Fibro-1	3132	21.87				
							Tenocytes	1119	7.81				
Old wild type	2	Female	24 mo	femurs	periosteal cells	1	Adipo-CAR	24	0.22	26886	20902	2167	95.9
							Osteo-CAR	23	0.21				
							Preosteoblasts	405	3.74				
							Osteoblasts	117	1.08				
							Osteocytes	21	0.19				
							Osteo-X	894	8.25				
							Fibro-2	6138	56.63				
							Fibro-1	2372	21.89				
							Tenocytes	844	7.79				

TABLE S1 - Experimental Summary

ID	n of mice	sex	age	bones	source	scRNA-seq	Clusters	cell number	percentage	n of total cells	median read/cell	median gene/cell	% maped on reference genome
Young wild type	2 + 2	Male	6 mo	femurs and tibias	endosteal cells	2	Adipo-CAR	269	15.03	(set1) 3963<br>(set2) 4777	(set1) 57009<br>(set2) 49593	(set1) 2811<br>(set2) 2834	(set1) 90.6<br>(set2) 93.1
							Osteo-CAR	164	9.16				
							Preosteoblasts	349	19.50				
							Osteoblasts	557	31.12				
							Osteocytes	47	2.63				
							Osteo-X	127	7.09				
							Fibro-2	121	6.76				
							Fibro-1	99	5.53				
							Tenocytes	57	3.18				
Old wild type	2 + 2	Male	24 mo	femurs and tibias	endosteal cells	2	Adipo-CAR	480	19.74	(set1) 2016<br>(set2) 6479	(set1) 115526<br>(set2) 36127	(set1) 3494<br>(set2) 2871	(set1) 92.2<br>(set2) 94.1
							Osteo-CAR	290	11.93				
							Preosteoblasts	688	28.30				
							Osteoblasts	360	14.81				
							Osteocytes	60	2.47				
							Osteo-X	162	6.66				
							Fibro-2	157	6.46				
							Fibro-1	143	5.88				
							Tenocytes	91	3.74				

ID	n of mice	sex	age	bones	source	scRNA-seq	Clusters	cell number	percentage	n of total cells	median read/cell	median gene/cell	% maped on reference genome
Young wild type	2+2	Male	6 mo	femurs	periosteal cells	2	Adipo-CAR	222	3.41	(set1) 15687<br>(set2) 7295	(set1) 24928<br>(set2) 31226	(set1) 2072<br>(set2) 2604	(set1) 95.8<br>(set2) 93.3
							Osteo-CAR	83	1.27				
							Preosteoblasts	481	7.39				
							Osteoblasts	37	0.57				
							Osteocytes	25	0.38				
							Osteo-X	1166	17.91				
							Fibro-2	3057	46.94				
							Fibro-1	1199	18.41				
							Tenocytes	242	3.72				
Old wild type	2+2	Male	24 mo	femurs	periosteal cells	2	Adipo-CAR	295	5.37	(set1) 16018<br>(set2) 7295	(set1) 24246<br>(set2) 31226	(set1) 2150<br>(set2) 2604	(set1) 95.4<br>(set2) 93.3
							Osteo-CAR	39	0.71				
							Preosteoblasts	304	5.54				
							Osteoblasts	31	0.56				
							Osteocytes	8	0.15				
							Osteo-X	433	7.89				
							Fibro-2	3081	56.12				
							Fibro-1	931	16.96				
							Tenocytes	368	6.70				

TABLE S1 - Experimental Summary

ID	n of mice	sex	age	bones	source	scRNA-seq	Clusters	cell number	percentage	n of total cells	median read/cell	median gene/cell	% maped on reference genome
Osx1-Cre	1	Male	5 mo	femurs and tibias	endosteal cells	1	Adipo-CAR	503	15.23	10960	47724	2071	90.01
							Osteo-CAR	271	8.20				
							Preosteoblasts	400	12.11				
							Osteoblasts	1723	52.16				
							Osteocytes	145	4.39				
							Osteo-X	177	5.36				
							Fibro-2	37	1.12				
							Fibro-1	17	0.51				
							Tenocytes	30	0.91				
Osx1-Cre;Atg7$^{f/f}$	1	Male	5 mo	femurs and tibias	endosteal cells	1	Adipo-CAR	352	18.39	10777	48394	2449	93.3
							Osteo-CAR	286	14.94				
							Preosteoblasts	158	8.25				
							Osteoblasts	513	26.80				
							Osteocytes	66	3.45				
							Osteo-X	449	23.46				
							Fibro-2	25	1.31				
							Fibro-1	65	3.40				
