# Supplementary material for: The Aging Landscape by scRNAseq of Mesenchymal Lineage Cells in Mouse Bone
Source: Aging Cell. 2025 Oct 13;24(12):e70256. doi: 10.1111/acel.70256 (PMC12686594; doi:10.1111/acel.70256)
Supplement: Supplementary file 12 — Table S2: acel70256‐sup‐0012‐TableS2.pdf. [file ACEL-24-e70256-s008.pdf]

Osteoblasts_UP					
Gene	p_val	avg_log2FC	pct.1	pct.2	p_val_adj
Lamc3	2.81E-48	4.596555828	0.132	0.009	1.56E-43
Cd28	3.73E-38	4.43811582	0.117	0.012	2.07E-33
Vps37b	1.37E-41	4.060851106	0.129	0.014	7.57E-37
Sik1	5.55E-103	3.464742468	0.335	0.05	3.07E-98
Lgr6	7.30E-51	3.432021957	0.166	0.02	4.05E-46
Foxc2	1.87E-31	3.055629531	0.114	0.016	1.04E-26
Rab7b	9.59E-43	2.959457244	0.164	0.026	5.31E-38
Mustn1	3.76E-37	2.804352881	0.257	0.106	2.08E-32
Gadd45a	2.44E-60	2.752465148	0.332	0.119	1.35E-55
Mmp13	1.32E-36	2.745529997	0.312	0.133	7.32E-32
Nr4a3	7.61E-35	2.658570002	0.167	0.041	4.22E-30
Ackr3	1.60E-33	2.633426408	0.19	0.056	8.88E-29
En1	1.76E-20	2.571849944	0.102	0.024	9.73E-16
Cdkn2a	1.83E-66	2.480400512	0.298	0.068	1.01E-61
Foxs1	2.70E-38	2.479589211	0.18	0.039	1.50E-33
Fstl3	6.81E-40	2.476323844	0.174	0.033	3.77E-35
Pitpnc1	1.19E-30	2.388982187	0.162	0.042	6.60E-26
Ube2ql1	4.40E-40	2.356337707	0.209	0.055	2.44E-35
Lrrc15	7.89E-28	2.317951716	0.156	0.043	4.37E-23
Notum	4.54E-42	2.315697548	0.219	0.056	2.51E-37
Tgif1	1.75E-74	2.24294328	0.397	0.146	9.69E-70
Angpt4	1.01E-47	2.232022566	0.289	0.095	5.61E-43
Plaur	1.05E-50	2.179241584	0.344	0.146	5.83E-46
Scube3	2.54E-25	2.146715294	0.144	0.039	1.41E-20
Ets1	3.55E-31	2.145765851	0.162	0.039	1.97E-26
Dact1	5.31E-48	2.140826134	0.281	0.086	2.94E-43
Rgs2	2.91E-70	2.125443462	0.511	0.267	1.61E-65
Chac1	5.72E-25	2.095317771	0.18	0.07	3.17E-20
Serpine2	8.36E-92	2.080854497	0.924	0.768	4.63E-87
Gdf15	5.11E-31	2.072820088	0.248	0.101	2.83E-26
Vegfa	1.14E-80	2.0024267	0.589	0.331	6.31E-76
Spp1	1.81E-36	1.979373684	0.802	0.652	1.01E-31
Ccn2	1.23E-42	1.933995258	0.423	0.218	6.79E-38
Srebf2	2.00E-22	1.933635864	0.146	0.044	1.11E-17
Spon1	2.97E-27	1.929075219	0.174	0.052	1.65E-22
Abl2	7.33E-58	1.928062621	0.408	0.172	4.06E-53
Spsb1	2.21E-33	1.926149784	0.214	0.066	1.22E-28
Cnnm4	1.01E-26	1.916805143	0.161	0.044	5.61E-22
Foxd1	1.55E-27	1.915185875	0.188	0.062	8.60E-23
Hivep3	5.04E-26	1.90819535	0.166	0.049	2.79E-21
Slc40a1	1.64E-23	1.906694109	0.171	0.061	9.09E-19
Mafb	4.25E-38	1.897341235	0.382	0.199	2.35E-33
Ppm1l	3.03E-32	1.892983128	0.193	0.054	1.68E-27
Yod1	9.22E-37	1.882779201	0.291	0.121	5.11E-32
Lgals3	5.43E-14	1.87144346	0.113	0.041	3.01E-09

Klhl21	6.02E-47	1.866962724	0.349	0.14	3.34E-42
Srxn1	8.67E-21	1.861195205	0.172	0.069	4.80E-16
Errfi1	6.68E-85	1.853249906	0.63	0.351	3.70E-80
Gm28875	3.88E-16	1.848166637	0.108	0.033	2.15E-11
Fbxo34	5.59E-25	1.848086977	0.222	0.093	3.10E-20
Slc7a5	3.83E-24	1.842676587	0.14	0.037	2.12E-19
Klf7	4.39E-49	1.835295586	0.356	0.151	2.43E-44
Sox9	1.70E-26	1.83248249	0.243	0.109	9.45E-22
Ece1	3.69E-47	1.813480396	0.349	0.139	2.04E-42
Cebpa	2.75E-30	1.813203123	0.221	0.08	1.52E-25
Adamts12	1.43E-16	1.807929649	0.127	0.046	7.91E-12
Mafk	2.86E-63	1.785072335	0.416	0.153	1.59E-58
Anxa1	2.26E-34	1.783082283	0.387	0.227	1.25E-29
Ccdc117	3.29E-40	1.773927321	0.291	0.112	1.82E-35
Timp3	3.37E-12	1.772437065	0.133	0.063	1.87E-07
Fhl2	2.36E-24	1.729844671	0.224	0.101	1.31E-19
Bach1	1.01E-45	1.726561219	0.348	0.138	5.57E-41
Thbs2	1.11E-35	1.724068312	0.289	0.116	6.14E-31
Slc20a1	4.15E-14	1.703513842	0.107	0.036	2.30E-09
Lrrc8c	1.94E-16	1.687239426	0.123	0.041	1.07E-11
Gal	1.66E-25	1.683742134	0.327	0.178	9.20E-21
Nr4a1	1.91E-77	1.680574625	0.667	0.459	1.06E-72
Sp140	3.35E-16	1.671852649	0.127	0.045	1.86E-11
Fosl1	2.01E-20	1.664645168	0.12	0.033	1.11E-15
Pim1	8.82E-80	1.659463026	0.598	0.394	4.89E-75
Ptch1	3.60E-20	1.641197649	0.221	0.119	1.99E-15
Rarres1	1.29E-16	1.636881394	0.132	0.046	7.12E-12
Lmbr1l	1.76E-15	1.636024196	0.13	0.05	9.77E-11
Tagln2	2.07E-38	1.623669918	0.372	0.193	1.15E-33
Egr3	3.61E-55	1.622170976	0.488	0.253	2.00E-50
Dusp10	2.66E-24	1.616241153	0.212	0.084	1.47E-19
Snai1	9.71E-56	1.602393358	0.503	0.309	5.38E-51
Fam214b	3.03E-24	1.597384978	0.213	0.086	1.68E-19
Nfatc1	7.11E-30	1.590576515	0.237	0.086	3.94E-25
Serpine1	1.20E-31	1.588158182	0.298	0.143	6.63E-27
Dot1l	2.76E-41	1.586467276	0.396	0.196	1.53E-36
Hk2	1.36E-50	1.585665765	0.474	0.251	7.53E-46
Arid5a	2.62E-42	1.580416335	0.362	0.158	1.45E-37
Adam30	4.10E-19	1.579640812	0.163	0.061	2.27E-14
Ston2	2.53E-26	1.573066706	0.228	0.089	1.40E-21
Trib1	4.23E-41	1.57127991	0.32	0.12	2.34E-36
Lpin1	1.83E-16	1.568985089	0.126	0.042	1.01E-11
Pgap6	1.91E-12	1.567680953	0.113	0.044	1.06E-07
C4b	1.02E-30	1.561828651	0.248	0.091	5.63E-26
Osmr	2.00E-19	1.558820273	0.17	0.065	1.11E-14
Emp1	7.74E-54	1.542271136	0.574	0.371	4.29E-49
Frmd6	7.41E-81	1.53927365	0.651	0.415	4.11E-76

Rgcc	1.05E-64	1.537413687	0.737	0.613	5.79E-60
Tfpi	1.67E-20	1.524740751	0.18	0.07	9.26E-16
Bcl2l11	8.40E-40	1.521636488	0.383	0.206	4.66E-35
Mdm2	7.55E-41	1.521456867	0.478	0.303	4.18E-36
Gli2	2.84E-14	1.521004467	0.127	0.048	1.57E-09
Irf2bpl	2.04E-59	1.514349337	0.536	0.304	1.13E-54
Pdgfrb	2.86E-12	1.501567819	0.108	0.041	1.59E-07
Adarb1	5.41E-44	1.499922207	0.386	0.177	3.00E-39
Sgk1	4.17E-33	1.492467455	0.43	0.242	2.31E-28
Epha2	8.75E-53	1.485256277	0.467	0.224	4.85E-48
Nlrc5	2.04E-10	1.484602227	0.102	0.043	1.13E-05
Tnfrsf12a	9.65E-39	1.482603993	0.409	0.207	5.34E-34
Fxyd5	2.56E-17	1.476322554	0.163	0.066	1.42E-12
Ddi2	8.50E-45	1.472011285	0.452	0.231	4.71E-40
Carmil1	1.99E-19	1.463323793	0.195	0.09	1.11E-14
Csdc2	2.62E-16	1.462676496	0.165	0.071	1.45E-11
Sh3bp4	3.47E-24	1.461664772	0.233	0.098	1.92E-19
Sesn2	1.52E-19	1.461502028	0.195	0.092	8.44E-15
Plb1	1.22E-22	1.460862203	0.219	0.091	6.79E-18
Stk40	2.08E-29	1.454011164	0.286	0.13	1.15E-24
Micall2	1.11E-10	1.450806009	0.107	0.047	6.13E-06
Mlf1	5.05E-44	1.443229598	0.461	0.252	2.80E-39
Has1	7.39E-21	1.441928777	0.267	0.133	4.10E-16
Arl4d	8.74E-65	1.437809471	0.594	0.511	4.84E-60
Phgdh	2.96E-28	1.437578048	0.284	0.133	1.64E-23
Lmna	5.47E-90	1.43019675	0.823	0.728	3.03E-85
Cnksr3	1.20E-24	1.429583306	0.254	0.119	6.66E-20
Pfkfb4	1.85E-12	1.426118489	0.123	0.051	1.02E-07
Gsg1l	1.11E-12	1.417384876	0.106	0.038	6.12E-08
Nr1d1	4.38E-29	1.415630564	0.284	0.123	2.43E-24
Slc9a3r1	1.68E-10	1.415539723	0.118	0.053	9.30E-06
Uap1	5.68E-50	1.407356651	0.578	0.434	3.15E-45
Ccnd1	1.77E-45	1.396463781	0.491	0.259	9.79E-41
Sfrp4	4.53E-12	1.395493785	0.175	0.091	2.51E-07
Gpx3	3.22E-138	1.395364714	0.97	0.953	1.79E-133
Cdt1	4.98E-11	1.393489889	0.1	0.038	2.76E-06
Map3k6	2.36E-15	1.389242675	0.148	0.059	1.31E-10
Ctdp1	3.79E-13	1.387296954	0.125	0.049	2.10E-08
Cyp26b1	2.19E-08	1.383176165	0.107	0.055	0.001211361
Dusp5	1.94E-15	1.379241334	0.195	0.095	1.07E-10
Apold1	1.69E-11	1.376797163	0.142	0.069	9.39E-07
Ttl	1.27E-12	1.376455434	0.132	0.057	7.06E-08
Tent4a	3.65E-14	1.373890594	0.149	0.064	2.02E-09
Pip5k1a	1.98E-35	1.363417841	0.373	0.191	1.09E-30
Zfp52	6.88E-14	1.361289076	0.158	0.072	3.81E-09
Stard13	6.30E-12	1.36078578	0.123	0.051	3.49E-07
Chst15	3.59E-23	1.358500764	0.223	0.091	1.99E-18

4930523C07Rik	2.30E-30	1.356263412	0.345	0.187	1.27E-25
Ehd4	3.02E-26	1.354868561	0.297	0.145	1.67E-21
Ecm1	2.43E-32	1.343769717	0.363	0.174	1.35E-27
Itga5	5.05E-12	1.329655109	0.136	0.063	2.80E-07
Itpkb	3.79E-18	1.327241121	0.207	0.095	2.10E-13
Fcer1g	9.53E-47	1.326459002	0.453	0.209	5.28E-42
Susd6	4.73E-21	1.319521232	0.248	0.128	2.62E-16
Ssh1	5.93E-20	1.316840445	0.22	0.103	3.28E-15
Gm20186	6.41E-12	1.314395875	0.146	0.07	3.55E-07
Tubb6	1.06E-30	1.311664252	0.349	0.172	5.87E-26
Niban2	2.50E-28	1.311518967	0.331	0.18	1.39E-23
Neat1	6.79E-67	1.311200959	0.904	0.813	3.76E-62
Klf3	2.83E-44	1.308487335	0.488	0.299	1.57E-39
Foxo1	6.80E-39	1.305830745	0.412	0.204	3.77E-34
Myl1	6.27E-11	1.299329225	0.125	0.056	3.48E-06
Wif1	1.12E-59	1.295056253	0.776	0.604	6.19E-55
Arrdc3	6.71E-11	1.294552412	0.133	0.068	3.72E-06
Tnc	6.40E-42	1.293827711	0.605	0.384	3.54E-37
Pdk4	1.73E-26	1.287916178	0.391	0.251	9.61E-22
Skil	8.19E-59	1.286769218	0.65	0.423	4.54E-54
Slc35e4	6.30E-42	1.282896988	0.497	0.328	3.49E-37
Camk2a	2.17E-10	1.278664347	0.119	0.054	1.20E-05
Tmie	1.63E-10	1.278208677	0.113	0.047	9.01E-06
Clstn1	4.34E-14	1.27804914	0.156	0.069	2.40E-09
Klhl33	4.90E-13	1.275784551	0.145	0.063	2.72E-08
Morrbid	1.29E-12	1.274318392	0.155	0.073	7.12E-08
Myo1e	1.29E-37	1.272823566	0.426	0.222	7.17E-33
Crebrf	4.43E-18	1.271038142	0.224	0.11	2.46E-13
Ank	4.27E-27	1.268987972	0.343	0.181	2.37E-22
Gm19705	2.96E-15	1.26675733	0.192	0.095	1.64E-10
Mylk	8.45E-37	1.266227518	0.415	0.211	4.68E-32
Tm4sf1	2.91E-52	1.26143546	0.787	0.724	1.61E-47
Gm16133	6.03E-10	1.259371585	0.125	0.06	3.34E-05
Slc25a33	2.74E-11	1.258279937	0.124	0.053	1.52E-06
Runx1	3.34E-40	1.256748656	0.512	0.302	1.85E-35
Larp6	1.91E-15	1.256237917	0.194	0.094	1.06E-10
Midn	8.03E-54	1.256206361	0.588	0.374	4.45E-49
Plec	5.99E-41	1.254334087	0.478	0.28	3.32E-36
Slc7a1	3.19E-21	1.25224527	0.243	0.111	1.77E-16
Noct	3.29E-10	1.250394021	0.103	0.042	1.82E-05
Arid5b	6.95E-41	1.248215532	0.547	0.343	3.85E-36
Atg14	4.35E-10	1.247194086	0.118	0.053	2.41E-05
Rhod	3.42E-22	1.245157816	0.29	0.156	1.89E-17
Cramp1l	3.38E-12	1.241299235	0.154	0.074	1.87E-07
Tamalin	6.52E-18	1.238120079	0.237	0.126	3.61E-13
Arhgap23	1.73E-45	1.237932782	0.5	0.281	9.61E-41
Denn2b	3.04E-12	1.235867322	0.11	0.042	1.69E-07

Coq10b	4.60E-35	1.232884591	0.431	0.274	2.55E-30
Sp6	8.62E-12	1.223993245	0.153	0.079	4.78E-07
Ephb3	6.32E-11	1.223916268	0.132	0.061	3.50E-06
Adamts5	6.27E-34	1.209523947	0.634	0.492	3.47E-29
Dennd5b	1.13E-23	1.207727663	0.304	0.156	6.27E-19
Aebp1	9.60E-11	1.207000594	0.117	0.05	5.32E-06
Rara	2.16E-19	1.203396854	0.228	0.104	1.19E-14
Fbxo32	1.55E-23	1.20054168	0.394	0.262	8.61E-19
Zfp618	2.89E-12	1.200128324	0.158	0.076	1.60E-07
Aqp1	2.28E-51	1.194346798	0.583	0.348	1.26E-46
Mir6236	6.85E-61	1.191465147	0.998	0.962	3.80E-56
Irx3	5.02E-25	1.191183974	0.384	0.236	2.78E-20
Sema4c	4.04E-20	1.190881012	0.264	0.132	2.24E-15
Smad7	3.24E-54	1.186691221	0.662	0.476	1.79E-49
Colec12	8.47E-60	1.182120276	0.728	0.545	4.70E-55
Acsl3	2.43E-12	1.17983029	0.18	0.094	1.35E-07
Zeb1	1.91E-13	1.177901409	0.168	0.078	1.06E-08
Rabgef1	1.66E-13	1.175131167	0.185	0.094	9.18E-09
Fosl2	3.05E-37	1.174208514	0.51	0.317	1.69E-32
Sema7a	1.62E-23	1.173573951	0.315	0.165	8.97E-19
Synm	5.91E-14	1.173232624	0.19	0.094	3.27E-09
Irs2	2.07E-29	1.171128887	0.387	0.209	1.15E-24
Ugdh	8.64E-42	1.17048984	0.627	0.435	4.79E-37
Kdm6b	3.02E-49	1.170063019	0.623	0.417	1.67E-44
B3galt2	3.08E-15	1.169483131	0.236	0.134	1.71E-10
Tsc22d3	4.98E-39	1.168832978	0.547	0.38	2.76E-34
Myom1	3.99E-14	1.162891017	0.195	0.098	2.21E-09
Map1b	6.50E-10	1.158914762	0.151	0.084	3.60E-05
Kremen1	3.12E-09	1.156718224	0.123	0.059	0.00017259
Uaca	3.33E-42	1.152638811	0.533	0.345	1.85E-37
Cd44	7.71E-32	1.150621532	0.435	0.27	4.27E-27
Hmgcr	3.69E-24	1.146705053	0.351	0.207	2.04E-19
Cyp51	1.57E-12	1.13979553	0.151	0.069	8.70E-08
Bmp8a	3.99E-19	1.135598954	0.281	0.155	2.21E-14
Slc7a2	2.08E-12	1.131861645	0.172	0.085	1.15E-07
Retreg1	4.70E-12	1.131566914	0.18	0.095	2.60E-07
Furin	1.24E-38	1.126378797	0.488	0.311	6.87E-34
Spats2	5.47E-15	1.125449457	0.217	0.113	3.03E-10
Zcchc14	2.19E-38	1.122155288	0.484	0.274	1.21E-33
Arhgef2	7.61E-10	1.12160127	0.136	0.066	4.21E-05
Plxna1	3.33E-11	1.119858829	0.162	0.084	1.85E-06
Apol6	1.24E-07	1.116400752	0.107	0.052	0.00687355
Ngef	4.14E-08	1.115547215	0.123	0.065	0.002295452
Tuba1c	1.59E-44	1.10956494	0.61	0.418	8.80E-40
Ifrd1	1.32E-42	1.109535935	0.748	0.662	7.34E-38
Btg2	3.30E-51	1.10836931	0.83	0.761	1.83E-46
Zfp451	4.67E-13	1.10790198	0.194	0.101	2.59E-08

Epb41	4.78E-11	1.107621088	0.142	0.066	2.65E-06
Aopep	1.79E-16	1.106859886	0.204	0.094	9.89E-12
C1ra	7.71E-07	1.105667245	0.116	0.065	0.04272271
Heg1	2.38E-32	1.104262589	0.45	0.258	1.32E-27
Hmox1	1.28E-20	1.100217391	0.474	0.32	7.11E-16
Atl2	3.02E-16	1.099980174	0.236	0.124	1.67E-11
Kdm3a	6.23E-14	1.099865935	0.178	0.083	3.45E-09
Birc3	1.78E-07	1.096799627	0.108	0.054	0.00983681
Rc3h1	9.31E-38	1.094138811	0.508	0.315	5.16E-33
Eaf1	1.50E-19	1.092720842	0.289	0.157	8.30E-15
Tns3	3.39E-17	1.0899821	0.269	0.155	1.88E-12
Bmt2	2.25E-07	1.088434713	0.113	0.061	0.012485896
Pi4k2a	5.12E-11	1.082284288	0.166	0.091	2.84E-06
Mdfic	2.30E-07	1.079484925	0.112	0.058	0.012730966
Arid3a	8.87E-09	1.077889871	0.145	0.078	0.000491695
Lbr	2.51E-18	1.076572063	0.276	0.151	1.39E-13
Tnfrsf1b	4.75E-07	1.074231607	0.11	0.057	0.026330945
Arhgef10l	9.27E-11	1.072806501	0.176	0.095	5.14E-06
Mtmr10	4.09E-09	1.068003182	0.133	0.066	0.000226824
Cpeb2	1.25E-08	1.067150974	0.111	0.052	0.000690703
Mpp5	3.85E-15	1.065464206	0.238	0.132	2.13E-10
Slc38a2	8.80E-78	1.063442326	0.874	0.773	4.87E-73
Klhl30	4.72E-10	1.063109014	0.109	0.047	2.62E-05
Rictor	1.02E-13	1.058320317	0.199	0.1	5.66E-09
Ier3	1.49E-45	1.056314273	0.861	0.835	8.27E-41
Sash1	8.95E-38	1.055264649	0.521	0.35	4.96E-33
Fosb	5.94E-81	1.054125509	0.91	0.799	3.29E-76
Nupr1	9.78E-109	1.052979398	0.998	0.999	5.42E-104
Lpin2	7.04E-17	1.050277022	0.281	0.164	3.90E-12
D830025C05Rik	4.34E-21	1.048967513	0.358	0.223	2.41E-16
Cks2	3.91E-12	1.04612791	0.242	0.153	2.16E-07
Asns	2.39E-13	1.045029855	0.237	0.139	1.32E-08
Sqstm1	2.36E-60	1.043693972	0.791	0.684	1.31E-55
Fam189a2	2.25E-08	1.040900807	0.147	0.082	0.001249203
Dmp1	6.24E-16	1.040051374	0.483	0.377	3.46E-11
Bhlhe40	1.61E-46	1.03851409	0.643	0.483	8.94E-42
Nup50	2.04E-22	1.038052064	0.349	0.215	1.13E-17
Pacsin2	1.72E-10	1.0369047	0.138	0.065	9.55E-06
Kpna1	2.20E-11	1.032282407	0.176	0.091	1.22E-06
Ralgds	2.69E-12	1.031598419	0.199	0.106	1.49E-07
Cplane1	1.09E-08	1.031201899	0.142	0.075	0.000601202
Col12a1	2.64E-13	1.029634862	0.255	0.149	1.46E-08
Zfp36l2	4.55E-21	1.029321662	0.378	0.241	2.52E-16
Ddit4	9.85E-23	1.029059861	0.447	0.313	5.46E-18
Csrp1	3.41E-23	1.024839409	0.415	0.303	1.89E-18
Chd7	5.55E-19	1.024745436	0.314	0.183	3.07E-14
Map3k3	2.94E-07	1.024286714	0.121	0.065	0.016298362

Ivns1abp	1.64E-28	1.02412144	0.452	0.276	9.10E-24
Rrm1	3.01E-09	1.024011997	0.144	0.074	0.000166658
Inafm2	1.01E-12	1.020658547	0.22	0.127	5.58E-08
Fbxo30	1.83E-17	1.020306787	0.303	0.176	1.02E-12
Ndst1	3.02E-11	1.019509354	0.185	0.098	1.68E-06
Hapln4	3.15E-10	1.017945038	0.181	0.103	1.75E-05
Lclat1	2.02E-13	1.016199206	0.225	0.125	1.12E-08
Ptpn1	2.74E-30	1.016193591	0.518	0.384	1.52E-25
Hdac4	3.37E-09	1.01539591	0.159	0.088	0.000186463
Hipk1	8.14E-33	1.013257481	0.511	0.318	4.51E-28
Hsd17b7	4.68E-11	1.007779057	0.132	0.061	2.59E-06
Frmd4a	6.60E-12	1.005666788	0.194	0.102	3.66E-07
Sbno2	5.23E-17	1.004516327	0.275	0.149	2.90E-12
Itpkc	1.87E-08	1.004418726	0.117	0.056	0.001036869
Zbtb10	4.75E-11	1.004398634	0.19	0.111	2.63E-06
Man2a2	2.13E-09	1.003865638	0.127	0.061	0.000117763
Taf4b	1.93E-10	1.001213791	0.164	0.084	1.07E-05
Mindy1	4.73E-13	0.997084117	0.228	0.127	2.62E-08
Myh9	2.34E-40	0.995332659	0.598	0.384	1.30E-35
Zfp703	2.43E-18	0.994990696	0.34	0.215	1.35E-13
Cp	5.15E-35	0.992429021	0.599	0.414	2.85E-30
Zc3h12a	1.53E-08	0.992041416	0.132	0.068	0.000846301
Med10	4.77E-41	0.990104247	0.596	0.509	2.64E-36
Tgm2	3.94E-10	0.985594771	0.183	0.102	2.19E-05
Nr4a2	1.70E-31	0.985538757	0.581	0.476	9.44E-27
Fam71a	7.67E-10	0.984959654	0.211	0.132	4.25E-05
Gm38126	3.31E-09	0.982854234	0.122	0.058	0.000183409
Kdm7a	1.41E-28	0.98185241	0.488	0.311	7.79E-24
Zfp655	2.93E-15	0.977817966	0.259	0.142	1.62E-10
Jmjd1c	2.78E-31	0.976863963	0.55	0.386	1.54E-26
Map2k3	3.22E-31	0.974320968	0.526	0.37	1.79E-26
Fam214a	2.67E-14	0.97400642	0.238	0.129	1.48E-09
Plaat3	3.96E-09	0.973213376	0.158	0.085	0.00021921
Adamts4	1.04E-07	0.969010883	0.103	0.049	0.0057659
Ankrd28	4.82E-17	0.968500868	0.292	0.165	2.67E-12
Fam20c	8.91E-27	0.965904404	0.493	0.353	4.94E-22
Slc16a1	1.13E-08	0.965899526	0.156	0.087	0.000626824
Rnf24	1.23E-12	0.96586899	0.228	0.129	6.84E-08
Tbc1d4	4.87E-08	0.964063081	0.148	0.084	0.002697329
Hbegf	4.24E-12	0.959196943	0.334	0.233	2.35E-07
1700017B05Rik	2.46E-08	0.958428175	0.139	0.073	0.001365101
Psmb8	4.41E-22	0.957239563	0.425	0.278	2.44E-17
Smg1	2.69E-17	0.955240617	0.312	0.183	1.49E-12
Gja1	3.29E-11	0.953681753	0.225	0.134	1.82E-06
Mdfi	2.42E-24	0.952179935	0.408	0.264	1.34E-19
Tcp11l2	3.00E-29	0.94962484	0.542	0.382	1.66E-24
Bok	4.98E-16	0.948800241	0.311	0.203	2.76E-11

Rab3gap2	1.55E-07	0.94709195	0.135	0.074	0.008575369
Ell	1.97E-13	0.944175538	0.234	0.129	1.09E-08
Kdsr	6.22E-08	0.943285204	0.154	0.093	0.003445249
Zmiz1	5.33E-12	0.941090686	0.245	0.152	2.95E-07
Aldh1l2	6.39E-21	0.940353127	0.386	0.244	3.54E-16
Slc41a1	1.65E-14	0.935936145	0.236	0.125	9.13E-10
Cspg4	1.56E-12	0.934325782	0.267	0.166	8.65E-08
Nckap5l	5.15E-17	0.93411686	0.33	0.201	2.85E-12
Phf3	4.40E-19	0.934012534	0.362	0.22	2.44E-14
Zfand5	1.62E-62	0.933700079	0.893	0.853	8.95E-58
Olfml2b	6.73E-07	0.932735115	0.137	0.078	0.037289191
Ahctf1	1.07E-13	0.932665675	0.252	0.142	5.94E-09
Cpd	2.18E-13	0.932119054	0.263	0.161	1.21E-08
Azin1	1.40E-79	0.930667847	0.874	0.728	7.75E-75
Mef2d	3.31E-24	0.930360733	0.435	0.266	1.83E-19
Emilin1	4.29E-46	0.929611156	0.687	0.479	2.38E-41
Mknk2	7.35E-21	0.9280406	0.405	0.268	4.07E-16
B9d2	1.29E-20	0.922651221	0.389	0.256	7.16E-16
Prkce	4.45E-07	0.92073924	0.139	0.08	0.024650918
Slc41a2	1.35E-11	0.918047316	0.236	0.145	7.47E-07
Ier5	1.02E-36	0.917055427	0.691	0.612	5.63E-32
Rb1cc1	4.86E-33	0.91694055	0.604	0.44	2.69E-28
Stat3	1.10E-34	0.916239322	0.584	0.388	6.11E-30
Ddit3	3.03E-18	0.914821421	0.424	0.295	1.68E-13
Bmp4	2.95E-10	0.913101135	0.177	0.095	1.64E-05
Ctdsp2	6.98E-27	0.912496328	0.476	0.305	3.86E-22
Spata13	3.25E-24	0.911506022	0.432	0.262	1.80E-19
Scpep1	4.72E-15	0.910778608	0.303	0.186	2.62E-10
Pim3	2.90E-22	0.910031349	0.474	0.331	1.61E-17
Pla2g4a	8.53E-17	0.90666603	0.334	0.201	4.72E-12
Odc1	4.69E-27	0.904842864	0.53	0.364	2.60E-22
Ppp1r15b	1.49E-18	0.90471436	0.353	0.215	8.24E-14
Tsc22d2	2.26E-23	0.903067535	0.465	0.301	1.25E-18
Cbfa2t3	2.00E-14	0.901366173	0.305	0.19	1.11E-09
H2-Q4	9.23E-17	0.90035902	0.352	0.232	5.11E-12
Trio	3.08E-17	0.899454872	0.358	0.237	1.71E-12
Prkx	6.34E-08	0.898451843	0.147	0.081	0.003513658
Hjurp	5.25E-12	0.896086697	0.243	0.141	2.91E-07
Rrp1b	8.07E-07	0.893635957	0.113	0.081	0.044701129
Ppard	2.58E-07	0.893508163	0.137	0.075	0.014321261
Tpp2	2.50E-16	0.891597292	0.339	0.213	1.38E-11
Klf16	4.43E-11	0.891230981	0.196	0.107	2.45E-06
Adamts9	5.09E-17	0.888939839	0.373	0.242	2.82E-12
Ptk7	3.71E-08	0.887937042	0.165	0.094	0.00205781
Lsmem1	8.49E-14	0.886742556	0.233	0.126	4.71E-09
Septin9	1.89E-28	0.885376281	0.531	0.353	1.05E-23
Mideas	1.99E-09	0.883895102	0.173	0.095	0.000110319

Chd1	2.88E-24	0.883711893	0.474	0.319	1.59E-19
Brd4	1.68E-30	0.883480111	0.568	0.41	9.32E-26
1110038B12Rik	6.03E-35	0.881880307	0.651	0.534	3.34E-30
Creb3l3	2.38E-24	0.881731051	0.468	0.336	1.32E-19
Mthfr	5.34E-07	0.878275185	0.111	0.057	0.029562162
Peli1	5.79E-07	0.878080614	0.134	0.075	0.032079812
Rab31	9.11E-13	0.877272959	0.267	0.163	5.05E-08
Cerk	2.77E-16	0.876392867	0.311	0.181	1.53E-11
Adamts1	5.62E-18	0.875160527	0.57	0.44	3.11E-13
Cdk5r1	2.38E-09	0.87456715	0.224	0.147	0.000132125
Kmt2d	1.12E-09	0.873741497	0.207	0.122	6.20E-05
Nfkb2	7.19E-10	0.872569354	0.202	0.117	3.98E-05
Map4k4	5.85E-46	0.868055115	0.724	0.562	3.24E-41
Klf4	7.34E-73	0.866811413	0.941	0.93	4.06E-68
Cdkn1a	1.14E-58	0.865985097	0.945	0.901	6.31E-54
Igf2r	3.79E-23	0.863974365	0.447	0.276	2.10E-18
Rin2	3.35E-09	0.862785443	0.202	0.122	0.000185699
Ppm1d	6.18E-10	0.861727079	0.231	0.143	3.42E-05
Tmbim1	9.73E-13	0.86147793	0.287	0.179	5.39E-08
Efna1	2.46E-12	0.860489813	0.291	0.206	1.36E-07
Fam167a	6.54E-09	0.860213089	0.2	0.127	0.000362306
Plk2	7.92E-07	0.86018793	0.192	0.124	0.043890089
5430416N02Rik	1.25E-20	0.859060017	0.451	0.301	6.95E-16
Usp32	1.69E-07	0.858813363	0.14	0.077	0.009377781
Insig1	1.87E-25	0.858757441	0.521	0.385	1.03E-20
Khsrp	4.79E-13	0.85747394	0.272	0.162	2.65E-08
Lima1	8.11E-14	0.855762525	0.31	0.207	4.50E-09
D16Ertd472e	9.79E-23	0.855399072	0.483	0.343	5.42E-18
Nt5dc3	3.22E-07	0.852093752	0.131	0.071	0.017838576
Tnks1bp1	1.63E-10	0.85163663	0.183	0.099	9.02E-06
Scaf8	7.16E-07	0.851615964	0.156	0.107	0.039658752
Kctd20	2.81E-15	0.850995939	0.319	0.194	1.56E-10
Mllt6	1.49E-09	0.849018605	0.207	0.122	8.24E-05
Panx3	2.30E-07	0.848919344	0.195	0.131	0.012732356
Nomo1	1.34E-10	0.848504666	0.247	0.156	7.45E-06
Tubb2a	4.71E-15	0.847277797	0.397	0.309	2.61E-10
Sardh	3.27E-10	0.846743232	0.211	0.122	1.81E-05
Trp53bp2	1.07E-12	0.84557557	0.276	0.166	5.95E-08
Kctd12	2.20E-10	0.844594108	0.235	0.142	1.22E-05
Elk4	1.81E-11	0.844120676	0.266	0.172	1.01E-06
Spred2	2.33E-08	0.841568672	0.187	0.112	0.001291398
Plekhf1	1.26E-09	0.841257916	0.19	0.107	7.00E-05
Esr1	7.94E-07	0.841233163	0.147	0.085	0.043966575
Suco	1.28E-17	0.840778607	0.397	0.273	7.10E-13
Stim2	6.33E-17	0.839070508	0.377	0.251	3.51E-12
Fam102b	1.85E-08	0.838707091	0.193	0.118	0.001023934
Gm37376	1.12E-08	0.838657012	0.257	0.185	0.000618225

Glul	3.69E-08	0.834113503	0.218	0.145	0.002046805
Mafg	8.11E-17	0.833621112	0.389	0.266	4.49E-12
Notch2	1.66E-19	0.833513494	0.453	0.322	9.19E-15
Tmem131	7.56E-11	0.832771936	0.252	0.17	4.19E-06
Nfkb1	1.67E-09	0.831563761	0.224	0.141	9.25E-05
D10Wsu102e	1.15E-10	0.831447016	0.327	0.229	6.39E-06
Ptbp1	1.07E-43	0.829594537	0.759	0.628	5.91E-39
Ulk1	2.41E-10	0.828443402	0.221	0.13	1.33E-05
Mxd1	1.20E-08	0.827889082	0.187	0.11	0.000664642
Msi1	2.75E-10	0.827848102	0.215	0.125	1.52E-05
Myo18a	1.15E-23	0.826744683	0.502	0.336	6.39E-19
Gab1	2.34E-07	0.82632393	0.151	0.086	0.012973891
Bbc3	6.00E-17	0.82624094	0.391	0.261	3.32E-12
Rai1	6.08E-10	0.82577766	0.231	0.141	3.37E-05
Hif1a	1.42E-07	0.8250779	0.175	0.108	0.007852578
Actn1	8.28E-22	0.824663474	0.464	0.314	4.59E-17
Scx	2.72E-10	0.82449936	0.27	0.182	1.51E-05
Tns2	5.20E-07	0.823710132	0.159	0.095	0.028806366
Wwtr1	2.13E-35	0.822949918	0.662	0.528	1.18E-30
Cyth3	1.07E-08	0.822692013	0.171	0.097	0.000593336
Fnip1	1.20E-21	0.82126884	0.459	0.296	6.67E-17
Csrp2	1.03E-10	0.819498365	0.299	0.215	5.73E-06
Phldb1	1.21E-18	0.818215717	0.425	0.285	6.69E-14
Igf1r	7.15E-08	0.817301108	0.195	0.127	0.003964084
Ppp4r1	4.72E-09	0.816999954	0.185	0.105	0.000261495
Ssh2	1.37E-07	0.816558303	0.183	0.113	0.007591888
Runx3	1.04E-07	0.815493129	0.203	0.138	0.005773053
Slc39a14	2.17E-17	0.815428883	0.4	0.272	1.20E-12
Plxnb1	4.24E-11	0.81533858	0.267	0.168	2.35E-06
Nr1d2	8.84E-09	0.814192126	0.2	0.119	0.000489871
Klhl24	4.47E-21	0.813949097	0.472	0.321	2.48E-16
Rev3l	5.91E-07	0.811549482	0.17	0.105	0.032742259
Agap1	3.20E-07	0.811220865	0.171	0.104	0.01771174
Xdh	1.76E-09	0.810079884	0.193	0.11	9.77E-05
Bcor	1.81E-12	0.808449577	0.248	0.142	1.00E-07
Fam117b	1.28E-11	0.807228962	0.234	0.134	7.09E-07
Fasn	9.15E-11	0.806083777	0.248	0.15	5.07E-06
Rnf19a	9.75E-14	0.805685222	0.315	0.196	5.40E-09
Prrc2a	5.38E-30	0.805229638	0.584	0.395	2.98E-25
Samhd1	8.98E-08	0.803695365	0.201	0.129	0.004975952
Kdm4a	1.31E-08	0.80306554	0.199	0.12	0.000727981
Fzd5	1.33E-16	0.801510342	0.392	0.261	7.34E-12
Slc3a2	3.01E-35	0.801204278	0.651	0.56	1.67E-30
Oaf	5.39E-07	0.800562549	0.179	0.117	0.029852608
Psmb9	1.77E-12	0.800376313	0.312	0.206	9.81E-08
Loxl4	5.03E-13	0.800197265	0.313	0.204	2.79E-08
Erf	7.99E-07	0.797811801	0.16	0.097	0.044283464

Appl2	2.36E-09	0.797224476	0.234	0.153	0.000130972
Maff	8.22E-17	0.797091161	0.446	0.307	4.56E-12
Vcl	4.67E-09	0.794496984	0.239	0.155	0.000258561
Fzd8	2.69E-10	0.793977197	0.309	0.244	1.49E-05
Rusc2	7.06E-07	0.793621263	0.144	0.083	0.039127584
Piezo1	3.97E-19	0.793272209	0.445	0.294	2.20E-14
Nudt4	1.81E-27	0.792500496	0.6	0.45	1.00E-22
Smurf2	6.11E-17	0.790868196	0.406	0.291	3.38E-12
Med13l	7.93E-27	0.788502065	0.584	0.413	4.39E-22
Igfbp4	1.93E-25	0.787886964	0.368	0.194	1.07E-20
Dipk2a	1.31E-28	0.786072602	0.655	0.518	7.24E-24
Ptgfrn	1.16E-13	0.785860902	0.338	0.224	6.41E-09
Ptprf	1.23E-13	0.781354876	0.37	0.256	6.84E-09
Tpm2	3.21E-09	0.78109921	0.291	0.208	0.000178022
Gadd45g	2.80E-36	0.780421042	0.953	0.946	1.55E-31
Ust	4.09E-29	0.77921484	0.599	0.412	2.26E-24
Gm3511	5.91E-16	0.779132845	0.369	0.239	3.28E-11
Bmpr2	4.92E-26	0.778932918	0.556	0.398	2.72E-21
Ubr5	2.58E-19	0.775818832	0.471	0.333	1.43E-14
Pnrc1	5.98E-52	0.774046503	0.843	0.787	3.31E-47
Epas1	1.23E-09	0.773842628	0.247	0.156	6.79E-05
Ppfibp1	7.82E-14	0.773021341	0.338	0.215	4.33E-09
Pmm1	5.79E-09	0.772494016	0.263	0.187	0.000320622
Nedd9	6.58E-13	0.771158019	0.379	0.273	3.65E-08
Zswim6	2.00E-16	0.771069128	0.405	0.266	1.11E-11
Fbxw7	1.18E-09	0.768781095	0.276	0.189	6.56E-05
Mdk	1.20E-08	0.768109664	0.258	0.173	0.000662456
Ptk2	8.91E-11	0.768095329	0.243	0.146	4.93E-06
Ube2h	1.09E-26	0.767514147	0.56	0.372	6.02E-22
Slc2a13	2.06E-10	0.766685798	0.244	0.148	1.14E-05
Sik3	8.98E-10	0.76617265	0.26	0.174	4.98E-05
Elf1	9.07E-13	0.765408856	0.339	0.242	5.02E-08
Ehbp1l1	4.61E-07	0.763506518	0.209	0.142	0.025565217
Cad	1.07E-09	0.763272289	0.248	0.156	5.91E-05
Plekho1	7.82E-11	0.761559757	0.25	0.151	4.33E-06
Gramd1a	2.22E-08	0.761233348	0.243	0.164	0.001229517
Pxdc1	2.71E-28	0.761173921	0.701	0.608	1.50E-23
Hk1	5.29E-14	0.759521847	0.269	0.153	2.93E-09
Cblb	1.96E-10	0.757506282	0.268	0.169	1.08E-05
Klf13	1.09E-33	0.755714483	0.682	0.568	6.06E-29
Lemd3	3.62E-07	0.753398495	0.185	0.116	0.020050571
Tap1	2.04E-07	0.753035507	0.214	0.143	0.011282973
Atf6	3.96E-13	0.749812187	0.353	0.236	2.19E-08
Poldip3	9.19E-11	0.749696203	0.299	0.209	5.09E-06
Agpat4	1.06E-09	0.749547403	0.275	0.185	5.87E-05
Mertk	7.61E-10	0.748515306	0.294	0.203	4.21E-05
Msl2	9.62E-10	0.748117957	0.244	0.151	5.33E-05

Chd2	7.95E-13	0.74514447	0.364	0.249	4.41E-08
Hdac5	2.24E-16	0.745113905	0.421	0.284	1.24E-11
Tjp1	1.57E-16	0.745078684	0.438	0.324	8.72E-12
Rapgef2	2.89E-07	0.744926592	0.182	0.112	0.016016694
Serping1	2.60E-18	0.7434814	0.474	0.318	1.44E-13
Tet2	2.56E-07	0.74272117	0.151	0.087	0.014182832
Atf5	5.73E-18	0.741983296	0.507	0.366	3.17E-13
Stox2	8.42E-16	0.741640224	0.394	0.255	4.66E-11
Zfp516	6.33E-08	0.738525278	0.224	0.15	0.003509612
Usp37	1.73E-07	0.738002634	0.204	0.129	0.009602032
Creld2	1.73E-13	0.735032385	0.553	0.45	9.59E-09
Ppp1r9b	1.77E-08	0.734643971	0.242	0.158	0.00098133
Hivep2	1.35E-07	0.733866324	0.228	0.158	0.007501387
Usp22	6.48E-10	0.732715262	0.269	0.175	3.59E-05
Klf10	1.88E-07	0.732587723	0.228	0.161	0.010406835
Gns	7.46E-08	0.731727783	0.203	0.126	0.004130661
Hs2st1	8.91E-09	0.730277164	0.23	0.145	0.000493619
Boc	1.45E-13	0.729750777	0.38	0.259	8.05E-09
Ehmt2	2.13E-11	0.728980864	0.32	0.211	1.18E-06
Slc66a2	4.72E-11	0.727164301	0.339	0.239	2.62E-06
Rel	3.14E-08	0.727033294	0.262	0.181	0.001741215
Klf9	1.90E-42	0.723857727	0.856	0.766	1.05E-37
Sod2	1.97E-07	0.723518407	0.19	0.118	0.010895248
Sde2	9.14E-24	0.723511082	0.614	0.487	5.06E-19
Sos1	1.64E-07	0.720266808	0.2	0.126	0.00908225
Txnrd1	2.81E-15	0.716851231	0.477	0.363	1.56E-10
Ppp1r18	4.06E-07	0.716272315	0.224	0.153	0.02250747
Sphk1	1.92E-10	0.714722913	0.335	0.237	1.06E-05
Slit3	2.84E-14	0.713020558	0.421	0.299	1.58E-09
Gatad2b	1.60E-11	0.708610277	0.345	0.243	8.87E-07
Arnt	8.25E-09	0.70731174	0.243	0.155	0.000456853
Timp1	5.71E-24	0.70649377	0.889	0.814	3.17E-19
Maml3	3.71E-10	0.704224074	0.319	0.22	2.06E-05
Zfp281	1.47E-07	0.703976922	0.224	0.146	0.008138858
Hnrnph1	7.22E-29	0.703097275	0.699	0.528	4.00E-24
Baiap2	3.57E-10	0.70246514	0.284	0.183	1.98E-05
Gpc1	5.58E-58	0.702139108	0.943	0.872	3.09E-53
Dhx40	5.10E-12	0.701414489	0.348	0.235	2.83E-07
Arid1b	8.11E-19	0.701061259	0.466	0.306	4.49E-14
Dyrk1a	2.17E-10	0.700866776	0.296	0.193	1.20E-05
Jmy	2.39E-13	0.699880496	0.383	0.287	1.32E-08
Fus	1.19E-29	0.699306187	0.698	0.53	6.59E-25
Srsf5	1.13E-39	0.699189162	0.787	0.691	6.29E-35
Nfe2l2	1.19E-21	0.696190528	0.61	0.483	6.59E-17
Gabarapl1	4.24E-15	0.695743273	0.453	0.35	2.35E-10
Scd2	1.82E-13	0.695273785	0.411	0.311	1.01E-08
Igsf3	3.36E-16	0.695009652	0.489	0.357	1.86E-11

Zbtb2	2.15E-07	0.694703714	0.219	0.142	0.011890635
Pgd	2.76E-10	0.694177956	0.311	0.219	1.53E-05
Tcof1	5.16E-08	0.693781834	0.234	0.152	0.002861424
Vars	4.65E-07	0.693185816	0.236	0.163	0.02577602
Coro1c	1.17E-10	0.692916531	0.314	0.209	6.50E-06
Picalm	6.54E-26	0.689252861	0.633	0.5	3.62E-21
Myo1d	3.49E-20	0.68859163	0.546	0.392	1.94E-15
Spen	1.89E-14	0.68798122	0.394	0.261	1.05E-09
Tapbp	3.15E-17	0.686262829	0.506	0.371	1.74E-12
Erc1	8.12E-07	0.685672613	0.214	0.143	0.044988484
Msi2	1.23E-14	0.685176009	0.439	0.315	6.84E-10
Tmem183a	6.49E-07	0.682657045	0.226	0.156	0.035932983
Tiparp	4.42E-16	0.681837654	0.563	0.468	2.45E-11
Chka	1.45E-14	0.681498566	0.433	0.298	8.06E-10
Copa	2.32E-42	0.679914241	0.823	0.672	1.28E-37
Atp2a2	4.10E-07	0.679266693	0.228	0.159	0.022727397
Sdf2l1	8.57E-07	0.676588005	0.273	0.195	0.047502727
Ube2o	1.94E-07	0.67649313	0.185	0.113	0.010740626
Baz1a	3.79E-07	0.676018785	0.24	0.17	0.02098108
Gna13	8.98E-14	0.675879714	0.431	0.319	4.97E-09
Mon2	9.14E-14	0.675047039	0.411	0.282	5.06E-09
Tpcn1	2.77E-09	0.674657987	0.244	0.153	0.00015373
Kdm5a	2.65E-18	0.674399562	0.538	0.402	1.47E-13
Ttc7	3.18E-09	0.673231791	0.312	0.223	0.000176185
Chst11	4.23E-21	0.672521413	0.585	0.446	2.34E-16
Rn18s	1.13E-22	0.672189267	1	1	6.29E-18
Sec24a	8.23E-15	0.672137304	0.443	0.316	4.56E-10
Irf2bp2	6.51E-25	0.670233534	0.654	0.482	3.61E-20
Plxnb2	2.23E-14	0.670032994	0.371	0.238	1.24E-09
Gadd45b	1.55E-25	0.666906458	0.873	0.848	8.61E-21
Ttc39b	2.31E-09	0.666782666	0.322	0.245	0.000128145
Pdlim4	4.25E-07	0.666769947	0.237	0.162	0.023559411
Zfhx4	1.80E-20	0.666769545	0.589	0.441	9.96E-16
Rasa2	8.01E-12	0.666693909	0.368	0.252	4.44E-07
Sec16b	3.32E-07	0.665685107	0.224	0.147	0.018392118
Mrc2	2.23E-40	0.665556015	0.865	0.694	1.23E-35
Ywhaz	4.24E-35	0.664120754	0.782	0.655	2.35E-30
Marchf6	2.20E-07	0.663180817	0.255	0.177	0.012214884
Myo10	1.26E-46	0.663137961	0.889	0.771	6.98E-42
Ubr2	1.22E-12	0.661838467	0.425	0.319	6.75E-08
Garre1	2.48E-10	0.661183933	0.305	0.201	1.37E-05
Kdm5c	5.96E-10	0.660513971	0.333	0.234	3.30E-05
Smg7	7.67E-08	0.659935803	0.228	0.147	0.00424884
Actg1	2.22E-10	0.659788656	0.347	0.247	1.23E-05
Slc25a25	8.36E-08	0.6593993	0.267	0.193	0.004633764
Rhob	6.01E-16	0.659294845	0.529	0.451	3.33E-11
Fam168b	1.24E-09	0.658083327	0.309	0.21	6.90E-05

Mbtps1	7.82E-14	0.656826978	0.42	0.29	4.33E-09
Cdc42bpa	5.23E-10	0.654858102	0.334	0.235	2.90E-05
Kansl3	5.65E-07	0.652832286	0.236	0.162	0.031321188
Eprs	4.88E-54	0.650392479	0.907	0.834	2.70E-49
Clip1	1.16E-08	0.649625997	0.303	0.212	0.000642734
Acox1	3.83E-08	0.64941498	0.232	0.148	0.002120193
Brd2	2.62E-37	0.649305038	0.885	0.817	1.45E-32
Lrrfip1	1.87E-07	0.649033191	0.243	0.162	0.010339664
Lrp1	8.70E-35	0.648558544	0.792	0.627	4.82E-30
Tgfbr2	2.39E-11	0.64814933	0.309	0.198	1.33E-06
Rela	2.53E-11	0.648059537	0.377	0.295	1.40E-06
Nab2	4.88E-10	0.647853824	0.356	0.273	2.70E-05
Clic4	6.85E-19	0.647803123	0.589	0.466	3.80E-14
Lrp4	8.74E-18	0.646658495	0.586	0.444	4.84E-13
Acot9	5.42E-07	0.646359783	0.247	0.179	0.030014085
Amotl2	4.48E-16	0.646033721	0.536	0.415	2.48E-11
Vdr	2.80E-24	0.64469052	0.721	0.578	1.55E-19
Aff1	1.77E-07	0.643671826	0.269	0.191	0.009826412
Rtn4rl1	1.06E-09	0.640988746	0.337	0.239	5.87E-05
Ccnl1	1.22E-28	0.640724896	0.817	0.706	6.78E-24
Gfpt1	3.06E-15	0.640595289	0.489	0.359	1.69E-10
Ankrd12	3.17E-13	0.639268658	0.483	0.384	1.76E-08
Ranbp2	7.29E-13	0.639215301	0.44	0.32	4.04E-08
Lasp1	1.66E-11	0.639043599	0.394	0.287	9.22E-07
Srgap2	6.03E-20	0.636787839	0.595	0.447	3.34E-15
Rapgef6	4.29E-07	0.635935459	0.264	0.191	0.023745318
Map7d1	9.96E-28	0.634230209	0.745	0.633	5.52E-23
B4galt1	5.99E-12	0.633950799	0.411	0.304	3.32E-07
Phf13	9.37E-08	0.63281408	0.267	0.182	0.005188796
Xpot	1.31E-08	0.632782158	0.309	0.222	0.000726401
Usp9x	3.35E-08	0.631528447	0.287	0.198	0.00185417
Qsox1	1.31E-10	0.63109869	0.391	0.309	7.25E-06
Map4k3	3.85E-18	0.629510167	0.566	0.416	2.13E-13
Adam12	1.27E-13	0.627689844	0.474	0.368	7.06E-09
Pard3	4.05E-08	0.626091082	0.303	0.223	0.002241962
Ilrun	3.18E-08	0.624857527	0.297	0.208	0.001761253
Cebpb	4.48E-18	0.624056806	0.781	0.753	2.48E-13
Asap1	5.88E-33	0.623895267	0.758	0.647	3.26E-28
Sec16a	1.99E-12	0.622675216	0.393	0.27	1.10E-07
Slc35f5	2.09E-10	0.622265919	0.338	0.229	1.16E-05
Smtn	7.50E-11	0.621272539	0.378	0.268	4.16E-06
Wipi2	1.36E-07	0.620269842	0.275	0.19	0.007557787
Wdr26	3.03E-21	0.619572502	0.642	0.509	1.68E-16
Cbx4	2.01E-09	0.618216413	0.35	0.251	0.000111608
Ythdc1	2.32E-12	0.617711627	0.432	0.33	1.29E-07
Tspan11	5.74E-07	0.615734926	0.283	0.211	0.031794993
Actr3	1.40E-22	0.615569669	0.66	0.552	7.76E-18

Tbc1d15	1.64E-18	0.615228826	0.588	0.487	9.09E-14
Tspo	9.47E-20	0.61497012	0.623	0.521	5.25E-15
Tcf7l2	9.58E-12	0.614853856	0.441	0.338	5.31E-07
Tgfb1	6.57E-39	0.614142838	0.838	0.748	3.64E-34
Wdr43	1.44E-11	0.614032618	0.423	0.31	7.99E-07
Sema6d	2.57E-07	0.613477472	0.293	0.214	0.014222064
Larp7	6.93E-08	0.61268707	0.303	0.224	0.003841347
Rassf1	3.24E-14	0.612351601	0.513	0.414	1.80E-09
Samd4	5.29E-17	0.611433843	0.567	0.438	2.93E-12
Syvn1	1.47E-08	0.610097743	0.28	0.187	0.000812019
Col22a1	4.50E-73	0.610046255	0.988	0.954	2.50E-68
Fndc3b	2.94E-36	0.609935421	0.847	0.73	1.63E-31
Siva1	1.15E-11	0.609810111	0.442	0.329	6.39E-07
Tor1aip2	1.35E-08	0.609203071	0.25	0.161	0.000748301
Rasgrp2	3.91E-07	0.609097824	0.279	0.221	0.021646626
Prex1	1.32E-16	0.606916936	0.563	0.426	7.31E-12
Myo1b	4.25E-33	0.606164266	0.803	0.722	2.35E-28
Lars	3.82E-14	0.605790201	0.502	0.387	2.11E-09
Scaf11	4.70E-21	0.605476078	0.654	0.535	2.60E-16
Zfp36	1.37E-25	0.603308892	0.861	0.811	7.58E-21
Aars	2.68E-11	0.603174207	0.397	0.281	1.48E-06
Ipo5	1.42E-08	0.602273614	0.305	0.21	0.000784247
Eef2k	3.09E-08	0.600532647	0.325	0.239	0.001711437
Atf4	3.59E-47	0.600505883	0.924	0.902	1.99E-42
Lats1	1.82E-07	0.599382406	0.255	0.172	0.010100229
Antxr1	2.47E-09	0.599062997	0.366	0.269	0.00013708
Ttc28	6.84E-09	0.598826635	0.355	0.265	0.000379231
Anxa2	3.52E-25	0.598793246	0.781	0.752	1.95E-20
Mcl1	3.19E-26	0.59797293	0.803	0.745	1.77E-21
Galnt10	1.73E-07	0.59777311	0.192	0.12	0.00958674
U2af1	2.66E-16	0.597261689	0.55	0.411	1.47E-11
Pom121	7.93E-07	0.595150845	0.289	0.218	0.043910259
Tmem64	7.49E-09	0.593563299	0.344	0.253	0.000414712
Eif4ebp1	1.31E-28	0.593351877	0.724	0.674	7.26E-24
Phip	4.32E-11	0.593340644	0.445	0.34	2.39E-06
Vgll4	2.81E-11	0.593267113	0.442	0.338	1.55E-06
Auts2	1.05E-10	0.59322823	0.444	0.341	5.82E-06
Polr2b	6.27E-07	0.593048238	0.268	0.19	0.034755166
Prrc2c	1.40E-24	0.593006331	0.738	0.639	7.77E-20
Oxct1	5.01E-13	0.5923461	0.484	0.362	2.78E-08
Tnrc6b	1.38E-13	0.591184152	0.508	0.392	7.67E-09
Lrch1	1.85E-10	0.591132791	0.358	0.246	1.03E-05
Smad3	3.40E-07	0.58968938	0.309	0.239	0.018834172
Prdm2	7.32E-11	0.58894819	0.41	0.294	4.06E-06
Avpi1	1.98E-11	0.58799845	0.471	0.383	1.10E-06
Pcyt1a	3.24E-08	0.586100653	0.33	0.243	0.001795588
Ski	4.11E-11	0.586047006	0.431	0.327	2.28E-06

Slc39a1	2.95E-10	0.584663177	0.425	0.33	1.63E-05
Ptpn14	1.77E-07	0.583784685	0.31	0.227	0.009801342
Srgap1	7.76E-10	0.583007652	0.411	0.32	4.30E-05
Npc1	3.61E-07	0.58283023	0.221	0.145	0.019999191
Col5a1	2.80E-73	0.582096626	0.989	0.954	1.55E-68
Eif4g1	1.14E-21	0.581981287	0.701	0.555	6.32E-17
Mif	2.00E-18	0.581476197	0.67	0.541	1.11E-13
Pdgfc	2.70E-12	0.581056121	0.476	0.368	1.50E-07
Sec24d	6.72E-30	0.579345603	0.742	0.643	3.72E-25
Cemip	2.00E-10	0.578952892	0.411	0.302	1.11E-05
Map3k2	2.26E-08	0.578933234	0.348	0.257	0.001251469
Clint1	2.68E-36	0.578769534	0.873	0.773	1.49E-31
Zranb1	7.40E-10	0.578154332	0.411	0.314	4.10E-05
Cdr2l	1.99E-15	0.577957983	0.554	0.446	1.10E-10
Rbm27	5.86E-10	0.577133408	0.404	0.307	3.25E-05
H2-DMb1	3.36E-11	0.577004474	0.298	0.19	1.86E-06
Erbin	3.98E-09	0.576746219	0.334	0.233	0.000220765
4932438A13Rik	3.78E-07	0.576223	0.278	0.195	0.020936723
Arid2	1.50E-07	0.575180616	0.314	0.246	0.008328533
Calr	2.51E-37	0.574183131	0.97	0.918	1.39E-32
Ddx3x	2.63E-28	0.573757857	0.849	0.731	1.46E-23
Kdm5b	2.74E-15	0.572387658	0.564	0.443	1.52E-10
Ftl1	1.89E-38	0.571955964	0.925	0.887	1.05E-33
Ifitm3	8.86E-24	0.571010442	0.893	0.812	4.91E-19
Nrip1	1.37E-07	0.568924719	0.329	0.243	0.007601795
Kcnq1ot1	3.36E-14	0.567160775	0.513	0.371	1.86E-09
Baz2b	1.16E-10	0.563874524	0.44	0.33	6.41E-06
Mthfd2	1.62E-12	0.562692158	0.502	0.42	8.95E-08
Rps28	9.35E-62	0.561875662	0.959	0.91	5.18E-57
Fbxo28	7.03E-09	0.561173703	0.37	0.271	0.000389671
Cab39	4.81E-09	0.560454288	0.377	0.281	0.000266262
Crebbp	5.99E-14	0.560289403	0.547	0.425	3.32E-09
Lrp6	4.68E-07	0.55634138	0.294	0.21	0.02590893
Klf6	2.79E-18	0.556261629	0.8	0.725	1.55E-13
Zfp131	7.53E-11	0.555878781	0.478	0.374	4.17E-06
Srrt	2.50E-07	0.554082108	0.327	0.244	0.013869558
Gas5	1.16E-58	0.553341779	0.982	0.973	6.40E-54
Capn2	1.35E-14	0.553256441	0.548	0.438	7.48E-10
Grb10	4.38E-12	0.55282776	0.507	0.392	2.43E-07
Tor1aip1	2.02E-11	0.551979101	0.469	0.368	1.12E-06
Ltbp3	9.14E-12	0.551848551	0.484	0.367	5.06E-07
Fads2	1.44E-07	0.551425826	0.36	0.278	0.007996037
Sertad2	4.06E-15	0.548100109	0.613	0.502	2.25E-10
Zfp469	1.21E-13	0.547308147	0.541	0.42	6.71E-09
Ube2j1	5.34E-11	0.546603341	0.445	0.361	2.96E-06
Atg101	3.16E-10	0.545448396	0.454	0.379	1.75E-05
Bzw1	1.80E-35	0.543555271	0.86	0.806	9.96E-31

Inppl1	7.21E-10	0.542433267	0.377	0.266	4.00E-05
Sdk2	4.12E-13	0.540900068	0.543	0.416	2.28E-08
Rad21	4.90E-09	0.540513813	0.396	0.299	0.000271662
Cltc	1.71E-21	0.538773646	0.748	0.625	9.46E-17
Ptpn4	3.37E-10	0.537797151	0.451	0.34	1.87E-05
Kmt5b	1.70E-09	0.537745679	0.423	0.322	9.44E-05
Zfp36l1	3.33E-17	0.536939915	0.786	0.721	1.85E-12
Trip12	7.94E-11	0.536885913	0.435	0.318	4.40E-06
Frmd4b	2.10E-09	0.536608339	0.421	0.317	0.000116285
Glrx5	1.32E-22	0.536467474	0.745	0.676	7.30E-18
Abr	2.54E-08	0.536371611	0.316	0.22	0.001408271
Fth1	2.32E-38	0.536223289	0.998	0.997	1.29E-33
Cxxc5	7.01E-09	0.535125385	0.399	0.313	0.000388216
Eif3b	5.25E-14	0.534556122	0.577	0.459	2.91E-09
Pkp4	4.03E-09	0.534175162	0.411	0.316	0.000223024
Tpst2	3.87E-13	0.533100788	0.536	0.449	2.14E-08
Pmepa1	2.88E-15	0.532463977	0.597	0.574	1.60E-10
Ncl	3.23E-36	0.532424508	0.959	0.914	1.79E-31
Birc6	8.47E-15	0.532375642	0.601	0.486	4.69E-10
Palld	4.86E-09	0.531351339	0.454	0.359	0.00026932
Cul3	1.47E-10	0.530511193	0.474	0.366	8.15E-06
Smchd1	1.11E-11	0.530493346	0.51	0.414	6.16E-07
Fbxo11	2.32E-07	0.529413119	0.337	0.248	0.012833277
Itga11	2.58E-07	0.527114371	0.365	0.282	0.014300034
Enah	1.31E-08	0.526147199	0.405	0.306	0.000724766
Tshz1	3.73E-07	0.525439934	0.351	0.28	0.020645966
Rps6	3.07E-36	0.523180362	0.896	0.806	1.70E-31
Tmem184b	5.78E-08	0.523098289	0.353	0.258	0.003200375
Edem3	6.56E-08	0.522461118	0.3	0.209	0.003636833
Rora	1.78E-10	0.520038854	0.478	0.408	9.87E-06
Tnpo1	2.19E-07	0.519084183	0.304	0.215	0.012151526
Ap2a2	5.58E-07	0.518848604	0.353	0.276	0.030924382
Actn4	2.95E-11	0.51873515	0.522	0.411	1.63E-06
Zfp622	4.07E-09	0.517675079	0.462	0.378	0.000225572
Pkdcc	1.96E-08	0.516974298	0.373	0.327	0.001087658
Kpnb1	1.28E-07	0.516124055	0.366	0.275	0.007118234
Safb	3.40E-08	0.515018517	0.363	0.265	0.001886041
Ubl3	8.42E-11	0.514448901	0.475	0.398	4.66E-06
Pabpc1	3.29E-40	0.511807338	0.939	0.873	1.82E-35
Dusp1	9.97E-14	0.511780352	0.751	0.698	5.53E-09
Nr3c1	5.13E-13	0.50649857	0.567	0.465	2.84E-08
Tanc1	5.15E-12	0.506440393	0.547	0.434	2.85E-07
Kdm2a	5.62E-07	0.50579507	0.348	0.262	0.031116202
Ifitm2	2.52E-10	0.505760122	0.493	0.386	1.40E-05
Cdc73	1.32E-10	0.504897118	0.5	0.401	7.31E-06
Olfml3	1.52E-25	0.504590949	0.881	0.846	8.40E-21
Creg1	4.23E-12	0.504587769	0.525	0.442	2.34E-07

Resf1	1.18E-11	0.504567989	0.702	0.609	6.52E-07
Atxn2l	6.81E-09	0.504452941	0.417	0.314	0.000377094
Dyrk2	2.30E-09	0.503997147	0.441	0.335	0.000127468
Gm10076	1.41E-42	0.501056256	0.942	0.883	7.79E-38
Lrrc58	2.20E-14	0.500922672	0.626	0.514	1.22E-09
Tab2	3.38E-09	0.500906659	0.477	0.377	0.000187185
Tmod3	4.87E-07	0.497839657	0.373	0.3	0.027009299
Ptpn13	4.94E-08	0.497502897	0.413	0.318	0.002737164
Cebpz	1.28E-08	0.49735736	0.45	0.353	0.000709323
Pqbp1	2.30E-08	0.495624181	0.424	0.329	0.001273903
Jak1	1.06E-17	0.492849358	0.784	0.709	5.89E-13
Zc3h7a	4.53E-08	0.491582909	0.439	0.359	0.002508184
Pum1	8.79E-12	0.491192683	0.541	0.418	4.87E-07
Dync1h1	5.17E-11	0.491178608	0.528	0.41	2.86E-06
Taok1	3.85E-09	0.490272444	0.46	0.366	0.000213404

Pre-Obs_UP					
Gene	p_val	avg_log2FC	pct.1	pct.2	p_val_adj
Hhip	1.11E-37	7.929861201	0.147	0.003	6.16E-33
Vps37b	4.04E-30	4.091727199	0.161	0.016	2.24E-25
Lamc3	8.13E-44	3.89092353	0.234	0.026	4.50E-39
Cd28	1.65E-32	3.843183752	0.194	0.028	9.12E-28
Nrtn	3.31E-31	3.715228412	0.174	0.019	1.83E-26
Matn2	2.73E-20	3.530607076	0.115	0.013	1.51E-15
Gli2	2.32E-19	3.247346067	0.124	0.021	1.28E-14
Sik1	3.16E-43	3.220382314	0.275	0.05	1.75E-38
Enpp2	6.88E-31	3.068323085	0.27	0.081	3.81E-26
Thsd4	3.21E-14	2.926075236	0.107	0.024	1.78E-09
Nr4a3	6.51E-27	2.85866309	0.212	0.049	3.61E-22
Plaur	1.79E-34	2.769893745	0.274	0.069	9.92E-30
Tnfsf11	6.71E-34	2.72175244	0.349	0.132	3.72E-29
Notch1	2.28E-31	2.71616267	0.281	0.083	1.26E-26
Cdkn2a	4.98E-32	2.543116125	0.257	0.062	2.76E-27
Gli1	9.93E-16	2.368236628	0.132	0.03	5.50E-11
Fyb	2.83E-10	2.202734301	0.1	0.028	1.57E-05
Foxd1	2.99E-23	2.143696839	0.214	0.055	1.66E-18
Slc7a1	1.06E-15	2.140228573	0.167	0.052	5.85E-11
Foxc2	3.93E-15	2.101469459	0.174	0.061	2.18E-10
Ston2	2.36E-21	2.087358445	0.231	0.079	1.31E-16
Adamts12	3.22E-14	2.051214946	0.15	0.045	1.78E-09
Klhl21	1.31E-23	2.050855335	0.259	0.095	7.23E-19
Gdf15	2.72E-15	1.97960405	0.228	0.098	1.51E-10
Prkab2	5.88E-10	1.947762916	0.117	0.039	3.26E-05
Rgs2	9.21E-28	1.931586028	0.449	0.241	5.10E-23
Vegfa	3.38E-54	1.91729488	0.673	0.434	1.87E-49
Nfatc1	8.28E-11	1.908510877	0.138	0.055	4.59E-06
Eya1	2.13E-11	1.905170863	0.139	0.048	1.18E-06
Ptch1	3.49E-29	1.891128369	0.408	0.197	1.93E-24
Cryba4	2.52E-10	1.86718832	0.136	0.052	1.39E-05
Cenpl	7.09E-10	1.832422894	0.124	0.048	3.93E-05
Lmna	2.93E-54	1.824350944	0.804	0.638	1.62E-49
Abl2	8.57E-24	1.822303689	0.294	0.111	4.75E-19
Myo1e	6.09E-30	1.82169191	0.372	0.159	3.38E-25
Rcan2	1.22E-25	1.812917521	0.337	0.149	6.75E-21
Arl5b	3.49E-09	1.786017284	0.116	0.043	0.000194
Dusp10	2.45E-11	1.777842246	0.144	0.052	1.36E-06
Dysf	1.33E-09	1.744631028	0.124	0.048	7.36E-05
Ackr3	2.34E-09	1.740833424	0.162	0.077	0.000129
Yod1	7.73E-18	1.737959284	0.249	0.107	4.28E-13
Dact1	6.11E-27	1.736355559	0.357	0.149	3.39E-22
Uap1	1.04E-29	1.709125516	0.5	0.31	5.76E-25
Peli1	8.91E-13	1.685317519	0.179	0.072	4.94E-08
Srxn1	2.48E-10	1.684573375	0.144	0.055	1.37E-05

Cry2	1.67E-10	1.675288479	0.136	0.048	9.25E-06
Niban2	1.04E-19	1.666858104	0.282	0.129	5.76E-15
Tbc1d4	4.36E-11	1.666122477	0.156	0.061	2.42E-06
Mest	8.84E-10	1.655892127	0.129	0.045	4.90E-05
Mdm2	1.19E-25	1.636435929	0.438	0.264	6.58E-21
Has1	1.44E-14	1.603052657	0.224	0.092	7.98E-10
Ets1	1.34E-19	1.600428662	0.302	0.139	7.44E-15
Uaca	5.01E-26	1.600040747	0.395	0.231	2.77E-21
Arl4d	1.50E-23	1.582372292	0.418	0.267	8.29E-19
Tnfrsf12a	2.97E-30	1.577636838	0.509	0.288	1.64E-25
Gadd45a	6.21E-25	1.570643513	0.43	0.228	3.44E-20
Dot1l	2.96E-23	1.555270584	0.355	0.169	1.64E-18
Nr4a2	8.19E-29	1.550486911	0.526	0.324	4.53E-24
Tgif1	1.06E-20	1.544923534	0.331	0.164	5.87E-16
Hivep3	2.06E-15	1.539426007	0.228	0.091	1.14E-10
Lmbr1l	2.35E-08	1.538803316	0.137	0.062	0.001304
Hmgcr	2.36E-16	1.535169092	0.262	0.119	1.31E-11
Sesn2	1.85E-12	1.52682288	0.194	0.082	1.03E-07
Fasn	7.13E-11	1.521372804	0.179	0.083	3.95E-06
Sardh	1.36E-07	1.520507374	0.123	0.061	0.007553
Ldlr	2.18E-07	1.519946928	0.117	0.05	0.012095
Irx3	5.33E-24	1.519573382	0.471	0.308	2.95E-19
Fstl3	2.88E-10	1.50148082	0.168	0.074	1.59E-05
Eaf1	2.92E-11	1.486735365	0.192	0.088	1.62E-06
Rictor	1.26E-13	1.483609508	0.226	0.101	7.00E-09
Dusp5	4.41E-19	1.482649364	0.339	0.168	2.44E-14
Hk2	1.65E-30	1.48096051	0.503	0.281	9.17E-26
Nr4a1	1.09E-38	1.478273812	0.712	0.526	6.05E-34
Tubb6	5.18E-25	1.469758762	0.429	0.231	2.87E-20
Ddit4	2.40E-20	1.441522346	0.446	0.296	1.33E-15
Mafk	1.14E-26	1.438541971	0.423	0.211	6.30E-22
Pdk4	3.41E-17	1.435086436	0.338	0.187	1.89E-12
Bach1	2.38E-20	1.433193051	0.338	0.172	1.32E-15
Auts2	7.05E-34	1.431552732	0.615	0.417	3.91E-29
Nt5dc3	1.41E-08	1.428646883	0.11	0.038	0.000779
Hdac4	1.90E-10	1.427598488	0.175	0.076	1.05E-05
Egr3	2.13E-30	1.394715132	0.553	0.322	1.18E-25
Cd44	2.64E-20	1.393321344	0.415	0.242	1.47E-15
Myom1	5.42E-12	1.39098831	0.212	0.097	3.01E-07
Ddi2	4.73E-21	1.385158212	0.373	0.195	2.62E-16
Sned1	1.77E-07	1.379827434	0.142	0.071	0.009829
Crebrf	8.65E-12	1.379690254	0.219	0.105	4.79E-07
Slc7a5	7.38E-10	1.377188626	0.109	0.034	4.09E-05
Aopep	6.99E-09	1.376612518	0.167	0.077	0.000387
Sh3bp4	8.58E-14	1.369035484	0.257	0.126	4.76E-09
Susd6	8.69E-13	1.368924176	0.237	0.12	4.82E-08
Timp3	1.19E-32	1.348197688	0.633	0.371	6.62E-28

Errfi1	8.43E-44	1.341238424	0.744	0.504	4.67E-39
Map3k3	9.36E-08	1.337231799	0.145	0.074	0.005186
Rgcc	1.18E-31	1.336001533	0.725	0.596	6.56E-27
Rell1	6.18E-10	1.331666763	0.19	0.091	3.42E-05
Ppp1r15b	1.14E-15	1.331643728	0.304	0.16	6.29E-11
Fosl2	6.94E-24	1.330137186	0.466	0.285	3.84E-19
Pi4k2a	1.43E-09	1.329812829	0.185	0.101	7.93E-05
Tex30	5.13E-09	1.327796504	0.173	0.095	0.000284
Srebf2	1.08E-10	1.325688171	0.178	0.074	5.99E-06
C4b	1.03E-18	1.304193973	0.378	0.202	5.71E-14
Tamalin	3.56E-14	1.291913563	0.29	0.187	1.97E-09
Fcer1g	3.58E-33	1.291477976	0.487	0.218	1.98E-28
Kdm6b	1.45E-29	1.284748484	0.594	0.395	8.06E-25
Stk40	1.20E-13	1.283488491	0.267	0.131	6.63E-09
Pitpnc1	1.69E-11	1.273472282	0.255	0.14	9.36E-07
C1s1	1.57E-13	1.27036704	0.274	0.135	8.68E-09
Adgrf5	3.77E-17	1.265182579	0.303	0.137	2.09E-12
Mideas	1.22E-09	1.263672267	0.203	0.106	6.75E-05
Atp10a	1.42E-07	1.263243046	0.134	0.058	0.007891
Tagln2	6.31E-36	1.26054941	0.693	0.458	3.50E-31
Rabgef1	5.06E-09	1.257298264	0.188	0.096	0.00028
Lpin1	3.88E-08	1.254319981	0.174	0.09	0.002151
Ifi211	5.68E-07	1.237611419	0.15	0.076	0.031496
Ifrd1	1.62E-30	1.197888446	0.77	0.639	8.98E-26
Arhgap23	4.91E-17	1.197184896	0.368	0.214	2.72E-12
Fnip1	2.77E-18	1.196439518	0.412	0.259	1.54E-13
Adam30	8.80E-07	1.192318181	0.132	0.061	0.048747
Efna5	2.73E-08	1.191205711	0.195	0.107	0.001513
Ezr	3.31E-07	1.189376108	0.169	0.09	0.018353
Midn	7.50E-26	1.188705676	0.55	0.362	4.16E-21
Emid1	1.87E-08	1.179161171	0.192	0.106	0.001038
Cerk	4.23E-08	1.177988723	0.194	0.113	0.002345
Vcl	1.26E-15	1.177330302	0.364	0.217	7.00E-11
Cyp51	2.85E-09	1.172615153	0.23	0.129	0.000158
Bcl2l11	4.98E-13	1.171262469	0.311	0.194	2.76E-08
Nrp1	3.62E-14	1.171108074	0.389	0.253	2.01E-09
Sqstm1	8.01E-37	1.168947626	0.765	0.631	4.44E-32
Slc35e4	3.46E-17	1.167993107	0.416	0.262	1.92E-12
Mir6236	6.15E-52	1.161780412	0.992	0.946	3.40E-47
Ugcg	1.82E-07	1.159517536	0.177	0.117	0.010083
Nfkb1	2.15E-10	1.152807155	0.255	0.158	1.19E-05
Arhgef2	1.54E-11	1.152333212	0.219	0.1	8.51E-07
Plec	6.34E-16	1.138582276	0.401	0.256	3.51E-11
Syne2	1.46E-09	1.131684311	0.245	0.139	8.08E-05
Ssh1	1.03E-07	1.126334297	0.199	0.112	0.005728
Epas1	7.29E-25	1.126109646	0.541	0.324	4.04E-20
Mdfi	4.20E-10	1.124323834	0.25	0.146	2.33E-05

Neat1	4.22E-28	1.120535487	0.85	0.745	2.34E-23
Gm3511	7.05E-11	1.1198584	0.253	0.134	3.90E-06
Camsap2	1.32E-11	1.119650536	0.283	0.179	7.30E-07
Slc39a14	2.13E-13	1.119211548	0.327	0.193	1.18E-08
Klf7	3.74E-12	1.108624467	0.317	0.195	2.07E-07
Lbr	8.60E-11	1.108380668	0.261	0.141	4.77E-06
Arid5b	4.72E-27	1.104811962	0.618	0.398	2.62E-22
Fbxo32	3.23E-12	1.104039175	0.337	0.226	1.79E-07
Mapkapk2	4.03E-17	1.096804536	0.415	0.252	2.23E-12
Thbs2	6.17E-08	1.095456597	0.213	0.125	0.003416
Csrp1	1.88E-10	1.0909902	0.343	0.261	1.04E-05
Ptpn1	1.65E-26	1.089397451	0.631	0.458	9.16E-22
Snai1	4.57E-13	1.087883607	0.369	0.232	2.53E-08
Trio	3.22E-12	1.086459902	0.303	0.172	1.78E-07
Smim3	1.14E-08	1.078370661	0.218	0.116	0.000633
Ilrun	1.23E-10	1.076706284	0.267	0.15	6.81E-06
Marchf6	6.54E-09	1.075921465	0.232	0.141	0.000362
Slc40a1	1.83E-12	1.074730085	0.373	0.243	1.02E-07
Stox2	2.48E-14	1.074686378	0.359	0.203	1.37E-09
Atl2	9.96E-08	1.071378456	0.2	0.113	0.005516
Anxa1	3.26E-18	1.067028748	0.66	0.523	1.81E-13
Stard13	6.63E-07	1.066241321	0.184	0.106	0.036751
Gse1	3.21E-08	1.063792931	0.209	0.112	0.001777
Usp37	5.80E-07	1.063722056	0.183	0.11	0.03211
Wif1	5.66E-34	1.059396876	0.832	0.651	3.13E-29
Cox4i2	1.77E-08	1.059222937	0.219	0.119	0.000982
Sema4c	1.10E-07	1.057279264	0.214	0.127	0.006103
Olfml2a	1.68E-08	1.056865691	0.252	0.146	0.000933
Zfp703	1.41E-12	1.056852678	0.38	0.255	7.83E-08
Col16a1	1.32E-09	1.056844823	0.276	0.159	7.31E-05
Rin2	2.22E-10	1.049931898	0.284	0.168	1.23E-05
Mpp5	2.23E-08	1.049645201	0.215	0.115	0.001237
Ppp1r18	5.08E-09	1.047856188	0.249	0.15	0.000281
Kmt2d	2.31E-09	1.045196804	0.2	0.096	0.000128
Pip5k1a	7.90E-12	1.044423736	0.324	0.203	4.38E-07
Cflar	2.59E-08	1.043269194	0.233	0.137	0.001436
Per1	3.96E-07	1.042246136	0.179	0.093	0.02193
Furin	2.50E-15	1.041857454	0.398	0.245	1.39E-10
Sulf1	6.50E-09	1.040502169	0.251	0.142	0.00036
Slc3a2	3.87E-19	1.039943766	0.561	0.43	2.14E-14
Igf2r	5.27E-14	1.039474337	0.4	0.259	2.92E-09
Coq10b	1.47E-13	1.039448851	0.381	0.237	8.17E-09
Txnrd1	1.53E-17	1.039074127	0.473	0.298	8.46E-13
Chd7	7.67E-09	1.038261465	0.237	0.132	0.000425
Pim1	9.47E-29	1.035261809	0.686	0.488	5.25E-24
Nr1d1	1.00E-09	1.033277136	0.253	0.137	5.57E-05
Maml1	2.18E-07	1.028940435	0.17	0.084	0.012075

Tsr1	3.01E-07	1.023283367	0.21	0.13	0.016666
Zcchc14	6.46E-22	1.021895939	0.539	0.333	3.58E-17
Frmd4a	3.69E-10	1.020133086	0.261	0.14	2.05E-05
Ivns1abp	6.42E-12	1.019852336	0.355	0.218	3.56E-07
Ahctf1	6.25E-08	1.018573373	0.223	0.136	0.003461
Mknk2	1.83E-11	1.016478672	0.326	0.209	1.01E-06
Jmjd1c	4.20E-24	1.015370063	0.607	0.424	2.33E-19
Frmd6	3.48E-25	1.013288732	0.657	0.499	1.93E-20
Suco	2.72E-13	1.009672353	0.414	0.271	1.51E-08
Gramd1a	3.89E-09	1.008924604	0.219	0.112	0.000215
Myh9	2.49E-28	1.008451731	0.654	0.426	1.38E-23
Acox1	8.98E-09	1.008196049	0.264	0.166	0.000497
Serpine1	9.23E-08	1.007201631	0.255	0.159	0.005115
Chst15	5.09E-10	1.004206634	0.302	0.184	2.82E-05
Cebpa	1.11E-07	1.000107431	0.22	0.127	0.006173
Pdgfrb	2.26E-17	0.994515055	0.486	0.305	1.25E-12
Dennd5b	1.14E-08	0.991994535	0.257	0.163	0.00063
Rai1	1.70E-09	0.991677095	0.275	0.163	9.39E-05
Rb1cc1	3.35E-20	0.987641464	0.534	0.383	1.86E-15
Mylk	1.09E-21	0.979600167	0.579	0.382	6.02E-17
Lsp1	7.97E-07	0.978560497	0.143	0.068	0.044167
Prrc2a	1.76E-17	0.97778652	0.502	0.34	9.74E-13
Arid5a	1.73E-10	0.975756714	0.299	0.173	9.56E-06
Tpm2	3.75E-13	0.974959564	0.424	0.304	2.08E-08
Itpr1	1.25E-08	0.969716994	0.253	0.145	0.000694
Tln2	3.33E-13	0.968648415	0.38	0.226	1.85E-08
Trib1	3.77E-11	0.968010654	0.307	0.173	2.09E-06
Bok	4.52E-09	0.967857928	0.281	0.174	0.00025
Ccn2	3.62E-31	0.963224823	0.813	0.607	2.01E-26
Slc41a1	2.13E-07	0.963054876	0.238	0.158	0.011824
Btg2	1.58E-15	0.960853933	0.74	0.712	8.75E-11
Pcyt1a	1.16E-08	0.960567702	0.261	0.177	0.000643
Fosb	3.69E-39	0.957428915	0.919	0.818	2.05E-34
Maff	5.65E-18	0.956727535	0.534	0.356	3.13E-13
Map2k3	4.24E-16	0.956467532	0.44	0.334	2.35E-11
Nup50	3.57E-09	0.954801789	0.288	0.189	0.000198
Ftl1	5.09E-31	0.953250432	0.814	0.735	2.82E-26
Mllt6	7.33E-07	0.949393943	0.213	0.129	0.040602
Wipi2	6.14E-08	0.947129635	0.238	0.163	0.003402
Ece1	8.86E-10	0.943626284	0.341	0.232	4.91E-05
Rela	4.63E-12	0.942712131	0.379	0.245	2.56E-07
Hivep2	1.69E-07	0.935876703	0.278	0.187	0.009348
Arih2	1.02E-07	0.927680346	0.232	0.134	0.005638
Brd4	9.79E-21	0.924350237	0.565	0.405	5.42E-16
Stat3	3.42E-20	0.921924991	0.587	0.406	1.89E-15
Runx1	1.81E-21	0.920617819	0.647	0.488	1.01E-16
Ralgds	3.62E-07	0.917892077	0.235	0.148	0.020081

Tpp2	1.13E-09	0.917727193	0.322	0.203	6.24E-05
Lima1	1.19E-10	0.916655419	0.365	0.241	6.59E-06
Elf1	2.28E-12	0.910760587	0.424	0.277	1.27E-07
Aqp1	7.23E-17	0.908483721	0.541	0.363	4.01E-12
Dyrk1a	3.63E-08	0.906190254	0.265	0.158	0.002014
Slc38a2	1.11E-32	0.904794407	0.862	0.744	6.13E-28
Emp1	1.51E-23	0.904741433	0.749	0.598	8.36E-19
Bhlhe40	8.74E-24	0.903437004	0.684	0.508	4.84E-19
Ddx3x	1.37E-34	0.901278085	0.842	0.662	7.62E-30
Irs2	6.20E-11	0.897753816	0.363	0.24	3.43E-06
Kctd20	2.64E-07	0.89768626	0.259	0.172	0.01464
Map7d1	1.14E-21	0.896216936	0.614	0.48	6.31E-17
Tcf7	1.13E-08	0.894268222	0.262	0.201	0.000628
Smurf2	1.48E-12	0.893898654	0.414	0.265	8.20E-08
Rc3h1	2.53E-15	0.891466777	0.509	0.357	1.40E-10
Smg1	1.36E-07	0.887366164	0.261	0.16	0.007517
Rassf1	1.41E-11	0.885647781	0.413	0.298	7.84E-07
Foxo1	1.32E-07	0.878344638	0.29	0.202	0.007292
Irf2bpl	2.36E-15	0.873046092	0.527	0.386	1.31E-10
Runx3	3.99E-08	0.872780048	0.315	0.226	0.002211
Cpd	2.66E-07	0.870046116	0.259	0.169	0.014727
Phldb1	6.94E-09	0.8686917	0.336	0.23	0.000385
Atf4	5.87E-33	0.86624429	0.82	0.733	3.25E-28
Actn1	2.07E-20	0.865675269	0.611	0.438	1.15E-15
Sash1	1.13E-09	0.864131433	0.352	0.246	6.26E-05
Elk4	7.02E-07	0.861733555	0.252	0.16	0.038904
Epha2	4.91E-08	0.860729222	0.339	0.233	0.002721
Mlf1	3.06E-12	0.857448656	0.438	0.286	1.69E-07
Azin1	1.94E-26	0.856907779	0.755	0.608	1.08E-21
Esr1	8.89E-11	0.849020958	0.374	0.236	4.93E-06
Zfp655	7.11E-07	0.84805122	0.218	0.125	0.039401
Flna	1.00E-16	0.846049436	0.555	0.388	5.57E-12
H2-Q4	2.68E-13	0.838532246	0.471	0.33	1.48E-08
Skil	1.69E-21	0.836149727	0.688	0.499	9.37E-17
Ywhag	1.63E-07	0.835009559	0.267	0.192	0.009051
Map3k2	1.76E-08	0.831890369	0.337	0.23	0.000975
Sbno2	2.47E-07	0.829795992	0.282	0.182	0.013696
Gna13	4.04E-11	0.827229246	0.419	0.301	2.24E-06
Dmp1	6.98E-07	0.827052436	0.459	0.478	0.038684
Ddit3	4.35E-07	0.822492605	0.342	0.24	0.024124
Klf13	3.82E-22	0.82146762	0.697	0.557	2.12E-17
Heg1	2.32E-09	0.820467786	0.378	0.255	0.000129
Arfgef1	2.07E-08	0.818342884	0.346	0.243	0.001148
Utrn	7.93E-13	0.818174988	0.5	0.375	4.39E-08
Crebbp	1.81E-15	0.813295173	0.539	0.369	1.00E-10
Adamts1	1.18E-10	0.811524333	0.568	0.441	6.55E-06
Hdac5	1.31E-08	0.809546222	0.335	0.251	0.000724

Kdm7a	6.44E-13	0.809262248	0.49	0.348	3.57E-08
Chd1	5.52E-10	0.80784285	0.399	0.291	3.06E-05
Vim	8.02E-25	0.803925542	0.929	0.937	4.44E-20
Mef2d	4.71E-08	0.803647616	0.312	0.227	0.002612
Serpine2	7.11E-36	0.803617518	0.986	0.948	3.94E-31
Dusp1	1.45E-14	0.803202472	0.739	0.671	8.02E-10
5430416N0	2.07E-10	0.803050464	0.423	0.296	1.14E-05
Srsf5	3.07E-25	0.799840033	0.765	0.663	1.70E-20
Ptpn14	4.61E-07	0.79817867	0.289	0.207	0.025524
Ptbp1	8.75E-21	0.796726951	0.697	0.578	4.85E-16
Siah2	6.77E-08	0.796392967	0.328	0.223	0.003751
Scpep1	3.41E-10	0.791183578	0.393	0.257	1.89E-05
Ywhaz	3.69E-25	0.788887032	0.746	0.61	2.05E-20
Glul	4.17E-08	0.784565436	0.378	0.272	0.002309
Hipk1	8.61E-17	0.778823487	0.56	0.371	4.77E-12
Klf4	3.49E-27	0.778629	0.91	0.836	1.93E-22
Nfkb2	6.79E-07	0.772795037	0.292	0.193	0.03763
Samd4	2.93E-09	0.770558809	0.419	0.31	0.000162
Emilin1	4.10E-16	0.755541238	0.611	0.462	2.27E-11
Septin9	2.71E-07	0.75433349	0.331	0.233	0.015
Mmp13	7.60E-43	0.753480492	0.862	0.585	4.21E-38
Spata13	4.26E-07	0.749826765	0.303	0.224	0.023613
Spon1	2.20E-08	0.74423424	0.346	0.224	0.001221
Atf6	3.62E-08	0.742682997	0.374	0.275	0.002004
Angpt4	2.38E-16	0.741690937	0.649	0.475	1.32E-11
Mafg	4.21E-07	0.740777933	0.326	0.227	0.023321
Med13l	2.13E-14	0.737879542	0.565	0.455	1.18E-09
Etf1	8.67E-12	0.736520678	0.509	0.397	4.80E-07
Hnrnph1	4.67E-18	0.734339119	0.688	0.547	2.59E-13
Sf1	8.63E-10	0.733636023	0.417	0.29	4.78E-05
Mdk	2.62E-09	0.732259474	0.397	0.262	0.000145
Eif4g1	7.81E-11	0.728305663	0.449	0.351	4.32E-06
Tuba1c	4.22E-14	0.725067291	0.645	0.546	2.34E-09
Fam20c	5.05E-09	0.722051177	0.536	0.435	0.00028
Tcp11l2	1.22E-11	0.720746928	0.468	0.388	6.78E-07
Arih1	2.17E-07	0.718999106	0.359	0.261	0.012032
Col22a1	2.44E-17	0.718141028	0.657	0.57	1.35E-12
Ttc28	1.51E-07	0.717756429	0.359	0.275	0.008373
Sh3pxd2a	5.65E-13	0.71478154	0.532	0.424	3.13E-08
Brd2	5.38E-22	0.714462677	0.837	0.712	2.98E-17
Pim3	9.29E-10	0.713626228	0.508	0.397	5.14E-05
Odc1	9.71E-08	0.713209898	0.417	0.32	0.00538
Ski	1.54E-10	0.710050044	0.474	0.357	8.54E-06
Kcnq1ot1	6.02E-09	0.709869238	0.477	0.347	0.000333
Mrc2	1.09E-16	0.708652732	0.675	0.527	6.06E-12
Ptbp3	2.85E-07	0.700014528	0.363	0.288	0.015773
Tbc1d15	1.99E-09	0.698623881	0.414	0.333	0.00011

Arhgef12	9.74E-08	0.69766907	0.373	0.265	0.005396
Cstb	3.08E-13	0.696696932	0.669	0.618	1.71E-08
Fth1	5.21E-28	0.696560026	0.999	1	2.89E-23
Wwtr1	1.29E-12	0.69643265	0.543	0.467	7.13E-08
Gpc1	6.50E-27	0.690875836	0.846	0.784	3.60E-22
Kdm5a	6.40E-11	0.690045805	0.467	0.382	3.55E-06
Pum2	2.82E-07	0.689436006	0.38	0.29	0.015619
Tnc	8.16E-34	0.687959117	0.986	0.941	4.52E-29
Rai14	9.14E-08	0.687888699	0.391	0.277	0.005062
Tns3	5.83E-13	0.686697896	0.634	0.512	3.23E-08
Cdkn1a	1.15E-20	0.686016366	0.871	0.821	6.35E-16
Ncl	3.46E-30	0.685139632	0.947	0.889	1.92E-25
Trip12	9.97E-08	0.682384947	0.389	0.284	0.005525
Tor1aip1	1.64E-08	0.682129737	0.408	0.314	0.000907
Bmpr2	1.75E-10	0.677639441	0.522	0.391	9.68E-06
Rora	7.44E-08	0.674904475	0.41	0.328	0.004125
Pxdc1	2.74E-07	0.665691317	0.431	0.339	0.015165
Ccnd1	3.96E-10	0.665452656	0.62	0.512	2.19E-05
Dpysl2	1.44E-08	0.665094847	0.422	0.337	0.000795
Rps28	1.86E-28	0.664605662	0.872	0.792	1.03E-23
Cltc	1.11E-12	0.660819721	0.558	0.46	6.16E-08
Ranbp2	6.84E-08	0.65934507	0.417	0.303	0.003792
Wac	6.69E-08	0.658981336	0.397	0.311	0.003707
Nudt4	9.65E-16	0.658002329	0.765	0.637	5.35E-11
Map4k4	1.37E-14	0.65596107	0.692	0.559	7.62E-10
Desi2	5.05E-08	0.645445984	0.381	0.305	0.002798
Gas5	9.42E-34	0.644216374	0.953	0.943	5.22E-29
Rn18s	2.68E-23	0.642181628	1	1	1.48E-18
Chka	2.02E-07	0.641601276	0.393	0.334	0.011207
Anxa2	4.73E-11	0.634341203	0.701	0.637	2.62E-06
Gm10076	2.37E-20	0.632527458	0.779	0.681	1.32E-15
Tm4sf1	7.58E-12	0.631917863	0.728	0.726	4.20E-07
Tiprl	6.46E-07	0.631720593	0.398	0.319	0.035794
Cxcl12	3.55E-10	0.630866885	0.471	0.322	1.97E-05
Sgk1	2.78E-09	0.630141671	0.528	0.387	0.000154
Lpp	7.52E-10	0.625424316	0.542	0.44	4.17E-05
Ctdsp2	5.54E-08	0.623448	0.455	0.339	0.003067
Zfp36	4.29E-15	0.617025068	0.866	0.811	2.38E-10
Tapbp	8.48E-10	0.611193416	0.551	0.448	4.70E-05
Pfdn2	1.30E-10	0.608597258	0.533	0.456	7.22E-06
Insig1	4.79E-07	0.606713067	0.423	0.337	0.026522
Lrrc58	1.24E-09	0.606026583	0.569	0.453	6.89E-05
Ier5	4.03E-11	0.601152998	0.718	0.654	2.23E-06
Clic4	8.49E-07	0.599996197	0.442	0.348	0.047051
1110038B1	1.80E-12	0.599397366	0.632	0.58	9.95E-08
Ptpn4	3.50E-07	0.598623513	0.369	0.325	0.01937
Qsox1	2.00E-07	0.597458834	0.496	0.396	0.011093

Nfe2l2	8.92E-08	0.589994575	0.511	0.411	0.004944
Ddx21	1.91E-07	0.58937166	0.499	0.397	0.010601
Lrp1	1.03E-12	0.589240622	0.688	0.609	5.70E-08
Fus	4.05E-12	0.583368979	0.654	0.549	2.24E-07
Adamts5	2.40E-11	0.582875493	0.739	0.647	1.33E-06
Myo1b	5.16E-07	0.581464927	0.471	0.378	0.028607
Mbnl1	9.92E-08	0.581405082	0.495	0.386	0.005496
Nedd9	3.53E-08	0.579255193	0.566	0.454	0.001953
Cyth2	7.99E-07	0.575889281	0.397	0.332	0.044252
Lrp4	1.02E-11	0.575778292	0.769	0.675	5.66E-07
Sec24d	2.81E-09	0.574971329	0.37	0.327	0.000156
Tiparp	2.08E-08	0.572379123	0.612	0.523	0.001153
Igsf3	4.91E-07	0.57078793	0.489	0.395	0.0272
Colec12	6.39E-23	0.568440656	0.923	0.839	3.54E-18
Glrx5	1.30E-09	0.565614148	0.526	0.489	7.20E-05
Sema5a	6.16E-09	0.563327927	0.603	0.527	0.000341
Wdr26	1.48E-09	0.552571944	0.497	0.454	8.19E-05
Ubr5	2.08E-07	0.550050713	0.453	0.332	0.011535
Rpl7	6.30E-39	0.54230963	0.977	0.961	3.49E-34
Efnb1	7.14E-09	0.540367927	0.51	0.46	0.000395
Hspd1	8.46E-09	0.539358029	0.581	0.497	0.000469
Pabpc1	1.60E-23	0.538694175	0.919	0.839	8.89E-19
Prrc2c	4.33E-11	0.538077295	0.722	0.651	2.40E-06
Golph3	4.13E-08	0.531550775	0.554	0.451	0.00229
Ecm1	8.27E-09	0.530774068	0.448	0.31	0.000458
Btg1	1.37E-10	0.530408817	0.723	0.622	7.57E-06
Mcl1	9.59E-15	0.528685826	0.842	0.763	5.31E-10
Zfhx4	7.93E-08	0.527975442	0.593	0.496	0.004394
Vcam1	3.90E-10	0.523191348	0.484	0.334	2.16E-05
Klf9	8.98E-14	0.522820978	0.831	0.725	4.98E-09
Tubb4b	1.21E-07	0.515055645	0.575	0.536	0.006693
Hsp90ab1	3.52E-38	0.512673978	0.991	0.995	1.95E-33
Ddx6	1.14E-08	0.511046414	0.607	0.527	0.000629
Gadd45g	5.52E-11	0.509520303	0.905	0.9	3.06E-06
Med10	3.00E-11	0.504223472	0.385	0.393	1.66E-06
Zfand5	2.08E-14	0.502048491	0.879	0.808	1.15E-09
Ubc	6.48E-19	0.498934759	0.952	0.943	3.59E-14
Rps2	3.51E-41	0.498369109	0.999	1	1.95E-36
Ncam1	1.62E-09	0.49493668	0.776	0.7	8.96E-05

OsteoCAR_UP					
Gene	p_val	avg_log2FC	pct.1	pct.2	p_val_adj
Mndal	6.47E-08	2.099705211	0.16	0.048	0.003585
Clip2	2.87E-07	1.334596537	0.27	0.133	0.01592
Ccnd1	1.00E-08	1.307563212	0.426	0.258	0.000554
C4b	1.58E-18	1.2776587	0.69	0.456	8.74E-14
Aebp1	1.34E-07	1.200216988	0.365	0.208	0.007438
C1s1	8.33E-15	1.174573981	0.587	0.376	4.62E-10
Chst15	2.78E-07	1.166935518	0.31	0.158	0.015391
Ltbp2	2.81E-09	1.116073052	0.501	0.343	0.000156
Neat1	2.20E-08	1.109082137	0.662	0.564	0.001217
Mir6236	1.48E-24	1.095966855	0.985	0.937	8.19E-20
Thsd4	1.93E-07	1.03550952	0.371	0.203	0.010706
Nr4a1	1.46E-08	0.957106574	0.552	0.414	0.000808
Fosb	2.29E-15	0.940776064	0.842	0.742	1.27E-10
Ecm1	2.14E-14	0.939149748	0.758	0.591	1.19E-09
Lmna	2.04E-07	0.84912679	0.602	0.491	0.011289
Mgp	4.38E-07	0.82384655	0.402	0.534	0.024269
Nrp1	5.18E-08	0.823677134	0.607	0.461	0.002869
Fus	1.96E-09	0.821676617	0.574	0.424	0.000109
Adgrf5	4.51E-07	0.753662775	0.532	0.416	0.024987
Emp1	1.33E-07	0.734392807	0.646	0.539	0.007385
Epas1	6.95E-07	0.722876961	0.631	0.504	0.038525
Myh9	3.68E-07	0.707401096	0.618	0.456	0.020414
Cxcl14	2.00E-08	0.654922968	0.787	0.769	0.001106
Canx	1.23E-08	0.62897253	0.756	0.639	0.000683
Rn18s	8.97E-12	0.620889031	1	1	4.97E-07
Serpine2	1.21E-16	0.612531312	1	0.997	6.71E-12
Lrp1	1.83E-07	0.57460678	0.782	0.674	0.010119
Mylk	5.06E-07	0.563495334	0.738	0.589	0.028059
Tnc	1.77E-26	0.552771111	1	0.997	9.81E-22
S100a6	1.04E-07	0.466266303	0.875	0.875	0.005783
Calr	7.72E-10	0.420099087	0.954	0.917	4.28E-05
Hsp90b1	5.99E-08	0.334745379	0.991	0.972	0.003316

AdipoCAR_UP					
Gene	p_val	avg_log2FC	pct.1	pct.2	p_val_adj
Ly6a	5.83E-11	2.533359	0.108	0.027	3.23E-06
Cxcl5	4.98E-14	2.242931	0.166	0.052	2.76E-09
Fndc1	2.83E-33	1.982088	0.386	0.136	1.57E-28
Scd1	2.00E-11	1.699546	0.167	0.06	1.11E-06
Ldlr	6.43E-10	1.633079	0.111	0.031	3.56E-05
Serpine1	2.92E-11	1.606283	0.218	0.105	1.62E-06
Arrdc3	2.58E-07	1.541021	0.11	0.039	0.01427
Adamdec1	2.55E-11	1.493253	0.172	0.063	1.42E-06
Tmcc3	4.86E-08	1.434247	0.142	0.061	0.002693
Nlrc5	4.64E-11	1.315193	0.178	0.068	2.57E-06
Tnfaip3	4.40E-10	1.314144	0.234	0.12	2.44E-05
Adgra2	2.93E-08	1.235588	0.182	0.09	0.001624
Sbno2	2.88E-10	1.20679	0.245	0.129	1.59E-05
Mrap	1.56E-11	1.205729	0.217	0.093	8.62E-07
Axl	2.22E-08	1.179646	0.172	0.076	0.001228
Cxcl13	8.98E-11	1.17915	0.372	0.23	4.97E-06
Thbs1	2.51E-16	1.12942	0.588	0.423	1.39E-11
Cxcl9	5.95E-11	1.126306	0.556	0.415	3.30E-06
Mir6236	1.19E-54	1.116665	0.987	0.928	6.62E-50
Ptx3	2.07E-18	1.110913	0.651	0.472	1.15E-13
Dcn	3.06E-19	1.071728	0.793	0.66	1.69E-14
Aebp1	2.68E-19	1.067289	0.47	0.265	1.49E-14
Nr4a1	2.86E-12	1.036312	0.432	0.29	1.58E-07
Vps13d	2.33E-07	1.034488	0.177	0.086	0.012936
P3h2	1.15E-10	1.01465	0.266	0.133	6.39E-06
Septin9	2.60E-07	1.013485	0.204	0.107	0.014383
Mafg	2.31E-07	0.987298	0.229	0.129	0.01282
Il17ra	9.57E-08	0.986399	0.236	0.132	0.005305
Ptgfrn	1.51E-10	0.972134	0.315	0.178	8.35E-06
Thbs2	2.65E-14	0.95673	0.494	0.333	1.47E-09
Gdf10	3.43E-10	0.947623	0.332	0.195	1.90E-05
Atp2b4	4.65E-07	0.946426	0.21	0.113	0.025783
Mc5r	5.45E-07	0.934534	0.223	0.124	0.030209
Adamts5	8.87E-19	0.919824	0.668	0.487	4.91E-14
Dhrs3	1.49E-16	0.889406	0.527	0.336	8.26E-12
Igf1r	2.80E-09	0.875855	0.303	0.173	0.000155
Lmna	2.41E-13	0.873992	0.549	0.389	1.33E-08
Jak2	4.41E-09	0.855205	0.277	0.152	0.000244
Ppp1r15b	1.87E-08	0.854084	0.278	0.158	0.001038
Cxcl14	1.89E-75	0.842897	0.999	0.982	1.05E-70
Hk2	4.59E-07	0.841578	0.32	0.218	0.025443
Thsd4	5.78E-13	0.826796	0.5	0.331	3.20E-08
Cyth3	5.82E-08	0.824981	0.277	0.161	0.003222
Sorbs2	2.12E-13	0.824447	0.428	0.252	1.17E-08
Gcnt2	6.42E-07	0.822026	0.195	0.103	0.03558

Gbp2	1.89E-09	0.819154	0.409	0.265	0.000105
Mrc2	2.70E-09	0.817255	0.336	0.201	0.00015
B4galt1	4.53E-07	0.816273	0.245	0.14	0.025102
Ly6e	2.57E-22	0.807677	0.739	0.54	1.42E-17
Serpina3f	1.44E-11	0.794556	0.503	0.343	8.00E-07
Golga3	2.44E-07	0.783474	0.172	0.087	0.013525
C4b	1.38E-38	0.763356	0.94	0.822	7.67E-34
Gbp3	9.40E-08	0.760853	0.302	0.182	0.00521
Sdk2	2.15E-08	0.759241	0.288	0.166	0.001192
Uba1	1.61E-07	0.758619	0.313	0.196	0.008944
Emp1	9.15E-22	0.758007	0.772	0.593	5.07E-17
Sdc4	6.24E-16	0.754438	0.64	0.457	3.46E-11
Cxcl16	3.25E-07	0.74426	0.274	0.163	0.018026
Tap1	1.62E-07	0.731666	0.352	0.233	0.00899
Emilin1	2.82E-11	0.708915	0.463	0.299	1.56E-06
Flna	9.68E-15	0.700624	0.557	0.363	5.36E-10
Errfi1	1.62E-16	0.697137	0.814	0.68	8.97E-12
Myl9	3.51E-12	0.68954	0.473	0.299	1.94E-07
Rbp1	1.38E-13	0.67972	0.57	0.385	7.66E-09
Amotl2	2.30E-11	0.67771	0.563	0.407	1.28E-06
Eif4g1	1.05E-09	0.667898	0.397	0.249	5.80E-05
Ak1	4.29E-07	0.663043	0.313	0.197	0.023752
Atp13a3	1.80E-09	0.649478	0.512	0.38	9.97E-05
Mgp	1.28E-07	0.646359	0.908	0.863	0.007077
Skil	3.36E-07	0.645795	0.366	0.244	0.018622
Stat3	8.56E-10	0.634602	0.54	0.4	4.74E-05
Frmd6	2.82E-11	0.633438	0.554	0.39	1.56E-06
Sdc3	3.74E-07	0.632904	0.383	0.26	0.020704
Phex	1.10E-13	0.632103	0.566	0.378	6.11E-09
P4ha1	1.71E-07	0.629995	0.289	0.176	0.009475
Vcl	7.46E-07	0.628238	0.393	0.279	0.041311
C1s1	9.71E-28	0.62519	0.891	0.703	5.38E-23
Fosb	1.76E-17	0.615687	0.841	0.687	9.75E-13
Kmt2a	1.29E-07	0.603731	0.42	0.29	0.007125
Sfrp1	1.44E-10	0.59827	0.637	0.501	7.98E-06
Itih5	1.57E-07	0.590318	0.498	0.374	0.008684
Gm10076	7.77E-07	0.589585	0.436	0.329	0.043061
Tgfbr3	1.61E-17	0.589002	0.802	0.667	8.94E-13
Cbln1	6.78E-07	0.585642	0.234	0.137	0.037552
Tapbp	4.71E-15	0.581632	0.712	0.531	2.61E-10
Serpina12	9.04E-08	0.578637	0.624	0.506	0.005011
Neat1	8.90E-08	0.575407	0.684	0.573	0.004933
Atp1a1	3.94E-11	0.571426	0.619	0.457	2.19E-06
S100a6	7.08E-14	0.570551	0.836	0.71	3.92E-09
Ehd1	6.89E-09	0.562322	0.379	0.242	0.000382
S1pr1	5.92E-08	0.557342	0.447	0.31	0.003279
C3	4.88E-13	0.557082	0.659	0.479	2.70E-08

Nampt	2.08E-07	0.556417	0.39	0.263	0.011544
Gpm6b	6.90E-07	0.556248	0.445	0.321	0.038242
Tnc	1.45E-17	0.551745	0.919	0.796	8.05E-13
Usp7	8.02E-07	0.537777	0.235	0.143	0.044428
Ddx3x	1.25E-11	0.536386	0.719	0.597	6.94E-07
Rn18s	4.31E-23	0.528428	1	1	2.39E-18
Clic4	3.91E-07	0.521588	0.549	0.43	0.021661
Vit	1.44E-08	0.520282	0.502	0.356	0.000799
Cavin1	1.98E-08	0.517256	0.554	0.412	0.001097
Camk2n1	3.03E-07	0.514763	0.495	0.365	0.016793
Actn1	5.72E-07	0.487111	0.466	0.337	0.03167
Picalm	3.71E-10	0.477037	0.616	0.456	2.06E-05
Lox	4.84E-11	0.468571	0.813	0.701	2.68E-06
Slit3	6.57E-09	0.466096	0.395	0.26	0.000364
Runx1	7.47E-13	0.455281	0.86	0.751	4.14E-08
Spon1	2.34E-08	0.455261	0.669	0.543	0.001299
S1pr3	9.15E-08	0.442925	0.692	0.581	0.005067
Svep1	6.48E-07	0.436756	0.504	0.374	0.035906
Nfib	3.96E-07	0.425965	0.643	0.524	0.021936
Actb	2.62E-25	0.425305	0.997	0.989	1.45E-20
Serpine2	3.96E-21	0.418545	0.997	0.986	2.20E-16
Lrp1	5.34E-10	0.417677	0.75	0.608	2.96E-05
Fus	7.55E-09	0.413636	0.573	0.423	0.000419
Psme1	8.80E-07	0.403328	0.682	0.577	0.048745
Nudt4	3.50E-10	0.402975	0.844	0.748	1.94E-05
Fndc3b	5.92E-07	0.402073	0.685	0.569	0.032772
Limch1	9.13E-08	0.400152	0.647	0.51	0.00506
Lrrc58	5.25E-07	0.398196	0.597	0.467	0.029064
Zcchc24	1.32E-07	0.395175	0.479	0.346	0.007289
Mylk	1.81E-08	0.382577	0.791	0.693	0.001002
Igfbp5	2.46E-12	0.365194	0.994	0.986	1.36E-07
Calr	1.26E-14	0.361926	0.96	0.914	6.98E-10
Zfhx4	2.27E-07	0.356125	0.641	0.507	0.012572
Tpm4	2.05E-07	0.349455	0.584	0.449	0.011382
Pdzrn4	7.21E-07	0.339671	0.589	0.459	0.039942
Ltbp2	1.45E-07	0.338919	0.766	0.761	0.008028
Epas1	3.40E-11	0.329767	0.942	0.865	1.88E-06
Gpx3	1.82E-09	0.318619	0.987	0.973	0.000101
Tnfrsf19	6.08E-08	0.314846	0.592	0.453	0.003366
Cp	1.64E-08	0.297116	0.891	0.799	0.000908
Kng2	2.24E-08	0.286372	0.919	0.845	0.001241
Cfh	1.21E-08	0.260884	0.955	0.897	0.000668
Cdh11	1.39E-08	0.254792	0.966	0.917	0.000773
Lars2	3.48E-09	0.251826	0.668	0.521	0.000193
Cst3	4.45E-07	0.239036	1	0.999	0.024649
Cxcl12	2.09E-18	0.235838	1	1	1.16E-13
B2m	6.66E-13	0.215575	0.999	0.997	3.69E-08

Fstl1	1.98E-07	0.215326	0.977	0.936	0.010968

Osteoblasts_DOWN					
Gene	p_val	avg_log2FC	pct.1	pct.2	p_val_adj
Snorc	1.87E-81	-8.103658726	0	0.182	1.04E-76
Mgp	3.35E-120	-2.84807531	0.137	0.539	1.86E-115
Chad	2.79E-26	-2.74174804	0.017	0.112	1.55E-21
Gm36827	5.56E-15	-1.82559138	0.04	0.118	3.08E-10
Pcsk6	3.48E-25	-1.699677551	0.068	0.199	1.93E-20
Plppr5	2.03E-19	-1.670217506	0.045	0.144	1.12E-14
Proser2	9.28E-26	-1.657560523	0.07	0.204	5.14E-21
7SK.293	1.11E-11	-1.62881385	0.123	0.214	6.15E-07
Alcam	1.12E-21	-1.544016105	0.07	0.19	6.22E-17
Eid2	7.92E-21	-1.542912935	0.065	0.181	4.39E-16
Gm40193	4.27E-18	-1.535301849	0.07	0.177	2.37E-13
Srgn	1.25E-34	-1.472707612	0.132	0.322	6.93E-30
Pi15	7.23E-13	-1.420706954	0.084	0.17	4.01E-08
Atp6v0e2	9.03E-13	-1.378536273	0.041	0.114	5.00E-08
Wnt4	1.70E-66	-1.332846627	0.427	0.692	9.44E-62
Scrn1	4.20E-08	-1.279727553	0.046	0.1	0.002327
Mt2	1.69E-15	-1.208298928	0.171	0.289	9.39E-11
Inhbb	2.25E-08	-1.205331126	0.048	0.106	0.001249
Fez1	2.88E-18	-1.190464103	0.092	0.21	1.59E-13
Ppp2cb	5.55E-25	-1.189921286	0.161	0.32	3.08E-20
Cdk15	8.67E-09	-1.157678341	0.065	0.13	0.00048
Gng12	7.60E-14	-1.133411289	0.087	0.185	4.21E-09
Shld1	3.88E-09	-1.109772786	0.063	0.13	0.000215
Wtip	1.00E-24	-1.105150882	0.172	0.338	5.55E-20
Mbd2	3.42E-98	-1.088051503	0.656	0.859	1.90E-93
Anxa3	1.84E-13	-1.082664888	0.127	0.228	1.02E-08
Loxl2	4.10E-57	-1.078278217	0.209	0.49	2.27E-52
Prss35	1.06E-93	-1.074854293	0.492	0.831	5.85E-89
Homer2	9.95E-18	-1.068725819	0.132	0.259	5.52E-13
Hnrnpa0	2.42E-73	-1.048198705	0.588	0.8	1.34E-68
Bglap2	1.07E-125	-1.033092446	0.964	0.999	5.94E-121
Bglap	3.54E-128	-1.015111281	0.98	1	1.96E-123
Ybx3	4.03E-35	-1.003796393	0.276	0.496	2.23E-30
Rsrp1	2.51E-50	-0.983040953	0.573	0.782	1.39E-45
Lipc	1.07E-100	-0.965492149	0.741	0.92	5.94E-96
Slc9a2	1.97E-13	-0.961617138	0.101	0.202	1.09E-08
Chn1	6.96E-49	-0.952655157	0.47	0.697	3.85E-44
Col3a1	2.44E-09	-0.951327387	0.046	0.108	0.000135
BC055402	6.16E-15	-0.942308946	0.144	0.264	3.41E-10
Tcea3	9.85E-19	-0.922307624	0.19	0.336	5.46E-14
Omd	1.47E-15	-0.883979697	0.315	0.443	8.17E-11
Pla2g5	3.73E-14	-0.879272265	0.221	0.335	2.06E-09
Cacna2d3	1.29E-13	-0.866872909	0.18	0.293	7.17E-09
1700066M	1.94E-12	-0.865266758	0.122	0.224	1.08E-07
Cdk2ap1	4.05E-31	-0.853917976	0.379	0.573	2.24E-26

Alg3	1.82E-08	-0.849636436	0.117	0.193	0.001008
Selenbp1	7.20E-15	-0.83389722	0.188	0.315	3.99E-10
Trmt61a	4.21E-08	-0.833534518	0.11	0.185	0.002331
Cela1	1.21E-07	-0.831894348	0.102	0.172	0.006709
Ing1	9.65E-17	-0.816027958	0.223	0.363	5.35E-12
C1galt1	4.88E-14	-0.813755969	0.222	0.341	2.70E-09
Cd1d1	8.02E-44	-0.806575128	0.587	0.76	4.44E-39
H1f10	1.53E-14	-0.798055325	0.171	0.295	8.46E-10
Osr1	2.65E-09	-0.797878246	0.082	0.156	0.000147
Ryk	3.69E-46	-0.788235807	0.594	0.772	2.05E-41
Cyhr1	1.23E-08	-0.785815928	0.115	0.196	0.000684
Cpz	1.80E-95	-0.781984729	0.881	0.968	9.97E-91
Mpp6	1.50E-10	-0.779102706	0.175	0.275	8.33E-06
Zfp637	8.12E-17	-0.774065748	0.249	0.391	4.50E-12
Dcn	1.55E-161	-0.770476769	0.988	1	8.60E-157
Chpt1	2.17E-08	-0.759365562	0.145	0.227	0.001203
Ecrg4	1.44E-07	-0.748337194	0.126	0.203	0.007973
Smagp	1.61E-22	-0.743124625	0.378	0.546	8.92E-18
Ubtd1	7.65E-08	-0.74129275	0.147	0.225	0.00424
Dync1i1	6.98E-12	-0.735442255	0.164	0.273	3.87E-07
Mpped2	8.70E-08	-0.730415795	0.113	0.189	0.004819
Mageh1	4.67E-22	-0.711103663	0.363	0.534	2.59E-17
Car3	3.01E-108	-0.710899154	1	1	1.67E-103
Terf2ip	6.99E-07	-0.710733007	0.14	0.211	0.038713
AU021092	2.44E-20	-0.710376684	0.49	0.611	1.35E-15
Fam136a	5.17E-11	-0.7070179	0.164	0.268	2.86E-06
Cmc4	1.39E-09	-0.699907925	0.148	0.242	7.68E-05
Lum	3.06E-39	-0.697910112	0.722	0.869	1.70E-34
Bambi	1.85E-42	-0.69620878	0.834	0.932	1.03E-37
Osbpl1a	7.66E-09	-0.695778638	0.124	0.208	0.000425
Sirt1	1.59E-08	-0.694792105	0.17	0.259	0.000882
Fibin	3.45E-18	-0.690471593	0.367	0.516	1.91E-13
Mamdc2	2.82E-07	-0.684180075	0.123	0.198	0.015608
Cbr4	3.92E-07	-0.682100306	0.082	0.143	0.021711
Col2a1	3.63E-64	-0.680363391	0.824	0.972	2.01E-59
Cpq	1.29E-49	-0.677165962	0.743	0.869	7.17E-45
Grrp1	3.13E-18	-0.661208461	0.413	0.559	1.73E-13
Filip1l	4.43E-21	-0.653706164	0.469	0.629	2.45E-16
Bcl7b	8.54E-12	-0.651684808	0.228	0.347	4.73E-07
Sertad3	1.60E-09	-0.651220798	0.177	0.275	8.88E-05
Crls1	3.07E-09	-0.650671347	0.206	0.303	0.00017
Ngrn	1.17E-07	-0.649805787	0.146	0.227	0.006503
Tsc22d1	1.72E-46	-0.638119788	0.951	0.991	9.52E-42
Bfsp1	3.91E-08	-0.631677587	0.156	0.243	0.002168
0610009B2	2.12E-12	-0.628086033	0.3	0.419	1.17E-07
Me1	2.65E-09	-0.627223574	0.206	0.306	0.000147
Fbn2	1.47E-25	-0.623983113	0.288	0.477	8.14E-21

Snx21	1.26E-08	-0.620074711	0.204	0.299	0.000697
Il12a	5.31E-13	-0.618714925	0.224	0.35	2.94E-08
Mplkip	5.00E-12	-0.618492412	0.276	0.398	2.77E-07
Dlx5	5.11E-16	-0.616169797	0.393	0.539	2.83E-11
Ccdc28b	1.63E-07	-0.610582798	0.175	0.259	0.009049
Psip1	5.54E-25	-0.608565016	0.606	0.733	3.07E-20
Nbl1	2.83E-29	-0.60343054	0.695	0.805	1.57E-24
Bad	5.14E-12	-0.600754105	0.295	0.417	2.85E-07
Shox2	2.82E-33	-0.597349168	0.748	0.872	1.56E-28
Rflnb	4.97E-15	-0.597233417	0.407	0.529	2.75E-10
Mt1	4.77E-12	-0.597178973	0.517	0.639	2.64E-07
Evl	1.32E-13	-0.592828931	0.339	0.473	7.30E-09
Tbxas1	6.02E-07	-0.592011811	0.105	0.171	0.033342
Rpp25l	1.05E-09	-0.590022213	0.196	0.299	5.80E-05
Dmwd	1.95E-07	-0.585316005	0.192	0.278	0.010821
1810058I2	2.62E-34	-0.584501101	0.752	0.869	1.45E-29
Slc35a2	1.37E-19	-0.582279232	0.518	0.642	7.61E-15
Ppp1r11	9.61E-12	-0.57830296	0.314	0.436	5.32E-07
Col11a1	2.76E-59	-0.569747467	0.978	0.999	1.53E-54
Tmem60	1.25E-08	-0.56072143	0.22	0.319	0.000692
Txnip	1.55E-20	-0.560121228	0.53	0.691	8.61E-16
Anxa4	5.06E-08	-0.551977764	0.226	0.32	0.002806
Prkra	3.43E-08	-0.551904358	0.238	0.333	0.0019
Hras	2.42E-18	-0.55136334	0.56	0.677	1.34E-13
Rbm7	8.40E-13	-0.547070251	0.399	0.52	4.65E-08
Hoxa10	6.41E-07	-0.545889054	0.207	0.291	0.035506
Dnajc30	5.69E-07	-0.543273131	0.199	0.283	0.031532
Ube2m	9.09E-30	-0.539475034	0.742	0.838	5.04E-25
Sigmar1	7.53E-07	-0.536730821	0.113	0.179	0.041746
Cdc26	1.38E-13	-0.534983941	0.425	0.545	7.63E-09
Triap1	5.70E-09	-0.534923616	0.23	0.332	0.000316
Tm2d3	1.20E-09	-0.534653859	0.325	0.432	6.66E-05
Pigyl	4.47E-11	-0.531439008	0.342	0.461	2.48E-06
Prorsd1	8.27E-18	-0.531301497	0.531	0.664	4.58E-13
Mgst3	2.65E-24	-0.529784994	0.738	0.842	1.47E-19
Ubn2	3.23E-09	-0.529437707	0.246	0.351	0.000179
Akt1	2.30E-13	-0.528534604	0.451	0.57	1.27E-08
Arpc5l	1.20E-16	-0.508389559	0.522	0.655	6.67E-12
Wwp1	8.48E-10	-0.495145924	0.339	0.451	4.70E-05
Alpl	3.89E-24	-0.49165384	0.891	0.949	2.15E-19
Gnai1	7.93E-31	-0.489977123	0.757	0.889	4.39E-26
Cd109	3.49E-07	-0.489594715	0.123	0.193	0.019329
Psmc2	9.17E-13	-0.488724816	0.476	0.587	5.08E-08
Col15a1	1.81E-12	-0.48603481	0.319	0.45	1.00E-07
Exoc7	6.58E-07	-0.484885015	0.238	0.326	0.036441
Gpr180	8.78E-18	-0.484009025	0.623	0.723	4.86E-13
Ccdc90b	5.17E-07	-0.482395116	0.256	0.345	0.028659

Gemin7	6.80E-11	-0.479913992	0.381	0.501	3.77E-06
Arpc1a	1.99E-18	-0.478979654	0.615	0.737	1.10E-13
Smim4	2.37E-13	-0.475539046	0.458	0.591	1.31E-08
Smad4	4.49E-08	-0.474239417	0.343	0.441	0.00249
Arfip2	1.16E-08	-0.471973241	0.363	0.456	0.000643
Dynll1	7.72E-43	-0.471972147	0.974	0.985	4.28E-38
Nnmt	2.74E-11	-0.470891288	0.511	0.617	1.52E-06
Vstm4	2.60E-10	-0.467971289	0.465	0.557	1.44E-05
Bbip1	2.79E-08	-0.459470377	0.283	0.386	0.001546
Cd9	1.04E-45	-0.458677516	0.979	0.986	5.74E-41
Ube2e3	2.21E-13	-0.458040048	0.536	0.646	1.22E-08
Nt5dc2	2.95E-16	-0.456267278	0.633	0.745	1.64E-11
Cfl2	4.15E-08	-0.455635487	0.296	0.397	0.002299
Mlip	4.05E-27	-0.454911302	0.814	0.878	2.25E-22
Atraid	4.78E-22	-0.453390368	0.744	0.838	2.65E-17
Ap1s2	6.84E-14	-0.453303667	0.581	0.687	3.79E-09
Ei24	2.46E-07	-0.449619147	0.356	0.432	0.013625
Exosc4	1.90E-09	-0.444742535	0.386	0.498	0.000105
Iah1	1.56E-09	-0.442453807	0.447	0.546	8.65E-05
Snapc5	1.57E-07	-0.4410679	0.335	0.432	0.00872
Mat2b	1.04E-08	-0.441027673	0.413	0.495	0.000577
BC031181	3.08E-17	-0.438082935	0.662	0.768	1.71E-12
Tprgl	5.97E-11	-0.436967905	0.519	0.606	3.31E-06
Sra1	2.89E-27	-0.436803936	0.858	0.919	1.60E-22
Sdf4	1.24E-41	-0.434485208	0.928	0.964	6.85E-37
Pltp	4.16E-20	-0.433740156	0.779	0.876	2.31E-15
Bag5	6.86E-09	-0.432337748	0.394	0.501	0.00038
Ech1	7.36E-13	-0.431886156	0.603	0.682	4.08E-08
Trappc3	2.63E-08	-0.430784426	0.402	0.491	0.00146
Cox19	8.17E-07	-0.430724918	0.326	0.415	0.045287
Rerg	3.64E-15	-0.428184262	0.651	0.747	2.02E-10
Hebp2	1.49E-07	-0.427755604	0.286	0.384	0.008254
Tmed4	4.62E-07	-0.427279121	0.278	0.371	0.025572
1700123O2	6.52E-07	-0.426232801	0.343	0.432	0.036103
Hadh	7.30E-15	-0.42304414	0.66	0.744	4.04E-10
Sdhaf1	6.96E-09	-0.421010124	0.292	0.399	0.000385
Tmem119	1.15E-19	-0.417253761	0.861	0.92	6.38E-15
Pard6g	3.57E-17	-0.416046697	0.744	0.826	1.98E-12
Thbs1	6.17E-16	-0.413584988	0.793	0.889	3.42E-11
Luc7l3	5.21E-11	-0.413381765	0.49	0.61	2.89E-06
Hpcal1	5.09E-07	-0.412900508	0.381	0.467	0.028214
Jkamp	2.53E-13	-0.412550244	0.623	0.716	1.40E-08
Coa3	1.21E-15	-0.409022473	0.675	0.778	6.69E-11
Fuca1	1.41E-12	-0.408491935	0.612	0.702	7.80E-08
Npnt	3.60E-11	-0.405664163	0.231	0.34	2.00E-06
Serpinb6a	4.66E-10	-0.404224795	0.504	0.613	2.58E-05
Podxl2	4.27E-10	-0.403174927	0.559	0.648	2.37E-05

Svbp	1.62E-15	-0.400819957	0.73	0.796	8.99E-11
Mrpl43	2.18E-12	-0.400168754	0.608	0.709	1.21E-07
Tmed2	1.65E-12	-0.396550991	0.642	0.718	9.12E-08
Ifitm5	2.70E-36	-0.395999315	0.987	0.993	1.50E-31
Fundc2	3.53E-15	-0.395317022	0.722	0.805	1.96E-10
Kctd12b	1.98E-10	-0.395181383	0.31	0.428	1.10E-05
Commd9	8.14E-07	-0.394877998	0.401	0.479	0.045074
Dynlt3	4.12E-08	-0.393967753	0.442	0.54	0.002281
Fam78b	2.85E-09	-0.393567199	0.534	0.63	0.000158
Aldh2	2.76E-20	-0.393201292	0.847	0.883	1.53E-15
Dleu2	5.49E-10	-0.392975548	0.435	0.554	3.04E-05
Tsg101	1.13E-08	-0.392523171	0.474	0.572	0.000627
Polr2j	2.11E-11	-0.391460785	0.573	0.681	1.17E-06
Fap	1.39E-37	-0.391333578	0.97	0.988	7.71E-33
Spag7	8.41E-07	-0.390816048	0.42	0.503	0.046588
Slc25a11	4.08E-08	-0.389581723	0.47	0.557	0.002258
Tmem165	2.24E-09	-0.3882177	0.501	0.604	0.000124
Glmp	6.45E-09	-0.384988659	0.511	0.602	0.000357
Dapk2	2.46E-24	-0.384591521	0.902	0.933	1.37E-19
Ccdc194	1.61E-07	-0.38274442	0.501	0.573	0.008906
Slc36a2	1.79E-25	-0.382207986	0.898	0.931	9.90E-21
Ppp1r14b	3.93E-16	-0.382102112	0.811	0.866	2.18E-11
Utp11	5.89E-07	-0.381253087	0.443	0.529	0.032616
Eif3f	5.86E-35	-0.381174557	0.959	0.98	3.24E-30
Fcgrt	1.81E-15	-0.379857733	0.778	0.828	1.00E-10
Hspb2	5.29E-12	-0.378683266	0.68	0.758	2.93E-07
Rab21	8.83E-08	-0.378252331	0.341	0.444	0.004893
Sf3b6	6.45E-09	-0.375251035	0.541	0.626	0.000357
Uqcc3	5.10E-08	-0.374152016	0.498	0.588	0.002827
Trappc4	5.10E-09	-0.373414272	0.531	0.62	0.000282
Cops6	1.18E-10	-0.371682525	0.616	0.706	6.54E-06
mt-Rnr2	1.08E-13	-0.370304017	1	1	5.98E-09
B3gat3	1.24E-10	-0.367475827	0.623	0.707	6.89E-06
Pigbos1	1.12E-08	-0.367451503	0.513	0.613	0.000621
Pink1	1.81E-07	-0.366285743	0.485	0.572	0.010014
Hexa	3.99E-11	-0.365559929	0.637	0.73	2.21E-06
Purb	3.03E-08	-0.36534155	0.564	0.609	0.001676
Eri3	2.13E-07	-0.365207432	0.493	0.575	0.011788
Cpe	3.45E-49	-0.363448091	0.984	0.995	1.91E-44
Arl6ip1	1.01E-07	-0.362418991	0.407	0.511	0.00562
Atp1b3	5.98E-19	-0.36074878	0.838	0.911	3.32E-14
Snx5	6.29E-07	-0.360176485	0.502	0.574	0.034856
Mlf2	8.41E-11	-0.358983532	0.647	0.718	4.66E-06
Pdgfd	2.88E-07	-0.357634539	0.417	0.516	0.015932
Srp19	7.68E-15	-0.356365858	0.798	0.862	4.25E-10
Sema3d	1.35E-13	-0.356111748	0.44	0.581	7.45E-09
Timm8b	3.27E-11	-0.356034263	0.67	0.747	1.81E-06

Sdf2	3.00E-09	-0.355556803	0.576	0.661	0.000166
Rnf187	6.97E-13	-0.353388652	0.737	0.812	3.86E-08
Yif1a	2.15E-14	-0.351210786	0.778	0.83	1.19E-09
Elof1	1.96E-07	-0.350672005	0.514	0.585	0.010886
Itm2b	3.17E-66	-0.347830142	0.998	0.999	1.76E-61
Chmp5	3.65E-10	-0.347585852	0.532	0.648	2.02E-05
Tecr	7.16E-14	-0.346098754	0.771	0.845	3.97E-09
Emc10	1.48E-11	-0.345443993	0.745	0.8	8.22E-07
Kdelr1	7.57E-29	-0.345316152	0.95	0.968	4.19E-24
Med28	3.03E-07	-0.341411668	0.529	0.606	0.016774
Cdo1	5.40E-09	-0.341076795	0.784	0.837	0.000299
Prdx4	2.21E-09	-0.340412958	0.791	0.835	0.000122
Psmd7	1.45E-12	-0.339411998	0.755	0.819	8.05E-08
Txndc17	2.05E-19	-0.339156932	0.866	0.918	1.13E-14
Txndc15	2.27E-07	-0.337788454	0.531	0.596	0.012596
Mcrip1	1.41E-12	-0.337677546	0.791	0.849	7.81E-08
Aga	2.18E-09	-0.336619912	0.599	0.696	0.000121
Glt8d1	6.42E-07	-0.335963619	0.528	0.599	0.035567
Trappc2l	2.56E-10	-0.335028746	0.664	0.739	1.42E-05
Bet1	3.91E-09	-0.33330955	0.637	0.716	0.000217
Tmem126a	1.50E-07	-0.332621779	0.542	0.593	0.008288
Snx17	3.29E-08	-0.331679847	0.54	0.639	0.001821
Cacnb3	1.19E-08	-0.326984294	0.661	0.72	0.000657
Eif1b	9.69E-09	-0.326544925	0.632	0.712	0.000537
Ptov1	1.35E-08	-0.324841503	0.637	0.719	0.000746
Ergic2	6.28E-07	-0.322426838	0.55	0.623	0.0348
Mir703	3.28E-12	-0.321619277	0.783	0.839	1.82E-07
Lamp1	5.84E-31	-0.320336243	0.966	0.972	3.23E-26
Rtraf	1.70E-19	-0.319610083	0.899	0.93	9.40E-15
Fkbp1a	2.60E-13	-0.319436355	0.82	0.874	1.44E-08
Cetn3	9.52E-11	-0.319398378	0.74	0.815	5.27E-06
Commd3	1.42E-12	-0.31872041	0.779	0.839	7.88E-08
1810037I1	8.74E-08	-0.317610023	0.613	0.693	0.004844
Cd81	1.86E-20	-0.311625281	0.934	0.953	1.03E-15
Rnf7	2.81E-11	-0.310096232	0.757	0.827	1.56E-06
Dpysl3	1.15E-09	-0.30962163	0.654	0.751	6.38E-05
C1d	5.44E-08	-0.308460466	0.609	0.694	0.003014
Gpx8	4.63E-28	-0.308217563	0.967	0.97	2.57E-23
Tmem147	1.96E-10	-0.307631899	0.748	0.816	1.08E-05
Cnih1	1.13E-09	-0.306148456	0.704	0.749	6.25E-05
Lamtor5	3.39E-08	-0.304664075	0.64	0.718	0.001878
Entpd3	7.15E-14	-0.303503761	0.861	0.921	3.96E-09
Bsg	9.38E-30	-0.303278123	0.989	0.988	5.20E-25
Tmed3	3.03E-27	-0.302215827	0.964	0.984	1.68E-22
Tmem176b	4.66E-26	-0.302198009	0.988	0.992	2.58E-21
Uqcrc2	7.50E-08	-0.29974184	0.646	0.722	0.004154
Cuedc2	1.09E-07	-0.297953409	0.594	0.686	0.006025

Taldo1	4.96E-09	-0.297948045	0.748	0.802	0.000275
Atp5c1	7.15E-18	-0.295307459	0.911	0.932	3.96E-13
Rabac1	5.03E-14	-0.294988311	0.873	0.906	2.78E-09
Rcn1	1.08E-15	-0.294194662	0.896	0.926	6.00E-11
Rer1	6.14E-17	-0.293799691	0.893	0.914	3.40E-12
Lamtor4	4.41E-09	-0.293534542	0.728	0.791	0.000244
Gsn	6.37E-18	-0.291899115	0.987	0.992	3.53E-13
Socs3	4.72E-07	-0.291356934	0.632	0.72	0.026144
Sorbs2	1.08E-07	-0.291188838	0.491	0.594	0.00599
Sppl2a	1.23E-10	-0.289337346	0.813	0.864	6.82E-06
Lrrfip2	1.39E-08	-0.288848262	0.71	0.785	0.000768
Insc	1.19E-12	-0.288406269	0.85	0.879	6.60E-08
Smim7	2.21E-07	-0.284624011	0.663	0.73	0.012248
Ergic3	1.22E-14	-0.284138151	0.872	0.894	6.74E-10
Arf5	6.20E-24	-0.283968215	0.964	0.984	3.44E-19
Mrps24	8.28E-08	-0.282289852	0.703	0.759	0.004586
Psmc5	1.10E-07	-0.280445232	0.699	0.754	0.006111
Tle5	8.47E-08	-0.274345326	0.722	0.787	0.004691
Mrpl34	3.98E-07	-0.27247906	0.623	0.709	0.02206
Ndufb7	7.78E-13	-0.27148965	0.892	0.931	4.31E-08
Mrps16	2.28E-08	-0.270390195	0.793	0.824	0.001261
Capns1	3.67E-07	-0.266299217	0.717	0.766	0.020323
Arf1	3.44E-12	-0.264356826	0.88	0.911	1.90E-07
Laptm4a	3.97E-32	-0.264340582	0.984	0.987	2.20E-27
Ppp1ca	2.59E-13	-0.263083164	0.909	0.931	1.44E-08
Cryab	2.23E-09	-0.262114743	0.93	0.964	0.000124
Erlec1	8.49E-09	-0.261884939	0.773	0.831	0.00047
Snhg18	2.79E-17	-0.26163735	0.947	0.961	1.55E-12
Clec11a	4.84E-08	-0.259635701	0.8	0.842	0.002682
Slc25a4	5.99E-16	-0.256925847	0.946	0.961	3.32E-11
Cope	2.69E-17	-0.256003201	0.977	0.976	1.49E-12
Clta	2.80E-16	-0.25426444	0.963	0.974	1.55E-11
Cd200	2.89E-10	-0.252782418	0.84	0.907	1.60E-05
Mmp23	2.81E-08	-0.246181477	0.838	0.86	0.001556
Brk1	2.59E-07	-0.246028916	0.741	0.783	0.014369
Fxyd1	1.19E-13	-0.245308027	0.917	0.959	6.57E-09
Taf10	4.86E-07	-0.245296598	0.78	0.82	0.026947
Edf1	4.52E-12	-0.243054234	0.919	0.943	2.50E-07
Gpx7	6.35E-09	-0.242204287	0.849	0.86	0.000352
Sparc	6.41E-49	-0.241585893	1	1	3.55E-44
Atp6v0b	3.55E-08	-0.241151297	0.828	0.868	0.001968
P3h4	3.74E-08	-0.239412206	0.846	0.87	0.002072
Ost4	1.44E-15	-0.236422311	0.946	0.949	7.99E-11
Pth1r	1.46E-12	-0.2359325	0.99	0.991	8.11E-08
Fis1	1.27E-08	-0.235282019	0.847	0.881	0.000704
Tmem14c	4.27E-09	-0.234301488	0.848	0.86	0.000237
Sf3b5	1.24E-10	-0.231425795	0.903	0.92	6.87E-06

Ssr4	4.10E-12	-0.230949383	0.986	0.988	2.27E-07
Tmem59	4.09E-18	-0.23022746	0.982	0.983	2.27E-13
Ubl5	3.11E-07	-0.229560639	0.82	0.851	0.017253
Uqcrh	4.79E-20	-0.229299804	0.978	0.972	2.65E-15
H13	3.43E-07	-0.226842934	0.798	0.809	0.019018
Calm1	2.84E-10	-0.225258266	0.962	0.972	1.57E-05
Copz2	7.13E-13	-0.224043183	0.954	0.975	3.95E-08
Scand1	1.60E-08	-0.223564568	0.865	0.911	0.000887
Eid1	6.45E-07	-0.222523269	0.815	0.843	0.035759
Serpinf1	2.00E-09	-0.218372971	0.988	0.995	0.000111
Fkbp7	7.43E-13	-0.209156005	0.967	0.973	4.12E-08
Gdi2	1.32E-07	-0.202269969	0.839	0.848	0.007322
Tmem176a	6.34E-12	-0.19941317	0.983	0.99	3.51E-07
Selenow	6.95E-07	-0.195813067	0.894	0.921	0.038484
Metrnl	1.44E-09	-0.19496562	0.947	0.975	8.00E-05
mt-Cytb	1.76E-10	-0.191073911	1	1	9.75E-06
Swi5	8.23E-09	-0.189731806	0.958	0.959	0.000456
Ndufa1	4.65E-07	-0.18956976	0.92	0.94	0.025754
Ift20	8.05E-09	-0.1887621	0.962	0.974	0.000446
Ppic	9.00E-15	-0.186507417	0.996	0.995	4.98E-10
Atp5h	1.31E-12	-0.182602115	0.984	0.989	7.27E-08
Ndfip1	1.32E-09	-0.18007361	0.936	0.939	7.33E-05
Ckb	1.33E-07	-0.178383775	0.983	0.986	0.00735
mt-Nd4	3.70E-07	-0.174319537	1	0.999	0.020512
Reep5	1.23E-07	-0.173556693	0.957	0.968	0.006807
Cgref1	4.69E-07	-0.164310651	0.973	0.976	0.025996
Plod2	2.72E-07	-0.163844946	0.961	0.982	0.015049
Gabarap	1.30E-11	-0.163754317	0.991	0.993	7.22E-07
Ndufb11	5.07E-07	-0.159873943	0.959	0.969	0.028089
Pfn1	2.37E-08	-0.157224023	0.978	0.975	0.00131
Cox8a	2.03E-07	-0.144628302	0.981	0.985	0.01126
Ppib	3.69E-07	-0.137375012	0.993	0.996	0.020438
Selenof	3.25E-08	-0.135469028	0.983	0.979	0.001802
Lgals1	3.32E-08	-0.134610386	0.998	1	0.001841
Gnas	4.78E-11	-0.130317253	0.998	1	2.65E-06

Pre-Obs_DOWN					
Gene	p_val	avg_log2FC	pct.1	pct.2	p_val_adj
Snorc	4.81E-42	-8.454020774	0.001	0.137	2.66E-37
Cpz	6.23E-43	-2.036007898	0.145	0.402	3.45E-38
Prss35	3.09E-20	-2.019712948	0.045	0.177	1.71E-15
Gm36827	1.18E-11	-1.916407056	0.027	0.105	6.53E-07
Eid2	5.77E-15	-1.828460009	0.03	0.124	3.19E-10
Bglap	3.58E-56	-1.784650599	0.864	0.976	1.98E-51
Mgp	4.20E-52	-1.772275692	0.164	0.491	2.33E-47
Plppr5	1.10E-11	-1.696857335	0.04	0.129	6.07E-07
Col11a1	2.44E-57	-1.663425199	0.485	0.772	1.35E-52
Cacna2d3	5.48E-13	-1.65921982	0.04	0.135	3.04E-08
Ecrg4	8.63E-12	-1.645699878	0.073	0.177	4.78E-07
Bglap2	1.05E-61	-1.579515862	0.796	0.974	5.81E-57
Dcn	1.19E-88	-1.554729762	0.691	0.936	6.57E-84
Slc13a5	2.94E-10	-1.552083762	0.058	0.148	1.63E-05
H1f10	3.88E-19	-1.493116615	0.141	0.309	2.15E-14
Srgn	3.22E-09	-1.490650248	0.057	0.14	0.000179
Gnai1	1.04E-33	-1.48856403	0.225	0.463	5.77E-29
Omd	6.50E-20	-1.468860047	0.158	0.338	3.60E-15
Lipc	6.20E-29	-1.425361546	0.206	0.431	3.43E-24
Ier5l	6.85E-12	-1.357864589	0.073	0.18	3.80E-07
Pi15	4.69E-19	-1.354556546	0.178	0.356	2.60E-14
Grrp1	4.19E-18	-1.351556164	0.114	0.274	2.32E-13
Wnt4	7.26E-28	-1.304440725	0.312	0.53	4.02E-23
Lum	1.80E-40	-1.285066404	0.48	0.723	9.96E-36
Ccn5	1.85E-08	-1.252413193	0.151	0.221	0.001022
C130050O1	2.85E-07	-1.251465947	0.038	0.101	0.015788
Mpp6	8.88E-13	-1.237500303	0.077	0.192	4.92E-08
Mtg2	5.57E-07	-1.215177804	0.047	0.113	0.030862
Jdp2	1.35E-13	-1.21290459	0.113	0.247	7.47E-09
B3galt6	4.33E-07	-1.18939798	0.059	0.131	0.024014
7SK.293	1.51E-07	-1.149756652	0.076	0.158	0.008361
Serpinb1a	3.99E-08	-1.146696388	0.057	0.135	0.002211
Entpd3	7.12E-15	-1.138390491	0.188	0.338	3.94E-10
Cdk2ap1	5.21E-25	-1.135293078	0.198	0.421	2.89E-20
Rdm1	1.10E-17	-1.128884913	0.121	0.281	6.10E-13
Sparc	1.56E-54	-1.103211996	0.996	1	8.66E-50
Car3	6.11E-52	-1.103074592	0.814	0.885	3.39E-47
Rpp25l	2.37E-09	-1.101668056	0.083	0.18	0.000131
Rsrp1	4.36E-37	-1.093990378	0.5	0.747	2.41E-32
Filip1l	1.13E-13	-1.081468935	0.152	0.299	6.24E-09
Chn1	4.77E-22	-1.055099964	0.152	0.346	2.64E-17
Prorsd1	1.46E-13	-1.0506696	0.174	0.322	8.07E-09
Ppp1r3b	3.45E-07	-1.034002641	0.052	0.12	0.019095
Tmem86a	7.30E-10	-1.03100674	0.235	0.332	4.04E-05
D830050J1	8.92E-07	-1.029450587	0.046	0.107	0.049441

AU021092	9.76E-15	-1.00856337	0.224	0.386	5.41E-10
Ppp2cb	4.04E-10	-0.997933951	0.094	0.199	2.24E-05
Serpinf1	5.59E-26	-0.986577759	0.661	0.805	3.10E-21
Cd1d1	2.48E-15	-0.9858394	0.23	0.395	1.37E-10
Il12a	1.19E-16	-0.980111164	0.148	0.311	6.59E-12
Mageh1	3.91E-09	-0.979083976	0.087	0.183	0.000216
Zfp637	9.30E-14	-0.974392844	0.111	0.243	5.15E-09
Smoc2	6.00E-07	-0.960530437	0.126	0.216	0.033249
Wtip	1.41E-13	-0.958994734	0.157	0.305	7.79E-09
0610009B2	2.20E-09	-0.958641954	0.109	0.216	0.000122
Anxa3	9.59E-09	-0.926199958	0.074	0.16	0.000532
A430005L1	5.52E-09	-0.922031624	0.119	0.226	0.000306
Ybx3	4.47E-16	-0.920820364	0.254	0.433	2.47E-11
Trappc1	2.00E-07	-0.913690524	0.086	0.17	0.011108
Ing1	6.25E-12	-0.910980292	0.167	0.306	3.46E-07
Ppp1r11	9.27E-10	-0.896429881	0.133	0.25	5.14E-05
Hnrnpa0	1.41E-32	-0.894456708	0.512	0.744	7.81E-28
Dkk1	1.26E-07	-0.892384109	0.167	0.271	0.006958
Exosc4	1.31E-12	-0.878536248	0.151	0.291	7.28E-08
C1d	5.68E-19	-0.873647579	0.324	0.523	3.15E-14
Tmed4	5.29E-09	-0.87115133	0.162	0.277	0.000293
Bckdk	1.72E-07	-0.870896144	0.11	0.203	0.009529
Col11a2	8.07E-22	-0.865019047	0.418	0.633	4.47E-17
Comp	2.25E-09	-0.862207239	0.303	0.431	0.000125
Slc36a2	5.90E-26	-0.855119592	0.466	0.682	3.27E-21
Smagp	1.44E-12	-0.843323974	0.227	0.382	7.98E-08
Slc35a2	1.07E-07	-0.843042832	0.13	0.23	0.005952
Sirt1	1.25E-08	-0.842856034	0.144	0.255	0.000695
Tmub2	1.25E-07	-0.839093848	0.118	0.214	0.006915
Lypla2	2.41E-08	-0.837663115	0.081	0.168	0.001335
Bet1	1.24E-13	-0.828323486	0.258	0.422	6.86E-09
Mospd1	2.30E-07	-0.821394312	0.068	0.141	0.012748
Fibp	1.16E-10	-0.821284481	0.195	0.332	6.40E-06
Sra1	1.14E-19	-0.813254231	0.389	0.588	6.32E-15
Mplkip	6.13E-13	-0.811854383	0.17	0.317	3.40E-08
Pigyl	3.44E-11	-0.810829904	0.202	0.344	1.90E-06
Coa3	7.46E-21	-0.805658772	0.349	0.567	4.13E-16
Mlip	7.80E-17	-0.805462849	0.359	0.537	4.32E-12
Hspb2	3.82E-14	-0.801315994	0.329	0.487	2.12E-09

Osteo-CAR_DOWN					
**Gene**	**p_val**	**avg_log2FC**	**pct.1**	**pct.2**	**p_val_adj**
Snorc	4.01E-16	-6.90769226	0.002	0.125	2.22E-11
Gm36827	3.00E-08	-2.915427433	0.035	0.135	0.001662
Ppp2cb	9.51E-08	-1.565364894	0.064	0.19	0.00527
Omd	2.69E-15	-1.509435498	0.268	0.511	1.49E-10
Mpp6	2.73E-10	-1.478157698	0.2	0.378	1.51E-05
Lum	4.74E-18	-1.371113313	0.363	0.627	2.63E-13
Eid2	4.35E-07	-1.354338508	0.066	0.185	0.024085
Rsrp1	9.39E-17	-1.244874726	0.411	0.652	5.20E-12
Znhit6	2.69E-08	-1.241313724	0.18	0.336	0.001491
Cdk2ap1	1.69E-08	-1.215612167	0.171	0.341	0.000937
Ybx3	1.52E-09	-1.205621397	0.202	0.388	8.42E-05
Col11a1	3.82E-08	-1.159040113	0.281	0.464	0.002117
Plppr5	1.71E-07	-1.158583632	0.136	0.291	0.009469
Ier5l	7.55E-08	-1.101263119	0.116	0.268	0.004185
Bglap	8.86E-18	-1.099853144	0.727	0.937	4.91E-13
Hnrnpa0	2.74E-18	-1.052576013	0.448	0.694	1.52E-13
Dcn	3.03E-16	-1.029941955	0.736	0.89	1.68E-11
Car3	2.47E-11	-0.993477935	0.556	0.732	1.37E-06
Mbd2	1.47E-09	-0.985991864	0.286	0.484	8.12E-05
Itm2a	2.00E-08	-0.940633853	0.288	0.471	0.00111
Twist1	3.14E-11	-0.906242916	0.389	0.614	1.74E-06
Fn1	7.96E-10	-0.776876574	0.396	0.614	4.41E-05
Metrnl	1.61E-07	-0.772743507	0.387	0.551	0.008924
Atraid	2.10E-10	-0.716624686	0.532	0.692	1.16E-05
Bglap2	2.62E-09	-0.690821029	0.673	0.847	0.000145
Rasl11a	6.35E-08	-0.667052944	0.503	0.689	0.003519
Lmo4	1.91E-10	-0.648931703	0.662	0.799	1.06E-05
Comp	1.70E-07	-0.647404565	0.418	0.607	0.009412
Vldlr	5.21E-07	-0.626868601	0.455	0.632	0.028841
Pmepa1	3.05E-08	-0.609635847	0.62	0.754	0.001687
Shox2	3.23E-09	-0.596644259	0.668	0.817	0.000179
Jun	6.53E-07	-0.486459152	0.895	0.915	0.036177
Wif1	1.84E-07	-0.479198529	0.897	0.937	0.010218
Aldh2	4.83E-08	-0.464596325	0.776	0.847	0.002675
Tmem59	3.93E-11	-0.421945732	0.908	0.965	2.18E-06
Fap	8.14E-08	-0.409719203	0.864	0.935	0.004511
Tpm1	8.09E-12	-0.373338333	0.998	1	4.48E-07
Rps7	6.65E-08	-0.347360811	0.934	0.942	0.003682
Laptm4a	3.40E-08	-0.312624118	0.954	0.962	0.001884

Adipo CAR_DOWN					
**Gene**	**p_val**	**avg_log2FC**	**pct.1**	**pct.2**	**p_val_adj**
Snorc	3.68E-36	-9.056082133	0	0.125	2.04E-31
Timp4	6.57E-17	-1.868430385	0.053	0.185	3.64E-12
Itm2a	7.77E-29	-1.77980079	0.126	0.352	4.30E-24
Car3	1.20E-10	-1.699324226	0.075	0.181	6.65E-06
Col2a1	4.47E-19	-1.687102278	0.065	0.218	2.48E-14
Col11a1	3.81E-15	-1.567879361	0.081	0.22	2.11E-10
Ier5l	1.20E-14	-1.481230651	0.122	0.261	6.62E-10
Cnnm1	1.02E-09	-1.477853149	0.075	0.165	5.67E-05
Bglap	4.27E-62	-1.400327472	0.364	0.767	2.37E-57
Ifitm1	1.73E-07	-1.397909581	0.08	0.162	0.009572
Wtip	6.83E-14	-1.379476216	0.095	0.233	3.79E-09
Ppp2cb	5.17E-11	-1.333212782	0.099	0.21	2.87E-06
Jdp2	4.13E-18	-1.299649532	0.174	0.346	2.29E-13
Col11a2	3.99E-08	-1.190888237	0.051	0.131	0.002209
Wnt4	4.64E-14	-1.160289358	0.173	0.328	2.57E-09
Apod	3.84E-08	-1.155737415	0.124	0.218	0.002125
Bglap2	2.00E-25	-1.134435058	0.216	0.461	1.11E-20
Cdk2ap1	5.93E-09	-1.105893759	0.127	0.224	0.000328
Basp1	3.45E-22	-1.054876823	0.368	0.497	1.91E-17
Amy1	1.12E-17	-1.034658268	0.299	0.468	6.19E-13
Mpp6	6.08E-08	-0.995092575	0.14	0.238	0.003369
Twist1	1.70E-11	-0.979392326	0.207	0.354	9.42E-07
Lamtor3	2.96E-07	-0.946936353	0.136	0.222	0.016425
Slc40a1	4.82E-08	-0.896972129	0.199	0.306	0.002672
Ccn5	1.30E-09	-0.863335009	0.319	0.439	7.21E-05
Mbd2	3.56E-16	-0.830719782	0.368	0.536	1.97E-11
Ibsp	4.23E-17	-0.798392064	0.662	0.808	2.34E-12
Comp	6.78E-09	-0.790351092	0.129	0.244	0.000376
Hnrnpa0	2.68E-16	-0.786315196	0.396	0.528	1.49E-11
Ybx3	3.31E-07	-0.773607721	0.217	0.316	0.018317
Rspo3	3.51E-15	-0.769821901	0.503	0.633	1.94E-10
Il34	2.77E-15	-0.740428304	0.517	0.615	1.53E-10
Wif1	2.31E-07	-0.730255061	0.366	0.404	0.012774
Tm4sf1	5.32E-10	-0.713105879	0.392	0.524	2.95E-05
Tmem159	3.16E-11	-0.707859585	0.344	0.416	1.75E-06
Agt	1.99E-35	-0.706378757	0.838	0.932	1.10E-30
Pon2	2.65E-16	-0.705164469	0.526	0.654	1.47E-11
Wls	3.56E-08	-0.672871144	0.304	0.395	0.001972
Vcam1	4.17E-44	-0.666540733	0.96	0.989	2.31E-39
Angptl1	3.11E-07	-0.660095152	0.35	0.446	0.017235
Ecrg4	4.05E-08	-0.646680501	0.365	0.486	0.002244
Idh2	5.89E-08	-0.642278512	0.319	0.431	0.003266
Smpdl3a	6.19E-11	-0.615317799	0.417	0.509	3.43E-06
Tsc22d1	2.66E-14	-0.615012064	0.65	0.744	1.47E-09
Tspo	2.95E-07	-0.601944341	0.359	0.45	0.016318

Rsrp1	1.28E-10	-0.594077288	0.504	0.622	7.07E-06
Spop	1.63E-07	-0.590308189	0.332	0.42	0.009054
Cd1d1	4.03E-11	-0.58883979	0.5	0.597	2.23E-06
Ypel3	3.78E-09	-0.584577841	0.434	0.541	0.00021
Eva1b	5.55E-07	-0.575370301	0.346	0.415	0.030762
Gadd45g	4.77E-07	-0.57161436	0.682	0.716	0.026438
Ube2m	5.18E-08	-0.567547208	0.384	0.475	0.00287
Sfrp4	5.89E-10	-0.554756357	0.678	0.751	3.27E-05
Prpf4b	8.34E-07	-0.553221647	0.351	0.4	0.046202
Id4	8.80E-10	-0.552192641	0.535	0.633	4.88E-05
Clu	1.29E-24	-0.549669353	0.904	0.951	7.17E-20
St3gal6	1.96E-11	-0.547785547	0.558	0.653	1.08E-06
Dstn	2.31E-07	-0.546376719	0.329	0.389	0.012823
Adipoq	6.33E-35	-0.542427157	0.976	0.99	3.51E-30
Fcgrt	1.35E-20	-0.54033599	0.777	0.852	7.47E-16
Cdkn1c	4.09E-07	-0.532517982	0.446	0.516	0.022666
Ryk	4.55E-07	-0.531012768	0.385	0.498	0.025214
Igf1	3.10E-13	-0.528707159	0.698	0.778	1.72E-08
Pten	6.60E-08	-0.519437834	0.431	0.506	0.003656
Cd9	6.12E-12	-0.511030333	0.671	0.747	3.39E-07
Cebpb	6.44E-07	-0.503841876	0.639	0.687	0.035664
Lpl	2.15E-10	-0.502789825	0.617	0.678	1.19E-05
Htra1	7.78E-08	-0.497245006	0.456	0.502	0.004312
Cpq	2.60E-12	-0.484814669	0.676	0.754	1.44E-07
Tm2d2	1.97E-07	-0.460522309	0.428	0.491	0.010932
Sbds	3.01E-09	-0.458970418	0.442	0.478	0.000167
Tspan4	1.18E-07	-0.45653132	0.499	0.548	0.006541
Hmgn3	2.52E-08	-0.455656259	0.56	0.63	0.001397
Epdr1	3.58E-07	-0.455345988	0.53	0.593	0.019814
Itm2b	8.52E-44	-0.452606219	0.995	0.997	4.72E-39
Col6a1	3.26E-14	-0.45177688	0.813	0.886	1.81E-09
Col6a2	1.94E-07	-0.446524065	0.582	0.646	0.01075
Sri	1.08E-08	-0.438744726	0.48	0.531	0.000597
Lrpap1	1.72E-07	-0.434065682	0.53	0.605	0.009526
mt-Rnr1	2.35E-11	-0.415251537	0.981	0.997	1.30E-06
Eif3h	8.99E-07	-0.409222941	0.497	0.556	0.04982
Vegfc	4.64E-08	-0.4014953	0.691	0.763	0.002569
Mgst1	1.68E-12	-0.401268746	0.844	0.867	9.33E-08
H3f3a	8.59E-10	-0.398209874	0.696	0.758	4.76E-05
mt-Rnr2	2.14E-16	-0.39514681	0.995	0.999	1.18E-11
Tmem59	6.71E-19	-0.390024897	0.902	0.913	3.72E-14
Mme	5.21E-08	-0.389697838	0.741	0.811	0.002887
Vdac2	5.28E-07	-0.387867588	0.496	0.536	0.02926
Ppic	1.22E-08	-0.384325403	0.678	0.718	0.000675
Jun	5.19E-12	-0.378201938	0.919	0.876	2.87E-07
Sparc	6.14E-22	-0.377966957	1	1	3.40E-17
Abi3bp	8.80E-09	-0.37584418	0.802	0.835	0.000488

Ergic3	3.15E-07	-0.363926191	0.583	0.624	0.017478
mt-Cytb	2.07E-24	-0.362403056	0.998	1	1.15E-19
Prdx4	4.11E-08	-0.358184801	0.727	0.741	0.002279
Rpl6	1.07E-11	-0.357589334	0.847	0.876	5.94E-07
Pappa	8.14E-07	-0.351767549	0.775	0.803	0.045078
Bsg	7.93E-15	-0.351382523	0.911	0.912	4.39E-10
Eif3f	1.10E-07	-0.345843293	0.706	0.756	0.006085
Slc25a4	2.81E-11	-0.340138209	0.845	0.868	1.55E-06
Pfdn5	7.57E-13	-0.339529803	0.884	0.91	4.19E-08
Dpep1	1.86E-11	-0.335222725	0.808	0.793	1.03E-06
Islr	1.72E-09	-0.330130453	0.824	0.839	9.53E-05
Cd81	1.82E-10	-0.329411096	0.895	0.918	1.01E-05
Eef1d	2.38E-07	-0.31344562	0.662	0.684	0.013169
H2-D1	6.24E-29	-0.308333912	1	1	3.46E-24
Snrpb	2.93E-07	-0.307575894	0.64	0.661	0.01621
Arf5	1.87E-07	-0.303901978	0.671	0.688	0.010358
mt-Nd4	2.08E-12	-0.30119765	0.992	0.997	1.15E-07
Gpx8	8.85E-10	-0.296286394	0.888	0.898	4.90E-05
Gdpd2	1.04E-07	-0.29032692	0.897	0.941	0.005778
Ptma	2.54E-10	-0.286675571	0.922	0.912	1.41E-05
Selenop	1.99E-07	-0.279442731	0.914	0.932	0.011024
Ppib	5.43E-12	-0.27453036	0.966	0.962	3.01E-07
Cd63	1.32E-10	-0.26769822	0.984	0.984	7.30E-06
Cops9	3.47E-09	-0.266348191	0.735	0.717	0.000192
Laptm4a	1.08E-11	-0.265993958	0.957	0.956	6.00E-07
Dynll1	2.97E-07	-0.261056812	0.847	0.856	0.016449
Rps5	2.75E-11	-0.258766358	0.968	0.974	1.52E-06
Ctsb	3.28E-09	-0.252304258	0.929	0.91	0.000182
Rpl18a	6.81E-09	-0.226855829	0.956	0.952	0.000377
Naca	6.22E-08	-0.223173348	0.869	0.852	0.003445
Lamp1	4.75E-07	-0.21370765	0.928	0.925	0.026296
Rpl8	2.19E-08	-0.212685526	0.974	0.978	0.001212
Rps2	2.17E-07	-0.212346695	0.965	0.973	0.012022
