# Supplementary material for: The Aging Landscape by scRNAseq of Mesenchymal Lineage Cells in Mouse Bone
Source: Aging Cell. 2025 Oct 13;24(12):e70256. doi: 10.1111/acel.70256 (PMC12686594; doi:10.1111/acel.70256)
Supplement: Supplementary file 13 — Table S3: acel70256‐sup‐0013‐TableS3.pdf. [file ACEL-24-e70256-s007.pdf]

Osteoblasts_UP					
**Gene**	**p_val**	**avg_log2FC**	**pct.1**	**pct.2**	**p_val_adj**
Mmp13	1.35E-12	3.053953394	0.312	0.127	7.45E-08
Thbs4	2.22E-08	2.690026229	0.153	0.04	0.001228
Nme2	5.12E-15	2.617466211	0.227	0.059	2.84E-10
Cdkn2a	8.20E-18	2.524878143	0.29	0.074	4.54E-13
Angpt4	3.55E-12	2.0788178	0.33	0.182	1.97E-07
Ccn2	1.03E-16	2.048523224	0.665	0.472	5.71E-12
Synpo	8.52E-09	2.044971534	0.207	0.087	0.000472
Scube3	6.32E-09	1.954744436	0.185	0.055	0.00035
Lsp1	1.32E-07	1.924919257	0.156	0.045	0.007303
S100a6	7.39E-17	1.734874087	0.673	0.464	4.09E-12
Myl9	1.71E-07	1.715050895	0.219	0.085	0.009459
Rgs5	2.74E-07	1.67663392	0.179	0.061	0.015202
Gchfr	2.01E-07	1.652412506	0.222	0.098	0.011148
Spp1	9.76E-18	1.566398451	0.966	0.875	5.41E-13
Aebp1	1.78E-08	1.460118869	0.349	0.191	0.000986
Uba52	1.61E-37	1.445973559	0.773	0.617	8.93E-33
Serpine2	3.25E-17	1.40729583	0.946	0.854	1.80E-12
Limch1	6.89E-10	1.399781908	0.463	0.366	3.82E-05
Porcn	6.97E-08	1.284305494	0.355	0.254	0.003861
Id3	1.60E-10	1.280501093	0.423	0.335	8.87E-06
Ccnd1	7.75E-16	1.233718854	0.739	0.53	4.29E-11
Mylk	1.17E-14	1.157652392	0.653	0.407	6.47E-10
Anxa1	1.56E-09	1.150811976	0.693	0.519	8.66E-05
Samhd1	8.76E-08	1.127883489	0.395	0.261	0.004851
Rpl29	1.82E-09	1.100911563	0.392	0.265	0.000101
Tagln2	3.77E-08	1.100197585	0.48	0.343	0.002091
Cp	1.19E-10	1.00428131	0.801	0.68	6.57E-06
Gstp1	4.02E-12	1.001134908	0.577	0.451	2.23E-07
Tmem208	5.32E-11	0.975821063	0.423	0.341	2.95E-06
Acta2	1.23E-07	0.960709781	0.182	0.061	0.006795
Wif1	1.44E-07	0.898847489	0.651	0.523	0.007961
Bst2	8.35E-07	0.890718127	0.699	0.585	0.046274
Snai1	2.69E-08	0.885170079	0.54	0.46	0.001493
Pdcd2	6.10E-07	0.875912143	0.284	0.233	0.033817
Gas6	3.35E-08	0.867897398	0.298	0.136	0.001855
Tspo	2.16E-11	0.828192447	0.679	0.557	1.19E-06
Tsc22d3	2.11E-07	0.806862372	0.594	0.534	0.011706
Psmg4	5.34E-08	0.795029527	0.565	0.447	0.002956
Psme1	1.58E-07	0.79045107	0.554	0.441	0.008763
Pdgfa	7.64E-13	0.782072415	0.903	0.814	4.23E-08
Tm4sf1	3.68E-07	0.779988789	0.784	0.695	0.020387
Timp2	1.57E-15	0.771863775	0.866	0.805	8.73E-11
Nudt4	2.01E-10	0.758691271	0.804	0.689	1.12E-05
Btf3	6.12E-27	0.756101376	0.912	0.83	3.39E-22
Gapdh	1.02E-21	0.753897476	0.895	0.83	5.64E-17

Park7	8.58E-08	0.749854277	0.494	0.411	0.004754
Timp1	4.11E-13	0.7471883	0.886	0.85	2.28E-08
H2-Q4	3.05E-08	0.743079041	0.696	0.559	0.00169
Rbm3	1.01E-10	0.722706144	0.75	0.631	5.58E-06
Ebp	4.20E-11	0.702023394	0.801	0.718	2.33E-06
Mrpl20	2.55E-11	0.699429821	0.702	0.636	1.41E-06
Arpc1b	2.53E-07	0.680388801	0.608	0.498	0.014035
Eno1	2.99E-07	0.677815407	0.602	0.458	0.016567
Lrp4	2.94E-07	0.677162175	0.656	0.604	0.016293
Ifitm3	1.04E-08	0.665638742	0.903	0.831	0.000575
Etfa	8.62E-09	0.66324503	0.693	0.568	0.000478
Nedd8	6.23E-09	0.660832139	0.685	0.638	0.000345
Gpx3	8.13E-15	0.660130314	0.991	0.975	4.50E-10
Nme1	6.48E-14	0.653643154	0.855	0.777	3.59E-09
Cst3	1.54E-15	0.642119579	0.997	0.996	8.51E-11
Ppia	8.48E-16	0.636869578	0.889	0.843	4.70E-11
Cdc42ep3	7.10E-09	0.627690108	0.892	0.835	0.000393
Slc29a1	8.12E-08	0.615264457	0.636	0.623	0.004498
Pfdn6	3.15E-10	0.609707729	0.673	0.642	1.74E-05
Myh9	7.13E-08	0.599472129	0.81	0.733	0.003951
Ndufv2	7.20E-13	0.589473609	0.835	0.765	3.99E-08
Rpl15	9.57E-20	0.579061224	0.92	0.873	5.30E-15
Psmd12	6.06E-07	0.570171808	0.688	0.597	0.03355
Colec12	6.18E-07	0.565132132	0.838	0.775	0.034267
Pdia6	1.42E-09	0.564065642	0.889	0.852	7.85E-05
Rps6	4.28E-20	0.562473559	0.949	0.902	2.37E-15
Ptgis	2.79E-17	0.551965676	0.966	0.934	1.55E-12
Gm10076	6.60E-13	0.551082721	0.938	0.873	3.66E-08
Ccdc47	8.97E-07	0.549567322	0.707	0.633	0.0497
Rbm8a	2.00E-09	0.547284874	0.793	0.729	0.000111
Psmb1	2.11E-19	0.545865368	0.94	0.879	1.17E-14
Anxa2	5.61E-09	0.541762726	0.844	0.843	0.000311
Lmna	6.66E-07	0.537110066	0.869	0.89	0.036882
Ndufb10	8.90E-16	0.535219107	0.884	0.848	4.93E-11
Ifitm2	6.76E-07	0.531219056	0.753	0.68	0.037426
Tubb5	1.90E-10	0.527571816	0.915	0.884	1.05E-05
Lbh	1.29E-07	0.511691834	0.784	0.775	0.007158
Actr3	1.18E-08	0.507655727	0.824	0.758	0.000654
Psma7	9.71E-10	0.502347088	0.92	0.839	5.38E-05
Rps18	3.30E-20	0.501176595	0.977	0.938	1.83E-15
Mcfd2	3.83E-07	0.496745264	0.741	0.688	0.021197
Clic1	7.10E-08	0.494911841	0.818	0.78	0.003932
Fam162a	6.58E-07	0.490917251	0.793	0.714	0.036475
Cct4	8.80E-07	0.490522844	0.801	0.733	0.048743
Ibsp	1.94E-07	0.482382681	1	0.998	0.01076
Gtf2h5	1.80E-09	0.474235399	0.886	0.828	9.96E-05
Dnaja2	6.51E-08	0.471488631	0.764	0.727	0.003605

Pebp1	4.72E-08	0.468760953	0.696	0.655	0.002615
Eef1a1	8.04E-21	0.46076271	0.991	0.964	4.45E-16
Psma4	1.63E-07	0.460173518	0.847	0.78	0.009049
Cct5	2.14E-07	0.45846815	0.875	0.794	0.011844
Rpl3	7.74E-20	0.457207649	0.98	0.955	4.29E-15
Phb2	6.28E-07	0.453506582	0.801	0.733	0.034797
Calr	5.33E-10	0.453333348	0.955	0.951	2.95E-05
Rpl36	1.06E-10	0.447750523	0.989	0.943	5.89E-06
Psmb4	1.83E-09	0.44612902	0.926	0.85	0.000101
Cyba	2.15E-07	0.446003139	0.855	0.778	0.011891
Psma2	7.13E-08	0.443487497	0.827	0.758	0.003948
Bag1	2.79E-09	0.441879618	0.915	0.837	0.000155
Fkbp11	2.74E-08	0.437772864	0.94	0.932	0.00152
Hint1	3.44E-12	0.434968975	0.94	0.886	1.90E-07
Tgfb1	2.83E-09	0.434413158	0.909	0.871	0.000157
H2-D1	7.17E-15	0.426150675	0.994	0.991	3.97E-10
Rps2	7.38E-15	0.422804014	0.997	0.991	4.09E-10
Yipf4	5.80E-08	0.422403321	0.795	0.761	0.003216
Emc7	8.13E-07	0.411508565	0.812	0.765	0.045059
Rps28	2.91E-09	0.411290538	0.92	0.886	0.000161
H2-K1	1.29E-09	0.408683531	0.994	0.973	7.17E-05
Hsp90ab1	1.13E-13	0.407482524	0.991	0.981	6.25E-09
Tsn	3.05E-07	0.405803869	0.824	0.801	0.016907
Rpl10a	4.67E-15	0.403106774	0.983	0.958	2.59E-10
Tmem176a	1.70E-09	0.393537753	0.977	0.962	9.41E-05
Psma3	1.94E-07	0.390327145	0.901	0.837	0.010761
Micos13	6.80E-08	0.385570732	0.898	0.856	0.003765
Psap	8.52E-07	0.385360133	0.881	0.854	0.047204
Rpl13	2.55E-18	0.383644017	1	0.981	1.41E-13
Rps3	2.55E-09	0.38305163	0.923	0.9	0.000141
Ftl1	1.33E-07	0.37772625	0.952	0.913	0.007389
Eif3k	1.44E-10	0.375429676	0.915	0.883	7.95E-06
Eif3c	2.27E-07	0.374218049	0.932	0.915	0.012582
Rpl27a	1.99E-11	0.366914784	0.963	0.936	1.10E-06
Myl6	3.12E-09	0.361544974	0.966	0.943	0.000173
Rpl7	1.32E-11	0.360001638	0.952	0.913	7.30E-07
Aurkaip1	5.23E-07	0.358077532	0.881	0.858	0.028949
Ybx1	1.81E-11	0.358042033	0.977	0.926	1.00E-06
Rpl8	8.98E-15	0.356572319	0.994	0.989	4.98E-10
Hspa8	2.98E-07	0.352726032	0.98	0.947	0.016489
Slc25a4	7.78E-09	0.349231859	0.949	0.92	0.000431
Rpl19	1.03E-14	0.347223028	0.986	0.964	5.71E-10
Pdia3	5.21E-08	0.346885604	0.972	0.955	0.002889
Srp14	3.18E-07	0.345766395	0.835	0.83	0.017614
Rpl18	5.46E-13	0.341859091	0.966	0.964	3.03E-08
Rpl12	5.65E-12	0.340448844	0.983	0.953	3.13E-07
Vapa	8.99E-08	0.33987291	0.889	0.894	0.004982

Rps5	7.00E-13	0.337142686	0.991	0.972	3.88E-08
Calm2	1.77E-09	0.33324243	0.991	0.975	9.82E-05
Atp5a1	1.75E-07	0.331933104	0.92	0.896	0.009676
Cox4i1	4.54E-11	0.327971217	0.972	0.934	2.51E-06
Tmem176b	1.54E-07	0.323793118	0.963	0.955	0.008552
Naca	1.18E-07	0.311140429	0.943	0.915	0.00652
Actb	9.62E-09	0.310250423	0.997	1	0.000533
Rpl32	2.29E-10	0.306126272	0.991	0.973	1.27E-05
Rpl17	1.62E-10	0.305231201	0.991	0.941	8.97E-06
Rpl14	3.66E-09	0.3027733	0.989	0.962	0.000203
Rps4x	3.03E-09	0.302234078	0.983	0.958	0.000168
Rhoa	5.06E-07	0.301550277	0.926	0.898	0.028043
Elob	1.97E-07	0.295387472	0.952	0.932	0.010931
Rpl9	4.08E-09	0.287433959	0.98	0.972	0.000226
Rps11	1.19E-09	0.282212365	0.974	0.949	6.60E-05
Rps3a1	2.58E-09	0.27868197	0.991	0.972	0.000143
Ppib	1.87E-07	0.273497494	0.972	0.968	0.01036
Rps9	4.33E-09	0.269866823	0.994	0.966	0.00024
Rplp0	2.05E-09	0.268090065	0.986	0.983	0.000114
Nedd4	3.89E-09	0.246792945	0.977	0.985	0.000215
Rps14	9.09E-08	0.239578428	0.989	0.958	0.005037
Rpl4	4.54E-07	0.232119648	0.994	0.985	0.025164
Rps8	1.71E-07	0.226287625	1	0.992	0.009466
Rpl26	5.60E-07	0.218854963	0.991	0.953	0.03103

Pre-Obs_UP					
**Gene**	**p_val**	**avg_log2FC**	**pct.1**	**pct.2**	**p_val_adj**
Gvin-ps7	1.36E-12	5.030845036	0.106	0.003	7.55E-08
F7	1.95E-08	2.795179426	0.136	0.034	0.001078
Sdf2l1	2.46E-07	2.616137036	0.215	0.101	0.013644
Cdkn2a	7.20E-09	1.844810478	0.209	0.073	0.000399
Cxcl14	2.46E-21	1.750399553	0.611	0.312	1.36E-16
Ace	6.57E-11	1.546982502	0.333	0.152	3.64E-06
Gas6	7.51E-18	1.149782526	0.827	0.626	4.16E-13
Uba52	1.36E-12	1.136271774	0.586	0.438	7.53E-08
H2-Q10	2.48E-07	1.094897838	0.396	0.244	0.013745
Ecm1	3.65E-08	0.900197264	0.561	0.404	0.002022
C1s1	1.75E-07	0.877403223	0.384	0.216	0.00968
Rarres2	4.94E-08	0.817625543	0.543	0.36	0.002737
Serping1	2.44E-08	0.816173573	0.698	0.57	0.001354
Tspo	1.33E-08	0.809094543	0.602	0.452	0.000736
Fhl2	1.90E-09	0.798414548	0.658	0.466	0.000105
Epas1	1.13E-07	0.785374653	0.665	0.539	0.006266
Ptgis	1.04E-14	0.722389196	0.919	0.879	5.78E-10
Hp	8.65E-07	0.719984436	0.58	0.41	0.04795
Fam20a	2.74E-08	0.707996136	0.426	0.244	0.001518
Spp1	2.72E-15	0.685854253	1	0.972	1.51E-10
Tns3	2.11E-10	0.669627833	0.865	0.758	1.17E-05
Cdc42ep3	1.95E-08	0.658857856	0.874	0.837	0.001082
Nudt4	1.07E-09	0.620753699	0.904	0.843	5.95E-05
Limch1	1.62E-09	0.616676785	0.842	0.744	8.98E-05
Ccn2	2.52E-07	0.520362288	0.924	0.826	0.013988
Tmem176a	2.95E-13	0.519002183	0.987	0.975	1.63E-08
Tmem176b	8.19E-13	0.503384764	0.99	0.961	4.54E-08
Timp2	5.43E-09	0.484648845	0.928	0.868	0.000301
S100a6	1.98E-07	0.451221208	0.918	0.829	0.010973
Mmp13	1.21E-14	0.425447082	0.916	0.725	6.68E-10
Colec12	1.19E-07	0.370301298	0.961	0.93	0.006583
Rpl7	1.13E-09	0.349805027	0.976	0.935	6.25E-05
Rpl3	7.48E-10	0.344670565	0.979	0.958	4.14E-05
Ubb	2.23E-07	0.335666716	0.985	0.983	0.012378
Calr	5.69E-07	0.334143638	0.858	0.91	0.031517
Cox4i1	2.77E-07	0.281865156	0.972	0.949	0.015337
Rpl19	3.71E-07	0.247401501	0.997	0.989	0.020549
Rpl13	3.09E-07	0.234781722	0.999	0.997	0.017139

Osteo CAR_UP					
**Gene**	**p_val**	**avg_log2FC**	**pct.1**	**pct.2**	**p_val_adj**
Aldh1a2	2.25E-09	1.949972262	0.355	0.129	0.000125
Tmem86a	2.34E-09	1.844395764	0.465	0.228	0.00013
Mgp	2.51E-08	1.731942073	0.676	0.49	0.001391
Prss23	7.39E-10	1.605797793	0.487	0.233	4.09E-05
Ecm1	2.70E-17	1.274189349	0.803	0.604	1.50E-12
Aebp1	5.04E-07	1.272523427	0.504	0.302	0.027913
Ltbp2	1.16E-07	1.256804213	0.521	0.292	0.006422
7530428D23	6.24E-08	1.15951221	0.631	0.446	0.003458
Cxcl14	4.31E-12	1.129127829	0.896	0.817	2.39E-07
Sfrp1	3.49E-08	1.113127886	0.594	0.356	0.001932
Rarres2	6.93E-10	1.107368773	0.746	0.554	3.84E-05
Fgf7	8.14E-09	1.08988327	0.752	0.599	0.000451
Kng2	1.01E-08	1.053143254	0.42	0.178	0.000558
C4b	9.46E-08	1.037605354	0.665	0.465	0.005243
Hp	2.68E-08	0.952935966	0.865	0.678	0.001484
C1s1	1.95E-07	0.952285501	0.628	0.406	0.010826
H2-Q10	6.30E-08	0.924667599	0.668	0.436	0.003492
Serping1	2.22E-09	0.716253979	0.944	0.861	0.000123
Tmem176b	2.07E-15	0.671435828	0.997	0.98	1.15E-10
Tmsb4x	2.34E-08	0.641260142	0.997	1	0.001297
Calr	1.45E-08	0.615772432	0.932	0.866	0.000803
Tmem176a	7.54E-14	0.59021031	0.983	0.98	4.18E-09
Fth1	1.21E-10	0.556681059	0.992	1	6.68E-06
Ifitm3	8.77E-08	0.471011128	0.992	0.975	0.004857

AdipoCAR_UP					
**Gene**	**p_val**	**avg_log2FC**	**pct.1**	**pct.2**	**p_val_adj**
Ly6a	1.44E-20	5.645612683	0.227	0.018	7.96E-16
H2-Ab1	1.88E-07	2.85640994	0.162	0.052	0.010401
Avpr1a	9.45E-08	2.713881211	0.127	0.028	0.005233
Gsn	6.41E-17	2.657292216	0.643	0.41	3.55E-12
Ccnd1	3.70E-09	2.643746808	0.166	0.043	0.000205
Snhg11	6.59E-08	2.344535171	0.158	0.043	0.00365
Lum	5.42E-11	2.181629811	0.339	0.171	3.00E-06
Cybb	3.53E-11	2.122096576	0.317	0.15	1.95E-06
Cxcl9	1.09E-07	2.068860626	0.499	0.358	0.006055
Trim30a	3.55E-09	1.968107065	0.249	0.092	0.000197
Mrap	3.21E-09	1.948518042	0.26	0.101	0.000178
Dcn	3.48E-20	1.858411899	0.89	0.749	1.93E-15
Pi15	3.08E-08	1.854346087	0.232	0.095	0.001708
Plac8	5.83E-10	1.736027135	0.355	0.211	3.23E-05
Aldh1a2	5.40E-14	1.703118782	0.433	0.205	2.99E-09
Gbp2	3.65E-08	1.652565934	0.494	0.33	0.002022
Mmp2	3.15E-07	1.637230543	0.192	0.064	0.017441
Gm12250	1.45E-07	1.604920075	0.306	0.156	0.008057
Fbln1	1.74E-13	1.563991009	0.479	0.26	9.66E-09
Uba52	9.69E-15	1.528886183	0.431	0.205	5.37E-10
Irf7	3.67E-08	1.467515	0.348	0.211	0.002033
Zbp1	7.52E-08	1.460886483	0.285	0.159	0.004166
Fndc1	1.03E-07	1.314412668	0.324	0.159	0.005714
C3	2.20E-14	1.311749386	0.751	0.547	1.22E-09
Iigp1	2.04E-08	1.31145069	0.748	0.636	0.001129
Rbp1	6.56E-08	1.216016256	0.42	0.281	0.003635
Tspo	5.25E-11	1.196258972	0.534	0.358	2.91E-06
Abca8a	1.82E-09	1.165331236	0.611	0.495	0.000101
Adamts5	3.99E-12	1.091641795	0.735	0.547	2.21E-07
Ly6e	7.48E-15	1.089515984	0.759	0.651	4.14E-10
Zc2hc1a	8.24E-07	1.068055323	0.372	0.235	0.045648
Aebp1	9.46E-09	1.035649705	0.545	0.401	0.000524
Mgp	5.19E-19	1.030053164	0.948	0.951	2.87E-14
Axl	8.22E-08	1.026925026	0.418	0.297	0.004553
Crip2	2.32E-09	0.98055575	0.541	0.364	0.000129
S100a6	4.50E-18	0.951919946	0.91	0.725	2.49E-13
Rhoj	3.23E-07	0.897356318	0.47	0.376	0.017908
S100a16	1.21E-10	0.799059934	0.713	0.56	6.73E-06
Lgals1	9.73E-10	0.684908014	0.847	0.746	5.39E-05
Enpp5	8.64E-07	0.683818255	0.613	0.483	0.04788
Mark1	4.77E-10	0.671121712	0.687	0.691	2.64E-05
Nsg1	8.07E-08	0.667185563	0.61	0.529	0.004472
Cd200	3.43E-07	0.646392861	0.635	0.578	0.019002
Rarres2	7.22E-18	0.6462804	0.991	0.957	4.00E-13
Igfbp5	1.13E-08	0.63326486	0.98	0.957	0.000624

H2-T23	1.82E-10	0.630497994	0.904	0.865	1.01E-05
Psmb9	1.67E-07	0.618302911	0.709	0.645	0.009233
Btf3	5.05E-10	0.614612704	0.764	0.67	2.80E-05
Cyba	4.21E-10	0.611174367	0.823	0.761	2.33E-05
Pmepa1	1.06E-10	0.59757695	0.869	0.85	5.88E-06
Lifr	5.70E-08	0.573227264	0.899	0.798	0.00316
Fhl2	6.10E-08	0.567187295	0.808	0.786	0.003378
Cygb	1.12E-07	0.5394617	0.855	0.789	0.006213
Tagln2	5.71E-07	0.52851599	0.843	0.771	0.031634
Cxcl14	2.02E-13	0.524436367	1	0.994	1.12E-08
Cst3	2.01E-17	0.521620025	1	1	1.11E-12
Tmed10	1.17E-07	0.493757613	0.81	0.746	0.0065
Cald1	8.78E-13	0.397853245	0.976	0.976	4.87E-08
Tmem176b	3.82E-14	0.353168577	1	1	2.12E-09
Tmsb4x	2.18E-08	0.3294946	1	1	0.001209
Lhfp	4.19E-07	0.315651887	0.884	0.893	0.02319
Epas1	1.63E-07	0.30075066	0.954	0.972	0.009034
Prkar1a	8.31E-07	0.294477839	0.853	0.884	0.046025
Ifitm3	3.43E-07	0.262620463	0.998	1	0.019017

Osteoblasts_DOWN					
**Gene**	**p_val**	**avg_log2FC**	**pct.1**	**pct.2**	**p_val_adj**
Gm48565	5.15E-15	-2.253601484	0.097	0.318	2.85E-10
Gm3336	5.45E-08	-1.878136254	0.045	0.167	0.003021
7SK.293	5.17E-07	-1.419197079	0.193	0.343	0.028651
Bglap	2.46E-29	-1.382517205	0.974	0.994	1.36E-24
Bglap2	2.39E-29	-1.29345713	0.96	0.987	1.33E-24
Iqcn	1.99E-07	-1.208576986	0.051	0.167	0.011011
Lipc	1.06E-19	-0.922499966	0.645	0.848	5.87E-15
Dab2	1.99E-07	-0.858774019	0.102	0.237	0.011038
Magi2	1.18E-07	-0.828017623	0.233	0.415	0.006535
Rsrp1	1.34E-11	-0.76208616	0.673	0.83	7.41E-07
Cpz	3.19E-16	-0.689360789	0.75	0.903	1.77E-11
Fbn2	3.94E-08	-0.688513467	0.247	0.436	0.002182
Thbs1	4.79E-09	-0.621510176	0.634	0.809	0.000265
Col11a1	8.08E-16	-0.542909575	0.974	1	4.48E-11
Col1a1	1.97E-22	-0.480712139	1	1	1.09E-17
Fhod3	1.84E-07	-0.469185937	0.48	0.669	0.010204
Dcn	4.37E-13	-0.439459169	0.986	0.996	2.42E-08
Col1a2	6.81E-22	-0.424888556	1	1	3.77E-17
Phex	2.38E-09	-0.381106465	0.517	0.723	0.000132
Col11a2	3.32E-07	-0.35939569	0.98	0.998	0.018383
Tns1	1.31E-07	-0.112788868	0.182	0.312	0.007276

Pre-Obs_DOWN					
**Gene**	**p_val**	**avg_log2FC**	**pct.1**	**pct.2**	**p_val_adj**
Pf4	1.11E-09	-3.02801975	0.019	0.112	6.17E-05
Cpz	4.02E-16	-2.676627223	0.055	0.225	2.23E-11
Ano1	2.40E-15	-2.046082543	0.085	0.278	1.33E-10
Smpd3	8.72E-13	-1.899737129	0.1	0.278	4.83E-08
Col11a2	2.12E-15	-1.804121976	0.265	0.463	1.18E-10
Bglap	3.61E-16	-1.711550082	0.807	0.924	2.00E-11
Adamts18	5.11E-09	-1.646276207	0.054	0.177	0.000283
Col1a1	6.49E-19	-1.585294894	0.972	0.992	3.59E-14
Bglap2	4.93E-14	-1.452936637	0.813	0.89	2.73E-09
Col1a2	3.64E-19	-1.452935851	0.996	1	2.02E-14
Lipc	1.62E-08	-1.350858426	0.13	0.278	0.000898
Col11a1	3.94E-17	-1.279839139	0.547	0.649	2.18E-12
Tmtc2	1.63E-10	-1.264566334	0.114	0.284	9.04E-06
Adam12	4.30E-09	-1.210331377	0.206	0.374	0.000238
Sparc	5.53E-19	-1.196431458	0.997	1	3.06E-14
Car3	8.81E-17	-1.137453011	0.622	0.728	4.88E-12
Phex	4.71E-07	-1.08818647	0.19	0.34	0.026094
Col5a2	1.69E-23	-0.97701489	0.816	0.933	9.35E-19
Serpinf1	1.45E-11	-0.94779083	0.728	0.739	8.02E-07
Lum	2.71E-11	-0.890933427	0.634	0.739	1.50E-06
Agt	3.01E-07	-0.876796961	0.323	0.472	0.016667
Col12a1	1.95E-10	-0.815385698	0.463	0.66	1.08E-05
Ccdc80	1.24E-19	-0.804677682	0.694	0.879	6.86E-15
Fat3	1.22E-12	-0.794860482	0.404	0.643	6.76E-08
Slc8a3	4.28E-08	-0.791657991	0.172	0.331	0.002369
Kdm6b	2.58E-07	-0.770224086	0.435	0.579	0.014307
Ifitm5	9.00E-07	-0.767764801	0.451	0.579	0.049891
Fn1	3.21E-07	-0.738366612	0.329	0.503	0.017766
Col22a1	1.13E-07	-0.708910713	0.442	0.61	0.006284
Cldn10	6.28E-07	-0.694345434	0.564	0.685	0.034793
Slc36a2	2.56E-09	-0.686315781	0.454	0.649	0.000142
Alpl	3.43E-08	-0.677699895	0.664	0.798	0.001898
Sh3pxd2a	2.03E-07	-0.634531192	0.474	0.64	0.011256
Col5a1	1.43E-09	-0.633638425	0.62	0.781	7.90E-05
Cpe	6.70E-13	-0.63238025	0.795	0.879	3.71E-08
Dcn	2.46E-10	-0.632282761	0.87	0.888	1.37E-05
Eny2	5.19E-08	-0.613056063	0.501	0.666	0.002877
Serpinh1	4.93E-14	-0.601019226	0.931	0.978	2.73E-09
Cdh2	2.25E-08	-0.541866727	0.649	0.778	0.001248
Top1	1.17E-07	-0.525435819	0.765	0.86	0.006473
Rab27b	9.64E-10	-0.497926456	0.117	0.25	5.34E-05
Plod2	1.06E-08	-0.472491926	0.776	0.896	0.000587
Dynll1	6.35E-07	-0.415109135	0.828	0.89	0.0352
Rrbp1	9.33E-08	-0.361842538	0.948	0.978	0.00517
Rpl41	2.97E-07	-0.332444021	0.988	0.992	0.016436

Rps29	2.90E-07	-0.330742959	0.886	0.955	0.016093

Osteo-CAR_DOWN					
**Gene**	**p_val**	**avg_log2FC**	**pct.1**	**pct.2**	**p_val_adj**
Ptprz1	1.13E-08	-1.668138464	0.13	0.347	0.000626
Abcc9	1.88E-11	-1.587438083	0.146	0.416	1.04E-06
Lum	1.73E-09	-1.418011567	0.4	0.624	9.60E-05
Bglap	4.04E-09	-1.23720784	0.485	0.733	0.000224
Cpe	7.19E-10	-1.151589164	0.363	0.614	3.98E-05
Car3	4.70E-07	-1.045498388	0.499	0.673	0.026056
Wif1	4.01E-13	-0.959696233	0.862	0.926	2.22E-08
Metrnl	1.22E-08	-0.958647469	0.442	0.668	0.000676
Hpgd	3.26E-07	-0.915921882	0.549	0.723	0.018087
Slc36a2	1.27E-07	-0.903151864	0.485	0.629	0.007061
Fign	2.38E-07	-0.872555479	0.454	0.678	0.01316
Ccdc80	5.86E-10	-0.769630389	0.735	0.847	3.25E-05
Ccdc3	8.43E-07	-0.697634512	0.451	0.678	0.046721

AdipoCAR_DOWN					
Gene	p_val	avg_log2FC	pct.1	pct.2	p_val_adj
7SK.293	6.45E-12	-3.106201435	0.028	0.162	3.58E-07
Dusp2	1.32E-13	-2.879367196	0.026	0.174	7.33E-09
Gm8251	8.87E-09	-2.669063941	0.031	0.141	0.000491
Cks2	5.64E-10	-2.274117202	0.063	0.205	3.13E-05
Pcdh10	7.94E-10	-2.258215734	0.039	0.168	4.40E-05
Hbegf	8.51E-08	-2.214271463	0.085	0.211	0.004713
Bglap	4.74E-26	-2.181492652	0.11	0.431	2.63E-21
Fmod	5.80E-09	-2.137840429	0.033	0.15	0.000321
C6	4.56E-32	-2.115054923	0.168	0.541	2.53E-27
Tuba4a	2.56E-12	-2.055606004	0.077	0.254	1.42E-07
Rbm38	1.20E-07	-1.964343524	0.035	0.141	0.006634
Arl4d	2.26E-07	-1.959214029	0.042	0.15	0.012513
Ptger3	5.79E-07	-1.820009475	0.042	0.147	0.032069
Hmox1	4.76E-11	-1.747546827	0.276	0.462	2.64E-06
Hspa2	2.56E-13	-1.745256855	0.155	0.361	1.42E-08
Bglap2	1.16E-11	-1.699825615	0.064	0.232	6.40E-07
Gm48565	6.25E-10	-1.689295235	0.12	0.294	3.46E-05
Atp6v1c2	1.15E-11	-1.654459357	0.11	0.3	6.36E-07
Nt5e	1.23E-11	-1.606974636	0.077	0.254	6.82E-07
Tob1	1.27E-19	-1.53948155	0.328	0.593	7.03E-15
Id2	7.27E-15	-1.520830271	0.223	0.48	4.03E-10
Pou3f1	1.25E-08	-1.484423685	0.11	0.269	0.00069
Tmem88	4.70E-07	-1.480876444	0.074	0.199	0.026058
Chka	1.10E-08	-1.474168177	0.142	0.303	0.000609
Plk2	1.80E-09	-1.446719196	0.098	0.263	1.00E-04
Ccl2	8.37E-10	-1.356553408	0.348	0.55	4.64E-05
Rbbp8	3.51E-07	-1.322766504	0.105	0.245	0.019452
Zbtb10	1.62E-13	-1.28274678	0.215	0.462	9.00E-09
Hexim1	1.72E-13	-1.281489773	0.413	0.602	9.55E-09
Fbxo32	1.33E-08	-1.279712647	0.21	0.385	0.000737
Cnnm1	9.90E-12	-1.277262125	0.107	0.297	5.49E-07
Rgs7bp	4.41E-18	-1.267185917	0.311	0.606	2.44E-13
Hspa1b	7.66E-11	-1.232241223	0.103	0.284	4.24E-06
Tle4	1.67E-10	-1.224971545	0.155	0.355	9.26E-06
Baz1a	7.41E-11	-1.174733785	0.306	0.498	4.11E-06
Mepce	5.72E-07	-1.14776818	0.18	0.327	0.031698
Taf1d	4.07E-10	-1.133920366	0.28	0.477	2.26E-05
Gm47283	3.29E-07	-1.10117755	0.221	0.382	0.018251
Hspa1a	5.68E-07	-1.069043857	0.166	0.324	0.031456
Sirt1	7.53E-08	-1.044745725	0.282	0.443	0.004173
Tmem62	8.79E-07	-1.02813797	0.085	0.205	0.048673
Cish	8.94E-08	-1.027059936	0.145	0.309	0.004953
Nasp	5.98E-08	-1.010486668	0.287	0.456	0.003315
Baiap2	3.12E-07	-0.99531167	0.317	0.471	0.01731
Sfrp4	1.06E-19	-0.991452397	0.731	0.911	5.87E-15

Ncald	3.90E-07	-0.979809346	0.157	0.315	0.021584
Map2k3	1.38E-10	-0.978378411	0.355	0.569	7.67E-06
Tmem178	2.29E-08	-0.978145959	0.144	0.312	0.00127
Ing2	6.97E-08	-0.974392371	0.258	0.44	0.003864
Nr1d2	2.25E-09	-0.971028109	0.313	0.498	0.000125
Itm2a	5.99E-17	-0.96657311	0.346	0.645	3.32E-12
Kdm6b	2.36E-10	-0.966137202	0.459	0.621	1.31E-05
Wsb1	8.18E-13	-0.964386833	0.545	0.691	4.53E-08
Slc38a2	1.60E-14	-0.952375717	0.692	0.823	8.88E-10
Dnajb1	3.25E-09	-0.940963121	0.519	0.694	0.00018
Ier5	5.71E-13	-0.93240292	0.68	0.801	3.16E-08
Emb	1.43E-14	-0.92816334	0.405	0.673	7.91E-10
Hnrnpa1	1.18E-08	-0.921711764	0.331	0.52	0.000651
Zfp131	4.63E-07	-0.909022969	0.313	0.465	0.025626
Chl1	3.86E-07	-0.901230352	0.308	0.471	0.02139
Ier3	1.85E-08	-0.89795578	0.587	0.734	0.001023
Ing1	3.90E-07	-0.896115984	0.35	0.514	0.021607
Twist1	8.27E-10	-0.892344042	0.394	0.606	4.58E-05
Cldn10	1.14E-15	-0.880350288	0.604	0.82	6.33E-11
Wif1	3.47E-08	-0.878477768	0.436	0.615	0.001922
Gadd45b	3.16E-14	-0.873209106	0.792	0.856	1.75E-09
Ifrd1	1.51E-08	-0.871908275	0.659	0.777	0.000836
Ppp1r10	1.14E-07	-0.861196227	0.433	0.547	0.006342
Ets1	1.95E-10	-0.857355064	0.488	0.673	1.08E-05
Gabbr2	3.21E-07	-0.856788405	0.085	0.199	0.017762
Nufip2	3.76E-13	-0.85591272	0.516	0.743	2.08E-08
Sertad2	2.53E-09	-0.850580264	0.484	0.639	0.00014
Pappa	8.85E-31	-0.849191109	0.882	0.985	4.90E-26
Slc26a7	8.12E-20	-0.846891301	0.672	0.896	4.50E-15
Marcksl1	4.87E-08	-0.829275921	0.551	0.682	0.002699
Gm17056	1.22E-08	-0.819137754	0.274	0.477	0.000676
Tob2	3.84E-08	-0.811531078	0.416	0.584	0.002127
Zbtb11	8.73E-07	-0.805640099	0.223	0.391	0.04835
Zfand5	2.34E-14	-0.804531856	0.779	0.908	1.30E-09
Foxo1	1.24E-07	-0.791370609	0.26	0.446	0.006857
Dnajb9	3.33E-07	-0.788127321	0.519	0.664	0.018432
Cdh2	2.38E-15	-0.787044773	0.536	0.789	1.32E-10
Uap1	7.21E-07	-0.781094265	0.368	0.541	0.039919
Trim8	9.43E-09	-0.778285535	0.396	0.596	0.000522
Ppp1r15a	3.29E-12	-0.773887953	0.7	0.832	1.82E-07
Grem1	1.34E-14	-0.757855287	0.799	0.948	7.42E-10
Nfkbia	2.25E-12	-0.755813697	0.888	0.945	1.24E-07
Midn	6.24E-09	-0.754788773	0.47	0.661	0.000346
Ibsp	1.89E-10	-0.749557331	0.7	0.85	1.05E-05
Rgcc	4.82E-14	-0.745864335	0.807	0.945	2.67E-09
Hsp90aa1	4.51E-10	-0.737199574	0.772	0.869	2.50E-05
Dact1	1.14E-09	-0.732499385	0.564	0.746	6.31E-05

Suco	8.95E-07	-0.727680538	0.376	0.544	0.0496
Pim1	1.18E-08	-0.722523663	0.508	0.691	0.000654
Brd2	9.68E-12	-0.721785897	0.768	0.859	5.36E-07
Eif5	2.46E-13	-0.718312172	0.77	0.878	1.36E-08
Cpeb4	1.56E-08	-0.716829292	0.455	0.648	0.000865
Wdr86	1.33E-08	-0.707189959	0.486	0.67	0.000737
Ptpn1	7.92E-09	-0.705761148	0.51	0.703	0.000439
Smad7	5.78E-10	-0.701257851	0.692	0.789	3.20E-05
Pcf11	7.68E-07	-0.700485137	0.431	0.599	0.042548
Bmp4	6.70E-12	-0.692797077	0.632	0.832	3.71E-07
Ehd3	7.41E-07	-0.641772085	0.387	0.569	0.041035
Fosl2	5.69E-09	-0.638799062	0.262	0.462	0.000315
Id4	6.46E-10	-0.632239075	0.698	0.85	3.58E-05
Adamts2	5.60E-18	-0.630136818	0.858	0.933	3.10E-13
Bmp6	8.94E-13	-0.617573811	0.718	0.89	4.95E-08
Sqstm1	5.19E-07	-0.59775919	0.716	0.78	0.028758
Azin1	6.61E-08	-0.597465386	0.503	0.694	0.003665
Med13	3.63E-08	-0.58972996	0.591	0.761	0.002011
Top1	2.01E-08	-0.578712574	0.772	0.884	0.001114
Nfkbiz	5.09E-07	-0.573430435	0.556	0.725	0.028185
Nxf1	7.37E-07	-0.569372816	0.193	0.343	0.040808
Mir703	5.55E-08	-0.567295731	0.331	0.526	0.003073
Ncl	6.13E-13	-0.555978795	0.91	0.939	3.40E-08
Tns1	2.81E-07	-0.536268472	0.396	0.587	0.015554
Adipoq	1.38E-08	-0.533239263	0.884	0.963	0.000766
Ankrd11	1.22E-09	-0.514739595	0.808	0.908	6.79E-05
Smpdl3a	8.35E-07	-0.5059353	0.545	0.719	0.046261
Clu	3.26E-13	-0.505744164	0.897	0.972	1.81E-08
Atf4	1.74E-07	-0.502131961	0.748	0.85	0.009664
Ubc	6.70E-08	-0.495134939	0.959	0.988	0.003712
Agt	6.35E-15	-0.484810375	0.818	0.966	3.52E-10
Tcf7l2	7.00E-08	-0.484742056	0.567	0.755	0.003881
Klf9	3.42E-10	-0.484321588	0.864	0.96	1.90E-05
Mpdz	1.19E-07	-0.475190461	0.777	0.884	0.006575
Ltbp1	1.50E-07	-0.471791393	0.715	0.85	0.008304
Pten	7.27E-10	-0.471563702	0.81	0.927	4.03E-05
Gdpd2	3.91E-12	-0.471028667	0.945	0.976	2.17E-07
Gas5	1.42E-08	-0.455835564	0.816	0.856	0.000784
Map1lc3b	9.13E-09	-0.438200756	0.871	0.924	0.000506
Ddx5	3.36E-11	-0.434078188	0.983	0.994	1.86E-06
Rock2	1.17E-07	-0.428872062	0.829	0.887	0.006481
Ftl1	4.42E-07	-0.407413077	0.936	0.963	0.024486
Dpep1	6.07E-08	-0.40356124	0.888	0.951	0.00336
Cebpa	8.44E-07	-0.399042139	0.746	0.878	0.046765
Rpl37a	6.00E-10	-0.39411718	0.952	0.972	3.33E-05
Calm2	2.68E-08	-0.39198296	0.936	0.96	0.001486
Rps21	1.27E-08	-0.382613994	0.89	0.969	0.000706

Selenop	6.02E-11	-0.381268754	0.993	0.991	3.34E-06
Plxna2	5.54E-09	-0.378253755	0.604	0.801	0.000307
Vegfc	1.25E-08	-0.373620323	0.777	0.92	0.000691
Gas6	7.67E-12	-0.370378918	0.978	1	4.25E-07
Rpl41	2.15E-09	-0.361756782	0.978	0.985	0.000119
Sdc2	9.56E-08	-0.340126576	0.816	0.939	0.005295
Col1a2	1.18E-10	-0.33814878	0.989	1	6.56E-06
Sparc	1.08E-11	-0.336677471	1	1	5.99E-07
Tnc	3.90E-07	-0.328449061	0.88	0.969	0.021621
Vcam1	1.30E-07	-0.323893678	0.991	0.982	0.007186
Islr	4.10E-07	-0.321224281	0.913	0.976	0.0227
Igfbp7	2.17E-09	-0.304178905	0.994	1	0.00012
Eif1	9.43E-09	-0.28661005	0.993	1	0.000522
Malat1	4.65E-07	-0.235985106	0.991	0.988	0.025754
