# Supplementary material for: The Aging Landscape by scRNAseq of Mesenchymal Lineage Cells in Mouse Bone
Source: Aging Cell. 2025 Oct 13;24(12):e70256. doi: 10.1111/acel.70256 (PMC12686594; doi:10.1111/acel.70256)
Supplement: Supplementary file 14 — Table S4: acel70256‐sup‐0014‐TableS4.pdf. [file ACEL-24-e70256-s013.pdf]

| Pre-Obs_UP | | | | | |
|---|---|---|---|---|---|
| Gene | p_val | avg_log2FC | pct.1 | pct.2 | p_val_adj |
| Msmp | 3.92E-40 | 4.305642533 | 0.289 | 0.016 | 2.17E-35 |
| Il12a | 8.05E-10 | 1.415916314 | 0.301 | 0.138 | 4.46E-05 |
| Spp1 | 1.33E-19 | 1.26547475 | 0.926 | 0.902 | 7.37E-15 |
| Enpp1 | 4.70E-08 | 0.894586435 | 0.526 | 0.369 | 0.002602633 |
| Fos | 1.76E-07 | 0.837775773 | 0.864 | 0.813 | 0.009772445 |
| Socs3 | 5.88E-08 | 0.751347585 | 0.8 | 0.707 | 0.003257461 |
| Zfp36 | 6.34E-07 | 0.572378587 | 0.681 | 0.549 | 0.035146531 |
| Ccnl1 | 1.70E-07 | 0.545407364 | 0.686 | 0.536 | 0.009413095 |
| Hspa5 | 1.36E-08 | 0.484690403 | 0.978 | 0.935 | 0.000753806 |
| Gadd45b | 8.52E-07 | 0.450151401 | 0.852 | 0.742 | 0.04721028 |

| Osteo-X_UP | | | | | |
|---|---|---|---|---|---|
| Gene | p_val | avg_log2FC | pct.1 | pct.2 | p_val_adj |
| Msmp | 1.69E-178 | 3.242140318 | 0.43 | 0.021 | 9.38E-174 |
| Gm36827 | 3.19E-13 | 1.53223948 | 0.144 | 0.063 | 1.77E-08 |
| Tagln | 5.54E-09 | 1.122442883 | 0.279 | 0.253 | 0.000307 |
| Aldh3a1 | 1.03E-11 | 1.083706368 | 0.246 | 0.149 | 5.71E-07 |
| Hpgd | 8.70E-14 | 1.079395424 | 0.267 | 0.152 | 4.82E-09 |
| Myl9 | 2.68E-20 | 1.004161061 | 0.447 | 0.319 | 1.49E-15 |
| Socs3 | 4.34E-34 | 0.913940283 | 0.766 | 0.654 | 2.41E-29 |
| Acta2 | 1.95E-11 | 0.913881722 | 0.432 | 0.369 | 1.08E-06 |
| Pim1 | 2.91E-10 | 0.869753267 | 0.263 | 0.161 | 1.61E-05 |
| Mt1 | 2.27E-19 | 0.84599845 | 0.742 | 0.686 | 1.26E-14 |
| Zfp36 | 6.27E-23 | 0.822821566 | 0.685 | 0.553 | 3.48E-18 |
| Msx1 | 1.08E-07 | 0.800584979 | 0.188 | 0.144 | 0.005977 |
| Tnfaip6 | 3.16E-13 | 0.778743574 | 0.422 | 0.314 | 1.75E-08 |
| Sertad1 | 6.01E-24 | 0.776703624 | 0.654 | 0.512 | 3.33E-19 |
| Nfil3 | 8.15E-10 | 0.770645202 | 0.303 | 0.211 | 4.52E-05 |
| Cebpd | 7.29E-23 | 0.770276395 | 0.746 | 0.658 | 4.04E-18 |
| Hspa2 | 1.89E-08 | 0.760573004 | 0.247 | 0.176 | 0.001046 |
| Atp8b1 | 6.35E-08 | 0.752128259 | 0.246 | 0.17 | 0.003517 |
| Csrp1 | 7.98E-10 | 0.749910799 | 0.341 | 0.262 | 4.42E-05 |
| Hk2 | 2.31E-11 | 0.74135605 | 0.35 | 0.25 | 1.28E-06 |
| Gadd45g | 1.84E-14 | 0.737128989 | 0.706 | 0.646 | 1.02E-09 |
| Thbs1 | 6.09E-10 | 0.734303526 | 0.46 | 0.389 | 3.37E-05 |
| Icam1 | 7.22E-08 | 0.731046472 | 0.208 | 0.186 | 0.003999 |
| Tob1 | 2.17E-17 | 0.72654944 | 0.492 | 0.373 | 1.20E-12 |
| Maff | 7.36E-08 | 0.72017752 | 0.244 | 0.154 | 0.004079 |
| Errfi1 | 4.41E-13 | 0.71389847 | 0.465 | 0.36 | 2.45E-08 |
| Gadd45a | 2.66E-09 | 0.712446392 | 0.352 | 0.281 | 0.000147 |
| Cdkn2d | 3.24E-10 | 0.695681565 | 0.372 | 0.275 | 1.80E-05 |
| Socs1 | 2.97E-09 | 0.690508395 | 0.346 | 0.264 | 0.000165 |
| Hes1 | 7.06E-10 | 0.687002217 | 0.413 | 0.335 | 3.91E-05 |
| Mt2 | 6.16E-10 | 0.667763562 | 0.443 | 0.395 | 3.41E-05 |
| Pim3 | 3.62E-07 | 0.662868545 | 0.249 | 0.165 | 0.020038 |
| Rgcc | 6.35E-12 | 0.659413967 | 0.51 | 0.454 | 3.52E-07 |
| Cebpb | 4.53E-12 | 0.640021772 | 0.598 | 0.533 | 2.51E-07 |
| Tubb6 | 8.76E-11 | 0.638594823 | 0.402 | 0.304 | 4.86E-06 |
| Ier3 | 7.98E-26 | 0.637681328 | 0.855 | 0.752 | 4.42E-21 |
| Fabp5 | 2.93E-07 | 0.636378634 | 0.104 | 0.119 | 0.016219 |
| Map1lc3a | 1.51E-25 | 0.632199923 | 0.903 | 0.849 | 8.37E-21 |
| Myc | 1.70E-07 | 0.631210592 | 0.345 | 0.285 | 0.009405 |
| Gabarapl2 | 7.79E-07 | 0.630934061 | 0.266 | 0.184 | 0.043172 |
| Atf3 | 2.28E-09 | 0.626554759 | 0.478 | 0.378 | 0.000127 |
| Sat1 | 8.12E-11 | 0.60959583 | 0.5 | 0.418 | 4.50E-06 |
| Ier5 | 8.78E-17 | 0.607445944 | 0.641 | 0.533 | 4.87E-12 |
| Gadd45b | 2.18E-19 | 0.596565263 | 0.801 | 0.702 | 1.21E-14 |
| Tuba1c | 1.65E-10 | 0.595826178 | 0.434 | 0.359 | 9.13E-06 |

| | | | | | |
|---|---|---|---|---|---|
| Gm26532 | 3.87E-07 | 0.58436937 | 0.347 | 0.268 | 0.021455 |
| Ubc | 2.11E-31 | 0.578376818 | 0.949 | 0.92 | 1.17E-26 |
| Mdk | 2.17E-16 | 0.563602575 | 0.71 | 0.602 | 1.20E-11 |
| Rasl11a | 6.49E-09 | 0.54935472 | 0.507 | 0.407 | 0.00036 |
| Klf4 | 2.17E-13 | 0.543735563 | 0.652 | 0.555 | 1.20E-08 |
| Ifrd1 | 6.04E-15 | 0.535260716 | 0.652 | 0.54 | 3.35E-10 |
| Bambi | 6.51E-07 | 0.532844824 | 0.5 | 0.442 | 0.036051 |
| Rarres2 | 1.87E-13 | 0.52836851 | 0.626 | 0.536 | 1.04E-08 |
| Ccnl1 | 5.28E-12 | 0.52759585 | 0.582 | 0.506 | 2.92E-07 |
| Wnt16 | 6.94E-12 | 0.514122602 | 0.625 | 0.512 | 3.85E-07 |
| Ier2 | 4.15E-12 | 0.50883184 | 0.701 | 0.616 | 2.30E-07 |
| Fosb | 4.64E-13 | 0.506188828 | 0.61 | 0.504 | 2.57E-08 |
| Id2 | 6.16E-19 | 0.506049269 | 0.878 | 0.842 | 3.42E-14 |
| Id1 | 2.17E-07 | 0.49796477 | 0.529 | 0.48 | 0.012031 |
| Bglap2 | 4.31E-22 | 0.496390537 | 0.988 | 0.949 | 2.39E-17 |
| Smad7 | 4.46E-07 | 0.49632426 | 0.468 | 0.403 | 0.024716 |
| Pnrc1 | 2.25E-14 | 0.492902466 | 0.685 | 0.607 | 1.25E-09 |
| Sgk1 | 4.34E-10 | 0.488171417 | 0.565 | 0.519 | 2.40E-05 |
| Tiparp | 5.92E-07 | 0.473435405 | 0.451 | 0.366 | 0.032785 |
| Sqstm1 | 1.20E-14 | 0.468181629 | 0.6 | 0.547 | 6.66E-10 |
| Herpud1 | 5.01E-10 | 0.467018883 | 0.585 | 0.511 | 2.78E-05 |
| Junb | 8.29E-16 | 0.464171788 | 0.89 | 0.865 | 4.60E-11 |
| Fos | 4.22E-10 | 0.453606012 | 0.891 | 0.872 | 2.34E-05 |
| Wsb1 | 2.71E-08 | 0.45269904 | 0.519 | 0.46 | 0.001503 |
| Ftl1 | 5.10E-19 | 0.448929015 | 0.837 | 0.791 | 2.83E-14 |
| Dapk2 | 4.56E-08 | 0.446628189 | 0.606 | 0.512 | 0.002528 |
| Ctsf | 1.90E-14 | 0.443394598 | 0.723 | 0.64 | 1.05E-09 |
| Actb | 2.50E-36 | 0.442060198 | 1 | 0.995 | 1.38E-31 |
| Nfkbia | 7.11E-12 | 0.441633098 | 0.79 | 0.717 | 3.94E-07 |
| Cdkn1c | 3.70E-13 | 0.438995256 | 0.785 | 0.721 | 2.05E-08 |
| Dnajb9 | 8.06E-08 | 0.437281938 | 0.517 | 0.445 | 0.004464 |
| Ctsl | 8.67E-36 | 0.436483129 | 0.989 | 0.957 | 4.80E-31 |
| Fbln1 | 7.17E-10 | 0.434501543 | 0.621 | 0.524 | 3.97E-05 |
| Pxdc1 | 6.62E-08 | 0.433042675 | 0.489 | 0.411 | 0.003671 |
| Tubb2a | 8.08E-08 | 0.426113906 | 0.521 | 0.464 | 0.00448 |
| Cd200 | 4.06E-08 | 0.419157596 | 0.645 | 0.604 | 0.002247 |
| Hsp90aa1 | 2.23E-12 | 0.416451926 | 0.871 | 0.822 | 1.23E-07 |
| Jund | 2.76E-22 | 0.415929106 | 0.947 | 0.925 | 1.53E-17 |
| H3f3b | 5.65E-30 | 0.415153096 | 0.991 | 0.965 | 3.13E-25 |
| Oaz1 | 2.62E-13 | 0.414988269 | 0.72 | 0.659 | 1.45E-08 |
| Ypel3 | 5.77E-08 | 0.413096813 | 0.645 | 0.571 | 0.003198 |
| Eif5 | 1.64E-16 | 0.405241019 | 0.786 | 0.739 | 9.11E-12 |
| Rsrp1 | 4.51E-16 | 0.400951764 | 0.83 | 0.79 | 2.50E-11 |
| Calm2 | 2.60E-24 | 0.397563492 | 0.974 | 0.965 | 1.44E-19 |
| Azin1 | 7.42E-07 | 0.39619723 | 0.498 | 0.434 | 0.041098 |
| Mmp13 | 1.23E-07 | 0.391873258 | 0.805 | 0.789 | 0.006821 |
| Tpm2 | 7.25E-10 | 0.37737915 | 0.85 | 0.815 | 4.02E-05 |

| | | | | | |
|---|---|---|---|---|---|
| Hspa5 | 1.82E-12 | 0.375391347 | 0.962 | 0.954 | 1.01E-07 |
| Cdkn1a | 1.70E-07 | 0.369459477 | 0.639 | 0.54 | 0.009413 |
| Ubb | 3.78E-27 | 0.365651836 | 0.978 | 0.971 | 2.10E-22 |
| Serpinf1 | 9.07E-18 | 0.363972711 | 0.993 | 0.991 | 5.02E-13 |
| Eif4a1 | 2.84E-14 | 0.362264621 | 0.734 | 0.708 | 1.57E-09 |
| Serping1 | 5.71E-18 | 0.361360325 | 0.927 | 0.896 | 3.17E-13 |
| Cnn2 | 9.66E-10 | 0.351545092 | 0.77 | 0.702 | 5.36E-05 |
| Cst3 | 2.60E-10 | 0.35018929 | 0.999 | 0.999 | 1.44E-05 |
| Gpx3 | 1.73E-28 | 0.344514946 | 0.999 | 0.998 | 9.60E-24 |
| Arl6ip5 | 2.77E-09 | 0.339108584 | 0.61 | 0.582 | 0.000154 |
| Eif1 | 3.23E-32 | 0.335409953 | 0.998 | 0.993 | 1.79E-27 |
| 1810037I1 | 1.70E-07 | 0.332856419 | 0.692 | 0.641 | 0.009395 |
| Tsc22d1 | 5.00E-11 | 0.330603914 | 0.859 | 0.795 | 2.77E-06 |
| Rap1b | 1.39E-09 | 0.322913305 | 0.695 | 0.651 | 7.71E-05 |
| Hspa8 | 3.70E-11 | 0.318723076 | 0.888 | 0.877 | 2.05E-06 |
| Map1lc3b | 6.28E-11 | 0.315256769 | 0.821 | 0.793 | 3.48E-06 |
| Atf4 | 3.59E-09 | 0.308392028 | 0.72 | 0.701 | 0.000199 |
| Gpx4 | 2.00E-07 | 0.308186627 | 0.67 | 0.62 | 0.011109 |
| Tmem176a | 3.85E-09 | 0.301593565 | 0.732 | 0.724 | 0.000213 |
| Klf9 | 2.14E-07 | 0.298289649 | 0.723 | 0.712 | 0.011884 |
| Slc38a2 | 7.87E-09 | 0.295422182 | 0.746 | 0.74 | 0.000436 |
| Cald1 | 1.92E-11 | 0.293865799 | 0.98 | 0.97 | 1.06E-06 |
| Dcn | 3.30E-15 | 0.290108598 | 1 | 0.999 | 1.83E-10 |
| Anxa1 | 8.12E-10 | 0.288426683 | 0.866 | 0.866 | 4.50E-05 |
| Dynlrb1 | 1.05E-08 | 0.279825721 | 0.862 | 0.837 | 0.000583 |
| Sdc4 | 1.85E-09 | 0.279414092 | 0.853 | 0.816 | 0.000103 |
| Fcgrt | 2.20E-08 | 0.276226159 | 0.78 | 0.748 | 0.001218 |
| Abracl | 7.20E-08 | 0.275273663 | 0.437 | 0.438 | 0.003992 |
| Ube2b | 3.44E-07 | 0.273168115 | 0.724 | 0.68 | 0.019051 |
| S100a13 | 1.48E-07 | 0.27293157 | 0.883 | 0.853 | 0.008225 |
| App | 2.08E-11 | 0.266811962 | 0.965 | 0.948 | 1.15E-06 |
| Selenop | 8.98E-07 | 0.260516119 | 0.718 | 0.631 | 0.049789 |
| Ddx5 | 9.25E-11 | 0.257393207 | 0.941 | 0.932 | 5.13E-06 |
| Fermt2 | 9.90E-09 | 0.253250534 | 0.774 | 0.783 | 0.000548 |
| Cfl1 | 2.20E-08 | 0.247104891 | 0.859 | 0.842 | 0.001217 |
| Cd302 | 2.91E-11 | 0.247034149 | 0.889 | 0.881 | 1.61E-06 |
| Itm2b | 3.00E-15 | 0.222835009 | 0.997 | 0.997 | 1.66E-10 |
| Ndfip1 | 5.05E-07 | 0.221664481 | 0.828 | 0.814 | 0.027961 |
| Dpysl3 | 1.45E-08 | 0.21948148 | 0.859 | 0.85 | 0.000802 |
| Fxyd1 | 3.52E-07 | 0.215079197 | 0.867 | 0.851 | 0.019517 |
| Txn1 | 5.12E-09 | 0.211527204 | 0.878 | 0.906 | 0.000283 |
| Dynll1 | 4.99E-07 | 0.21105036 | 0.926 | 0.92 | 0.027627 |
| Kif5b | 1.87E-07 | 0.209155851 | 0.562 | 0.578 | 0.010367 |
| Igfbp6 | 9.72E-08 | 0.195012677 | 0.783 | 0.831 | 0.005384 |
| Selenok | 5.21E-08 | 0.194455899 | 0.938 | 0.931 | 0.002885 |
| Fth1 | 9.64E-10 | 0.152545509 | 1 | 1 | 5.34E-05 |
| Rpl21 | 2.47E-07 | 0.139045912 | 1 | 0.997 | 0.013662 |

| Fibro-1_UP | | | | | |
|---|---|---|---|---|---|
| Gene | p_val | avg_log2FC | pct.1 | pct.2 | p_val_adj |
| Msmp | 4.28E-173 | 3.235757766 | 0.284 | 0.028 | 2.37E-168 |
| Gpha2 | 5.16E-16 | 1.49369298 | 0.113 | 0.054 | 2.86E-11 |
| Angptl7 | 1.38E-31 | 1.216643907 | 0.3 | 0.17 | 7.62E-27 |
| Gsta3 | 1.81E-09 | 1.14892057 | 0.102 | 0.059 | 0.0001 |
| Fgf18 | 1.67E-22 | 1.106035456 | 0.221 | 0.131 | 9.24E-18 |
| Pthlh | 1.94E-09 | 1.075797931 | 0.101 | 0.065 | 0.000108 |
| Aldh3a1 | 4.34E-21 | 0.991929687 | 0.281 | 0.202 | 2.41E-16 |
| Palmd | 1.44E-12 | 0.975026194 | 0.143 | 0.084 | 8.00E-08 |
| Wnt16 | 4.11E-19 | 0.798475412 | 0.32 | 0.242 | 2.28E-14 |
| Plk2 | 4.67E-07 | 0.793822934 | 0.117 | 0.078 | 0.025899 |
| Fosb | 7.76E-33 | 0.789972456 | 0.558 | 0.476 | 4.30E-28 |
| Prss12 | 1.38E-07 | 0.779447681 | 0.116 | 0.073 | 0.007645 |
| Maff | 1.09E-15 | 0.769050987 | 0.26 | 0.198 | 6.02E-11 |
| Mfap4 | 1.62E-19 | 0.757556465 | 0.454 | 0.355 | 8.99E-15 |
| Gm26532 | 2.25E-20 | 0.75156591 | 0.384 | 0.305 | 1.25E-15 |
| Nr4a3 | 1.32E-07 | 0.741057326 | 0.146 | 0.104 | 0.007336 |
| Chad | 5.67E-31 | 0.731016259 | 0.629 | 0.521 | 3.14E-26 |
| Penk | 1.58E-07 | 0.711102212 | 0.358 | 0.315 | 0.008743 |
| Gadd45b | 4.35E-36 | 0.672719226 | 0.734 | 0.667 | 2.41E-31 |
| Klf4 | 9.97E-39 | 0.671537324 | 0.739 | 0.652 | 5.52E-34 |
| Pim1 | 6.14E-10 | 0.667560914 | 0.207 | 0.16 | 3.40E-05 |
| Egfl6 | 6.19E-08 | 0.652770509 | 0.146 | 0.095 | 0.003428 |
| Ppp1r15a | 1.71E-33 | 0.648176589 | 0.605 | 0.496 | 9.49E-29 |
| Zfp36 | 9.92E-27 | 0.643630386 | 0.616 | 0.506 | 5.49E-22 |
| Klf2 | 4.07E-31 | 0.633966231 | 0.715 | 0.629 | 2.25E-26 |
| Nr4a1 | 1.44E-18 | 0.630677287 | 0.495 | 0.43 | 7.98E-14 |
| Hbegf | 6.38E-10 | 0.620475972 | 0.234 | 0.175 | 3.53E-05 |
| Pdgfrl | 5.91E-30 | 0.602475508 | 0.661 | 0.569 | 3.28E-25 |
| Rbp1 | 1.60E-11 | 0.602465318 | 0.298 | 0.271 | 8.88E-07 |
| Thbs1 | 1.18E-10 | 0.600347719 | 0.427 | 0.38 | 6.52E-06 |
| Socs3 | 2.70E-19 | 0.600271806 | 0.612 | 0.558 | 1.50E-14 |
| Ier3 | 2.40E-25 | 0.598622567 | 0.702 | 0.627 | 1.33E-20 |
| Ifrd1 | 4.11E-24 | 0.585077672 | 0.651 | 0.574 | 2.28E-19 |
| Ccn2 | 1.61E-18 | 0.584033215 | 0.599 | 0.537 | 8.93E-14 |
| Sord | 5.69E-09 | 0.57923448 | 0.208 | 0.158 | 0.000316 |
| Ier5 | 1.63E-21 | 0.576907846 | 0.562 | 0.478 | 9.03E-17 |
| Tob1 | 2.27E-26 | 0.575344607 | 0.54 | 0.433 | 1.26E-21 |
| Coq10b | 2.17E-12 | 0.574247937 | 0.289 | 0.231 | 1.20E-07 |
| Hspa2 | 1.74E-07 | 0.570873691 | 0.193 | 0.143 | 0.009632 |
| Nr4a2 | 8.27E-16 | 0.564009447 | 0.517 | 0.453 | 4.58E-11 |
| Crabp2 | 3.10E-09 | 0.562791771 | 0.279 | 0.209 | 0.000172 |
| Pim3 | 5.63E-11 | 0.54305956 | 0.278 | 0.203 | 3.12E-06 |
| Odc1 | 1.06E-08 | 0.542272253 | 0.215 | 0.171 | 0.000588 |
| Esr1 | 2.74E-08 | 0.531278269 | 0.235 | 0.181 | 0.001516 |
| Btg2 | 3.38E-21 | 0.524684104 | 0.642 | 0.558 | 1.87E-16 |

| | | | | | |
|---|---|---|---|---|---|
| Clu | 6.36E-07 | 0.521040931 | 0.555 | 0.522 | 0.03527 |
| Gpc3 | 5.30E-07 | 0.517718222 | 0.309 | 0.262 | 0.029387 |
| H1f2 | 8.04E-10 | 0.512523834 | 0.307 | 0.276 | 4.46E-05 |
| Fgl2 | 9.27E-15 | 0.509180098 | 0.503 | 0.418 | 5.14E-10 |
| Rasl11b | 6.34E-08 | 0.508303166 | 0.256 | 0.202 | 0.003514 |
| Spock2 | 2.20E-09 | 0.506355364 | 0.296 | 0.235 | 0.000122 |
| Lmcd1 | 6.85E-08 | 0.49722067 | 0.226 | 0.191 | 0.003794 |
| Hes1 | 1.13E-11 | 0.496835742 | 0.448 | 0.405 | 6.29E-07 |
| Sertad1 | 1.49E-17 | 0.494365306 | 0.568 | 0.497 | 8.28E-13 |
| Pnrc1 | 6.70E-28 | 0.488713091 | 0.726 | 0.645 | 3.71E-23 |
| Dnajb9 | 1.99E-17 | 0.478620722 | 0.55 | 0.468 | 1.10E-12 |
| Cilp2 | 5.38E-17 | 0.478585809 | 0.575 | 0.476 | 2.98E-12 |
| Fibin | 1.09E-09 | 0.477594835 | 0.474 | 0.422 | 6.07E-05 |
| Nufip2 | 1.86E-10 | 0.475625594 | 0.337 | 0.262 | 1.03E-05 |
| Pam | 1.96E-35 | 0.474488035 | 0.813 | 0.731 | 1.09E-30 |
| Itgbl1 | 1.46E-33 | 0.47390048 | 0.843 | 0.78 | 8.08E-29 |
| Scx | 2.00E-07 | 0.472334693 | 0.328 | 0.275 | 0.011082 |
| Cebpd | 1.69E-09 | 0.47069938 | 0.675 | 0.65 | 9.35E-05 |
| Apod | 8.80E-20 | 0.466488486 | 0.809 | 0.766 | 4.88E-15 |
| Glul | 2.63E-13 | 0.461942225 | 0.472 | 0.383 | 1.46E-08 |
| Id1 | 3.37E-12 | 0.456046834 | 0.52 | 0.449 | 1.87E-07 |
| Igfbp6 | 5.88E-35 | 0.446178642 | 0.98 | 0.972 | 3.26E-30 |
| Tenm3 | 4.52E-10 | 0.43737525 | 0.368 | 0.294 | 2.50E-05 |
| Calhm5 | 2.72E-07 | 0.435621808 | 0.285 | 0.235 | 0.015074 |
| Fos | 9.01E-16 | 0.435133126 | 0.844 | 0.837 | 4.99E-11 |
| Epha3 | 3.78E-09 | 0.429293768 | 0.362 | 0.289 | 0.000209 |
| Cdkn1b | 2.53E-08 | 0.427380153 | 0.322 | 0.256 | 0.001399 |
| Mgp | 8.82E-20 | 0.422682209 | 0.919 | 0.901 | 4.89E-15 |
| Ccnl1 | 1.01E-13 | 0.417051577 | 0.52 | 0.46 | 5.58E-09 |
| Nr1d1 | 4.72E-08 | 0.412229112 | 0.378 | 0.318 | 0.002617 |
| Rgcc | 1.33E-07 | 0.412221893 | 0.497 | 0.471 | 0.00737 |
| Nop58 | 1.60E-07 | 0.407010002 | 0.349 | 0.317 | 0.008857 |
| Fbln1 | 4.20E-19 | 0.406833997 | 0.669 | 0.597 | 2.33E-14 |
| Cdkn1c | 2.37E-21 | 0.40634661 | 0.8 | 0.732 | 1.31E-16 |
| Nfkbia | 1.74E-19 | 0.405169988 | 0.772 | 0.717 | 9.63E-15 |
| Herpud1 | 5.37E-20 | 0.39143394 | 0.669 | 0.621 | 2.97E-15 |
| Ubc | 2.14E-16 | 0.39070623 | 0.927 | 0.91 | 1.19E-11 |
| Ppp1r10 | 6.23E-07 | 0.388447601 | 0.312 | 0.262 | 0.03455 |
| Ltbp1 | 9.11E-11 | 0.386317581 | 0.52 | 0.462 | 5.05E-06 |
| Ndrg1 | 2.76E-07 | 0.384001976 | 0.359 | 0.305 | 0.015299 |
| Junb | 1.12E-13 | 0.379912163 | 0.852 | 0.837 | 6.22E-09 |
| Fmo1 | 7.38E-08 | 0.379224034 | 0.394 | 0.331 | 0.004087 |
| Skil | 1.71E-10 | 0.376622741 | 0.457 | 0.416 | 9.45E-06 |
| Dcn | 1.97E-20 | 0.37343726 | 0.997 | 0.998 | 1.09E-15 |
| Septin11 | 9.61E-09 | 0.372644963 | 0.397 | 0.339 | 0.000533 |
| Dlx5 | 6.34E-12 | 0.370298502 | 0.435 | 0.416 | 3.51E-07 |
| Col8a1 | 1.88E-15 | 0.359919406 | 0.591 | 0.488 | 1.04E-10 |

| | | | | | |
|---|---|---|---|---|---|
| Tnfrsf12a | 5.57E-08 | 0.358373323 | 0.38 | 0.364 | 0.003089 |
| Tubb2a | 2.72E-10 | 0.358161124 | 0.591 | 0.532 | 1.51E-05 |
| Col12a1 | 1.65E-11 | 0.353261817 | 0.751 | 0.718 | 9.12E-07 |
| Gnas | 4.64E-20 | 0.347287799 | 0.996 | 0.996 | 2.57E-15 |
| Sqstm1 | 1.49E-13 | 0.346215582 | 0.68 | 0.623 | 8.28E-09 |
| Sat1 | 4.17E-07 | 0.345306558 | 0.487 | 0.434 | 0.023121 |
| Mir6236 | 3.58E-19 | 0.344382557 | 0.903 | 0.853 | 1.98E-14 |
| Bhlhe40 | 8.62E-07 | 0.341368818 | 0.473 | 0.438 | 0.047745 |
| Hexim1 | 4.59E-08 | 0.338299884 | 0.442 | 0.387 | 0.002542 |
| Ddit3 | 6.93E-07 | 0.337985994 | 0.384 | 0.325 | 0.03841 |
| Snhg12 | 6.12E-07 | 0.330394281 | 0.398 | 0.36 | 0.033897 |
| Wsb1 | 9.59E-09 | 0.329706008 | 0.448 | 0.412 | 0.000532 |
| Rsrp1 | 1.53E-18 | 0.328007063 | 0.825 | 0.774 | 8.48E-14 |
| Peg3 | 2.13E-11 | 0.320423546 | 0.443 | 0.35 | 1.18E-06 |
| Csrp2 | 1.25E-09 | 0.32025626 | 0.59 | 0.532 | 6.91E-05 |
| Egr1 | 3.10E-11 | 0.310641009 | 0.776 | 0.763 | 1.72E-06 |
| Mt1 | 1.06E-07 | 0.310253732 | 0.771 | 0.801 | 0.005848 |
| Ctsl | 2.96E-26 | 0.30843669 | 0.976 | 0.973 | 1.64E-21 |
| Bnip3l | 8.84E-07 | 0.307338477 | 0.389 | 0.347 | 0.048973 |
| Map1b | 8.69E-08 | 0.302746893 | 0.637 | 0.587 | 0.004815 |
| Smad7 | 3.04E-07 | 0.300892759 | 0.49 | 0.44 | 0.01684 |
| Hspa5 | 4.71E-16 | 0.298737317 | 0.954 | 0.951 | 2.61E-11 |
| Ier2 | 2.41E-08 | 0.295774212 | 0.647 | 0.61 | 0.001338 |
| Smoc2 | 1.48E-10 | 0.295589672 | 0.715 | 0.649 | 8.20E-06 |
| Serpinf1 | 7.62E-17 | 0.293979112 | 0.976 | 0.98 | 4.22E-12 |
| Wfdc1 | 3.99E-07 | 0.293823993 | 0.465 | 0.41 | 0.022135 |
| Hspa8 | 1.32E-15 | 0.285776808 | 0.876 | 0.865 | 7.34E-11 |
| Tubb4b | 8.35E-07 | 0.28476588 | 0.467 | 0.434 | 0.046284 |
| Selenop | 2.06E-14 | 0.280884632 | 0.802 | 0.752 | 1.14E-09 |
| Svil | 2.57E-07 | 0.277809773 | 0.527 | 0.491 | 0.014228 |
| Ubb | 9.72E-25 | 0.275575063 | 0.965 | 0.954 | 5.39E-20 |
| Brd2 | 1.55E-07 | 0.2719151 | 0.623 | 0.582 | 0.008593 |
| Ftl1 | 1.54E-15 | 0.265359145 | 0.845 | 0.791 | 8.53E-11 |
| Rarres2 | 7.71E-08 | 0.265103172 | 0.699 | 0.683 | 0.004273 |
| Eif5 | 4.50E-14 | 0.264190393 | 0.801 | 0.742 | 2.49E-09 |
| Snhg8 | 1.99E-07 | 0.260913399 | 0.583 | 0.548 | 0.01103 |
| Plpp3 | 9.79E-08 | 0.258054561 | 0.669 | 0.617 | 0.005426 |
| Klf9 | 5.81E-11 | 0.256729195 | 0.794 | 0.755 | 3.22E-06 |
| H3f3b | 3.34E-14 | 0.25639386 | 0.965 | 0.954 | 1.85E-09 |
| Emb | 2.99E-07 | 0.254481803 | 0.599 | 0.54 | 0.016559 |
| Lars2 | 5.34E-07 | 0.24963968 | 0.651 | 0.612 | 0.029569 |
| 2410006H1 | 2.31E-08 | 0.245137831 | 0.731 | 0.697 | 0.00128 |
| Top1 | 6.21E-08 | 0.243796227 | 0.652 | 0.632 | 0.003444 |
| Slc38a2 | 7.56E-10 | 0.243580801 | 0.746 | 0.723 | 4.19E-05 |
| Mt2 | 2.22E-07 | 0.24245618 | 0.535 | 0.55 | 0.012301 |
| Mcl1 | 2.28E-07 | 0.236570388 | 0.656 | 0.624 | 0.01264 |
| Ptprd | 8.06E-09 | 0.236216519 | 0.747 | 0.732 | 0.000446 |

| | | | | | |
|---|---|---|---|---|---|
| Rn18s | 6.49E-16 | 0.23515563 | 1 | 1 | 3.60E-11 |
| Hnrnpa2b1 | 2.58E-09 | 0.234534328 | 0.702 | 0.666 | 0.000143 |
| Ppia | 4.21E-09 | 0.230370166 | 0.727 | 0.68 | 0.000233 |
| Tax1bp1 | 3.26E-09 | 0.21833725 | 0.712 | 0.682 | 0.000181 |
| Jund | 8.75E-08 | 0.211574322 | 0.938 | 0.931 | 0.00485 |
| Tpr | 9.99E-08 | 0.205845722 | 0.656 | 0.633 | 0.005536 |
| Eif1 | 1.67E-22 | 0.204185337 | 0.995 | 0.994 | 9.28E-18 |
| Abi3bp | 5.41E-08 | 0.194784763 | 0.948 | 0.93 | 0.002997 |
| Eef1a1 | 1.11E-08 | 0.192354135 | 0.838 | 0.806 | 0.000615 |
| Gpx3 | 6.03E-12 | 0.189856162 | 0.961 | 0.959 | 3.34E-07 |
| Map1lc3b | 8.63E-08 | 0.187655207 | 0.841 | 0.808 | 0.004782 |
| Ddx5 | 2.30E-10 | 0.18311233 | 0.941 | 0.936 | 1.27E-05 |
| Cfh | 1.24E-07 | 0.177868672 | 0.787 | 0.803 | 0.006849 |
| Dnajc3 | 2.86E-07 | 0.176989498 | 0.8 | 0.787 | 0.015871 |
| Rpl10 | 1.21E-11 | 0.143691553 | 0.996 | 0.995 | 6.70E-07 |
| Selenok | 6.24E-08 | 0.120640959 | 0.925 | 0.93 | 0.003457 |
| Rpl17 | 2.64E-08 | 0.116625774 | 0.996 | 0.991 | 0.001466 |

| Fibro-2_UP | | | | | |
|---|---|---|---|---|---|
| Gene | p_val | avg_log2FC | pct.1 | pct.2 | p_val_adj |
| Msmp | 0 | 4.811664 | 0.334 | 0.021 | 0 |
| Cxcl1 | 2.77E-42 | 1.191694 | 0.335 | 0.271 | 1.54E-37 |
| Ccl7 | 9.87E-14 | 1.02056 | 0.192 | 0.187 | 5.47E-09 |
| Aldh3a1 | 2.18E-25 | 0.975446 | 0.13 | 0.083 | 1.21E-20 |
| Sfrp2 | 1.64E-14 | 0.782756 | 0.192 | 0.149 | 9.09E-10 |
| Tnfaip6 | 7.74E-29 | 0.734515 | 0.389 | 0.387 | 4.29E-24 |
| Il6 | 7.57E-10 | 0.734051 | 0.12 | 0.093 | 4.19E-05 |
| Irf1 | 6.10E-12 | 0.720782 | 0.104 | 0.072 | 3.38E-07 |
| Kdm6b | 4.53E-47 | 0.707259 | 0.357 | 0.301 | 2.51E-42 |
| Hspa2 | 3.11E-21 | 0.697602 | 0.156 | 0.116 | 1.72E-16 |
| Fosb | 5.05E-48 | 0.69674 | 0.472 | 0.404 | 2.80E-43 |
| Plau | 4.84E-24 | 0.685742 | 0.235 | 0.2 | 2.68E-19 |
| Tgif1 | 1.98E-21 | 0.681707 | 0.164 | 0.125 | 1.10E-16 |
| Gpc3 | 7.75E-69 | 0.676627 | 0.457 | 0.313 | 4.29E-64 |
| Ccl2 | 1.17E-07 | 0.672503 | 0.131 | 0.134 | 0.006481 |
| Tnfsf9 | 2.02E-18 | 0.653882 | 0.186 | 0.144 | 1.12E-13 |
| Ppp1r15a | 1.39E-66 | 0.652707 | 0.552 | 0.46 | 7.70E-62 |
| Ier5 | 2.21E-50 | 0.642449 | 0.513 | 0.437 | 1.22E-45 |
| Zfp36 | 1.06E-52 | 0.637986 | 0.591 | 0.511 | 5.87E-48 |
| Mt2 | 1.26E-46 | 0.635158 | 0.735 | 0.728 | 7.00E-42 |
| Gadd45b | 4.43E-49 | 0.63375 | 0.53 | 0.462 | 2.46E-44 |
| Peg3 | 5.16E-17 | 0.63196 | 0.188 | 0.14 | 2.86E-12 |
| Lsamp | 7.32E-24 | 0.630346 | 0.21 | 0.143 | 4.06E-19 |
| Hes1 | 3.47E-40 | 0.625487 | 0.394 | 0.358 | 1.92E-35 |
| Adamts1 | 1.57E-39 | 0.620617 | 0.443 | 0.397 | 8.70E-35 |
| Btg2 | 6.66E-52 | 0.618563 | 0.542 | 0.455 | 3.69E-47 |
| Pim3 | 3.62E-20 | 0.601876 | 0.196 | 0.152 | 2.01E-15 |
| Gsn | 4.41E-198 | 0.597267 | 1 | 0.999 | 2.44E-193 |
| Fgl2 | 9.95E-35 | 0.583903 | 0.507 | 0.419 | 5.51E-30 |
| Ptgs2 | 1.05E-07 | 0.582346 | 0.134 | 0.118 | 0.005846 |
| Hk2 | 3.49E-26 | 0.575709 | 0.278 | 0.24 | 1.93E-21 |
| Myc | 2.32E-27 | 0.57109 | 0.325 | 0.262 | 1.28E-22 |
| Socs3 | 4.07E-47 | 0.561474 | 0.603 | 0.568 | 2.25E-42 |
| Icam1 | 2.03E-24 | 0.551746 | 0.367 | 0.315 | 1.13E-19 |
| Meg3 | 6.33E-10 | 0.54462 | 0.108 | 0.083 | 3.51E-05 |
| Socs1 | 2.01E-25 | 0.544094 | 0.253 | 0.215 | 1.11E-20 |
| Ier3 | 5.65E-42 | 0.541532 | 0.564 | 0.521 | 3.13E-37 |
| Arid5a | 1.01E-11 | 0.538771 | 0.112 | 0.093 | 5.59E-07 |
| Has1 | 3.01E-33 | 0.529387 | 0.344 | 0.373 | 1.67E-28 |
| Smoc2 | 6.35E-19 | 0.525618 | 0.353 | 0.33 | 3.52E-14 |
| Cebpd | 1.38E-32 | 0.518564 | 0.695 | 0.673 | 7.63E-28 |
| Rad51b | 1.17E-09 | 0.515618 | 0.107 | 0.084 | 6.48E-05 |
| Nfkbia | 4.80E-59 | 0.514892 | 0.736 | 0.695 | 2.66E-54 |
| Junb | 4.85E-53 | 0.504401 | 0.81 | 0.775 | 2.69E-48 |
| Ifrd1 | 1.34E-36 | 0.502489 | 0.581 | 0.553 | 7.41E-32 |

| | | | | | |
|---|---|---|---|---|---|
| Scg3 | 6.36E-16 | 0.498529 | 0.267 | 0.221 | 3.53E-11 |
| Bambi | 2.39E-15 | 0.495667 | 0.205 | 0.181 | 1.33E-10 |
| Fst | 4.49E-28 | 0.495322 | 0.341 | 0.257 | 2.49E-23 |
| Map3k8 | 3.59E-15 | 0.492357 | 0.172 | 0.144 | 1.99E-10 |
| Ppp1r10 | 8.77E-21 | 0.484763 | 0.243 | 0.206 | 4.86E-16 |
| Penk | 5.14E-20 | 0.483676 | 0.301 | 0.238 | 2.85E-15 |
| Mt1 | 4.30E-27 | 0.481536 | 0.912 | 0.913 | 2.38E-22 |
| Dkk2 | 8.00E-18 | 0.473547 | 0.3 | 0.24 | 4.43E-13 |
| Ptgir | 3.06E-14 | 0.470101 | 0.148 | 0.13 | 1.70E-09 |
| Ptx3 | 2.11E-11 | 0.468115 | 0.112 | 0.129 | 1.17E-06 |
| Rasl11a | 1.38E-08 | 0.462005 | 0.141 | 0.116 | 0.000766 |
| Tle4 | 6.36E-12 | 0.461387 | 0.141 | 0.12 | 3.53E-07 |
| Wsb1 | 4.68E-34 | 0.458212 | 0.408 | 0.365 | 2.59E-29 |
| Tmeff2 | 3.75E-14 | 0.449292 | 0.202 | 0.174 | 2.08E-09 |
| Negr1 | 8.82E-07 | 0.448155 | 0.112 | 0.089 | 0.048861 |
| Pnrc1 | 4.12E-62 | 0.442375 | 0.72 | 0.678 | 2.28E-57 |
| Meox1 | 1.83E-07 | 0.441047 | 0.146 | 0.124 | 0.010152 |
| Ddit3 | 1.46E-24 | 0.44083 | 0.335 | 0.293 | 8.08E-20 |
| Sertad1 | 6.29E-34 | 0.433537 | 0.464 | 0.427 | 3.48E-29 |
| Gm13588 | 1.80E-12 | 0.433037 | 0.175 | 0.148 | 9.95E-08 |
| Esr1 | 1.32E-11 | 0.429298 | 0.154 | 0.133 | 7.34E-07 |
| Id1 | 1.45E-13 | 0.425739 | 0.225 | 0.204 | 8.05E-09 |
| Cdkn2aip | 2.00E-10 | 0.42045 | 0.15 | 0.128 | 1.11E-05 |
| Fos | 2.24E-35 | 0.41569 | 0.761 | 0.771 | 1.24E-30 |
| Calml4 | 3.51E-15 | 0.415356 | 0.203 | 0.178 | 1.94E-10 |
| Sat1 | 7.68E-32 | 0.41513 | 0.52 | 0.496 | 4.25E-27 |
| Bmp4 | 8.77E-14 | 0.411375 | 0.249 | 0.216 | 4.86E-09 |
| Gadd45g | 1.85E-24 | 0.41135 | 0.572 | 0.561 | 1.02E-19 |
| Ccnl1 | 2.24E-37 | 0.408347 | 0.536 | 0.497 | 1.24E-32 |
| Tiparp | 1.68E-22 | 0.40703 | 0.388 | 0.35 | 9.29E-18 |
| Abca8a | 1.28E-34 | 0.405922 | 0.619 | 0.544 | 7.11E-30 |
| Pim1 | 1.94E-09 | 0.401271 | 0.171 | 0.144 | 0.000107 |
| Hexim1 | 1.09E-25 | 0.40117 | 0.363 | 0.323 | 6.02E-21 |
| Cytl1 | 2.75E-17 | 0.400619 | 0.248 | 0.266 | 1.52E-12 |
| Rabgef1 | 3.21E-10 | 0.40046 | 0.131 | 0.117 | 1.78E-05 |
| Ripk1 | 1.05E-19 | 0.39892 | 0.226 | 0.21 | 5.82E-15 |
| Nop58 | 3.03E-20 | 0.396714 | 0.329 | 0.303 | 1.68E-15 |
| Maml3 | 9.77E-10 | 0.394684 | 0.139 | 0.122 | 5.41E-05 |
| Procr | 5.81E-22 | 0.393678 | 0.359 | 0.342 | 3.22E-17 |
| Pik3ip1 | 6.13E-14 | 0.392245 | 0.196 | 0.174 | 3.40E-09 |
| Klhl21 | 1.81E-13 | 0.390718 | 0.131 | 0.123 | 1.01E-08 |
| Ier2 | 9.83E-33 | 0.390367 | 0.587 | 0.548 | 5.45E-28 |
| Egr1 | 1.96E-29 | 0.387897 | 0.682 | 0.672 | 1.09E-24 |
| 2900041M | 1.40E-12 | 0.386911 | 0.128 | 0.119 | 7.74E-08 |
| Txnip | 2.18E-52 | 0.386404 | 0.796 | 0.802 | 1.21E-47 |
| Taf7 | 2.33E-09 | 0.384736 | 0.16 | 0.138 | 0.000129 |
| Mknk2 | 3.92E-22 | 0.383081 | 0.297 | 0.269 | 2.17E-17 |

| | | | | | |
|---|---|---|---|---|---|
| Irs2 | 5.07E-15 | 0.380249 | 0.233 | 0.216 | 2.81E-10 |
| Apod | 7.49E-22 | 0.371398 | 0.793 | 0.779 | 4.15E-17 |
| Cdkn1a | 1.86E-37 | 0.370505 | 0.718 | 0.664 | 1.03E-32 |
| Kif21a | 4.21E-10 | 0.370195 | 0.143 | 0.127 | 2.33E-05 |
| Gm26532 | 2.14E-20 | 0.369322 | 0.298 | 0.282 | 1.19E-15 |
| Ophn1 | 4.64E-14 | 0.368256 | 0.184 | 0.165 | 2.57E-09 |
| Akap12 | 1.02E-19 | 0.367976 | 0.384 | 0.354 | 5.66E-15 |
| Myoc | 1.15E-34 | 0.367939 | 0.76 | 0.725 | 6.35E-30 |
| Cbx4 | 3.43E-08 | 0.365968 | 0.132 | 0.116 | 0.001903 |
| Nup98 | 6.20E-08 | 0.364007 | 0.14 | 0.121 | 0.003438 |
| Trib1 | 3.55E-12 | 0.362287 | 0.162 | 0.157 | 1.97E-07 |
| Bclaf3 | 9.92E-09 | 0.361517 | 0.123 | 0.111 | 0.00055 |
| Mturn | 4.88E-11 | 0.361135 | 0.153 | 0.138 | 2.71E-06 |
| Rps27rt | 1.81E-08 | 0.361013 | 0.135 | 0.117 | 0.001003 |
| Nasp | 2.68E-26 | 0.360684 | 0.273 | 0.261 | 1.48E-21 |
| Atf3 | 2.43E-18 | 0.360039 | 0.433 | 0.412 | 1.34E-13 |
| Spry2 | 2.11E-17 | 0.355868 | 0.248 | 0.231 | 1.17E-12 |
| Mafk | 1.09E-19 | 0.355611 | 0.225 | 0.214 | 6.03E-15 |
| Ifi207 | 3.09E-18 | 0.355478 | 0.407 | 0.374 | 1.71E-13 |
| Gstm1 | 5.75E-50 | 0.349453 | 0.848 | 0.81 | 3.19E-45 |
| Ndrg1 | 9.43E-23 | 0.348452 | 0.548 | 0.538 | 5.23E-18 |
| Nr4a1 | 1.42E-19 | 0.345128 | 0.427 | 0.413 | 7.88E-15 |
| Bdh2 | 1.48E-13 | 0.344358 | 0.259 | 0.23 | 8.22E-09 |
| Ccng2 | 5.20E-08 | 0.344306 | 0.117 | 0.105 | 0.002883 |
| Fgf18 | 3.65E-16 | 0.343256 | 0.262 | 0.245 | 2.02E-11 |
| Podn | 1.56E-12 | 0.34299 | 0.264 | 0.231 | 8.66E-08 |
| Acvr2a | 8.05E-10 | 0.342511 | 0.234 | 0.206 | 4.46E-05 |
| Tob1 | 1.26E-25 | 0.341731 | 0.47 | 0.438 | 6.98E-21 |
| Pdgfrl | 7.32E-12 | 0.340753 | 0.18 | 0.178 | 4.05E-07 |
| Brd2 | 1.40E-35 | 0.340387 | 0.557 | 0.53 | 7.76E-31 |
| Litaf | 3.01E-30 | 0.340158 | 0.391 | 0.381 | 1.67E-25 |
| Slc41a1 | 9.03E-18 | 0.339725 | 0.192 | 0.192 | 5.00E-13 |
| Fbxo30 | 4.27E-13 | 0.339654 | 0.185 | 0.172 | 2.37E-08 |
| Slc38a2 | 1.16E-38 | 0.339325 | 0.671 | 0.657 | 6.43E-34 |
| Jpt1 | 1.01E-30 | 0.337891 | 0.511 | 0.484 | 5.59E-26 |
| Tuba1c | 4.11E-22 | 0.335741 | 0.211 | 0.211 | 2.28E-17 |
| Vcam1 | 3.96E-11 | 0.335417 | 0.198 | 0.217 | 2.20E-06 |
| Cdc42ep4 | 1.57E-09 | 0.333423 | 0.155 | 0.142 | 8.70E-05 |
| Baz1a | 3.42E-13 | 0.331987 | 0.129 | 0.127 | 1.89E-08 |
| Hspa8 | 1.21E-42 | 0.33173 | 0.895 | 0.88 | 6.73E-38 |
| Syne3 | 1.98E-09 | 0.330165 | 0.128 | 0.119 | 0.00011 |
| Maff | 4.59E-17 | 0.32983 | 0.325 | 0.313 | 2.54E-12 |
| Crlf1 | 7.29E-09 | 0.328977 | 0.2 | 0.185 | 0.000404 |
| Sparcl1 | 3.07E-19 | 0.32883 | 0.464 | 0.46 | 1.70E-14 |
| F3 | 8.74E-07 | 0.327181 | 0.201 | 0.167 | 0.048419 |
| Tmsb10 | 9.65E-55 | 0.326177 | 0.906 | 0.88 | 5.35E-50 |
| Plaur | 7.99E-11 | 0.325967 | 0.133 | 0.131 | 4.43E-06 |

| | | | | | |
|---|---|---|---|---|---|
| Rps10-ps1 | 1.42E-11 | 0.325225 | 0.12 | 0.114 | 7.88E-07 |
| Rsrp1 | 1.60E-47 | 0.325153 | 0.816 | 0.783 | 8.86E-43 |
| Angpt1 | 2.30E-08 | 0.324779 | 0.242 | 0.215 | 0.001276 |
| Plcb4 | 2.38E-08 | 0.322929 | 0.12 | 0.112 | 0.001318 |
| Sirt1 | 5.82E-10 | 0.322549 | 0.179 | 0.162 | 3.23E-05 |
| Nfkbiz | 1.77E-15 | 0.321282 | 0.281 | 0.269 | 9.78E-11 |
| Glt8d2 | 1.34E-10 | 0.321042 | 0.16 | 0.148 | 7.42E-06 |
| Ccl11 | 4.02E-07 | 0.319988 | 0.266 | 0.24 | 0.022258 |
| Gls | 2.71E-27 | 0.317789 | 0.399 | 0.379 | 1.50E-22 |
| Tpbg | 2.31E-17 | 0.317718 | 0.186 | 0.184 | 1.28E-12 |
| Jam2 | 1.41E-11 | 0.317176 | 0.213 | 0.196 | 7.84E-07 |
| Yod1 | 2.31E-10 | 0.316855 | 0.118 | 0.117 | 1.28E-05 |
| Gstt1 | 4.24E-17 | 0.315827 | 0.371 | 0.336 | 2.35E-12 |
| Bcl3 | 4.84E-09 | 0.315804 | 0.154 | 0.145 | 0.000268 |
| Cdk12 | 1.38E-16 | 0.313365 | 0.229 | 0.217 | 7.65E-12 |
| Ubald2 | 4.08E-09 | 0.313324 | 0.122 | 0.113 | 0.000226 |
| Zbtb10 | 8.65E-10 | 0.312492 | 0.143 | 0.135 | 4.79E-05 |
| Rarres2 | 3.50E-43 | 0.312397 | 0.897 | 0.879 | 1.94E-38 |
| Sp3os | 2.28E-11 | 0.312071 | 0.16 | 0.149 | 1.27E-06 |
| Ifitm2 | 4.78E-36 | 0.311167 | 0.627 | 0.588 | 2.65E-31 |
| Nufip2 | 3.99E-13 | 0.310399 | 0.24 | 0.221 | 2.21E-08 |
| Zfp395 | 3.58E-07 | 0.310101 | 0.154 | 0.139 | 0.019852 |
| Lama2 | 1.38E-21 | 0.31005 | 0.375 | 0.359 | 7.64E-17 |
| Coq10b | 3.35E-18 | 0.308665 | 0.277 | 0.267 | 1.86E-13 |
| Cpeb2 | 3.09E-11 | 0.308557 | 0.116 | 0.114 | 1.71E-06 |
| Rpl9-ps6 | 4.61E-09 | 0.30748 | 0.132 | 0.122 | 0.000256 |
| Cebpb | 2.51E-17 | 0.30647 | 0.611 | 0.599 | 1.39E-12 |
| Lysmd2 | 5.29E-27 | 0.304879 | 0.601 | 0.571 | 2.93E-22 |
| Midn | 1.32E-16 | 0.302487 | 0.285 | 0.27 | 7.34E-12 |
| Ing1 | 3.32E-14 | 0.299336 | 0.243 | 0.228 | 1.84E-09 |
| Trip10 | 1.26E-23 | 0.296682 | 0.411 | 0.389 | 6.98E-19 |
| Pvr | 2.01E-14 | 0.296623 | 0.112 | 0.114 | 1.11E-09 |
| Pcf11 | 8.42E-15 | 0.295442 | 0.299 | 0.279 | 4.67E-10 |
| Klf2 | 7.70E-20 | 0.29483 | 0.75 | 0.713 | 4.27E-15 |
| Hnrnpa1 | 1.18E-10 | 0.293763 | 0.225 | 0.209 | 6.53E-06 |
| Dnajb9 | 9.03E-29 | 0.292793 | 0.454 | 0.448 | 5.00E-24 |
| Ms4a4d | 2.80E-07 | 0.291695 | 0.226 | 0.194 | 0.015494 |
| Arhgap10 | 3.68E-23 | 0.291292 | 0.345 | 0.33 | 2.04E-18 |
| Sqstm1 | 1.84E-34 | 0.290275 | 0.673 | 0.66 | 1.02E-29 |
| Zfp36l2 | 1.45E-19 | 0.289854 | 0.482 | 0.45 | 8.03E-15 |
| Nop14 | 9.43E-16 | 0.289136 | 0.243 | 0.24 | 5.22E-11 |
| Txnrd1 | 5.51E-18 | 0.288841 | 0.294 | 0.289 | 3.05E-13 |
| Clec3b | 1.66E-37 | 0.288345 | 0.907 | 0.877 | 9.22E-33 |
| Dpp4 | 8.97E-15 | 0.28746 | 0.314 | 0.307 | 4.97E-10 |
| Kin | 5.26E-16 | 0.286314 | 0.154 | 0.155 | 2.91E-11 |
| Id2 | 5.30E-17 | 0.286168 | 0.535 | 0.513 | 2.94E-12 |
| Btg1 | 4.95E-15 | 0.286067 | 0.381 | 0.361 | 2.75E-10 |

| | | | | | |
|---|---|---|---|---|---|
| Mepce | 3.27E-07 | 0.285652 | 0.149 | 0.138 | 0.018105 |
| Adamtsl3 | 2.93E-10 | 0.283732 | 0.248 | 0.231 | 1.62E-05 |
| Tamalin | 5.72E-08 | 0.281532 | 0.208 | 0.189 | 0.003171 |
| Ppl | 4.69E-10 | 0.281481 | 0.29 | 0.26 | 2.60E-05 |
| Nr4a2 | 1.06E-11 | 0.281464 | 0.308 | 0.302 | 5.88E-07 |
| Agmo | 6.98E-12 | 0.280988 | 0.107 | 0.107 | 3.87E-07 |
| Rap2b | 5.04E-14 | 0.277918 | 0.18 | 0.172 | 2.79E-09 |
| Odc1 | 4.98E-09 | 0.27791 | 0.121 | 0.118 | 0.000276 |
| Gm37376 | 3.50E-08 | 0.276878 | 0.226 | 0.222 | 0.001937 |
| Dynlt1f | 5.16E-11 | 0.276408 | 0.12 | 0.117 | 2.86E-06 |
| Gfpt2 | 1.09E-15 | 0.276011 | 0.509 | 0.507 | 6.05E-11 |
| Dot1l | 7.55E-13 | 0.274097 | 0.122 | 0.124 | 4.18E-08 |
| U2af1 | 1.99E-17 | 0.273495 | 0.289 | 0.273 | 1.11E-12 |
| Ubb | 3.97E-57 | 0.273072 | 0.949 | 0.949 | 2.20E-52 |
| Gpx3 | 6.54E-33 | 0.272178 | 0.833 | 0.808 | 3.63E-28 |
| Phlda1 | 5.38E-12 | 0.271277 | 0.539 | 0.513 | 2.98E-07 |
| Sfpq | 4.29E-14 | 0.271216 | 0.284 | 0.267 | 2.38E-09 |
| Nosip | 1.58E-12 | 0.270551 | 0.137 | 0.136 | 8.75E-08 |
| Glul | 6.31E-14 | 0.270123 | 0.503 | 0.491 | 3.50E-09 |
| Phf13 | 4.23E-08 | 0.26946 | 0.129 | 0.122 | 0.002346 |
| Fbln1 | 2.03E-19 | 0.269286 | 0.549 | 0.526 | 1.12E-14 |
| Hivep1 | 6.08E-07 | 0.26877 | 0.13 | 0.123 | 0.033694 |
| H3f3b | 1.59E-40 | 0.268058 | 0.929 | 0.932 | 8.82E-36 |
| Tlcd2 | 2.92E-07 | 0.267732 | 0.131 | 0.125 | 0.016162 |
| Ogfrl1 | 1.80E-08 | 0.266628 | 0.167 | 0.16 | 0.000995 |
| Taf1d | 3.62E-09 | 0.266556 | 0.174 | 0.165 | 0.000201 |
| Smarcc2 | 1.80E-07 | 0.264943 | 0.106 | 0.103 | 0.009989 |
| Tmem140 | 7.84E-11 | 0.264438 | 0.195 | 0.186 | 4.35E-06 |
| Jup | 7.05E-11 | 0.264261 | 0.206 | 0.196 | 3.91E-06 |
| Uap1 | 3.06E-12 | 0.262792 | 0.644 | 0.643 | 1.70E-07 |
| Bmyc | 3.99E-09 | 0.262076 | 0.227 | 0.213 | 0.000221 |
| Gm9843 | 2.33E-12 | 0.261728 | 0.239 | 0.224 | 1.29E-07 |
| Jund | 1.30E-44 | 0.261224 | 0.985 | 0.98 | 7.21E-40 |
| Pink1 | 8.23E-23 | 0.260964 | 0.439 | 0.419 | 4.56E-18 |
| Cited2 | 3.58E-11 | 0.260761 | 0.319 | 0.303 | 1.98E-06 |
| 2410006H1 | 3.94E-28 | 0.259845 | 0.64 | 0.62 | 2.19E-23 |
| Slc66a2 | 1.48E-09 | 0.259604 | 0.148 | 0.148 | 8.20E-05 |
| Ets1 | 1.11E-10 | 0.25891 | 0.168 | 0.164 | 6.13E-06 |
| Eif1 | 4.96E-76 | 0.258456 | 0.996 | 0.995 | 2.75E-71 |
| Akt2 | 6.66E-08 | 0.257857 | 0.113 | 0.109 | 0.00369 |
| Gramd3 | 7.07E-14 | 0.257832 | 0.102 | 0.105 | 3.92E-09 |
| Asap2 | 2.37E-13 | 0.256782 | 0.235 | 0.227 | 1.31E-08 |
| Nfkb2 | 4.81E-12 | 0.255855 | 0.173 | 0.169 | 2.66E-07 |
| Hnrnph3 | 1.82E-14 | 0.254463 | 0.259 | 0.25 | 1.01E-09 |
| Xaf1 | 6.99E-10 | 0.253652 | 0.106 | 0.108 | 3.88E-05 |
| Rpl21 | 5.61E-87 | 0.253454 | 0.998 | 0.998 | 3.11E-82 |
| Gem | 3.84E-15 | 0.251819 | 0.606 | 0.594 | 2.13E-10 |

| | | | | | |
|---|---|---|---|---|---|
| Rpl13 | 7.47E-91 | 0.251606 | 0.999 | 0.998 | 4.14E-86 |
| Abca8b | 1.35E-13 | 0.251564 | 0.447 | 0.414 | 7.49E-09 |
| Qpct | 5.96E-11 | 0.251306 | 0.32 | 0.301 | 3.30E-06 |
| Apbb2 | 1.96E-15 | 0.25051 | 0.239 | 0.233 | 1.08E-10 |
| Snu13 | 2.76E-22 | 0.250174 | 0.304 | 0.305 | 1.53E-17 |
| Mill2 | 2.01E-14 | 0.247678 | 0.19 | 0.187 | 1.11E-09 |
| Mustn1 | 7.97E-15 | 0.24664 | 0.555 | 0.536 | 4.42E-10 |
| Atf4 | 1.85E-29 | 0.246018 | 0.718 | 0.713 | 1.02E-24 |
| Rel | 8.15E-09 | 0.245993 | 0.108 | 0.111 | 0.000451 |
| Ifi203 | 1.92E-07 | 0.245316 | 0.162 | 0.163 | 0.010645 |
| Dleu2 | 1.18E-10 | 0.244934 | 0.194 | 0.189 | 6.53E-06 |
| Pcdh18 | 1.52E-08 | 0.244109 | 0.22 | 0.205 | 0.000843 |
| Fam111a | 1.51E-10 | 0.243643 | 0.167 | 0.165 | 8.38E-06 |
| Il17ra | 2.08E-07 | 0.243293 | 0.119 | 0.117 | 0.011542 |
| Lrrn4cl | 1.66E-22 | 0.242854 | 0.528 | 0.517 | 9.21E-18 |
| Rpl17 | 3.40E-73 | 0.242383 | 0.996 | 0.993 | 1.89E-68 |
| Tut4 | 2.34E-11 | 0.24082 | 0.281 | 0.269 | 1.30E-06 |
| Luc7l | 1.11E-08 | 0.240787 | 0.177 | 0.169 | 0.000614 |
| Abca9 | 7.01E-07 | 0.239085 | 0.283 | 0.258 | 0.038845 |
| Rps4l | 7.86E-15 | 0.239081 | 0.225 | 0.227 | 4.36E-10 |
| Rpl10 | 1.34E-89 | 0.238786 | 0.994 | 0.995 | 7.45E-85 |
| Rarg | 1.23E-21 | 0.238238 | 0.366 | 0.36 | 6.81E-17 |
| Gm28875 | 1.55E-15 | 0.238151 | 0.255 | 0.251 | 8.60E-11 |
| Xdh | 4.97E-13 | 0.238051 | 0.341 | 0.324 | 2.75E-08 |
| Prkx | 1.32E-09 | 0.238049 | 0.133 | 0.132 | 7.33E-05 |
| Arhgap20 | 8.62E-11 | 0.237814 | 0.296 | 0.277 | 4.78E-06 |
| Pdlim2 | 9.09E-22 | 0.237533 | 0.638 | 0.624 | 5.04E-17 |
| Ltbp4 | 2.26E-21 | 0.237316 | 0.868 | 0.836 | 1.25E-16 |
| Fsd1l | 1.88E-08 | 0.236613 | 0.126 | 0.122 | 0.00104 |
| Pcolce2 | 6.90E-23 | 0.23583 | 0.791 | 0.774 | 3.83E-18 |
| Fas | 2.88E-12 | 0.235299 | 0.173 | 0.174 | 1.59E-07 |
| Rps6 | 4.76E-39 | 0.2351 | 0.82 | 0.812 | 2.64E-34 |
| Ndn | 5.85E-14 | 0.23407 | 0.522 | 0.477 | 3.24E-09 |
| Ppp2r2a | 1.74E-10 | 0.233338 | 0.164 | 0.162 | 9.64E-06 |
| Arid4a | 3.76E-14 | 0.233266 | 0.254 | 0.25 | 2.08E-09 |
| Mpp5 | 3.56E-16 | 0.233157 | 0.16 | 0.166 | 1.97E-11 |
| Gnl3 | 2.49E-17 | 0.232196 | 0.21 | 0.213 | 1.38E-12 |
| Rpl6 | 5.29E-46 | 0.232127 | 0.971 | 0.965 | 2.93E-41 |
| Stim1 | 6.72E-16 | 0.231488 | 0.203 | 0.204 | 3.72E-11 |
| Rap2a | 8.88E-14 | 0.230625 | 0.341 | 0.33 | 4.92E-09 |
| Psip1 | 4.56E-16 | 0.230495 | 0.357 | 0.351 | 2.53E-11 |
| Ugdh | 6.23E-10 | 0.230382 | 0.609 | 0.602 | 3.46E-05 |
| Klf4 | 2.72E-18 | 0.230019 | 0.862 | 0.842 | 1.51E-13 |
| Mgp | 1.19E-11 | 0.229012 | 0.861 | 0.851 | 6.59E-07 |
| Srsf2 | 4.03E-27 | 0.228338 | 0.604 | 0.599 | 2.23E-22 |
| Snhg8 | 4.37E-22 | 0.228291 | 0.532 | 0.52 | 2.42E-17 |
| Palm | 1.17E-16 | 0.226983 | 0.338 | 0.33 | 6.51E-12 |

| | | | | | |
|---|---|---|---|---|---|
| Septin10 | 1.97E-07 | 0.226629 | 0.123 | 0.12 | 0.010893 |
| Gnb4 | 4.13E-13 | 0.226549 | 0.269 | 0.263 | 2.29E-08 |
| Anp32a | 7.41E-20 | 0.225799 | 0.438 | 0.426 | 4.11E-15 |
| Pir | 3.28E-14 | 0.225579 | 0.249 | 0.245 | 1.82E-09 |
| Snhg1 | 1.18E-22 | 0.224574 | 0.393 | 0.401 | 6.56E-18 |
| Irf2 | 4.31E-12 | 0.224434 | 0.238 | 0.23 | 2.39E-07 |
| Hsp90aa1 | 2.21E-13 | 0.223413 | 0.819 | 0.819 | 1.23E-08 |
| Arl13b | 1.10E-13 | 0.222664 | 0.309 | 0.302 | 6.10E-09 |
| Usp46 | 1.59E-11 | 0.222425 | 0.127 | 0.13 | 8.83E-07 |
| Gnptg | 6.88E-13 | 0.221431 | 0.275 | 0.265 | 3.82E-08 |
| Pdgfra | 4.97E-25 | 0.221284 | 0.826 | 0.815 | 2.75E-20 |
| Mgme1 | 4.89E-08 | 0.221194 | 0.104 | 0.103 | 0.002711 |
| Sertad2 | 1.78E-18 | 0.221056 | 0.251 | 0.258 | 9.86E-14 |
| Rhobtb3 | 1.19E-11 | 0.220103 | 0.195 | 0.193 | 6.61E-07 |
| Itih5 | 1.52E-13 | 0.219386 | 0.769 | 0.749 | 8.43E-09 |
| Rpl37rt | 1.02E-12 | 0.218992 | 0.173 | 0.171 | 5.65E-08 |
| Gramd1a | 9.74E-10 | 0.218248 | 0.105 | 0.107 | 5.40E-05 |
| Ftl1 | 6.40E-31 | 0.217944 | 0.836 | 0.816 | 3.55E-26 |
| Rbmxl1 | 4.51E-12 | 0.21789 | 0.202 | 0.199 | 2.50E-07 |
| Rpl27a | 1.68E-33 | 0.217677 | 0.854 | 0.839 | 9.33E-29 |
| Tmem11 | 1.24E-13 | 0.217437 | 0.248 | 0.241 | 6.84E-09 |
| Msantd2 | 4.05E-07 | 0.217124 | 0.105 | 0.105 | 0.022452 |
| Rpl28-ps1 | 6.07E-07 | 0.216285 | 0.12 | 0.117 | 0.033623 |
| Jun | 4.11E-14 | 0.215906 | 0.872 | 0.872 | 2.28E-09 |
| Immt | 8.42E-12 | 0.215114 | 0.207 | 0.204 | 4.67E-07 |
| Thra | 5.72E-24 | 0.213271 | 0.529 | 0.523 | 3.17E-19 |
| Rpl7 | 7.25E-46 | 0.213156 | 0.972 | 0.969 | 4.02E-41 |
| Zfp655 | 2.49E-07 | 0.212979 | 0.132 | 0.129 | 0.013779 |
| Nfkb1 | 1.86E-19 | 0.212884 | 0.298 | 0.317 | 1.03E-14 |
| Smad7 | 1.78E-13 | 0.211846 | 0.329 | 0.323 | 9.85E-09 |
| Zfas1 | 1.39E-19 | 0.21116 | 0.304 | 0.307 | 7.71E-15 |
| Arhgap24 | 9.03E-09 | 0.210176 | 0.098 | 0.101 | 0.0005 |
| Dcn | 6.04E-50 | 0.209473 | 1 | 0.999 | 3.35E-45 |
| Rpl10a-ps1 | 4.40E-12 | 0.209131 | 0.128 | 0.13 | 2.44E-07 |
| Eif4e | 2.44E-09 | 0.208634 | 0.16 | 0.16 | 0.000135 |
| Eif5 | 5.65E-25 | 0.208115 | 0.74 | 0.742 | 3.13E-20 |
| Metrnl | 5.91E-21 | 0.207933 | 0.508 | 0.529 | 3.27E-16 |
| Morc4 | 6.21E-11 | 0.207728 | 0.185 | 0.187 | 3.44E-06 |
| Pde12 | 2.20E-09 | 0.207598 | 0.139 | 0.143 | 0.000122 |
| Gm4149 | 4.03E-11 | 0.207016 | 0.194 | 0.19 | 2.23E-06 |
| Tubb6 | 5.28E-18 | 0.206975 | 0.411 | 0.412 | 2.93E-13 |
| Smc1a | 4.81E-23 | 0.206779 | 0.543 | 0.545 | 2.67E-18 |
| Chaserr | 1.04E-10 | 0.206207 | 0.282 | 0.273 | 5.76E-06 |
| Selenbp1 | 4.91E-07 | 0.206099 | 0.213 | 0.206 | 0.027187 |
| Ncoa7 | 4.28E-10 | 0.206095 | 0.159 | 0.159 | 2.37E-05 |
| Ubc | 4.67E-22 | 0.205375 | 0.928 | 0.925 | 2.59E-17 |
| Gm2000 | 2.23E-12 | 0.204815 | 0.239 | 0.235 | 1.24E-07 |

| | | | | | |
|---|---|---|---|---|---|
| Cdkn2d | 5.24E-12 | 0.204812 | 0.206 | 0.206 | 2.90E-07 |
| Chd1 | 2.03E-11 | 0.204597 | 0.22 | 0.219 | 1.12E-06 |
| Slc25a28 | 3.75E-08 | 0.204591 | 0.214 | 0.205 | 0.002078 |
| Slf2 | 3.21E-07 | 0.204585 | 0.129 | 0.129 | 0.017814 |
| Arhgap6 | 3.97E-11 | 0.204426 | 0.182 | 0.182 | 2.20E-06 |
| Col4a1 | 9.81E-19 | 0.204208 | 0.642 | 0.663 | 5.44E-14 |
| Tmpo | 1.16E-12 | 0.203718 | 0.211 | 0.21 | 6.43E-08 |
| Rpl19 | 7.10E-70 | 0.203574 | 0.995 | 0.996 | 3.93E-65 |
| Dnaja1 | 9.52E-17 | 0.203473 | 0.498 | 0.504 | 5.27E-12 |
| Cntln | 8.26E-15 | 0.203416 | 0.247 | 0.25 | 4.58E-10 |
| Sipa1 | 7.98E-10 | 0.203382 | 0.129 | 0.132 | 4.42E-05 |
| Sp100 | 2.23E-07 | 0.203175 | 0.11 | 0.11 | 0.012349 |
| Vps4b | 3.80E-09 | 0.202557 | 0.133 | 0.134 | 0.000211 |
| Gabarapl2 | 1.13E-12 | 0.202099 | 0.216 | 0.216 | 6.25E-08 |
| H2-T23 | 2.63E-07 | 0.201467 | 0.264 | 0.25 | 0.014547 |
| Nrp1 | 1.83E-17 | 0.201383 | 0.454 | 0.457 | 1.01E-12 |
| Skil | 2.25E-22 | 0.201008 | 0.367 | 0.381 | 1.25E-17 |
| Chad | 7.80E-09 | 0.200762 | 0.129 | 0.149 | 0.000433 |
| Snhg12 | 6.94E-16 | 0.200662 | 0.318 | 0.321 | 3.85E-11 |
| Sowahc | 5.28E-07 | 0.199572 | 0.114 | 0.118 | 0.029267 |
| C1qtnf7 | 2.07E-08 | 0.199543 | 0.211 | 0.21 | 0.001149 |
| Gm9794 | 4.57E-11 | 0.199154 | 0.233 | 0.23 | 2.53E-06 |
| Il11ra1 | 9.96E-20 | 0.198594 | 0.814 | 0.789 | 5.52E-15 |
| Tcf7l1 | 2.39E-08 | 0.198419 | 0.225 | 0.218 | 0.001327 |
| Gulp1 | 8.02E-13 | 0.197687 | 0.266 | 0.263 | 4.44E-08 |
| Adamts5 | 6.75E-12 | 0.197104 | 0.676 | 0.658 | 3.74E-07 |
| Akap9 | 5.33E-25 | 0.197074 | 0.361 | 0.372 | 2.96E-20 |
| Rbp1 | 5.16E-07 | 0.196404 | 0.332 | 0.298 | 0.028622 |
| Fmr1 | 9.69E-15 | 0.196254 | 0.315 | 0.316 | 5.37E-10 |
| Fem1b | 1.49E-13 | 0.195438 | 0.163 | 0.172 | 8.24E-09 |
| Nfil3 | 7.28E-15 | 0.195193 | 0.314 | 0.32 | 4.03E-10 |
| Fmo1 | 1.41E-07 | 0.194762 | 0.252 | 0.24 | 0.007804 |
| Zfand5 | 2.78E-24 | 0.193967 | 0.557 | 0.565 | 1.54E-19 |
| Srsf7 | 2.58E-23 | 0.193674 | 0.462 | 0.471 | 1.43E-18 |
| Cox7a2l | 2.80E-22 | 0.193565 | 0.516 | 0.513 | 1.55E-17 |
| Micu3 | 3.72E-07 | 0.193392 | 0.12 | 0.121 | 0.020596 |
| Phyh | 2.63E-09 | 0.193325 | 0.221 | 0.218 | 0.000146 |
| Clk1 | 2.06E-19 | 0.193198 | 0.437 | 0.439 | 1.14E-14 |
| Tnfrsf12a | 1.03E-14 | 0.192967 | 0.411 | 0.43 | 5.70E-10 |
| Hdgfl3 | 5.30E-16 | 0.192845 | 0.357 | 0.355 | 2.94E-11 |
| Fez1 | 1.37E-17 | 0.192183 | 0.463 | 0.461 | 7.59E-13 |
| Rps4x | 9.22E-47 | 0.191861 | 0.999 | 0.998 | 5.11E-42 |
| Rpl35a | 9.91E-42 | 0.191852 | 0.993 | 0.993 | 5.49E-37 |
| Hnrnpul2 | 1.28E-09 | 0.191572 | 0.194 | 0.193 | 7.11E-05 |
| Acbd4 | 1.51E-09 | 0.191253 | 0.122 | 0.124 | 8.37E-05 |
| Bglap2 | 8.06E-52 | 0.190935 | 0.173 | 0.268 | 4.47E-47 |
| Ppia | 8.16E-25 | 0.190891 | 0.673 | 0.668 | 4.52E-20 |

| | | | | | |
|---|---|---|---|---|---|
| Prdm2 | 6.58E-15 | 0.1907 | 0.241 | 0.246 | 3.64E-10 |
| Ccdc66 | 2.54E-08 | 0.190356 | 0.128 | 0.13 | 0.001408 |
| Elf1 | 3.67E-20 | 0.19009 | 0.416 | 0.424 | 2.04E-15 |
| Zrsr2 | 1.76E-11 | 0.189901 | 0.182 | 0.183 | 9.77E-07 |
| Crtc2 | 8.55E-07 | 0.189511 | 0.11 | 0.111 | 0.047387 |
| Tmem159 | 1.86E-22 | 0.189228 | 0.362 | 0.369 | 1.03E-17 |
| Cdkn1b | 1.94E-10 | 0.187718 | 0.294 | 0.29 | 1.08E-05 |
| Blvrb | 6.09E-23 | 0.187578 | 0.643 | 0.648 | 3.38E-18 |
| Klf3 | 3.01E-15 | 0.187277 | 0.533 | 0.54 | 1.67E-10 |
| Snrnp48 | 3.54E-13 | 0.186735 | 0.226 | 0.228 | 1.96E-08 |
| Cxcl14 | 1.00E-10 | 0.186704 | 0.339 | 0.351 | 5.55E-06 |
| Klf9 | 5.68E-19 | 0.186242 | 0.796 | 0.8 | 3.15E-14 |
| Selenop | 5.06E-22 | 0.186011 | 0.854 | 0.829 | 2.81E-17 |
| Mylip | 1.09E-07 | 0.185571 | 0.255 | 0.248 | 0.006028 |
| Snap29 | 5.39E-08 | 0.185463 | 0.108 | 0.111 | 0.002989 |
| Cyb5a | 1.38E-27 | 0.185422 | 0.907 | 0.904 | 7.65E-23 |
| Cep83 | 1.51E-09 | 0.184683 | 0.162 | 0.165 | 8.39E-05 |
| Abhd14b | 2.69E-12 | 0.184533 | 0.241 | 0.241 | 1.49E-07 |
| Rpl3 | 2.65E-31 | 0.184293 | 0.973 | 0.97 | 1.47E-26 |
| Upf2 | 2.02E-08 | 0.184219 | 0.177 | 0.175 | 0.001118 |
| Rps7 | 4.38E-41 | 0.183643 | 0.984 | 0.985 | 2.43E-36 |
| Pold2 | 7.41E-08 | 0.183546 | 0.129 | 0.13 | 0.004104 |
| Mcmbp | 1.17E-08 | 0.183393 | 0.102 | 0.105 | 0.00065 |
| Sap18 | 9.05E-24 | 0.183058 | 0.355 | 0.365 | 5.02E-19 |
| Tbc1d17 | 8.07E-14 | 0.182838 | 0.171 | 0.178 | 4.47E-09 |
| Hmgb1 | 3.21E-20 | 0.182368 | 0.719 | 0.704 | 1.78E-15 |
| Camk2n1 | 2.54E-13 | 0.181632 | 0.596 | 0.58 | 1.41E-08 |
| Itm2a | 1.51E-24 | 0.181588 | 0.68 | 0.592 | 8.38E-20 |
| Dtnbp1 | 6.37E-17 | 0.181414 | 0.487 | 0.484 | 3.53E-12 |
| Ifngr1 | 1.46E-26 | 0.181168 | 0.586 | 0.601 | 8.09E-22 |
| Rbm26 | 9.21E-12 | 0.180882 | 0.222 | 0.223 | 5.10E-07 |
| Tpt1 | 7.25E-42 | 0.180798 | 0.999 | 0.999 | 4.02E-37 |
| Hspa5 | 5.37E-13 | 0.17924 | 0.94 | 0.95 | 2.98E-08 |
| Ddah2 | 3.85E-24 | 0.178797 | 0.786 | 0.79 | 2.13E-19 |
| Rpl18 | 2.99E-33 | 0.178703 | 0.973 | 0.969 | 1.66E-28 |
| Suco | 1.49E-10 | 0.178407 | 0.181 | 0.184 | 8.27E-06 |
| Ncl | 7.13E-22 | 0.177951 | 0.812 | 0.813 | 3.95E-17 |
| Hnrnpa2b1 | 7.77E-20 | 0.17788 | 0.624 | 0.622 | 4.31E-15 |
| Ogfr | 3.16E-12 | 0.176929 | 0.146 | 0.151 | 1.75E-07 |
| Tbc1d12 | 4.11E-15 | 0.176537 | 0.197 | 0.205 | 2.28E-10 |
| Tlnrd1 | 2.82E-11 | 0.176272 | 0.154 | 0.158 | 1.56E-06 |
| Nol7 | 6.13E-20 | 0.176147 | 0.418 | 0.42 | 3.40E-15 |
| Arid5b | 3.56E-12 | 0.175956 | 0.509 | 0.499 | 1.97E-07 |
| Tbc1d10a | 2.06E-15 | 0.175639 | 0.245 | 0.249 | 1.14E-10 |
| Tsr2 | 3.93E-08 | 0.174869 | 0.118 | 0.122 | 0.002177 |
| Pi4k2b | 1.26E-10 | 0.174706 | 0.152 | 0.156 | 6.98E-06 |
| Ezh1 | 4.36E-08 | 0.174618 | 0.196 | 0.193 | 0.002418 |

| | | | | | |
|---|---|---|---|---|---|
| Zbtb11 | 1.89E-10 | 0.174535 | 0.131 | 0.136 | 1.05E-05 |
| Larp1b | 5.88E-07 | 0.174494 | 0.132 | 0.134 | 0.03256 |
| Zfp277 | 2.26E-08 | 0.174482 | 0.162 | 0.162 | 0.001255 |
| Zfp326 | 1.24E-10 | 0.174363 | 0.207 | 0.209 | 6.89E-06 |
| Tcp11l2 | 9.27E-17 | 0.174179 | 0.238 | 0.247 | 5.13E-12 |
| Bcl10 | 4.21E-12 | 0.173741 | 0.223 | 0.227 | 2.33E-07 |
| Fyn | 4.60E-18 | 0.17363 | 0.519 | 0.522 | 2.55E-13 |
| Marf1 | 2.38E-11 | 0.173463 | 0.177 | 0.182 | 1.32E-06 |
| Klhl7 | 5.64E-08 | 0.173258 | 0.13 | 0.132 | 0.003124 |
| Eif4a2 | 9.37E-22 | 0.173049 | 0.435 | 0.441 | 5.19E-17 |
| Luzp1 | 1.44E-16 | 0.172887 | 0.345 | 0.353 | 7.96E-12 |
| Cd302 | 2.04E-20 | 0.172773 | 0.778 | 0.774 | 1.13E-15 |
| Trmt10c | 8.73E-08 | 0.172722 | 0.097 | 0.101 | 0.004838 |
| Rps27a | 3.23E-41 | 0.17242 | 0.996 | 0.996 | 1.79E-36 |
| Mir6236 | 2.02E-12 | 0.172112 | 0.827 | 0.82 | 1.12E-07 |
| Ppp1r2 | 8.82E-18 | 0.172007 | 0.514 | 0.518 | 4.89E-13 |
| Dcaf6 | 4.59E-08 | 0.171428 | 0.102 | 0.106 | 0.002544 |
| Btf3 | 4.90E-19 | 0.171356 | 0.593 | 0.586 | 2.72E-14 |
| Cks1b | 1.03E-10 | 0.171274 | 0.275 | 0.274 | 5.68E-06 |
| Pja1 | 5.21E-12 | 0.170974 | 0.218 | 0.228 | 2.89E-07 |
| Acvrl1 | 2.59E-14 | 0.170915 | 0.347 | 0.347 | 1.44E-09 |
| Ypel3 | 4.34E-17 | 0.170585 | 0.589 | 0.585 | 2.40E-12 |
| Erf | 3.48E-15 | 0.170194 | 0.202 | 0.213 | 1.93E-10 |
| Mier3 | 4.02E-14 | 0.170188 | 0.163 | 0.17 | 2.23E-09 |
| Wdr61 | 9.13E-11 | 0.16988 | 0.163 | 0.167 | 5.06E-06 |
| Osbpl1a | 2.37E-13 | 0.16977 | 0.293 | 0.297 | 1.31E-08 |
| Ndufa6 | 4.02E-19 | 0.168819 | 0.722 | 0.719 | 2.23E-14 |
| Kif3a | 9.66E-15 | 0.168614 | 0.202 | 0.211 | 5.36E-10 |
| Rpl15 | 4.87E-17 | 0.168531 | 0.626 | 0.616 | 2.70E-12 |
| Tmem219 | 1.87E-08 | 0.168338 | 0.206 | 0.205 | 0.001035 |
| Trmt1 | 6.55E-07 | 0.168316 | 0.118 | 0.119 | 0.036314 |
| Cabin1 | 5.48E-07 | 0.168308 | 0.14 | 0.142 | 0.030341 |
| Diaph1 | 3.23E-21 | 0.167992 | 0.452 | 0.462 | 1.79E-16 |
| Ahsa1 | 6.28E-19 | 0.167864 | 0.31 | 0.318 | 3.48E-14 |
| Ethe1 | 7.73E-13 | 0.167673 | 0.189 | 0.197 | 4.28E-08 |
| Jmjd1c | 1.27E-19 | 0.167598 | 0.408 | 0.423 | 7.03E-15 |
| Zfp280d | 2.03E-08 | 0.16705 | 0.145 | 0.148 | 0.001124 |
| Sbno2 | 5.30E-14 | 0.166971 | 0.182 | 0.196 | 2.94E-09 |
| Trim47 | 9.06E-16 | 0.166773 | 0.414 | 0.418 | 5.02E-11 |
| Nip7 | 2.43E-13 | 0.166699 | 0.205 | 0.21 | 1.35E-08 |
| Chic2 | 4.53E-22 | 0.166441 | 0.562 | 0.566 | 2.51E-17 |
| Herpud1 | 3.19E-19 | 0.165817 | 0.539 | 0.551 | 1.77E-14 |
| Sptbn1 | 1.61E-17 | 0.165809 | 0.918 | 0.917 | 8.94E-13 |
| Mob2 | 1.68E-14 | 0.165628 | 0.223 | 0.231 | 9.33E-10 |
| Prpf6 | 2.28E-11 | 0.165207 | 0.158 | 0.165 | 1.27E-06 |
| Dnttip2 | 1.39E-10 | 0.165128 | 0.215 | 0.218 | 7.70E-06 |
| Slc16a1 | 5.33E-07 | 0.165126 | 0.147 | 0.149 | 0.029511 |

| | | | | | |
|---|---|---|---|---|---|
| Myo6 | 5.14E-12 | 0.165058 | 0.178 | 0.185 | 2.85E-07 |
| Rbm3 | 2.09E-09 | 0.16478 | 0.147 | 0.151 | 0.000116 |
| Smg6 | 9.45E-10 | 0.164722 | 0.234 | 0.236 | 5.24E-05 |
| Aldh2 | 2.78E-20 | 0.164607 | 0.841 | 0.838 | 1.54E-15 |
| Erh | 3.72E-09 | 0.16432 | 0.129 | 0.133 | 0.000206 |
| Yy1 | 3.02E-20 | 0.164311 | 0.399 | 0.407 | 1.67E-15 |
| Tgfb2 | 1.34E-15 | 0.164068 | 0.453 | 0.472 | 7.43E-11 |
| Smim3 | 1.71E-14 | 0.163742 | 0.109 | 0.124 | 9.49E-10 |
| Rasa2 | 4.27E-11 | 0.163277 | 0.148 | 0.155 | 2.37E-06 |
| Rbbp6 | 1.07E-08 | 0.163225 | 0.335 | 0.33 | 0.000594 |
| Calcoco1 | 2.89E-08 | 0.163156 | 0.124 | 0.127 | 0.001604 |
| Ncoa1 | 7.07E-11 | 0.162884 | 0.22 | 0.223 | 3.92E-06 |
| Sgce | 7.24E-23 | 0.162717 | 0.474 | 0.485 | 4.01E-18 |
| Azin1 | 7.64E-21 | 0.16246 | 0.402 | 0.417 | 4.24E-16 |
| Rpl5 | 2.61E-23 | 0.16239 | 0.968 | 0.966 | 1.45E-18 |
| Lamb1 | 4.76E-11 | 0.162348 | 0.273 | 0.282 | 2.64E-06 |
| Xpa | 1.55E-09 | 0.161876 | 0.21 | 0.212 | 8.62E-05 |
| Rab7b | 4.40E-16 | 0.161828 | 0.239 | 0.249 | 2.44E-11 |
| Ddx5 | 1.19E-19 | 0.161805 | 0.948 | 0.949 | 6.58E-15 |
| Ddx46 | 1.73E-15 | 0.161748 | 0.342 | 0.348 | 9.59E-11 |
| Zfp945 | 1.34E-10 | 0.161556 | 0.187 | 0.191 | 7.40E-06 |
| Rab40b | 2.90E-08 | 0.161441 | 0.108 | 0.111 | 0.001605 |
| Tra2b | 1.35E-16 | 0.16102 | 0.369 | 0.375 | 7.49E-12 |
| Hnrnph1 | 2.01E-18 | 0.160566 | 0.522 | 0.532 | 1.11E-13 |
| Mrps35 | 1.77E-13 | 0.160062 | 0.154 | 0.163 | 9.79E-09 |
| 2410004B1 | 2.34E-07 | 0.15983 | 0.181 | 0.181 | 0.012981 |
| Llph | 8.54E-17 | 0.159565 | 0.183 | 0.196 | 4.73E-12 |
| Serpinb1a | 1.06E-11 | 0.159513 | 0.262 | 0.272 | 5.87E-07 |
| Six1 | 2.69E-08 | 0.159222 | 0.138 | 0.142 | 0.001492 |
| Cys1 | 3.67E-11 | 0.159141 | 0.199 | 0.206 | 2.03E-06 |
| Bclaf1 | 4.07E-14 | 0.159033 | 0.318 | 0.327 | 2.26E-09 |
| Mgst1 | 3.79E-13 | 0.158189 | 0.794 | 0.784 | 2.10E-08 |
| Uhrf2 | 2.95E-11 | 0.157446 | 0.176 | 0.182 | 1.63E-06 |
| Tax1bp1 | 1.52E-22 | 0.157423 | 0.725 | 0.73 | 8.42E-18 |
| Maco1 | 1.02E-14 | 0.157284 | 0.223 | 0.233 | 5.67E-10 |
| Pcdh7 | 3.08E-11 | 0.156783 | 0.221 | 0.226 | 1.70E-06 |
| Eif4g3 | 4.95E-15 | 0.156555 | 0.375 | 0.382 | 2.74E-10 |
| Top1 | 1.20E-17 | 0.156443 | 0.596 | 0.611 | 6.62E-13 |
| Hmgb2 | 3.40E-11 | 0.156414 | 0.138 | 0.147 | 1.88E-06 |
| Rnd3 | 3.54E-15 | 0.156376 | 0.379 | 0.39 | 1.96E-10 |
| Plpp3 | 1.09E-10 | 0.15597 | 0.871 | 0.873 | 6.04E-06 |
| Zscan26 | 1.72E-07 | 0.155886 | 0.172 | 0.172 | 0.009526 |
| Papss1 | 4.46E-15 | 0.155788 | 0.351 | 0.356 | 2.47E-10 |
| Prpf4b | 1.48E-14 | 0.155633 | 0.39 | 0.396 | 8.18E-10 |
| Mier1 | 6.94E-18 | 0.155621 | 0.438 | 0.446 | 3.85E-13 |
| Arhgap12 | 8.77E-09 | 0.155111 | 0.127 | 0.131 | 0.000486 |
| Cdc42se2 | 2.36E-08 | 0.155008 | 0.116 | 0.121 | 0.001309 |

| | | | | | |
|---|---|---|---|---|---|
| Impdh2 | 6.69E-16 | 0.154999 | 0.359 | 0.366 | 3.71E-11 |
| Acp1 | 1.19E-14 | 0.154835 | 0.228 | 0.238 | 6.59E-10 |
| Npr2 | 6.72E-07 | 0.154699 | 0.175 | 0.173 | 0.037248 |
| Rps16 | 1.78E-36 | 0.154655 | 0.996 | 0.996 | 9.84E-32 |
| Dipk1b | 2.39E-11 | 0.154652 | 0.13 | 0.137 | 1.32E-06 |
| Gpx4-ps2 | 1.30E-08 | 0.154447 | 0.132 | 0.137 | 0.000723 |
| Hsf1 | 8.84E-08 | 0.15435 | 0.108 | 0.113 | 0.004901 |
| Dtd1 | 1.24E-07 | 0.154239 | 0.234 | 0.23 | 0.006884 |
| Arhgap35 | 2.84E-11 | 0.153746 | 0.206 | 0.211 | 1.57E-06 |
| Rpa3 | 4.41E-12 | 0.153651 | 0.186 | 0.195 | 2.44E-07 |
| Aven | 1.75E-07 | 0.153606 | 0.127 | 0.13 | 0.009709 |
| Las1l | 1.29E-11 | 0.153503 | 0.156 | 0.166 | 7.16E-07 |
| Dnase1l1 | 3.44E-07 | 0.153308 | 0.147 | 0.149 | 0.019062 |
| Abcc5 | 3.46E-10 | 0.153133 | 0.116 | 0.123 | 1.92E-05 |
| Ascc3 | 3.20E-08 | 0.152533 | 0.157 | 0.161 | 0.001773 |
| Cdc37l1 | 8.42E-10 | 0.152531 | 0.149 | 0.155 | 4.67E-05 |
| Eif4a1 | 1.67E-24 | 0.152466 | 0.664 | 0.684 | 9.26E-20 |
| Actg1 | 1.18E-08 | 0.152444 | 0.22 | 0.221 | 0.000651 |
| Supt16 | 2.61E-14 | 0.152167 | 0.364 | 0.368 | 1.45E-09 |
| Ice1 | 1.40E-07 | 0.152133 | 0.127 | 0.13 | 0.007772 |
| Dek | 5.57E-18 | 0.151914 | 0.482 | 0.485 | 3.09E-13 |
| Wdr44 | 1.03E-09 | 0.1518 | 0.144 | 0.15 | 5.72E-05 |
| Chchd5 | 1.61E-12 | 0.15174 | 0.103 | 0.115 | 8.92E-08 |
| Ets2 | 7.95E-07 | 0.151533 | 0.168 | 0.169 | 0.044065 |
| Rps3a1 | 1.02E-34 | 0.151446 | 0.998 | 0.998 | 5.67E-30 |
| Rhog | 2.80E-12 | 0.15117 | 0.193 | 0.202 | 1.55E-07 |
| C1ra | 1.06E-16 | 0.150864 | 0.674 | 0.674 | 5.89E-12 |
| Il10rb | 1.17E-14 | 0.150811 | 0.195 | 0.205 | 6.46E-10 |
| Rpl30 | 5.77E-28 | 0.15077 | 0.989 | 0.991 | 3.20E-23 |
| Sf3a2 | 6.83E-11 | 0.150721 | 0.147 | 0.155 | 3.78E-06 |
| Psenen | 1.02E-12 | 0.150667 | 0.367 | 0.367 | 5.66E-08 |
| Ier5l | 2.56E-07 | 0.150551 | 0.168 | 0.172 | 0.014195 |
| Crybg3 | 4.04E-14 | 0.150548 | 0.259 | 0.269 | 2.24E-09 |
| Alyref | 8.31E-20 | 0.150508 | 0.384 | 0.394 | 4.61E-15 |
| Katna1 | 5.57E-08 | 0.150265 | 0.188 | 0.191 | 0.003088 |
| Polr3gl | 2.19E-11 | 0.149907 | 0.16 | 0.167 | 1.21E-06 |
| Foxn2 | 1.28E-08 | 0.149513 | 0.155 | 0.16 | 0.000709 |
| Baiap2 | 7.86E-25 | 0.149508 | 0.329 | 0.356 | 4.35E-20 |
| Nr3c1 | 1.19E-16 | 0.149484 | 0.724 | 0.724 | 6.58E-12 |
| Mcu | 1.06E-10 | 0.149179 | 0.17 | 0.176 | 5.87E-06 |
| Nsa2 | 2.46E-15 | 0.149111 | 0.487 | 0.495 | 1.36E-10 |
| Tsc22d4 | 4.48E-08 | 0.149068 | 0.205 | 0.205 | 0.002483 |
| Eif4b | 2.91E-17 | 0.148892 | 0.482 | 0.49 | 1.61E-12 |
| Pcna | 1.62E-09 | 0.148488 | 0.197 | 0.202 | 8.98E-05 |
| Lims2 | 5.52E-15 | 0.148381 | 0.231 | 0.243 | 3.06E-10 |
| Rpl9 | 8.75E-35 | 0.148348 | 0.996 | 0.997 | 4.85E-30 |
| Mpst | 3.96E-10 | 0.148093 | 0.231 | 0.233 | 2.20E-05 |

| | | | | | |
|---|---|---|---|---|---|
| Rapgef6 | 3.79E-13 | 0.147523 | 0.247 | 0.254 | 2.10E-08 |
| Rplp0 | 6.26E-26 | 0.147098 | 0.994 | 0.994 | 3.47E-21 |
| Amd1 | 2.22E-08 | 0.146898 | 0.119 | 0.124 | 0.001228 |
| Rps10 | 6.01E-16 | 0.146827 | 0.93 | 0.932 | 3.33E-11 |
| Nono | 8.64E-07 | 0.146129 | 0.127 | 0.128 | 0.047881 |
| Rdm1 | 4.29E-16 | 0.145944 | 0.218 | 0.229 | 2.38E-11 |
| Hebp2 | 1.49E-12 | 0.145882 | 0.209 | 0.218 | 8.25E-08 |
| Csf1 | 8.35E-20 | 0.145869 | 0.514 | 0.535 | 4.63E-15 |
| Topors | 2.04E-11 | 0.145826 | 0.241 | 0.25 | 1.13E-06 |
| H2-M3 | 1.40E-09 | 0.145817 | 0.172 | 0.177 | 7.78E-05 |
| Errfi1 | 3.00E-09 | 0.14563 | 0.387 | 0.405 | 0.000166 |
| Twf2 | 3.98E-11 | 0.145498 | 0.208 | 0.213 | 2.21E-06 |
| Iqsec2 | 1.02E-10 | 0.145322 | 0.115 | 0.122 | 5.67E-06 |
| Chd6 | 5.24E-15 | 0.145282 | 0.253 | 0.265 | 2.90E-10 |
| Ube2q2 | 5.09E-07 | 0.144579 | 0.125 | 0.128 | 0.028192 |
| Smc3 | 4.88E-18 | 0.144146 | 0.451 | 0.463 | 2.70E-13 |
| Cc2d2a | 2.75E-14 | 0.144015 | 0.259 | 0.269 | 1.52E-09 |
| Gsk3a | 1.63E-22 | 0.143993 | 0.284 | 0.303 | 9.02E-18 |
| Ehd4 | 5.56E-10 | 0.143981 | 0.177 | 0.182 | 3.08E-05 |
| Ythdc1 | 2.77E-10 | 0.143386 | 0.247 | 0.252 | 1.53E-05 |
| Igbp1 | 5.06E-11 | 0.143378 | 0.295 | 0.297 | 2.80E-06 |
| Anp32b | 2.76E-15 | 0.143375 | 0.584 | 0.583 | 1.53E-10 |
| Phactr2 | 7.27E-16 | 0.143296 | 0.397 | 0.406 | 4.03E-11 |
| Uap1l1 | 1.85E-09 | 0.143098 | 0.199 | 0.204 | 0.000102 |
| Aldh9a1 | 6.93E-08 | 0.143075 | 0.122 | 0.126 | 0.003841 |
| Baz2b | 6.38E-14 | 0.142965 | 0.255 | 0.264 | 3.54E-09 |
| Sde2 | 3.89E-13 | 0.142 | 0.293 | 0.303 | 2.16E-08 |
| Golm2 | 1.98E-08 | 0.141922 | 0.278 | 0.279 | 0.001095 |
| Zswim6 | 3.69E-10 | 0.141722 | 0.132 | 0.142 | 2.04E-05 |
| Tcea1 | 1.09E-09 | 0.141351 | 0.2 | 0.204 | 6.06E-05 |
| Abca1 | 1.35E-09 | 0.141048 | 0.303 | 0.307 | 7.46E-05 |
| Eef1d | 9.07E-18 | 0.140989 | 0.901 | 0.903 | 5.02E-13 |
| Crebrf | 1.29E-10 | 0.140871 | 0.199 | 0.207 | 7.12E-06 |
| Npepps | 1.91E-20 | 0.140755 | 0.21 | 0.229 | 1.06E-15 |
| Rps5 | 1.97E-29 | 0.140747 | 0.998 | 0.998 | 1.09E-24 |
| Ypel5 | 1.75E-16 | 0.140484 | 0.187 | 0.2 | 9.72E-12 |
| Rps23 | 2.64E-26 | 0.140097 | 0.997 | 0.995 | 1.46E-21 |
| Cr1l | 1.03E-15 | 0.139583 | 0.377 | 0.386 | 5.73E-11 |
| Mcee | 2.11E-14 | 0.139098 | 0.302 | 0.309 | 1.17E-09 |
| Pnn | 6.94E-16 | 0.139035 | 0.431 | 0.444 | 3.84E-11 |
| Tdrd3 | 6.07E-07 | 0.138824 | 0.141 | 0.145 | 0.033623 |
| Thap3 | 5.56E-09 | 0.138799 | 0.174 | 0.178 | 0.000308 |
| Gab2 | 5.54E-08 | 0.138791 | 0.097 | 0.104 | 0.00307 |
| Alkbh7 | 2.15E-13 | 0.138748 | 0.166 | 0.177 | 1.19E-08 |
| Sf3b1 | 3.06E-20 | 0.138383 | 0.651 | 0.666 | 1.70E-15 |
| Ahnak2 | 3.51E-17 | 0.138361 | 0.632 | 0.646 | 1.94E-12 |
| Srpr | 7.90E-12 | 0.137771 | 0.31 | 0.316 | 4.38E-07 |

| | | | | | |
|---|---|---|---|---|---|
| Rps24 | 1.92E-27 | 0.137747 | 0.999 | 0.999 | 1.07E-22 |
| Ubn1 | 4.49E-14 | 0.137508 | 0.344 | 0.351 | 2.49E-09 |
| Nfkbib | 1.51E-10 | 0.137215 | 0.129 | 0.137 | 8.35E-06 |
| Col14a1 | 2.70E-07 | 0.137166 | 0.801 | 0.795 | 0.014975 |
| Prrc2c | 8.73E-18 | 0.136989 | 0.619 | 0.63 | 4.84E-13 |
| Rock1 | 7.47E-18 | 0.136873 | 0.642 | 0.649 | 4.14E-13 |
| Aggf1 | 1.90E-13 | 0.135549 | 0.222 | 0.231 | 1.05E-08 |
| Plxdc2 | 2.11E-11 | 0.135523 | 0.832 | 0.838 | 1.17E-06 |
| Add3 | 4.82E-14 | 0.135402 | 0.71 | 0.713 | 2.67E-09 |
| Mxi1 | 1.59E-15 | 0.135367 | 0.205 | 0.218 | 8.84E-11 |
| Fdx1 | 8.47E-15 | 0.135274 | 0.378 | 0.39 | 4.70E-10 |
| Ablim1 | 6.54E-14 | 0.135238 | 0.679 | 0.69 | 3.63E-09 |
| Eloa | 1.61E-13 | 0.135092 | 0.218 | 0.231 | 8.91E-09 |
| Fip1l1 | 4.51E-17 | 0.13482 | 0.316 | 0.331 | 2.50E-12 |
| Eef1a1 | 8.12E-18 | 0.134811 | 0.83 | 0.831 | 4.50E-13 |
| Ppp1r12a | 2.08E-15 | 0.134582 | 0.255 | 0.268 | 1.15E-10 |
| H2az1 | 5.58E-19 | 0.134578 | 0.213 | 0.229 | 3.09E-14 |
| Rnf103 | 7.34E-12 | 0.134483 | 0.195 | 0.204 | 4.07E-07 |
| Rps20 | 1.35E-22 | 0.134462 | 0.999 | 0.998 | 7.50E-18 |
| Rpl11 | 1.56E-25 | 0.134295 | 0.998 | 0.997 | 8.65E-21 |
| Dipk2a | 1.55E-13 | 0.133606 | 0.166 | 0.178 | 8.57E-09 |
| Ptpn13 | 3.56E-08 | 0.133452 | 0.147 | 0.153 | 0.001976 |
| Vegfc | 1.94E-08 | 0.13341 | 0.154 | 0.158 | 0.001077 |
| Tcf7l2 | 4.10E-10 | 0.133209 | 0.366 | 0.367 | 2.27E-05 |
| Nid1 | 1.12E-07 | 0.132817 | 0.647 | 0.653 | 0.006219 |
| Rbm39 | 1.52E-14 | 0.132764 | 0.745 | 0.751 | 8.42E-10 |
| Pfdn5 | 4.43E-16 | 0.132668 | 0.97 | 0.966 | 2.46E-11 |
| Hat1 | 2.22E-07 | 0.132575 | 0.123 | 0.127 | 0.012275 |
| Dapk1 | 7.18E-10 | 0.131836 | 0.332 | 0.336 | 3.98E-05 |
| Rab5a | 7.07E-08 | 0.131797 | 0.151 | 0.156 | 0.003918 |
| Slit3 | 2.13E-11 | 0.13179 | 0.356 | 0.364 | 1.18E-06 |
| Acaa2 | 6.06E-17 | 0.13124 | 0.344 | 0.357 | 3.36E-12 |
| Rpl14 | 5.03E-21 | 0.131216 | 0.993 | 0.992 | 2.79E-16 |
| Ptpn12 | 4.82E-15 | 0.131201 | 0.246 | 0.26 | 2.67E-10 |
| Rpl34 | 3.90E-23 | 0.130766 | 0.993 | 0.992 | 2.16E-18 |
| Rpl18a | 2.26E-21 | 0.130511 | 0.996 | 0.997 | 1.25E-16 |
| Nfe2l2 | 1.71E-18 | 0.130118 | 0.492 | 0.51 | 9.45E-14 |
| Gsto1 | 1.53E-07 | 0.130106 | 0.098 | 0.105 | 0.008501 |
| Thrap3 | 2.86E-13 | 0.129924 | 0.23 | 0.242 | 1.59E-08 |
| Ahcyl2 | 9.14E-13 | 0.129739 | 0.099 | 0.112 | 5.06E-08 |
| Gabpb2 | 1.56E-13 | 0.129721 | 0.185 | 0.196 | 8.63E-09 |
| Dusp16 | 2.63E-11 | 0.129631 | 0.105 | 0.114 | 1.46E-06 |
| Hyi | 8.38E-19 | 0.129547 | 0.344 | 0.36 | 4.64E-14 |
| Arntl | 7.44E-15 | 0.129515 | 0.189 | 0.202 | 4.12E-10 |
| Mtus1 | 3.29E-10 | 0.129317 | 0.109 | 0.117 | 1.82E-05 |
| Ifi211 | 1.23E-19 | 0.129242 | 0.337 | 0.362 | 6.80E-15 |
| Arpc1b | 1.57E-17 | 0.129087 | 0.54 | 0.548 | 8.72E-13 |

| | | | | | |
|---|---|---|---|---|---|
| Rps3 | 4.25E-15 | 0.129077 | 0.841 | 0.844 | 2.35E-10 |
| Ar | 1.79E-11 | 0.128913 | 0.606 | 0.608 | 9.91E-07 |
| Agfg2 | 9.15E-09 | 0.128713 | 0.12 | 0.127 | 0.000507 |
| Senp6 | 1.25E-14 | 0.128689 | 0.468 | 0.474 | 6.91E-10 |
| Magi3 | 8.70E-07 | 0.128637 | 0.142 | 0.146 | 0.048197 |
| Nipbl | 1.89E-22 | 0.128527 | 0.519 | 0.543 | 1.04E-17 |
| Msx1 | 1.51E-08 | 0.128322 | 0.287 | 0.294 | 0.000836 |
| Plin3 | 4.08E-20 | 0.128243 | 0.428 | 0.447 | 2.26E-15 |
| Fmnl2 | 7.00E-14 | 0.128156 | 0.217 | 0.23 | 3.88E-09 |
| Gbp2 | 1.49E-09 | 0.128146 | 0.182 | 0.191 | 8.27E-05 |
| Disp1 | 6.02E-08 | 0.128073 | 0.133 | 0.14 | 0.003333 |
| Eif3j1 | 1.83E-10 | 0.127773 | 0.196 | 0.204 | 1.01E-05 |
| Ntan1 | 3.36E-15 | 0.127732 | 0.473 | 0.48 | 1.86E-10 |
| Map2k3 | 1.62E-16 | 0.127678 | 0.296 | 0.315 | 8.97E-12 |
| Syncrip | 6.24E-07 | 0.12758 | 0.164 | 0.167 | 0.034567 |
| Nop53 | 7.39E-14 | 0.127143 | 0.526 | 0.528 | 4.09E-09 |
| Fbln5 | 1.24E-13 | 0.126964 | 0.551 | 0.563 | 6.89E-09 |
| Kdm5b | 6.77E-10 | 0.126693 | 0.213 | 0.219 | 3.75E-05 |
| Rps25 | 2.88E-16 | 0.126601 | 0.953 | 0.955 | 1.60E-11 |
| Etf1 | 2.46E-18 | 0.126516 | 0.376 | 0.395 | 1.37E-13 |
| Ubap1 | 1.18E-12 | 0.126491 | 0.141 | 0.153 | 6.55E-08 |
| Rpl8 | 9.60E-22 | 0.125961 | 0.997 | 0.997 | 5.32E-17 |
| Ubr7 | 1.68E-07 | 0.125866 | 0.095 | 0.101 | 0.009338 |
| Pura | 1.01E-13 | 0.125799 | 0.675 | 0.68 | 5.60E-09 |
| Oaz1 | 1.17E-17 | 0.125552 | 0.612 | 0.623 | 6.47E-13 |
| Eea1 | 9.50E-18 | 0.125212 | 0.467 | 0.483 | 5.27E-13 |
| Foxp4 | 2.08E-09 | 0.125156 | 0.111 | 0.12 | 0.000115 |
| Senp7 | 1.61E-15 | 0.124929 | 0.177 | 0.191 | 8.94E-11 |
| Tpr | 7.03E-14 | 0.124762 | 0.654 | 0.661 | 3.89E-09 |
| Arsk | 3.10E-09 | 0.124706 | 0.214 | 0.221 | 0.000172 |
| Rb1cc1 | 1.58E-18 | 0.124654 | 0.42 | 0.437 | 8.75E-14 |
| Tmod2 | 4.92E-10 | 0.124642 | 0.584 | 0.591 | 2.73E-05 |
| Rps26 | 3.86E-18 | 0.124255 | 0.994 | 0.994 | 2.14E-13 |
| Luc7l3 | 7.05E-14 | 0.124241 | 0.469 | 0.48 | 3.90E-09 |
| Tjp1 | 3.44E-15 | 0.124204 | 0.244 | 0.259 | 1.91E-10 |
| Hpcal1 | 9.42E-12 | 0.123928 | 0.18 | 0.19 | 5.22E-07 |
| Rpl26 | 1.25E-20 | 0.12341 | 0.996 | 0.996 | 6.92E-16 |
| Notch2 | 3.22E-18 | 0.123349 | 0.321 | 0.338 | 1.78E-13 |
| Gm33989 | 1.31E-07 | 0.123344 | 0.144 | 0.151 | 0.007258 |
| Rnf19a | 3.18E-09 | 0.123329 | 0.171 | 0.178 | 0.000176 |
| Hsp90ab1 | 2.75E-13 | 0.123287 | 0.987 | 0.988 | 1.52E-08 |
| Klhdc2 | 7.92E-15 | 0.122998 | 0.368 | 0.378 | 4.39E-10 |
| Cbr3 | 1.38E-12 | 0.122887 | 0.362 | 0.37 | 7.63E-08 |
| Fubp1 | 1.72E-07 | 0.122822 | 0.196 | 0.199 | 0.009522 |
| Rpl35 | 1.24E-15 | 0.122645 | 0.959 | 0.958 | 6.89E-11 |
| Rps9 | 9.92E-20 | 0.122613 | 0.997 | 0.997 | 5.50E-15 |
| Bdp1 | 2.77E-14 | 0.122487 | 0.225 | 0.238 | 1.53E-09 |

| | | | | | |
|---|---|---|---|---|---|
| Cenpv | 6.44E-10 | 0.122423 | 0.155 | 0.164 | 3.57E-05 |
| Bcl7c | 1.93E-18 | 0.122227 | 0.466 | 0.481 | 1.07E-13 |
| Rlim | 8.14E-13 | 0.122038 | 0.208 | 0.219 | 4.51E-08 |
| Cct5 | 1.53E-14 | 0.121974 | 0.631 | 0.64 | 8.50E-10 |
| Mphosph8 | 2.40E-14 | 0.121632 | 0.26 | 0.275 | 1.33E-09 |
| Bach1 | 9.52E-09 | 0.121432 | 0.155 | 0.162 | 0.000527 |
| U2af2 | 3.29E-08 | 0.12128 | 0.109 | 0.116 | 0.001824 |
| Slfn5 | 8.62E-07 | 0.121061 | 0.391 | 0.394 | 0.047783 |
| Bag3 | 1.14E-08 | 0.120232 | 0.518 | 0.533 | 0.00063 |
| Twist1 | 1.77E-11 | 0.120196 | 0.559 | 0.57 | 9.81E-07 |
| Kras | 1.34E-16 | 0.120014 | 0.435 | 0.453 | 7.42E-12 |
| Lpcat1 | 2.22E-12 | 0.120002 | 0.143 | 0.154 | 1.23E-07 |
| Afdn | 8.49E-13 | 0.119932 | 0.327 | 0.337 | 4.70E-08 |
| Mtarc2 | 1.38E-16 | 0.119882 | 0.474 | 0.485 | 7.63E-12 |
| Kif2a | 9.06E-19 | 0.119718 | 0.266 | 0.289 | 5.02E-14 |
| Rpl4 | 1.92E-14 | 0.119691 | 0.972 | 0.969 | 1.06E-09 |
| Rsf1 | 1.39E-19 | 0.119405 | 0.261 | 0.28 | 7.72E-15 |
| Cmklr1 | 1.16E-10 | 0.119269 | 0.111 | 0.121 | 6.42E-06 |
| Ncoa6 | 1.98E-12 | 0.11915 | 0.135 | 0.147 | 1.10E-07 |
| Hdac7 | 4.17E-10 | 0.119045 | 0.258 | 0.266 | 2.31E-05 |
| Plekho2 | 2.67E-21 | 0.118755 | 0.228 | 0.25 | 1.48E-16 |
| Clk4 | 1.08E-07 | 0.118727 | 0.164 | 0.169 | 0.006008 |
| Eif3h | 6.71E-15 | 0.118629 | 0.83 | 0.838 | 3.72E-10 |
| Mrpl38 | 1.76E-11 | 0.118627 | 0.167 | 0.178 | 9.77E-07 |
| Nsmce4a | 1.45E-10 | 0.118508 | 0.357 | 0.362 | 8.05E-06 |
| Cygb | 2.87E-10 | 0.118455 | 0.743 | 0.749 | 1.59E-05 |
| Nop56 | 8.31E-09 | 0.118123 | 0.13 | 0.137 | 0.000461 |
| Fcgrt | 3.95E-15 | 0.117967 | 0.735 | 0.743 | 2.19E-10 |
| Rpl7a | 2.34E-18 | 0.117942 | 0.978 | 0.979 | 1.29E-13 |
| Epb41l4aos | 2.60E-07 | 0.117811 | 0.188 | 0.192 | 0.014395 |
| Rps8 | 2.95E-26 | 0.11772 | 0.999 | 0.999 | 1.64E-21 |
| Cd34 | 5.22E-09 | 0.117556 | 0.863 | 0.84 | 0.000289 |
| Rest | 4.99E-14 | 0.117298 | 0.287 | 0.305 | 2.77E-09 |
| Phip | 3.15E-10 | 0.11729 | 0.318 | 0.329 | 1.75E-05 |
| Mia2 | 7.26E-15 | 0.117242 | 0.27 | 0.286 | 4.02E-10 |
| Nsd3 | 3.24E-12 | 0.116969 | 0.313 | 0.323 | 1.79E-07 |
| Orc4 | 1.79E-08 | 0.116764 | 0.16 | 0.165 | 0.000989 |
| Plcd1 | 2.70E-15 | 0.116704 | 0.169 | 0.183 | 1.50E-10 |
| Sp3 | 1.09E-15 | 0.116593 | 0.404 | 0.42 | 6.04E-11 |
| Il3ra | 1.52E-11 | 0.116589 | 0.181 | 0.192 | 8.42E-07 |
| Ppp2r5d | 6.13E-11 | 0.116487 | 0.123 | 0.131 | 3.40E-06 |
| Reck | 1.05E-09 | 0.116136 | 0.296 | 0.303 | 5.83E-05 |
| Rsrc2 | 1.77E-13 | 0.116077 | 0.429 | 0.443 | 9.82E-09 |
| Ralbp1 | 1.21E-16 | 0.115932 | 0.634 | 0.652 | 6.73E-12 |
| Snw1 | 1.88E-17 | 0.115879 | 0.418 | 0.435 | 1.04E-12 |
| Hp1bp3 | 1.36E-14 | 0.115821 | 0.5 | 0.511 | 7.55E-10 |
| Rab6b | 2.03E-11 | 0.115753 | 0.222 | 0.232 | 1.12E-06 |

| | | | | | |
|---|---|---|---|---|---|
| Rps13 | 5.07E-17 | 0.115587 | 0.994 | 0.996 | 2.81E-12 |
| Rsbn1 | 6.46E-11 | 0.11542 | 0.19 | 0.2 | 3.58E-06 |
| Itm2b | 4.23E-25 | 0.115339 | 0.999 | 0.999 | 2.34E-20 |
| Pnpla2 | 8.31E-10 | 0.11533 | 0.096 | 0.106 | 4.61E-05 |
| Jade1 | 6.23E-10 | 0.115244 | 0.191 | 0.203 | 3.45E-05 |
| Stk40 | 3.31E-13 | 0.115207 | 0.187 | 0.2 | 1.83E-08 |
| Pabpc1 | 2.12E-09 | 0.115086 | 0.788 | 0.785 | 0.000118 |
| Hnrnpf | 5.64E-20 | 0.115022 | 0.436 | 0.457 | 3.12E-15 |
| Rfxap | 2.39E-09 | 0.114969 | 0.167 | 0.174 | 0.000133 |
| Taf13 | 6.26E-10 | 0.114683 | 0.124 | 0.133 | 3.47E-05 |
| Polr1f | 2.28E-13 | 0.114653 | 0.189 | 0.203 | 1.26E-08 |
| Znhit6 | 4.76E-09 | 0.114635 | 0.127 | 0.136 | 0.000264 |
| Srrm2 | 7.33E-12 | 0.114538 | 0.709 | 0.714 | 4.06E-07 |
| Arl5a | 1.99E-10 | 0.114371 | 0.236 | 0.246 | 1.10E-05 |
| Rnf146 | 7.36E-15 | 0.114365 | 0.206 | 0.219 | 4.08E-10 |
| Kmt2c | 1.37E-13 | 0.114309 | 0.405 | 0.419 | 7.58E-09 |
| Atad2b | 7.12E-08 | 0.11426 | 0.141 | 0.147 | 0.003944 |
| Rpl12 | 2.47E-14 | 0.114225 | 0.995 | 0.995 | 1.37E-09 |
| Zfp131 | 4.63E-12 | 0.114116 | 0.268 | 0.278 | 2.57E-07 |
| Mir703 | 1.05E-14 | 0.114092 | 0.678 | 0.693 | 5.82E-10 |
| Rela | 4.01E-18 | 0.114027 | 0.314 | 0.333 | 2.22E-13 |
| Kdm1a | 4.22E-15 | 0.113931 | 0.322 | 0.335 | 2.34E-10 |
| Surf2 | 1.63E-10 | 0.113799 | 0.13 | 0.139 | 9.04E-06 |
| Rps14 | 1.31E-23 | 0.113603 | 0.999 | 0.998 | 7.24E-19 |
| Sdccag8 | 1.83E-08 | 0.113449 | 0.1 | 0.109 | 0.001013 |
| Zfp68 | 7.55E-11 | 0.113131 | 0.15 | 0.16 | 4.18E-06 |
| Ift57 | 4.74E-11 | 0.112905 | 0.191 | 0.201 | 2.63E-06 |
| Tmod3 | 8.26E-14 | 0.112765 | 0.5 | 0.513 | 4.58E-09 |
| Zfp830 | 5.26E-09 | 0.112632 | 0.111 | 0.12 | 0.000291 |
| Hnrnpu | 1.89E-21 | 0.11234 | 0.481 | 0.506 | 1.05E-16 |
| Nfya | 9.83E-08 | 0.112115 | 0.105 | 0.111 | 0.005446 |
| Eif3m | 2.15E-16 | 0.111997 | 0.543 | 0.556 | 1.19E-11 |
| Smc6 | 8.48E-19 | 0.111825 | 0.541 | 0.561 | 4.70E-14 |
| Eps8 | 1.43E-12 | 0.111627 | 0.278 | 0.293 | 7.92E-08 |
| Spry1 | 1.82E-07 | 0.111521 | 0.163 | 0.171 | 0.010087 |
| Rbm18 | 1.30E-11 | 0.111509 | 0.186 | 0.198 | 7.20E-07 |
| Nmi | 8.65E-09 | 0.111185 | 0.14 | 0.148 | 0.000479 |
| Apbb1ip | 2.58E-12 | 0.111016 | 0.316 | 0.33 | 1.43E-07 |
| Stxbp5 | 4.72E-09 | 0.110861 | 0.134 | 0.142 | 0.000262 |
| Brcc3 | 4.77E-14 | 0.110581 | 0.145 | 0.16 | 2.65E-09 |
| Arid4b | 1.90E-14 | 0.110553 | 0.372 | 0.387 | 1.05E-09 |
| Ppp4r2 | 4.38E-19 | 0.11005 | 0.251 | 0.27 | 2.43E-14 |
| Tiprl | 6.45E-19 | 0.109756 | 0.337 | 0.358 | 3.57E-14 |
| Smndc1 | 2.19E-12 | 0.109745 | 0.252 | 0.265 | 1.21E-07 |
| Eif3f | 4.25E-11 | 0.109393 | 0.88 | 0.884 | 2.36E-06 |
| Kmt2a | 4.35E-12 | 0.109115 | 0.395 | 0.407 | 2.41E-07 |
| Wasf2 | 3.13E-14 | 0.109057 | 0.675 | 0.687 | 1.73E-09 |

| | | | | | |
|---|---|---|---|---|---|
| Fermt2 | 1.75E-08 | 0.109033 | 0.738 | 0.739 | 0.000971 |
| Mnt | 8.91E-08 | 0.108928 | 0.095 | 0.103 | 0.004938 |
| Tent5a | 2.53E-09 | 0.108908 | 0.464 | 0.477 | 0.00014 |
| Nktr | 9.37E-16 | 0.108663 | 0.334 | 0.352 | 5.19E-11 |
| Arhgap21 | 1.34E-16 | 0.108475 | 0.405 | 0.423 | 7.43E-12 |
| Bok | 3.14E-19 | 0.108462 | 0.21 | 0.232 | 1.74E-14 |
| Gps2 | 2.26E-17 | 0.108157 | 0.216 | 0.237 | 1.25E-12 |
| Nfatc1 | 3.47E-10 | 0.107966 | 0.145 | 0.155 | 1.92E-05 |
| Fau | 1.36E-14 | 0.107663 | 0.995 | 0.995 | 7.53E-10 |
| Smarce1 | 5.88E-18 | 0.107556 | 0.229 | 0.249 | 3.26E-13 |
| Pea15a | 3.14E-12 | 0.107485 | 0.421 | 0.433 | 1.74E-07 |
| Rpl24 | 4.15E-13 | 0.107321 | 0.723 | 0.729 | 2.30E-08 |
| Creb1 | 6.24E-09 | 0.107165 | 0.273 | 0.282 | 0.000346 |
| Spin1 | 1.61E-11 | 0.107036 | 0.183 | 0.194 | 8.91E-07 |
| Cdk11b | 3.75E-17 | 0.106795 | 0.307 | 0.326 | 2.08E-12 |
| Ccs | 5.38E-18 | 0.10676 | 0.321 | 0.339 | 2.98E-13 |
| Itpr2 | 8.55E-20 | 0.106739 | 0.263 | 0.289 | 4.74E-15 |
| Naca | 6.62E-13 | 0.106465 | 0.977 | 0.98 | 3.67E-08 |
| Dennd2a | 4.06E-07 | 0.106176 | 0.125 | 0.131 | 0.022496 |
| Srsf10 | 3.44E-14 | 0.106157 | 0.258 | 0.272 | 1.91E-09 |
| Nsmf | 7.47E-21 | 0.105662 | 0.135 | 0.157 | 4.14E-16 |
| Hic1 | 1.23E-07 | 0.105519 | 0.237 | 0.245 | 0.006802 |
| Cox20 | 2.57E-17 | 0.105123 | 0.411 | 0.43 | 1.43E-12 |
| Map1lc3b | 1.00E-09 | 0.104977 | 0.808 | 0.808 | 5.57E-05 |
| Fxyd1 | 2.54E-11 | 0.104543 | 0.833 | 0.843 | 1.41E-06 |
| L3mbtl3 | 2.62E-08 | 0.104458 | 0.094 | 0.103 | 0.001449 |
| Mtmr1 | 2.95E-10 | 0.104442 | 0.116 | 0.127 | 1.64E-05 |
| Sned1 | 3.79E-10 | 0.104262 | 0.418 | 0.44 | 2.10E-05 |
| Dkc1 | 4.16E-10 | 0.10425 | 0.206 | 0.215 | 2.30E-05 |
| Map3k3 | 8.27E-11 | 0.104237 | 0.164 | 0.175 | 4.58E-06 |
| Hdac1 | 7.69E-10 | 0.104064 | 0.289 | 0.297 | 4.26E-05 |
| Crtc3 | 5.36E-12 | 0.103812 | 0.215 | 0.23 | 2.97E-07 |
| Rcor3 | 2.78E-12 | 0.103781 | 0.187 | 0.2 | 1.54E-07 |
| Tshz2 | 2.24E-09 | 0.103678 | 0.471 | 0.487 | 0.000124 |
| Arfgef1 | 6.44E-10 | 0.103536 | 0.253 | 0.261 | 3.57E-05 |
| Rtn4rl1 | 4.17E-08 | 0.103459 | 0.177 | 0.188 | 0.002311 |
| Rpl22 | 4.48E-13 | 0.103322 | 0.99 | 0.992 | 2.48E-08 |
| N4bp1 | 9.13E-08 | 0.103221 | 0.138 | 0.146 | 0.005057 |
| Cdr2l | 3.68E-15 | 0.103201 | 0.132 | 0.15 | 2.04E-10 |
| Med4 | 9.32E-09 | 0.10302 | 0.125 | 0.133 | 0.000516 |
| Prkra | 9.68E-12 | 0.10302 | 0.174 | 0.186 | 5.36E-07 |
| Bzw1 | 1.15E-09 | 0.102844 | 0.576 | 0.582 | 6.38E-05 |
| Rpl10a | 2.38E-11 | 0.102683 | 0.967 | 0.969 | 1.32E-06 |
| Ldlrap1 | 6.23E-10 | 0.102578 | 0.096 | 0.106 | 3.45E-05 |
| Iws1 | 4.23E-09 | 0.102533 | 0.174 | 0.184 | 0.000234 |
| Smad1 | 4.83E-15 | 0.102045 | 0.236 | 0.255 | 2.67E-10 |
| Git1 | 2.29E-15 | 0.101942 | 0.176 | 0.19 | 1.27E-10 |

| | | | | | |
|---|---|---|---|---|---|
| Samhd1 | 2.72E-12 | 0.101871 | 0.273 | 0.288 | 1.51E-07 |
| Mrrf | 4.14E-08 | 0.101863 | 0.111 | 0.119 | 0.002293 |
| Pdcl3 | 3.69E-15 | 0.101654 | 0.464 | 0.479 | 2.05E-10 |
| Rpl36 | 4.27E-09 | 0.101377 | 0.982 | 0.982 | 0.000237 |
| Nolc1 | 2.34E-15 | 0.101369 | 0.257 | 0.277 | 1.29E-10 |
| Prdx6 | 1.30E-15 | 0.101109 | 0.476 | 0.492 | 7.21E-11 |
| Fbxo6 | 8.29E-12 | 0.10108 | 0.182 | 0.194 | 4.59E-07 |
| Abca5 | 1.48E-10 | 0.101069 | 0.153 | 0.166 | 8.18E-06 |
| Dync2li1 | 2.43E-07 | 0.101056 | 0.184 | 0.192 | 0.013484 |
| Bhlhe40 | 7.17E-13 | 0.101007 | 0.469 | 0.494 | 3.98E-08 |
| Sh3d19 | 8.64E-19 | 0.100974 | 0.475 | 0.499 | 4.79E-14 |
| Zranb1 | 5.67E-14 | 0.100701 | 0.277 | 0.297 | 3.14E-09 |
| Slc39a10 | 5.01E-12 | 0.100685 | 0.172 | 0.186 | 2.78E-07 |
| Snx21 | 7.35E-13 | 0.10066 | 0.206 | 0.221 | 4.07E-08 |
| Gm10076 | 1.37E-15 | 0.100522 | 0.631 | 0.65 | 7.61E-11 |
| Osbpl8 | 5.56E-11 | 0.100468 | 0.186 | 0.199 | 3.08E-06 |
| Kcmf1 | 2.28E-26 | 0.100438 | 0.347 | 0.379 | 1.27E-21 |
| Epc2 | 8.59E-14 | 0.100226 | 0.276 | 0.29 | 4.76E-09 |
| Gng5 | 4.60E-17 | 0.100131 | 0.632 | 0.653 | 2.55E-12 |
| Ints8 | 3.83E-08 | 0.100105 | 0.109 | 0.117 | 0.00212 |

| Pre-Obs_DOWN | | | | | |
|---|---|---|---|---|---|
| Gene | p_val | avg_log2FC | pct.1 | pct.2 | p_val_adj |
| Pvalb | 1.32E-12 | -4.480665394 | 0.005 | 0.106 | 7.33E-08 |
| Ckm | 8.09E-29 | -3.834391988 | 0.027 | 0.275 | 4.49E-24 |
| Mylpf | 9.20E-53 | -3.547493783 | 0.064 | 0.484 | 5.10E-48 |
| Myl1 | 7.32E-36 | -3.484030831 | 0.049 | 0.362 | 4.05E-31 |
| Tnnc2 | 1.21E-45 | -3.364893669 | 0.072 | 0.46 | 6.70E-41 |
| Tnni2 | 6.64E-33 | -3.24060349 | 0.049 | 0.348 | 3.68E-28 |
| Atp2a1 | 4.15E-09 | -3.232240296 | 0.012 | 0.1 | 0.00023 |
| Acta1 | 4.48E-64 | -3.208507481 | 0.119 | 0.617 | 2.48E-59 |
| Tnnt3 | 6.23E-37 | -3.131721944 | 0.059 | 0.392 | 3.45E-32 |
| Tcap | 4.99E-09 | -2.961618328 | 0.017 | 0.111 | 0.000277 |
| Col10a1 | 4.38E-09 | -2.891289012 | 0.015 | 0.106 | 0.000243 |
| Hbb-bt | 1.26E-36 | -1.328189431 | 0.19 | 0.584 | 6.97E-32 |
| Hbb-bs | 1.20E-77 | -1.259372696 | 0.494 | 0.938 | 6.64E-73 |
| Hba-a1 | 6.64E-44 | -1.25006928 | 0.207 | 0.644 | 3.68E-39 |
| Tnfrsf19 | 1.71E-07 | -1.019478584 | 0.232 | 0.377 | 0.009461 |
| Prg4 | 1.13E-12 | -0.96178795 | 0.509 | 0.709 | 6.26E-08 |
| Plac9a | 3.60E-07 | -0.792557251 | 0.21 | 0.367 | 0.019969 |
| Hba-a2 | 1.70E-15 | -0.786032764 | 0.156 | 0.388 | 9.41E-11 |
| Alpl | 5.17E-10 | -0.683551882 | 0.536 | 0.717 | 2.86E-05 |
| Atp5mpl | 2.71E-08 | -0.616050511 | 0.533 | 0.655 | 0.001501 |
| mt-Nd1 | 1.26E-17 | -0.550301506 | 0.995 | 0.998 | 6.97E-13 |
| mt-Nd2 | 2.44E-18 | -0.53065348 | 0.998 | 0.997 | 1.35E-13 |
| Ndufs6 | 8.07E-07 | -0.513462747 | 0.528 | 0.59 | 0.044746 |
| mt-Nd4 | 3.87E-16 | -0.49756161 | 0.998 | 0.997 | 2.14E-11 |
| Col2a1 | 1.37E-10 | -0.449868473 | 0.254 | 0.448 | 7.59E-06 |
| mt-Cytb | 5.66E-13 | -0.393124329 | 1 | 1 | 3.14E-08 |
| Atp5e | 6.04E-07 | -0.338146325 | 0.928 | 0.949 | 0.033482 |

| OsteoX_DOWN | | | | | |
|---|---|---|---|---|---|
| Gene | p_val | avg_log2FC | pct.1 | pct.2 | p_val_adj |
| Car2 | 5.05E-37 | -4.273782591 | 0.007 | 0.134 | 2.80E-32 |
| Myl1 | 5.93E-80 | -3.337944828 | 0.044 | 0.338 | 3.29E-75 |
| Tnni2 | 3.67E-69 | -3.282372084 | 0.047 | 0.315 | 2.03E-64 |
| Mylpf | 2.25E-111 | -3.270653387 | 0.074 | 0.465 | 1.25E-106 |
| Tnnc2 | 1.76E-95 | -3.246128835 | 0.06 | 0.407 | 9.76E-91 |
| Ckm | 1.42E-57 | -3.20858937 | 0.034 | 0.258 | 7.86E-53 |
| Pvalb | 7.38E-25 | -2.893596293 | 0.023 | 0.136 | 4.09E-20 |
| Tnnt3 | 9.05E-65 | -2.610736092 | 0.058 | 0.327 | 5.02E-60 |
| Acta1 | 5.10E-94 | -2.328728757 | 0.152 | 0.533 | 2.83E-89 |
| Eno3 | 1.46E-16 | -2.232394022 | 0.034 | 0.124 | 8.08E-12 |
| Cd24a | 7.49E-15 | -2.001630927 | 0.023 | 0.102 | 4.15E-10 |
| Col2a1 | 4.76E-41 | -1.934746576 | 0.072 | 0.278 | 2.64E-36 |
| H1f4 | 1.94E-10 | -1.422782352 | 0.038 | 0.108 | 1.07E-05 |
| Mir6236 | 6.75E-17 | -1.391732994 | 0.857 | 0.871 | 3.74E-12 |
| Rarres1 | 1.35E-10 | -1.239739079 | 0.072 | 0.156 | 7.46E-06 |
| Plac9a | 1.40E-25 | -1.197721956 | 0.29 | 0.487 | 7.78E-21 |
| Col6a3 | 4.05E-21 | -1.119264034 | 0.276 | 0.447 | 2.25E-16 |
| Dpt | 1.14E-08 | -1.065145797 | 0.103 | 0.186 | 0.000631 |
| Ppp2cb | 7.11E-08 | -1.051492674 | 0.06 | 0.128 | 0.003939 |
| Col6a2 | 3.88E-57 | -1.001760532 | 0.701 | 0.852 | 2.15E-52 |
| Atp8b2 | 2.68E-07 | -0.991964285 | 0.087 | 0.157 | 0.01486 |
| Fign | 1.61E-07 | -0.960038034 | 0.092 | 0.165 | 0.008923 |
| Hbb-bt | 1.93E-157 | -0.912977019 | 0.114 | 0.621 | 1.07E-152 |
| Wtip | 3.61E-12 | -0.875515711 | 0.124 | 0.236 | 2.00E-07 |
| Col6a1 | 5.28E-54 | -0.871303626 | 0.792 | 0.91 | 2.93E-49 |
| Fgf13 | 4.83E-08 | -0.869675465 | 0.124 | 0.207 | 0.002676 |
| Megf6 | 8.92E-09 | -0.811252257 | 0.197 | 0.277 | 0.000494 |
| 4632427E1 | 2.19E-07 | -0.808620611 | 0.055 | 0.112 | 0.012131 |
| Lars2 | 6.58E-13 | -0.736623561 | 0.515 | 0.598 | 3.65E-08 |
| Rn18s | 1.25E-12 | -0.694139722 | 1 | 1 | 6.94E-08 |
| Acan | 4.66E-13 | -0.649138722 | 0.438 | 0.531 | 2.58E-08 |
| Hspa1b | 4.31E-07 | -0.630232215 | 0.537 | 0.626 | 0.023893 |
| Cd109 | 7.61E-09 | -0.613176425 | 0.276 | 0.385 | 0.000422 |
| S100a4 | 5.79E-07 | -0.604087758 | 0.333 | 0.431 | 0.032087 |
| Crispld2 | 9.47E-16 | -0.601025186 | 0.568 | 0.693 | 5.25E-11 |
| Mbd2 | 4.84E-12 | -0.593706179 | 0.327 | 0.465 | 2.68E-07 |
| Sdc3 | 1.66E-12 | -0.580813201 | 0.456 | 0.586 | 9.22E-08 |
| Hnrnpa0 | 9.01E-19 | -0.568130381 | 0.511 | 0.665 | 4.99E-14 |
| Prelp | 6.11E-08 | -0.550194024 | 0.195 | 0.294 | 0.003385 |
| Ibsp | 1.69E-12 | -0.528786279 | 0.54 | 0.679 | 9.35E-08 |
| Ybx3 | 6.87E-12 | -0.515058732 | 0.455 | 0.581 | 3.81E-07 |
| Bgn | 1.33E-14 | -0.495245417 | 0.625 | 0.735 | 7.39E-10 |
| Itga10 | 3.65E-08 | -0.494059029 | 0.177 | 0.271 | 0.002025 |
| Ryk | 1.26E-11 | -0.480471429 | 0.421 | 0.557 | 7.00E-07 |
| Hba-a1 | 1.11E-194 | -0.478875533 | 0.131 | 0.701 | 6.14E-190 |

| | | | | | |
|---|---|---|---|---|---|
| Hbb-bs | 1.33E-275 | -0.478807516 | 0.415 | 0.96 | 7.37E-271 |
| 2310009B1 | 2.61E-07 | -0.473028406 | 0.216 | 0.313 | 0.014477 |
| Ptgfrn | 8.34E-07 | -0.451286306 | 0.364 | 0.459 | 0.046201 |
| Nfix | 6.24E-07 | -0.449093191 | 0.422 | 0.51 | 0.034594 |
| Pmepa1 | 7.58E-11 | -0.433338222 | 0.694 | 0.761 | 4.20E-06 |
| Colgalt1 | 4.05E-08 | -0.432862524 | 0.425 | 0.53 | 0.002246 |
| Tsc22d3 | 2.56E-10 | -0.427502842 | 0.661 | 0.766 | 1.42E-05 |
| Eef1a1 | 1.06E-11 | -0.423971395 | 0.689 | 0.771 | 5.85E-07 |
| Aqp1 | 3.72E-09 | -0.418696345 | 0.635 | 0.724 | 0.000206 |
| Tnc | 2.15E-13 | -0.410501206 | 0.874 | 0.913 | 1.19E-08 |
| Myo10 | 5.45E-07 | -0.401129691 | 0.364 | 0.469 | 0.030214 |
| Tmem119 | 8.67E-08 | -0.396114019 | 0.519 | 0.612 | 0.004806 |
| Col5a1 | 7.08E-13 | -0.3779875 | 0.818 | 0.883 | 3.92E-08 |
| H2bc4 | 1.33E-07 | -0.361474077 | 0.378 | 0.489 | 0.007362 |
| Serpinh1 | 3.85E-26 | -0.354087547 | 0.985 | 0.995 | 2.14E-21 |
| Sec31a | 5.94E-08 | -0.344896441 | 0.581 | 0.674 | 0.003291 |
| Txndc5 | 7.14E-08 | -0.344385741 | 0.535 | 0.63 | 0.003956 |
| Aldoa | 2.59E-07 | -0.337806583 | 0.672 | 0.751 | 0.014372 |
| Loxl1 | 3.85E-07 | -0.336034423 | 0.662 | 0.752 | 0.021324 |
| Pth1r | 3.50E-11 | -0.330604781 | 0.843 | 0.906 | 1.94E-06 |
| Col8a2 | 4.99E-08 | -0.324146435 | 0.609 | 0.715 | 0.002765 |
| Fkbp9 | 4.94E-08 | -0.31701084 | 0.668 | 0.753 | 0.002738 |
| Surf4 | 8.16E-07 | -0.313774311 | 0.56 | 0.65 | 0.045239 |
| Ssr4 | 6.53E-08 | -0.284065455 | 0.805 | 0.857 | 0.00362 |
| P4hb | 5.93E-07 | -0.271541603 | 0.678 | 0.762 | 0.032861 |
| Fmod | 1.46E-17 | -0.260837677 | 0.879 | 0.953 | 8.07E-13 |
| Fn1 | 2.55E-09 | -0.224012417 | 0.884 | 0.939 | 0.000141 |
| Col16a1 | 8.34E-09 | -0.219944384 | 0.871 | 0.935 | 0.000462 |
| Rcn3 | 3.06E-07 | -0.219680471 | 0.96 | 0.978 | 0.016954 |
| Hba-a2 | 4.56E-66 | -0.219113807 | 0.113 | 0.408 | 2.53E-61 |
| Serpine2 | 8.51E-07 | -0.204158031 | 0.717 | 0.804 | 0.047153 |
| Igfbp7 | 1.66E-09 | -0.194604206 | 0.772 | 0.867 | 9.19E-05 |
| Rps17 | 6.54E-07 | -0.181762698 | 0.935 | 0.964 | 0.036262 |
| Hsp90b1 | 7.20E-08 | -0.180847153 | 0.928 | 0.969 | 0.00399 |

| Fibro-1_DOWN | | | | | |
|---|---|---|---|---|---|
| Gene | p_val | avg_log2FC | pct.1 | pct.2 | p_val_adj |
| Ckm | 3.57E-141 | -3.227814565 | 0.035 | 0.275 | 1.98E-136 |
| Mylpf | 2.67E-234 | -3.187986044 | 0.078 | 0.453 | 1.48E-229 |
| Tnnc2 | 4.48E-199 | -2.967414756 | 0.063 | 0.398 | 2.48E-194 |
| Myl1 | 3.73E-165 | -2.961243956 | 0.054 | 0.342 | 2.07E-160 |
| Tnni2 | 1.27E-146 | -2.832199188 | 0.048 | 0.309 | 7.04E-142 |
| Col10a1 | 2.76E-47 | -2.751873953 | 0.017 | 0.112 | 1.53E-42 |
| Acta1 | 8.10E-245 | -2.749744793 | 0.108 | 0.514 | 4.49E-240 |
| Col2a1 | 1.87E-107 | -2.629658257 | 0.046 | 0.255 | 1.04E-102 |
| Tnnt3 | 1.47E-135 | -2.599678099 | 0.071 | 0.335 | 8.12E-131 |
| Car2 | 5.93E-18 | -1.039440716 | 0.062 | 0.134 | 3.29E-13 |
| Eno3 | 6.18E-10 | -1.01307829 | 0.061 | 0.109 | 3.42E-05 |
| Cox4i2 | 2.30E-09 | -1.003237381 | 0.064 | 0.112 | 0.000128 |
| C4b | 1.92E-18 | -0.949720197 | 0.127 | 0.218 | 1.06E-13 |
| Hspa1a | 3.67E-17 | -0.946971283 | 0.648 | 0.711 | 2.03E-12 |
| Dpt | 7.02E-34 | -0.939415128 | 0.237 | 0.386 | 3.89E-29 |
| Hbb-bt | 1.18E-156 | -0.910698733 | 0.26 | 0.618 | 6.54E-152 |
| Plac9a | 1.03E-119 | -0.896853679 | 0.82 | 0.935 | 5.72E-115 |
| Smpd3 | 1.97E-10 | -0.875950067 | 0.08 | 0.137 | 1.09E-05 |
| Pamr1 | 2.00E-07 | -0.869523875 | 0.072 | 0.114 | 0.011095 |
| Vcan | 4.91E-13 | -0.857759949 | 0.073 | 0.136 | 2.72E-08 |
| Hba-a1 | 1.60E-172 | -0.836010876 | 0.315 | 0.693 | 8.88E-168 |
| Hspa1b | 2.35E-23 | -0.780166285 | 0.491 | 0.601 | 1.30E-18 |
| Hbb-bs | 2.59E-301 | -0.778289832 | 0.562 | 0.951 | 1.43E-296 |
| Acan | 7.19E-18 | -0.759434119 | 0.175 | 0.276 | 3.99E-13 |
| Hba-a2 | 1.71E-45 | -0.736451979 | 0.213 | 0.392 | 9.50E-41 |
| Cd248 | 4.38E-11 | -0.711710076 | 0.13 | 0.198 | 2.43E-06 |
| Ssc5d | 4.25E-14 | -0.709239069 | 0.171 | 0.256 | 2.35E-09 |
| Sulf2 | 3.47E-08 | -0.699468334 | 0.115 | 0.167 | 0.001923 |
| Heg1 | 2.35E-07 | -0.695778703 | 0.066 | 0.108 | 0.013 |
| Gm48565 | 1.57E-11 | -0.695353431 | 0.113 | 0.18 | 8.70E-07 |
| Alpl | 5.98E-27 | -0.670637744 | 0.366 | 0.505 | 3.31E-22 |
| C1qtnf6 | 1.17E-14 | -0.667069433 | 0.16 | 0.247 | 6.47E-10 |
| Cd276 | 4.91E-08 | -0.646974662 | 0.126 | 0.179 | 0.002722 |
| Mamdc2 | 2.24E-07 | -0.630843954 | 0.115 | 0.167 | 0.012393 |
| Tmem254a | 1.05E-23 | -0.625316961 | 0.354 | 0.475 | 5.80E-19 |
| Cdk6 | 2.23E-07 | -0.618223379 | 0.082 | 0.128 | 0.012333 |
| Large1 | 2.66E-07 | -0.60496937 | 0.072 | 0.115 | 0.014756 |
| Col6a2 | 2.33E-54 | -0.604890299 | 0.673 | 0.813 | 1.29E-49 |
| Gm8696 | 6.59E-07 | -0.592697447 | 0.114 | 0.164 | 0.03653 |
| C3 | 1.06E-09 | -0.589933155 | 0.121 | 0.184 | 5.87E-05 |
| Ly6a | 3.36E-21 | -0.572944744 | 0.375 | 0.499 | 1.86E-16 |
| Fbln7 | 7.62E-13 | -0.560437192 | 0.324 | 0.416 | 4.22E-08 |
| Sned1 | 3.80E-12 | -0.545539102 | 0.104 | 0.17 | 2.11E-07 |
| Pth1r | 6.54E-22 | -0.539193724 | 0.522 | 0.634 | 3.63E-17 |
| Npy | 1.22E-08 | -0.537561684 | 0.074 | 0.122 | 0.000675 |

| | | | | | |
|---|---|---|---|---|---|
| Bglap | 3.65E-17 | -0.53505284 | 0.476 | 0.594 | 2.02E-12 |
| Ttyh3 | 7.38E-07 | -0.526788037 | 0.123 | 0.175 | 0.040875 |
| Itga10 | 9.90E-11 | -0.525933781 | 0.188 | 0.266 | 5.49E-06 |
| Tnxb | 8.02E-14 | -0.520608569 | 0.157 | 0.241 | 4.45E-09 |
| Srpx2 | 3.06E-11 | -0.516811709 | 0.245 | 0.329 | 1.69E-06 |
| Fn1 | 1.32E-43 | -0.509263224 | 0.836 | 0.92 | 7.34E-39 |
| Gdf10 | 1.10E-13 | -0.509182016 | 0.364 | 0.459 | 6.11E-09 |
| P4ha3 | 1.14E-07 | -0.507490016 | 0.147 | 0.205 | 0.006323 |
| Tmem119 | 3.01E-24 | -0.496645517 | 0.565 | 0.665 | 1.67E-19 |
| Tgfbi | 1.30E-08 | -0.496015609 | 0.228 | 0.298 | 0.000723 |
| Aqp1 | 5.74E-25 | -0.495231144 | 0.578 | 0.697 | 3.18E-20 |
| Fhl1 | 7.19E-14 | -0.487380754 | 0.41 | 0.504 | 3.99E-09 |
| Postn | 2.18E-07 | -0.486280423 | 0.244 | 0.312 | 0.012066 |
| Sp7 | 7.88E-09 | -0.483051594 | 0.165 | 0.231 | 0.000436 |
| Pkdcc | 7.17E-08 | -0.469254383 | 0.172 | 0.235 | 0.003975 |
| Creb5 | 3.53E-10 | -0.458429084 | 0.116 | 0.176 | 1.96E-05 |
| Bmp1 | 2.39E-10 | -0.455277884 | 0.248 | 0.328 | 1.32E-05 |
| Sparcl1 | 9.26E-09 | -0.454672355 | 0.156 | 0.22 | 0.000513 |
| Ccn5 | 7.12E-10 | -0.45401852 | 0.218 | 0.295 | 3.94E-05 |
| Angptl2 | 2.52E-10 | -0.453786914 | 0.363 | 0.434 | 1.40E-05 |
| P4ha2 | 7.13E-10 | -0.449951925 | 0.198 | 0.273 | 3.95E-05 |
| Bglap2 | 6.20E-08 | -0.444470764 | 0.469 | 0.548 | 0.003438 |
| Col3a1 | 4.01E-35 | -0.443263336 | 0.923 | 0.962 | 2.22E-30 |
| Marveld1 | 1.35E-07 | -0.436223527 | 0.237 | 0.302 | 0.007506 |
| Htra3 | 1.13E-10 | -0.431381965 | 0.346 | 0.425 | 6.24E-06 |
| Col6a1 | 7.38E-37 | -0.429994799 | 0.851 | 0.907 | 4.09E-32 |
| Ak1 | 4.93E-09 | -0.424093253 | 0.291 | 0.365 | 0.000273 |
| Ddit4l | 1.60E-08 | -0.421956655 | 0.185 | 0.252 | 0.000887 |
| Hmcn1 | 4.02E-07 | -0.413901109 | 0.112 | 0.161 | 0.022304 |
| Slc27a3 | 1.80E-07 | -0.410685688 | 0.238 | 0.304 | 0.009996 |
| Enpp1 | 1.40E-07 | -0.410564414 | 0.24 | 0.308 | 0.007746 |
| Celf2 | 7.20E-08 | -0.407283383 | 0.142 | 0.199 | 0.003992 |
| Ifi204 | 2.11E-08 | -0.405220542 | 0.154 | 0.216 | 0.00117 |
| Cilp | 3.34E-10 | -0.403621271 | 0.323 | 0.406 | 1.85E-05 |
| Dbi | 1.60E-18 | -0.399900099 | 0.595 | 0.683 | 8.86E-14 |
| Ifi207 | 1.46E-08 | -0.396042748 | 0.113 | 0.165 | 0.000808 |
| Uqcc2 | 1.50E-24 | -0.394893177 | 0.655 | 0.752 | 8.29E-20 |
| Lgmn | 2.60E-07 | -0.394315098 | 0.187 | 0.248 | 0.014384 |
| Mef2c | 1.66E-11 | -0.393897601 | 0.364 | 0.458 | 9.21E-07 |
| Cdh13 | 2.49E-07 | -0.388899974 | 0.237 | 0.303 | 0.013822 |
| Loxl1 | 7.80E-13 | -0.388771816 | 0.501 | 0.577 | 4.32E-08 |
| Tppp3 | 1.45E-12 | -0.386918887 | 0.226 | 0.315 | 8.01E-08 |
| Col5a1 | 9.19E-21 | -0.38631623 | 0.631 | 0.729 | 5.09E-16 |
| Tpm1 | 2.57E-17 | -0.383776157 | 0.695 | 0.783 | 1.43E-12 |
| S100a4 | 1.86E-17 | -0.378880367 | 0.561 | 0.668 | 1.03E-12 |
| Ecm1 | 7.36E-20 | -0.373024738 | 0.587 | 0.704 | 4.08E-15 |
| Mif | 4.54E-09 | -0.369025951 | 0.557 | 0.615 | 0.000252 |

| | | | | | |
|---|---|---|---|---|---|
| Snai2 | 1.84E-10 | -0.3627394 | 0.269 | 0.353 | 1.02E-05 |
| 2310039H( | 6.19E-07 | -0.358742314 | 0.199 | 0.259 | 0.034278 |
| Olfml2b | 5.77E-13 | -0.354439442 | 0.556 | 0.635 | 3.20E-08 |
| Sh3pxd2a | 2.10E-11 | -0.350518074 | 0.37 | 0.464 | 1.16E-06 |
| Aspn | 2.95E-11 | -0.347269189 | 0.879 | 0.897 | 1.63E-06 |
| mt-Nd3 | 7.19E-12 | -0.345369772 | 0.558 | 0.639 | 3.99E-07 |
| Thbs3 | 8.39E-09 | -0.344592324 | 0.381 | 0.459 | 0.000465 |
| Cd44 | 3.62E-10 | -0.342805781 | 0.279 | 0.363 | 2.00E-05 |
| Timp1 | 2.21E-15 | -0.342513614 | 0.511 | 0.62 | 1.22E-10 |
| Tnc | 5.10E-09 | -0.338962756 | 0.555 | 0.622 | 0.000282 |
| mt-Nd1 | 6.17E-33 | -0.338904488 | 0.999 | 0.998 | 3.42E-28 |
| Kctd12 | 4.78E-08 | -0.331334626 | 0.522 | 0.588 | 0.002651 |
| Ptprs | 1.25E-10 | -0.330557926 | 0.486 | 0.563 | 6.94E-06 |
| Spp1 | 1.82E-17 | -0.328221888 | 0.403 | 0.519 | 1.01E-12 |
| Sh3pxd2b | 3.09E-07 | -0.328119912 | 0.278 | 0.347 | 0.017146 |
| Ibsp | 6.44E-53 | -0.324216871 | 0.384 | 0.584 | 3.57E-48 |
| Serpinh1 | 9.37E-41 | -0.324174598 | 0.96 | 0.977 | 5.19E-36 |
| Serpine2 | 1.51E-20 | -0.32235209 | 0.657 | 0.765 | 8.36E-16 |
| Cpe | 3.37E-09 | -0.321473467 | 0.287 | 0.366 | 0.000187 |
| Uqcrq | 6.89E-14 | -0.32052632 | 0.548 | 0.643 | 3.82E-09 |
| Ap1s2 | 2.90E-07 | -0.318820202 | 0.239 | 0.305 | 0.016067 |
| Josd2 | 3.50E-07 | -0.318559742 | 0.217 | 0.279 | 0.019381 |
| 7SK.293 | 3.25E-13 | -0.317525727 | 0.282 | 0.377 | 1.80E-08 |
| Ntn1 | 2.40E-07 | -0.312334428 | 0.183 | 0.241 | 0.01328 |
| Gpc1 | 4.49E-08 | -0.311431403 | 0.504 | 0.57 | 0.002486 |
| Islr | 2.61E-11 | -0.308557413 | 0.701 | 0.743 | 1.45E-06 |
| Nucb1 | 8.42E-12 | -0.308213672 | 0.569 | 0.648 | 4.67E-07 |
| Crip1 | 3.33E-14 | -0.30801919 | 0.955 | 0.972 | 1.85E-09 |
| Trps1 | 8.66E-12 | -0.307516948 | 0.517 | 0.606 | 4.80E-07 |
| Capg | 4.99E-10 | -0.306544047 | 0.529 | 0.601 | 2.77E-05 |
| Aldoa | 3.41E-17 | -0.302975796 | 0.774 | 0.827 | 1.89E-12 |
| Col1a1 | 3.69E-17 | -0.295165906 | 0.987 | 0.994 | 2.04E-12 |
| Emilin1 | 5.31E-07 | -0.291347342 | 0.417 | 0.488 | 0.02945 |
| Fkbp10 | 2.17E-08 | -0.290774298 | 0.488 | 0.551 | 0.001203 |
| Hspg2 | 2.20E-10 | -0.28968209 | 0.575 | 0.625 | 1.22E-05 |
| Ybx3 | 1.28E-11 | -0.287138888 | 0.603 | 0.668 | 7.09E-07 |
| Surf4 | 5.10E-09 | -0.285004297 | 0.516 | 0.59 | 0.000283 |
| Calr | 3.89E-27 | -0.284740745 | 0.916 | 0.943 | 2.16E-22 |
| Col5a2 | 3.91E-18 | -0.281552238 | 0.836 | 0.884 | 2.17E-13 |
| Ppic | 2.07E-16 | -0.280675892 | 0.739 | 0.807 | 1.15E-11 |
| Ifitm3 | 2.22E-18 | -0.278888845 | 0.872 | 0.917 | 1.23E-13 |
| P4hb | 5.44E-12 | -0.276734996 | 0.644 | 0.701 | 3.01E-07 |
| Fgfr1 | 1.72E-10 | -0.273793312 | 0.719 | 0.774 | 9.54E-06 |
| Lgals1 | 1.95E-29 | -0.273766909 | 0.998 | 0.998 | 1.08E-24 |
| mt-Nd4 | 4.66E-25 | -0.271001725 | 0.995 | 0.996 | 2.58E-20 |
| Ccdc12 | 1.74E-07 | -0.270409803 | 0.516 | 0.583 | 0.009623 |
| Tmem258 | 2.54E-11 | -0.268670305 | 0.666 | 0.727 | 1.41E-06 |

| | | | | | |
|---|---|---|---|---|---|
| Ppp1ca | 6.36E-11 | -0.26437229 | 0.634 | 0.705 | 3.53E-06 |
| Mgst3 | 4.47E-07 | -0.262394459 | 0.485 | 0.557 | 0.02476 |
| mt-Nd2 | 2.51E-22 | -0.262092944 | 0.997 | 0.998 | 1.39E-17 |
| Pdlim4 | 5.99E-08 | -0.259038978 | 0.464 | 0.539 | 0.003317 |
| Fgfr2 | 8.66E-08 | -0.257538661 | 0.509 | 0.58 | 0.004801 |
| Col1a2 | 3.12E-16 | -0.253707755 | 0.998 | 0.998 | 1.73E-11 |
| Sppl3 | 8.96E-07 | -0.250056007 | 0.215 | 0.272 | 0.049645 |
| Ldha | 1.24E-10 | -0.249592223 | 0.74 | 0.786 | 6.87E-06 |
| Lpp | 4.57E-07 | -0.247532328 | 0.496 | 0.566 | 0.025304 |
| Txndc5 | 1.32E-07 | -0.246388856 | 0.529 | 0.596 | 0.007293 |
| Pltp | 7.09E-11 | -0.245164134 | 0.64 | 0.719 | 3.93E-06 |
| Atp5k | 3.10E-07 | -0.239907649 | 0.594 | 0.648 | 0.017201 |
| Sdc2 | 7.56E-08 | -0.238749494 | 0.731 | 0.774 | 0.004189 |
| Mtpn | 1.10E-08 | -0.23753781 | 0.412 | 0.494 | 0.000609 |
| Atp2b4 | 6.06E-08 | -0.236523853 | 0.297 | 0.368 | 0.003356 |
| Cox6b1 | 3.59E-11 | -0.227851406 | 0.793 | 0.837 | 1.99E-06 |
| Cox6c | 4.27E-17 | -0.22564386 | 0.884 | 0.904 | 2.36E-12 |
| Rex1bd | 1.37E-08 | -0.224475322 | 0.651 | 0.709 | 0.000758 |
| S100a13 | 2.32E-09 | -0.223659783 | 0.699 | 0.757 | 0.000128 |
| Uqcr11 | 7.59E-09 | -0.222243667 | 0.587 | 0.663 | 0.00042 |
| Mydgf | 4.33E-07 | -0.219106665 | 0.501 | 0.574 | 0.023985 |
| Pkm | 5.47E-07 | -0.216152513 | 0.766 | 0.799 | 0.030309 |
| Psmb3 | 9.75E-08 | -0.215101995 | 0.663 | 0.721 | 0.005404 |
| Cyb5r3 | 1.73E-07 | -0.212896794 | 0.728 | 0.769 | 0.009591 |
| Mmp2 | 3.46E-12 | -0.208389979 | 0.911 | 0.929 | 1.92E-07 |
| Prdx2 | 4.64E-10 | -0.20816748 | 0.794 | 0.84 | 2.57E-05 |
| Ifi27 | 7.37E-08 | -0.205248975 | 0.718 | 0.761 | 0.004086 |
| Tmsb4x | 1.05E-09 | -0.203002152 | 0.956 | 0.969 | 5.83E-05 |
| Atp5e | 1.71E-15 | -0.202770125 | 0.935 | 0.948 | 9.49E-11 |
| Tmed3 | 1.13E-09 | -0.202148588 | 0.808 | 0.834 | 6.27E-05 |
| Ccdc80 | 3.48E-09 | -0.201542038 | 0.784 | 0.815 | 0.000193 |
| Mbnl1 | 6.66E-07 | -0.200173708 | 0.813 | 0.837 | 0.036885 |
| Htra1 | 1.11E-08 | -0.199612704 | 0.85 | 0.887 | 0.000613 |
| Ssr4 | 1.37E-09 | -0.199144681 | 0.814 | 0.844 | 7.58E-05 |
| Atp5g1 | 3.42E-07 | -0.197885892 | 0.717 | 0.76 | 0.018926 |
| Dap | 1.28E-08 | -0.197104307 | 0.782 | 0.809 | 0.000711 |
| Cd109 | 4.14E-07 | -0.193634729 | 0.437 | 0.51 | 0.022915 |
| Rps27l | 4.99E-09 | -0.19354199 | 0.829 | 0.866 | 0.000277 |
| Tagln2 | 4.15E-07 | -0.191853162 | 0.745 | 0.786 | 0.023023 |
| Hnrnpa0 | 5.29E-08 | -0.191219165 | 0.58 | 0.656 | 0.002929 |
| Lum | 6.24E-22 | -0.18484147 | 0.83 | 0.914 | 3.46E-17 |
| Emp3 | 2.47E-10 | -0.184472925 | 0.952 | 0.962 | 1.37E-05 |
| Rpl39 | 1.31E-13 | -0.183585578 | 0.976 | 0.981 | 7.24E-09 |
| mt-Nd5 | 4.77E-08 | -0.183035283 | 0.907 | 0.932 | 0.002645 |
| mt-Co1 | 2.09E-11 | -0.182752265 | 0.954 | 0.965 | 1.16E-06 |
| Uqcrb | 7.43E-08 | -0.178807768 | 0.794 | 0.82 | 0.004117 |
| Myl6 | 3.63E-15 | -0.171183811 | 0.969 | 0.976 | 2.01E-10 |

| | | | | | |
|---|---|---|---|---|---|
| mt-Cytb | 1.72E-14 | -0.168375346 | 1 | 1 | 9.51E-10 |
| Sec61b | 2.12E-07 | -0.167588235 | 0.856 | 0.882 | 0.011744 |
| Rcn3 | 2.45E-08 | -0.164975596 | 0.926 | 0.931 | 0.00136 |
| Rps29 | 1.93E-07 | -0.149857015 | 0.939 | 0.948 | 0.010672 |
| Anxa2 | 2.62E-07 | -0.147729492 | 0.944 | 0.947 | 0.014514 |
| Ctsb | 2.00E-07 | -0.137025855 | 0.937 | 0.944 | 0.011065 |
| S100a6 | 2.09E-09 | -0.132728975 | 0.999 | 0.999 | 0.000116 |
| Sem1 | 2.23E-09 | -0.130704237 | 0.963 | 0.964 | 0.000123 |
| Rpl41 | 2.72E-09 | -0.128902911 | 0.996 | 0.997 | 0.000151 |
| Rpl37a | 5.66E-09 | -0.126671134 | 0.993 | 0.996 | 0.000314 |
| Ppib | 1.77E-08 | -0.126465288 | 0.976 | 0.98 | 0.00098 |
| Cd63 | 3.95E-09 | -0.119704815 | 0.99 | 0.99 | 0.000219 |
| Pcolce | 8.31E-07 | -0.119511884 | 0.964 | 0.97 | 0.046064 |
| Rps15 | 9.48E-08 | -0.111336901 | 0.99 | 0.993 | 0.005253 |
| Rps12 | 9.03E-08 | -0.104580095 | 0.997 | 0.999 | 0.005002 |

| Fibro-2_DOWN | | | | | |
|---|---|---|---|---|---|
| Gene | p_val | avg_log2FC | pct.1 | pct.2 | p_val_adj |
| Ccl8 | 0.005806 | -0.351527804 | 0.108 | 0.125 | 1 |
| Barx1 | 4.32E-06 | -0.311107201 | 0.097 | 0.124 | 0.239295 |
| Cd55 | 3.40E-06 | -0.287099072 | 0.44 | 0.475 | 0.188677 |
| Slc6a2 | 4.79E-05 | -0.272295019 | 0.1 | 0.123 | 1 |
| Pdia5 | 2.50E-06 | -0.266804881 | 0.081 | 0.103 | 0.138494 |
| Rasa3 | 1.01E-05 | -0.238004642 | 0.098 | 0.121 | 0.562336 |
| Ackr2 | 1.22E-05 | -0.237623703 | 0.086 | 0.107 | 0.67488 |
| Eln | 0.457196 | -0.236753558 | 0.173 | 0.18 | 1 |
| Mdk | 0.000249 | -0.234951569 | 0.181 | 0.209 | 1 |
| Ptch1 | 6.81E-06 | -0.227043365 | 0.099 | 0.122 | 0.377435 |
| Mmp3 | 0.000782 | -0.22492888 | 0.231 | 0.26 | 1 |
| Dtx4 | 2.05E-05 | -0.22367429 | 0.093 | 0.114 | 1 |
| Pi15 | 0.085245 | -0.218861163 | 0.153 | 0.167 | 1 |
| Ogfod3 | 2.85E-06 | -0.215032383 | 0.079 | 0.1 | 0.157789 |
| Retreg1 | 1.43E-06 | -0.21046763 | 0.1 | 0.124 | 0.079281 |
| Osr1 | 1.00E-06 | -0.196194644 | 0.255 | 0.295 | 0.055398 |
| Thbd | 1.53E-05 | -0.1912605 | 0.422 | 0.462 | 0.848813 |
| Cdo1 | 0.045839 | -0.190803777 | 0.094 | 0.107 | 1 |
| Trim32 | 1.79E-06 | -0.190446558 | 0.083 | 0.104 | 0.099135 |
| Sema3d | 0.000244 | -0.189224451 | 0.082 | 0.1 | 1 |
| Dcaf1 | 8.07E-06 | -0.186275998 | 0.129 | 0.153 | 0.447385 |
| Plxna2 | 0.002778 | -0.185736675 | 0.085 | 0.1 | 1 |
| Tsix | 4.92E-05 | -0.182688053 | 0.124 | 0.148 | 1 |
| Nrbp2 | 1.02E-05 | -0.177862656 | 0.088 | 0.107 | 0.564542 |
| Crispld1 | 0.000879 | -0.173022403 | 0.144 | 0.165 | 1 |
| Igf1 | 0.000298 | -0.167678856 | 0.725 | 0.72 | 1 |
| Adprm | 1.05E-06 | -0.166416575 | 0.082 | 0.101 | 0.058153 |
| Prkag2 | 5.23E-06 | -0.165317551 | 0.11 | 0.133 | 0.289808 |
| Comtd1 | 1.43E-06 | -0.16510245 | 0.102 | 0.124 | 0.079321 |
| Cd248 | 1.02E-06 | -0.16470959 | 0.644 | 0.679 | 0.056334 |
| Prkcd | 1.66E-06 | -0.16428112 | 0.085 | 0.104 | 0.091969 |
| Sema3c | 0.000564 | -0.159754695 | 0.477 | 0.509 | 1 |
| Gng11 | 0.001824 | -0.157269563 | 0.667 | 0.692 | 1 |
| Srgap3 | 0.000107 | -0.154717331 | 0.087 | 0.105 | 1 |
| Nhsl1 | 1.21E-06 | -0.153407941 | 0.134 | 0.159 | 0.066997 |
| Dop1b | 1.53E-06 | -0.152923838 | 0.082 | 0.101 | 0.084803 |
| Twist2 | 9.59E-07 | -0.152256577 | 0.136 | 0.162 | 0.053169 |
| Gclc | 2.38E-06 | -0.15054729 | 0.201 | 0.231 | 0.131952 |
| Gtf2f2 | 1.69E-05 | -0.147950017 | 0.084 | 0.102 | 0.936064 |
| Cyp26b1 | 0.095381 | -0.147717188 | 0.096 | 0.107 | 1 |
| Vegfd | 1.13E-05 | -0.146842315 | 0.096 | 0.115 | 0.625938 |
| Lum | 0.001762 | -0.145109538 | 0.956 | 0.954 | 1 |
| Tceal1 | 6.75E-06 | -0.143421022 | 0.107 | 0.127 | 0.374259 |
| Creb5 | 1.48E-06 | -0.14293553 | 0.553 | 0.598 | 0.082054 |
| Hmox1 | 0.017864 | -0.140306019 | 0.122 | 0.125 | 1 |

| | | | | | |
|---|---|---|---|---|---|
| Cp | 0.0029 | -0.139614191 | 0.173 | 0.195 | 1 |
| Extl2 | 1.50E-05 | -0.138160518 | 0.11 | 0.13 | 0.829313 |
| Angptl1 | 0.000146 | -0.137854379 | 0.631 | 0.611 | 1 |
| Ggt5 | 0.001244 | -0.136847501 | 0.117 | 0.135 | 1 |
| Mfap2 | 1.62E-05 | -0.135855359 | 0.368 | 0.407 | 0.899328 |
| Cxcl16 | 0.004425 | -0.135648714 | 0.119 | 0.135 | 1 |
| Fryl | 1.40E-06 | -0.135373275 | 0.112 | 0.134 | 0.077322 |
| Prss23 | 9.36E-07 | -0.135245461 | 0.559 | 0.605 | 0.051854 |
| Gorab | 1.30E-06 | -0.132945707 | 0.097 | 0.118 | 0.071769 |
| Kat5 | 1.69E-05 | -0.131334334 | 0.109 | 0.128 | 0.938278 |
| Uvssa | 2.14E-06 | -0.129007408 | 0.107 | 0.126 | 0.118516 |
| Mocs2 | 9.14E-07 | -0.12851405 | 0.244 | 0.278 | 0.050634 |
| Dynlt2b | 0.000259 | -0.127692279 | 0.11 | 0.128 | 1 |
| Cxcl12 | 0.000409 | -0.120911032 | 0.163 | 0.186 | 1 |
| Cep290 | 4.61E-06 | -0.119781695 | 0.097 | 0.117 | 0.25546 |
| Syvn1 | 2.78E-06 | -0.119416292 | 0.124 | 0.146 | 0.15396 |
| Dhx8 | 5.90E-06 | -0.118341223 | 0.087 | 0.105 | 0.327201 |
| Zfp62 | 1.34E-06 | -0.116012121 | 0.103 | 0.122 | 0.074316 |
| Gpatch1 | 2.30E-06 | -0.11489908 | 0.096 | 0.114 | 0.127205 |
| Kctd12b | 0.000102 | -0.114243337 | 0.089 | 0.104 | 1 |
| Clcn3 | 2.03E-06 | -0.114113892 | 0.091 | 0.109 | 0.112585 |
| Phactr4 | 0.001951 | -0.113704774 | 0.087 | 0.1 | 1 |
| Nrip1 | 0.000262 | -0.11299199 | 0.286 | 0.313 | 1 |
| Hps5 | 3.78E-06 | -0.112501484 | 0.084 | 0.101 | 0.209399 |
| Gapdh | 2.54E-05 | -0.109539187 | 0.753 | 0.784 | 1 |
| Siah2 | 3.93E-05 | -0.109244801 | 0.101 | 0.119 | 1 |
| Meis3 | 5.70E-05 | -0.108806229 | 0.098 | 0.114 | 1 |
| Slc23a2 | 2.57E-05 | -0.108642514 | 0.101 | 0.118 | 1 |
| Rnaseh2b | 4.82E-05 | -0.107448884 | 0.101 | 0.118 | 1 |
| H1f4 | 0.000254 | -0.107312822 | 0.103 | 0.119 | 1 |
| Nr2f2 | 1.10E-06 | -0.106685602 | 0.12 | 0.144 | 0.061111 |
| Cherp | 9.22E-07 | -0.106318598 | 0.106 | 0.125 | 0.051109 |
| Ccn3 | 0.021845 | -0.103090662 | 0.65 | 0.663 | 1 |
| Rgmb | 1.43E-05 | -0.100571913 | 0.157 | 0.18 | 0.790491 |
| Atad3a | 5.56E-06 | -0.100542959 | 0.091 | 0.108 | 0.307993 |
