# Supplementary material for: The Aging Landscape by scRNAseq of Mesenchymal Lineage Cells in Mouse Bone
Source: Aging Cell. 2025 Oct 13;24(12):e70256. doi: 10.1111/acel.70256 (PMC12686594; doi:10.1111/acel.70256)
Supplement: Supplementary file 15 — Table S5: acel70256‐sup‐0015‐TableS5.pdf. [file ACEL-24-e70256-s004.pdf]

Pre-Obs_UP					
Gene	p_val	avg_log2FC	pct.1	pct.2	p_val_adj
Hbb-bs	3.27E-10	2.807317839	0.734	0.584	1.81E-05
Ndrg1	3.18E-08	0.97576978	0.398	0.304	0.001759683
Rps27	8.26E-23	0.842089415	0.888	0.773	4.57E-18
Rpl38	1.79E-12	0.808642884	0.635	0.52	9.90E-08
Rps29	8.59E-23	0.805446531	0.859	0.778	4.76E-18
Crip1	3.88E-08	0.641501643	0.727	0.686	0.002150752
Rpl37	3.43E-23	0.626072073	0.967	0.958	1.90E-18
Rpl36	9.81E-14	0.529504121	0.964	0.929	5.44E-09
Rps21	1.54E-15	0.505462242	0.99	0.977	8.53E-11
Rpl37a	3.21E-14	0.467462648	0.98	0.981	1.78E-09
Rpl39	1.68E-10	0.463195089	0.924	0.904	9.32E-06
Rpl35	5.48E-07	0.450415951	0.845	0.788	0.03035444
Rplp2	1.02E-13	0.423232562	0.99	0.992	5.64E-09
Rpl34	7.23E-12	0.413519981	0.974	0.977	4.01E-07
Rpl30	5.31E-08	0.390300213	0.957	0.944	0.00294119
Rpl36a	8.30E-09	0.369172426	0.918	0.906	0.000459853
H2-D1	6.44E-07	0.334000472	0.714	0.78	0.035660453
Rpl35a	9.08E-09	0.31826228	0.957	0.977	0.000502941
Fau	4.36E-09	0.278710404	0.974	0.988	0.000241861

Osteo-X_UP					
Gene	p_val	avg_log2FC	pct.1	pct.2	p_val_adj
Hba-a1	4.66E-09	3.149986106	0.351	0.239	0.000258
Mmp3	6.66E-13	2.923231425	0.136	0.045	3.69E-08
Hbb-bs	2.07E-18	2.824565049	0.732	0.555	1.15E-13
Hbb-bt	4.78E-07	2.636202501	0.298	0.182	0.026516
Cyp26b1	6.09E-11	2.520309348	0.115	0.034	3.38E-06
Upk1b	3.31E-23	2.091393128	0.303	0.104	1.84E-18
Clu	1.97E-19	2.061962395	0.381	0.289	1.09E-14
Ccn2	8.08E-35	1.955770463	0.621	0.357	4.48E-30
Cpne8	2.71E-11	1.955153684	0.145	0.051	1.5E-06
Itga1	5.41E-13	1.875267878	0.189	0.063	3E-08
Fbln7	1.12E-08	1.837374732	0.155	0.069	0.00062
Thbs1	7.84E-32	1.830326932	0.564	0.346	4.34E-27
Plac9a	2.51E-22	1.805580753	0.388	0.223	1.39E-17
Fxyd6	3.9E-32	1.799816657	0.443	0.164	2.16E-27
Fst	1.2E-18	1.789025715	0.356	0.187	6.63E-14
Prelp	1.44E-15	1.749657932	0.277	0.113	8E-11
Penk	1.52E-12	1.610995934	0.275	0.151	8.41E-08
Sparcl1	3.47E-09	1.589937456	0.176	0.07	0.000192
Serpine1	1.68E-09	1.510085271	0.206	0.096	9.28E-05
Atp2b4	1.13E-09	1.498654947	0.189	0.087	6.27E-05
Apod	7.92E-19	1.417229311	0.508	0.337	4.39E-14
Gm9843	6.9E-08	1.40182892	0.162	0.076	0.003822
Slc16a2	3.88E-09	1.396789929	0.199	0.087	0.000215
S100a4	1.11E-11	1.391111626	0.303	0.172	6.13E-07
Mt2	8.87E-16	1.344127975	0.42	0.298	4.92E-11
Ndrg1	8.49E-11	1.328666318	0.259	0.123	4.7E-06
Enpp2	9.79E-09	1.285799341	0.24	0.125	0.000543
Nt5e	8.25E-08	1.233477117	0.231	0.124	0.004575
Comp	2.9E-33	1.230218195	0.905	0.874	1.6E-28
Abca8a	2.35E-07	1.201536368	0.222	0.118	0.013009
Mt1	7.25E-18	1.193693308	0.688	0.604	4.02E-13
Tagln	2.23E-08	1.186373084	0.226	0.21	0.001234
Matn4	2.48E-07	1.185155035	0.141	0.136	0.013716
Tns2	2.75E-07	1.175305591	0.208	0.105	0.015257
Npnt	3.48E-08	1.141680908	0.245	0.138	0.001926
Hes1	2.75E-12	1.125616499	0.395	0.233	1.52E-07
Thbs4	5.52E-08	1.114582349	0.286	0.16	0.00306
Zfp36	5.13E-19	1.059407582	0.647	0.452	2.84E-14
Tcf7l2	3.85E-09	1.058363712	0.266	0.166	0.000213
Dusp1	1.2E-17	1.018717051	0.591	0.435	6.65E-13
Nr4a1	1.03E-08	1.011255614	0.374	0.256	0.000571
2410006H1	4.18E-18	1.002418668	0.566	0.391	2.32E-13
Lgals3	2.14E-20	0.949259096	0.654	0.528	1.18E-15
Zfas1	1.28E-08	0.948537405	0.356	0.218	0.000708
Fosb	7.47E-14	0.925365539	0.566	0.407	4.14E-09

Rps27	2.88E-44	0.916124006	0.882	0.731	1.6E-39
Rgcc	9.84E-10	0.891275384	0.524	0.413	5.45E-05
Septin11	8.77E-08	0.868067656	0.33	0.219	0.004861
Nop53	3.14E-11	0.86267052	0.372	0.277	1.74E-06
Ahnak	1.66E-09	0.852727598	0.27	0.206	9.18E-05
Rpl38	3.79E-18	0.837488216	0.598	0.437	2.1E-13
Rpl37	7.66E-49	0.830922651	0.965	0.891	4.25E-44
Gadd45g	4.31E-15	0.826483194	0.709	0.587	2.39E-10
Zfp36l1	1.17E-27	0.800668041	0.885	0.708	6.5E-23
Samd4	7.38E-11	0.798909022	0.483	0.37	4.09E-06
Scara3	3.84E-10	0.791728126	0.427	0.354	2.13E-05
Rpl36	2.85E-40	0.788575278	0.931	0.851	1.58E-35
Klf4	5.88E-14	0.780102057	0.665	0.527	3.26E-09
Snhg8	2.12E-08	0.778030338	0.393	0.28	0.001174
Timp3	9.36E-16	0.772821992	0.813	0.719	5.19E-11
Rras	6.05E-08	0.769515632	0.397	0.287	0.003355
Fhl2	1.05E-07	0.767830282	0.342	0.271	0.0058
Ifrd1	1.06E-12	0.762638027	0.594	0.502	5.86E-08
C1ra	7E-14	0.738297647	0.577	0.458	3.88E-09
Eif4b	3.6E-07	0.735900419	0.349	0.274	0.019957
Rpl37a	6.14E-48	0.726341791	0.979	0.938	3.4E-43
Sertad1	3.99E-10	0.722644058	0.575	0.431	2.21E-05
Rps28	9.36E-12	0.721573876	0.499	0.387	5.19E-07
Cdc42ep3	5.19E-09	0.719706942	0.446	0.355	0.000287
Kirrel	1.4E-07	0.718622604	0.328	0.25	0.00776
Rps29	1.81E-24	0.709215666	0.797	0.682	1E-19
Btg2	3.77E-12	0.70491208	0.67	0.534	2.09E-07
Gsn	1.05E-22	0.699058611	0.972	0.959	5.82E-18
Rpl34	2.54E-41	0.69202017	0.984	0.929	1.41E-36
Rpl39	4.12E-32	0.691404753	0.928	0.855	2.28E-27
Fos	8.64E-14	0.686169504	0.903	0.842	4.79E-09
Nrp2	2.06E-09	0.677778973	0.596	0.459	0.000114
Zfp36l2	4.66E-09	0.671554964	0.52	0.407	0.000258
Serpine2	6.22E-11	0.667742853	0.748	0.69	3.44E-06
Egr1	2.53E-17	0.664320387	0.875	0.796	1.4E-12
Abi3bp	4.16E-13	0.660799811	0.831	0.797	2.3E-08
Rps21	2.06E-39	0.64559945	0.968	0.955	1.14E-34
Ier5	1.46E-10	0.634700469	0.624	0.501	8.09E-06
Cebpd	6.09E-07	0.633893164	0.64	0.56	0.033775
Camk2n1	2.08E-08	0.631057981	0.473	0.379	0.001152
Rpl36a	7.1E-20	0.626690625	0.882	0.762	3.93E-15
Ccn1	4.09E-11	0.622274918	0.82	0.68	2.27E-06
Gas5	1.49E-19	0.620739026	0.871	0.779	8.25E-15
Ebf1	1.28E-07	0.619815823	0.476	0.391	0.007107
Igfbp7	4.18E-11	0.618292255	0.79	0.737	2.32E-06
Gadd45b	1.45E-14	0.614269943	0.824	0.724	8.03E-10
Vim	3.67E-16	0.602927684	0.935	0.943	2.03E-11

Emp1	7.17E-09	0.598476566	0.603	0.492	0.000397
Slc38a2	4.4E-14	0.597113101	0.755	0.622	2.44E-09
Rpl22l1	3.02E-15	0.596324669	0.709	0.613	1.67E-10
Gstm1	1.67E-07	0.592646615	0.577	0.431	0.009249
Fgfr1	8.14E-10	0.588320475	0.626	0.553	4.51E-05
Syne1	1.11E-07	0.587017084	0.501	0.405	0.006175
Jun	2.33E-15	0.586236953	0.905	0.849	1.29E-10
Rpl27	1.32E-09	0.585339451	0.603	0.495	7.32E-05
Aplp2	4.24E-21	0.581240375	0.889	0.854	2.35E-16
Ttc28	1.87E-07	0.58084826	0.464	0.407	0.010336
Pltp	1.29E-09	0.576002604	0.559	0.491	7.15E-05
Cebpb	8.54E-08	0.572935057	0.64	0.541	0.004734
Rpl35a	7.27E-27	0.566986801	0.97	0.924	4.03E-22
Rpl24	1.83E-08	0.561251883	0.52	0.438	0.001017
Rpl32	1.43E-35	0.553371959	0.998	0.98	7.92E-31
Cst3	2.97E-23	0.547418776	1	0.999	1.65E-18
Anxa1	2.5E-12	0.545432	0.834	0.817	1.38E-07
Pcbp2	2.42E-09	0.535124627	0.651	0.549	0.000134
Brd2	6.03E-08	0.532540455	0.619	0.533	0.00334
Plxdc2	2.3E-09	0.515138189	0.594	0.544	0.000128
Socs3	2.23E-09	0.512663627	0.7	0.557	0.000123
Rpl30	2.86E-21	0.51150984	0.891	0.865	1.59E-16
Rps23	2.74E-23	0.509432803	0.972	0.944	1.52E-18
Rps26	2.51E-21	0.505738706	0.979	0.952	1.39E-16
Myo1d	3.28E-07	0.503398685	0.48	0.422	0.018178
Junb	4.68E-12	0.499246504	0.924	0.851	2.59E-07
Fau	1.23E-28	0.49828103	0.988	0.982	6.83E-24
Rpl22	2.15E-19	0.496261497	0.94	0.895	1.19E-14
Rps13	3.16E-25	0.495432124	0.984	0.953	1.75E-20
Cd302	5.94E-12	0.479734443	0.813	0.761	3.29E-07
Rpl17	4.96E-21	0.477614614	0.945	0.948	2.75E-16
Rps15a	8.42E-20	0.476643421	0.975	0.951	4.66E-15
Cd9	7.65E-14	0.476399691	0.896	0.887	4.24E-09
Rps20	2.2E-20	0.46777248	0.982	0.971	1.22E-15
Rpl23	5.66E-26	0.455424569	1	0.991	3.14E-21
Rpl41	6.77E-21	0.443695163	0.991	0.972	3.75E-16
Rps25	5.34E-14	0.434953683	0.824	0.8	2.96E-09
Rps18	6.7E-12	0.431820893	0.878	0.83	3.71E-07
Selenop	2.1E-07	0.424494236	0.665	0.608	0.011613
Pabpc1	8.65E-07	0.422639718	0.679	0.599	0.04792
mt-Co1	1.9E-08	0.416627241	0.905	0.836	0.001052
Rps17	2.82E-11	0.41283625	0.866	0.8	1.56E-06
Rpl35	6.47E-09	0.410474075	0.737	0.675	0.000359
Rps16	2.48E-18	0.406136333	0.991	0.979	1.38E-13
Sqstm1	7.45E-08	0.405966355	0.647	0.58	0.004131
Rplp2	2.36E-15	0.40319876	0.97	0.943	1.31E-10
Pnrc1	2.3E-07	0.397787357	0.693	0.651	0.012746

Rps19	9.94E-13	0.393285472	0.97	0.925	5.51E-08
Srsf2	3.34E-07	0.383683669	0.533	0.501	0.018519
Rpl28	8.44E-16	0.382077755	0.972	0.964	4.68E-11
Rplp1	7.87E-23	0.381592372	1	0.993	4.36E-18
Npm1	7.44E-09	0.37836242	0.864	0.821	0.000413
Colec12	1.47E-09	0.376906025	0.864	0.809	8.13E-05
Rps15	3.14E-14	0.375444927	0.979	0.972	1.74E-09
mt-Nd5	2.42E-08	0.372712609	0.866	0.822	0.001339
Rps14	2.44E-18	0.369932053	0.998	0.994	1.35E-13
Ier3	6.37E-07	0.369823016	0.901	0.82	0.035291
Pfdn5	4.91E-11	0.369002869	0.933	0.895	2.72E-06
Rps12	9.34E-18	0.364580003	0.998	0.991	5.17E-13
Rpl26	8.06E-13	0.345337875	0.968	0.959	4.47E-08
Rpl23a	4.93E-08	0.335528484	0.905	0.868	0.002732
mt-Rnr1	5.11E-14	0.334567492	1	0.997	2.83E-09
Rpl18a	6.3E-12	0.328869815	0.988	0.979	3.49E-07
Angptl4	3.87E-10	0.327809232	0.286	0.383	2.14E-05
Rps5	1.89E-14	0.323303105	1	0.99	1.05E-09
Rpl4	3.97E-07	0.319153706	0.885	0.832	0.021988
Rps24	8.16E-17	0.318624853	1	0.997	4.52E-12
Rps27a	8.35E-12	0.318122314	0.988	0.977	4.63E-07
Fmod	5.92E-07	0.303221686	0.947	0.889	0.032798
Rpl21	7.91E-14	0.29818135	0.998	0.993	4.38E-09
mt-Nd2	8.31E-08	0.297247939	0.998	0.992	0.004604
mt-Nd1	3.07E-07	0.294277809	0.986	0.991	0.01702
Rps7	5.33E-07	0.284096385	0.947	0.911	0.02951
Rpl12	7.77E-08	0.268210293	0.972	0.956	0.004306
mt-Rnr2	5.8E-10	0.221147849	1	1	3.22E-05
Rpl9	1.92E-07	0.220756991	0.998	0.994	0.010634
Tpt1	2.16E-07	0.19621966	0.995	0.997	0.011982

Fibro-1_UP					
Gene	p_val	avg_log2FC	pct.1	pct.2	p_val_adj
Dio3	3.91E-35	3.384437945	0.236	0.078	2.17E-30
Thbs1	9.50E-37	2.089475996	0.47	0.271	5.27E-32
Dio3os	1.85E-11	2.051001924	0.101	0.03	1.03E-06
Penk	2.03E-42	2.040423942	0.603	0.471	1.12E-37
Hrct1	1.63E-09	1.836356524	0.11	0.044	9.03E-05
Upk1b	8.96E-14	1.737581062	0.121	0.034	4.97E-09
Mustn1	2.61E-36	1.649118586	0.423	0.189	1.44E-31
Galnt15	8.42E-29	1.614731306	0.337	0.148	4.67E-24
Clu	2.91E-21	1.55644302	0.552	0.483	1.61E-16
C4b	3.81E-17	1.442057932	0.262	0.122	2.11E-12
Cilp	1.77E-32	1.435878279	0.504	0.294	9.82E-28
Inhbb	7.74E-11	1.363405942	0.156	0.078	4.29E-06
2310010J1	1.25E-08	1.361609875	0.129	0.066	0.000694
Gm9794	6.06E-09	1.212242998	0.163	0.088	0.000336
Serpine1	8.55E-07	1.146781598	0.132	0.069	0.047375
Angptl7	1.22E-17	1.127303129	0.381	0.408	6.76E-13
Vgll3	1.43E-07	1.121639901	0.164	0.088	0.00792
Ccn2	4.90E-33	1.118693571	0.692	0.511	2.71E-28
Stc2	5.47E-07	1.11679338	0.158	0.106	0.030299
Mitf	4.65E-10	1.060468466	0.22	0.117	2.58E-05
Aldh6a1	3.22E-07	1.055881545	0.172	0.103	0.017833
Gm26802	2.59E-07	1.023302884	0.193	0.125	0.014328
Dock11	7.41E-07	1.007523392	0.161	0.093	0.041041
Gm9843	2.57E-08	0.986121792	0.202	0.115	0.001427
Sgms2	2.18E-07	0.973218065	0.241	0.19	0.01208
Nt5e	7.41E-15	0.958036299	0.374	0.26	4.10E-10
Fst	1.22E-10	0.937457974	0.34	0.23	6.74E-06
Atf3	1.18E-09	0.924521922	0.315	0.205	6.53E-05
Gm28875	1.39E-09	0.921422953	0.251	0.158	7.69E-05
Svep1	1.68E-10	0.909835938	0.331	0.232	9.34E-06
Fosb	5.84E-16	0.906535314	0.523	0.408	3.24E-11
Adgrf5	8.86E-08	0.897227686	0.234	0.151	0.004909
Itga10	3.83E-08	0.888972818	0.223	0.133	0.002124
Rpl38	2.65E-31	0.882949659	0.671	0.479	1.47E-26
Pltp	2.92E-25	0.859066202	0.67	0.506	1.62E-20
Cdh13	1.14E-07	0.84585881	0.272	0.178	0.006335
Cyth3	6.45E-13	0.83779155	0.387	0.264	3.58E-08
Scara3	3.61E-16	0.824080272	0.55	0.44	2.00E-11
Ndrg1	6.68E-15	0.819766642	0.489	0.354	3.70E-10
Lgals3	1.79E-32	0.814873495	0.78	0.586	9.90E-28
Pik3ip1	5.34E-07	0.80780792	0.22	0.149	0.029609
Dusp1	1.85E-17	0.777222199	0.642	0.508	1.03E-12
Pcdh9	3.33E-07	0.768212922	0.287	0.205	0.018469
Ccn5	1.23E-08	0.756975354	0.289	0.183	0.000681
Cp	1.13E-10	0.74728039	0.424	0.307	6.26E-06

Mt1	8.43E-26	0.740691509	0.883	0.809	4.67E-21
Rps28	8.03E-19	0.737450483	0.593	0.435	4.45E-14
Plat	5.60E-07	0.736405037	0.324	0.257	0.031011
Rpl37	2.35E-85	0.735381955	0.976	0.932	1.30E-80
Rps27	5.76E-48	0.723284228	0.902	0.817	3.19E-43
Gabarapl1	4.83E-12	0.71783807	0.407	0.298	2.68E-07
Nr4a1	1.23E-08	0.70894805	0.482	0.381	0.00068
Rpl37a	6.92E-78	0.698092283	0.989	0.964	3.83E-73
Abca8a	6.92E-10	0.697801655	0.429	0.319	3.84E-05
Rpl39	1.32E-61	0.68059088	0.947	0.905	7.32E-57
Rpl36	1.30E-54	0.67177328	0.948	0.889	7.20E-50
Snhg8	1.51E-12	0.659718182	0.479	0.358	8.38E-08
Plac9a	5.18E-43	0.653990847	0.929	0.782	2.87E-38
Igf1r	4.39E-08	0.649945043	0.375	0.29	0.002431
Sertad1	8.14E-14	0.647422122	0.585	0.486	4.51E-09
Fos	9.64E-16	0.644210386	0.893	0.853	5.34E-11
Rps21	1.77E-71	0.643780038	0.99	0.973	9.83E-67
Mt2	3.83E-13	0.628783223	0.713	0.607	2.12E-08
Ccn1	2.99E-11	0.621088088	0.715	0.629	1.66E-06
Synm	1.72E-07	0.617765032	0.327	0.254	0.009527
Rps29	8.63E-33	0.615587392	0.853	0.76	4.78E-28
Prelp	2.69E-15	0.611585346	0.766	0.681	1.49E-10
Nuak1	2.19E-09	0.603658649	0.393	0.27	0.000121
C1ra	3.38E-12	0.594944774	0.541	0.445	1.87E-07
Septin11	2.08E-07	0.588628091	0.427	0.346	0.011547
Btg2	1.09E-13	0.586285455	0.7	0.633	6.04E-09
Myo1d	2.88E-09	0.581486749	0.481	0.382	0.00016
Cpne8	4.07E-07	0.580011198	0.385	0.28	0.022536
Sgk1	5.91E-10	0.575453694	0.561	0.5	3.28E-05
Rpl30	9.97E-37	0.559462716	0.96	0.917	5.52E-32
Serpine2	8.86E-21	0.555952198	0.801	0.66	4.91E-16
S100a4	8.06E-13	0.554164924	0.637	0.515	4.47E-08
Fn1	1.85E-10	0.553120059	0.618	0.641	1.03E-05
Rpl34	7.18E-50	0.551731923	0.982	0.947	3.98E-45
Adamts5	3.77E-09	0.543684637	0.62	0.527	0.000209
Vim	1.14E-22	0.53854182	0.96	0.939	6.33E-18
mt-Nd5	1.55E-22	0.537763067	0.895	0.815	8.61E-18
Samd4	3.94E-07	0.535419755	0.373	0.31	0.021817
C1s1	3.76E-11	0.53223692	0.589	0.498	2.08E-06
Ier2	3.84E-10	0.526352696	0.69	0.615	2.13E-05
Neat1	7.09E-11	0.522651548	0.744	0.663	3.93E-06
Zfp36	5.85E-09	0.522332955	0.552	0.463	0.000324
Socs3	2.23E-13	0.519426558	0.639	0.495	1.23E-08
Rpl35a	1.85E-40	0.516625338	0.975	0.942	1.02E-35
Cavin1	2.30E-11	0.503155872	0.699	0.602	1.28E-06
Syne1	4.92E-08	0.500256256	0.517	0.438	0.002724
2410006H1	5.34E-11	0.495067988	0.619	0.505	2.96E-06

Gadd45g	1.89E-07	0.488565113	0.706	0.625	0.010475
Ecm1	2.76E-08	0.4806962	0.555	0.445	0.001527
Rpl36a	6.05E-23	0.473160467	0.912	0.833	3.35E-18
Colec12	7.55E-17	0.468657066	0.865	0.793	4.18E-12
Rpl35	2.52E-16	0.464648895	0.774	0.698	1.39E-11
Rps26	1.76E-32	0.464463374	0.98	0.954	9.75E-28
Pls3	7.17E-07	0.458980814	0.438	0.365	0.039737
Anxa1	8.12E-21	0.450176856	0.902	0.833	4.50E-16
Zfp36l2	2.02E-08	0.449938157	0.6	0.51	0.001121
Cebpb	3.37E-07	0.444229197	0.648	0.553	0.018664
Egr1	7.17E-10	0.436042202	0.835	0.779	3.97E-05
Rpl32	1.79E-35	0.432844429	0.996	0.981	9.94E-31
Jund	9.46E-17	0.432006061	0.884	0.834	5.25E-12
Rplp2	9.16E-32	0.426993591	0.987	0.956	5.08E-27
Comp	1.64E-14	0.419288585	0.98	0.964	9.10E-10
Rpl41	1.11E-34	0.416380644	0.987	0.976	6.18E-30
Mbnl1	1.17E-09	0.413654995	0.727	0.671	6.48E-05
Klf4	2.39E-07	0.407235165	0.759	0.699	0.013223
Rps18	9.96E-19	0.402829954	0.924	0.871	5.52E-14
Emp1	3.80E-09	0.401448896	0.715	0.666	0.00021
Crip1	1.26E-08	0.400067376	0.894	0.896	0.0007
Zfp36l1	4.53E-09	0.396498934	0.842	0.779	0.000251
Aplp2	4.70E-16	0.391967396	0.892	0.852	2.60E-11
Rpl22	7.25E-22	0.387489384	0.957	0.929	4.02E-17
Cd200	2.09E-08	0.385932145	0.642	0.535	0.001156
Pnrc1	1.39E-08	0.382983073	0.763	0.681	0.000771
Pcbp2	4.45E-07	0.375688216	0.62	0.565	0.024672
Rpl31	2.94E-08	0.372175296	0.588	0.535	0.00163
Rpl17	7.45E-23	0.37167098	0.987	0.967	4.13E-18
Rps15a	4.55E-21	0.371022885	0.983	0.962	2.52E-16
Cebpd	2.84E-07	0.365463712	0.711	0.619	0.015748
Rps20	1.33E-26	0.359853855	0.987	0.98	7.39E-22
Rps23	3.95E-25	0.357635217	0.979	0.967	2.19E-20
Slc38a2	8.93E-07	0.350464249	0.726	0.67	0.049495
Rpl23	5.35E-28	0.339895885	1	0.997	2.97E-23
Rpl27a	4.84E-08	0.339766166	0.599	0.573	0.002684
mt-Rnr1	8.89E-21	0.335756172	1	0.998	4.93E-16
Gas5	1.52E-11	0.335450521	0.93	0.899	8.44E-07
Fau	1.91E-21	0.326533517	0.992	0.984	1.06E-16
Rps10	3.66E-09	0.319346326	0.841	0.814	0.000203
Rpl22l1	1.13E-09	0.317615698	0.711	0.694	6.26E-05
Cd9	1.31E-14	0.317528203	0.996	0.983	7.26E-10
Junb	1.07E-08	0.30901987	0.882	0.847	0.000593
Rps13	1.83E-16	0.305120131	0.983	0.974	1.01E-11
Rps17	8.93E-11	0.302257501	0.892	0.863	4.95E-06
Rps19	1.04E-17	0.301002112	0.985	0.942	5.76E-13
Rps24	5.72E-22	0.29013847	1	0.994	3.17E-17

Rps12	1.19E-17	0.288628871	0.997	0.995	6.57E-13
Pfdn5	5.73E-12	0.28545132	0.937	0.919	3.17E-07
Rpl23a	4.69E-08	0.272370539	0.939	0.889	0.002598
Rpl11	2.06E-13	0.265822829	0.989	0.982	1.14E-08
mt-Co1	2.54E-07	0.264548955	0.907	0.85	0.014051
Rps25	3.19E-07	0.25522159	0.893	0.86	0.017672
Rplp1	4.26E-20	0.254715318	1	1	2.36E-15
Rps15	9.64E-11	0.250356256	0.971	0.965	5.34E-06
mt-Rnr2	1.11E-16	0.248230704	1	1	6.15E-12
Fth1	1.22E-10	0.24460055	1	0.999	6.75E-06
Rpl26	5.53E-12	0.24397461	0.987	0.979	3.07E-07
Rps14	7.47E-15	0.239811579	0.996	0.996	4.14E-10
Rpl21	2.83E-12	0.220286787	0.997	0.992	1.57E-07
mt-Nd2	4.12E-09	0.210164935	0.994	0.994	0.000228
Rpl12	1.83E-09	0.208291083	0.981	0.967	0.000101
Rps16	1.67E-10	0.199860157	0.998	0.985	9.27E-06
mt-Nd1	3.40E-07	0.19601503	0.999	0.994	0.018831
Rpl13	2.59E-08	0.193189134	0.996	0.991	0.001434
Rpl28	1.68E-07	0.179541138	0.988	0.98	0.0093
Rps5	5.55E-08	0.170095269	0.999	0.997	0.003076
Malat1	3.85E-07	0.164173717	1	1	0.021312
Hbb-bs	1.93E-18	0.118185807	0.756	0.593	1.07E-13

Fibro-2_UP					
Gene	p_val	avg_log2FC	pct.1	pct.2	p_val_adj
Angptl7	4.52E-20	1.503465	0.158	0.088	2.50E-15
Cthrc1	3.14E-10	1.267198	0.11	0.073	1.74E-05
Htra4	1.53E-11	1.081998	0.115	0.07	8.50E-07
Cdo1	4.04E-24	1.039265	0.214	0.12	2.24E-19
mt-Nd3	7.65E-23	1.009615	0.199	0.118	4.24E-18
Pltp	4.53E-80	0.99602	0.621	0.412	2.51E-75
Edil3	3.01E-16	0.976286	0.167	0.098	1.67E-11
Nt5e	3.64E-12	0.952177	0.107	0.057	2.01E-07
Tnfrsf11b	3.94E-11	0.925157	0.125	0.078	2.18E-06
Gm4149	5.87E-10	0.904134	0.103	0.063	3.25E-05
Epb41l4aos	3.09E-15	0.875908	0.167	0.105	1.71E-10
Serpine1	6.43E-08	0.865625	0.119	0.079	0.003564
Ackr2	1.65E-09	0.829502	0.123	0.08	9.15E-05
Mt1	9.52E-49	0.809381	0.913	0.868	5.27E-44
Nr1d1	1.61E-13	0.80652	0.18	0.114	8.94E-09
Mt2	8.92E-35	0.791994	0.75	0.663	4.94E-30
Foxd1	9.89E-17	0.780719	0.242	0.165	5.48E-12
Bnip3	1.96E-17	0.767674	0.237	0.157	1.09E-12
Snhg6	2.44E-10	0.755172	0.15	0.1	1.35E-05
Rgcc	4.79E-15	0.751779	0.33	0.249	2.66E-10
Gm2000	1.31E-13	0.749727	0.171	0.119	7.26E-09
Nr1d2	1.48E-11	0.739543	0.173	0.114	8.19E-07
Ltbp2	6.17E-11	0.735697	0.2	0.154	3.42E-06
mt-Nd6	3.00E-09	0.723566	0.127	0.095	0.000166
Sncg	7.05E-07	0.718857	0.105	0.067	0.03907
Cpe	8.31E-13	0.714408	0.227	0.167	4.60E-08
Gm3511	2.30E-07	0.702636	0.117	0.079	0.012738
Plaur	6.95E-09	0.682178	0.124	0.097	0.000385
Uaca	1.55E-11	0.680972	0.187	0.132	8.56E-07
Chst15	1.08E-09	0.655157	0.179	0.132	5.98E-05
Rpl38	3.41E-55	0.647263	0.672	0.545	1.89E-50
Inhbb	5.85E-07	0.644474	0.13	0.096	0.032425
Prg4	6.22E-14	0.625104	0.78	0.755	3.45E-09
Nr4a3	8.29E-07	0.613468	0.145	0.108	0.045945
Sox5	2.86E-09	0.609265	0.194	0.148	0.000158
Gm9794	4.67E-10	0.605283	0.17	0.132	2.59E-05
Nr4a1	9.71E-18	0.605155	0.422	0.333	5.38E-13
Aebp1	7.01E-48	0.604758	0.873	0.817	3.89E-43
Piezo1	2.24E-09	0.601255	0.176	0.134	0.000124
Igf1r	6.98E-21	0.59527	0.396	0.317	3.87E-16
S100a4	6.38E-40	0.595129	0.665	0.532	3.54E-35
Fbln7	2.94E-15	0.594227	0.405	0.338	1.63E-10
Crip1	3.72E-39	0.590753	0.967	0.955	2.06E-34
Synm	3.00E-22	0.576321	0.414	0.321	1.66E-17
Papss2	4.67E-09	0.574398	0.155	0.126	0.000259

Rps27	1.21E-89	0.57379	0.916	0.857	6.71E-85
Galnt15	2.24E-23	0.572254	0.529	0.439	1.24E-18
Gfpt2	8.16E-22	0.570094	0.51	0.445	4.52E-17
Rps28	2.89E-41	0.567257	0.623	0.535	1.60E-36
Smim26	3.29E-07	0.553386	0.121	0.098	0.018231
Olfml2a	1.73E-16	0.550647	0.379	0.303	9.60E-12
Snhg8	4.52E-18	0.549055	0.397	0.31	2.50E-13
mt-Nd5	7.01E-77	0.548777	0.906	0.816	3.89E-72
Adarb1	3.47E-08	0.547066	0.171	0.136	0.001921
mt-Rnr1	6.71E-134	0.546263	0.999	0.995	3.72E-129
Flnc	1.51E-10	0.544236	0.19	0.167	8.35E-06
Sgms2	5.81E-17	0.538175	0.341	0.272	3.22E-12
Tnfaip2	2.32E-11	0.537617	0.305	0.252	1.28E-06
Hk2	6.84E-10	0.537214	0.228	0.191	3.79E-05
Itga5	1.94E-11	0.533498	0.26	0.215	1.08E-06
Ndufb1	6.99E-18	0.531174	0.362	0.293	3.87E-13
Snrpg	2.06E-16	0.528653	0.341	0.271	1.14E-11
Neat1	4.79E-34	0.524385	0.783	0.701	2.65E-29
Slc6a6	3.80E-17	0.522662	0.493	0.432	2.10E-12
Ccn5	1.25E-12	0.521584	0.403	0.325	6.92E-08
Gm9843	6.15E-07	0.519088	0.174	0.138	0.034065
Cp	3.94E-10	0.506507	0.289	0.229	2.18E-05
Runx1	1.34E-13	0.503216	0.382	0.316	7.44E-09
Ccn2	1.13E-11	0.498952	0.46	0.403	6.25E-07
Fn1	2.10E-19	0.497027	0.924	0.917	1.17E-14
Ntn4	7.00E-09	0.494871	0.23	0.191	0.000388
Rpl36	8.75E-93	0.493956	0.966	0.934	4.85E-88
Rps29	1.06E-62	0.493236	0.88	0.831	5.85E-58
Sema3e	1.47E-10	0.492538	0.337	0.268	8.16E-06
Dkk3	9.61E-13	0.49062	0.359	0.303	5.33E-08
Ssc5d	2.61E-18	0.489424	0.37	0.314	1.45E-13
Rps21	1.33E-117	0.489087	0.991	0.978	7.38E-113
Ackr3	8.52E-22	0.48834	0.6	0.535	4.72E-17
Pik3ip1	4.10E-09	0.488198	0.221	0.181	0.000227
Sgk1	2.19E-13	0.485994	0.431	0.376	1.21E-08
Mitf	1.11E-11	0.485569	0.323	0.256	6.14E-07
Mustn1	3.19E-19	0.484632	0.645	0.567	1.77E-14
2410006H1	5.60E-20	0.484374	0.529	0.455	3.11E-15
Atp5md	2.00E-12	0.484013	0.322	0.26	1.11E-07
Rpl39	9.87E-101	0.480274	0.971	0.949	5.47E-96
Lox	1.62E-11	0.47665	0.381	0.316	8.98E-07
Lgals3	4.86E-34	0.470937	0.794	0.704	2.69E-29
Mrpl33	7.28E-10	0.465661	0.232	0.196	4.03E-05
Crim1	1.60E-07	0.465026	0.223	0.183	0.008878
Rcan2	6.40E-07	0.46174	0.216	0.178	0.035458
Tgfb1	1.70E-07	0.459541	0.194	0.156	0.009395
Glul	4.68E-17	0.458688	0.531	0.456	2.59E-12

Bola2	7.97E-07	0.457506	0.194	0.155	0.044176
Ppp1r12b	1.78E-07	0.453183	0.182	0.157	0.009848
Ctsk	1.48E-21	0.451423	0.668	0.606	8.20E-17
Adgrg2	1.54E-08	0.450862	0.263	0.209	0.000852
Ptprd	1.28E-07	0.448498	0.258	0.225	0.007073
mt-Nd2	6.36E-91	0.443712	0.997	0.99	3.52E-86
Rpl37a	1.37E-102	0.441347	0.991	0.983	7.57E-98
Gm37376	9.69E-08	0.440802	0.229	0.201	0.005371
Foxc1	1.10E-08	0.439074	0.277	0.23	0.000607
Septin11	8.51E-12	0.435277	0.37	0.31	4.72E-07
Abca1	4.26E-12	0.433547	0.378	0.315	2.36E-07
Rpl37	1.05E-83	0.429159	0.985	0.964	5.83E-79
1110038B1	5.55E-08	0.416783	0.237	0.211	0.003078
Aqp1	2.11E-21	0.416213	0.438	0.317	1.17E-16
Errfi1	3.30E-10	0.414133	0.432	0.377	1.83E-05
Ccdc3	3.41E-07	0.413728	0.282	0.251	0.018912
Pamr1	1.52E-11	0.410273	0.392	0.33	8.42E-07
Ugdh	2.01E-12	0.409715	0.592	0.553	1.11E-07
mt-Co1	3.92E-43	0.403283	0.908	0.852	2.17E-38
Klf4	2.50E-22	0.402532	0.854	0.841	1.38E-17
Ildr2	1.17E-07	0.400754	0.329	0.283	0.006484
Tomm7	4.33E-21	0.397127	0.562	0.495	2.40E-16
Naglu	4.79E-07	0.396437	0.167	0.15	0.026558
Pim1	7.74E-07	0.394817	0.207	0.157	0.042882
Samd4	1.93E-12	0.393036	0.303	0.275	1.07E-07
Tob1	2.86E-13	0.385408	0.443	0.402	1.58E-08
mt-Nd4	2.83E-68	0.382746	0.994	0.983	1.57E-63
mt-Nd1	1.08E-64	0.380315	0.995	0.988	5.99E-60
Plod2	2.03E-09	0.378723	0.291	0.273	0.000113
Creb5	3.38E-15	0.376216	0.591	0.527	1.87E-10
Ndufa1	6.25E-16	0.374722	0.451	0.402	3.46E-11
Cd44	3.04E-10	0.374293	0.494	0.444	1.69E-05
Snrpf	5.29E-10	0.372878	0.376	0.324	2.93E-05
Tsc22d2	4.75E-08	0.372386	0.254	0.231	0.002634
Prss23	1.62E-10	0.367078	0.478	0.405	8.99E-06
Anxa8	1.08E-08	0.364799	0.463	0.421	0.000599
Sulf2	1.22E-08	0.363724	0.405	0.344	0.000678
Atp5mpl	1.27E-14	0.360473	0.461	0.415	7.01E-10
Tmem258	1.03E-10	0.355487	0.393	0.354	5.73E-06
Clic4	6.45E-18	0.354831	0.657	0.614	3.57E-13
Ezr	1.19E-09	0.34989	0.351	0.318	6.62E-05
Klf2	1.31E-10	0.349106	0.607	0.592	7.25E-06
Zmiz1	2.50E-14	0.348924	0.488	0.448	1.39E-09
Syne1	2.68E-10	0.348041	0.416	0.377	1.48E-05
Rpl36a	4.59E-40	0.344102	0.927	0.905	2.54E-35
Rpl41	4.76E-79	0.343209	0.996	0.987	2.64E-74
mt-Rnr2	5.55E-74	0.340931	1	1	3.08E-69

Nsg1	5.31E-14	0.340189	0.622	0.544	2.94E-09
Rpl31	5.03E-20	0.3389	0.648	0.599	2.79E-15
Notch2	1.17E-07	0.33785	0.3	0.271	0.006458
Colec12	4.14E-17	0.337693	0.675	0.64	2.29E-12
Cox7c	2.07E-11	0.337608	0.396	0.357	1.15E-06
Ndrg1	1.27E-13	0.330389	0.601	0.54	7.04E-09
Gabarapl1	9.15E-09	0.328763	0.392	0.355	0.000507
Rcor3	4.51E-07	0.328089	0.195	0.182	0.024967
Comp	3.81E-07	0.327916	0.686	0.644	0.02111
Agap1	3.30E-07	0.326232	0.302	0.273	0.018274
Rplp2	5.48E-61	0.323274	0.99	0.988	3.04E-56
Btg2	1.47E-08	0.32291	0.52	0.505	0.000816
Uap1	5.68E-11	0.32218	0.615	0.598	3.15E-06
Abi1	3.94E-07	0.315957	0.327	0.29	0.021813
Nox4	3.37E-10	0.311893	0.324	0.305	1.87E-05
Ppp3ca	4.49E-12	0.310188	0.561	0.515	2.49E-07
Serpine2	5.31E-15	0.307112	0.883	0.851	2.94E-10
Ctdsp2	2.65E-12	0.303578	0.53	0.491	1.47E-07
Tmem65	5.42E-10	0.303412	0.427	0.394	3.00E-05
Rbpj	1.70E-10	0.302737	0.446	0.414	9.40E-06
Rpl35	6.05E-29	0.302386	0.874	0.837	3.35E-24
Brd2	5.90E-11	0.301862	0.471	0.442	3.27E-06
Bhlhe40	3.62E-07	0.301478	0.402	0.359	0.02006
Rabgap1l	3.14E-07	0.29775	0.414	0.37	0.017382
Romo1	1.89E-13	0.297384	0.505	0.469	1.05E-08
Rps25	1.32E-25	0.294717	0.91	0.886	7.33E-21
Serinc3	1.91E-10	0.291694	0.52	0.476	1.06E-05
Smurf2	9.85E-08	0.290372	0.45	0.409	0.005459
Cox6c	5.34E-19	0.288289	0.759	0.719	2.96E-14
Mmp2	4.15E-11	0.287389	0.737	0.723	2.30E-06
Rpl34	1.78E-39	0.287016	0.981	0.974	9.84E-35
Utrn	1.00E-09	0.28571	0.491	0.458	5.54E-05
Pdgfrb	9.72E-08	0.281284	0.397	0.37	0.005387
Egr1	3.04E-07	0.280981	0.656	0.622	0.016841
Tm4sf1	4.95E-07	0.278332	0.497	0.47	0.027424
Aplp2	4.47E-16	0.277311	0.717	0.694	2.48E-11
Zfp36l1	1.32E-10	0.277278	0.829	0.818	7.30E-06
Mbnl1	7.39E-13	0.276684	0.786	0.767	4.10E-08
Rpl27	9.20E-17	0.27491	0.683	0.644	5.10E-12
Auts2	4.29E-07	0.268505	0.373	0.343	0.023762
Sdc4	3.04E-11	0.268279	0.696	0.658	1.69E-06
Dpm3	6.54E-07	0.267783	0.452	0.411	0.036216
Scara3	1.34E-08	0.265618	0.698	0.667	0.000745
Abi3bp	1.59E-17	0.2652	0.909	0.888	8.83E-13
mt-Cytb	1.77E-53	0.265117	1	0.999	9.83E-49
Prkca	2.15E-07	0.264713	0.303	0.289	0.01189
B2m	1.61E-18	0.261624	0.893	0.868	8.94E-14

Atp2b4	2.85E-07	0.261362	0.418	0.4	0.015815
Zcchc24	1.17E-09	0.260973	0.508	0.478	6.48E-05
Pcbp2	1.15E-13	0.259141	0.686	0.645	6.38E-09
Gas5	1.63E-13	0.258336	0.833	0.801	9.01E-09
Cavin1	1.26E-15	0.256303	0.825	0.801	7.01E-11
Pgrmc1	1.67E-08	0.24978	0.431	0.407	0.000926
Lrrfip1	1.81E-08	0.248469	0.222	0.223	0.001003
Atp5k	6.04E-07	0.247047	0.309	0.296	0.033472
Uqcr11	2.59E-09	0.246008	0.475	0.449	0.000143
Srsf5	2.11E-07	0.245538	0.437	0.414	0.011683
Polr2i	2.49E-07	0.24548	0.271	0.261	0.013795
Rpl35a	1.96E-27	0.245186	0.976	0.971	1.09E-22
Dusp1	4.92E-09	0.244036	0.602	0.555	0.000273
Rps17	1.42E-20	0.24225	0.934	0.916	7.85E-16
Mrpl52	8.72E-11	0.240107	0.499	0.475	4.83E-06
Dpysl2	3.65E-09	0.239536	0.637	0.604	0.000202
Sh3pxd2a	3.76E-07	0.23795	0.256	0.249	0.020826
Ltbp1	2.74E-09	0.234941	0.462	0.459	0.000152
Cd109	1.19E-07	0.234141	0.534	0.505	0.006612
Ndufa6	1.46E-08	0.233698	0.608	0.573	0.000809
Qk	2.57E-07	0.22854	0.531	0.5	0.014261
Rpl30	5.90E-22	0.2282	0.971	0.967	3.27E-17
Pkd1	7.54E-08	0.224589	0.372	0.358	0.004178
Selenos	1.92E-09	0.221448	0.574	0.55	0.000106
Rock2	4.94E-09	0.219458	0.734	0.708	0.000274
Atox1	1.89E-10	0.218605	0.652	0.651	1.05E-05
Dnm1	5.77E-07	0.2149	0.4	0.387	0.031993
Actn4	7.03E-07	0.214392	0.419	0.404	0.038939
Son	6.38E-10	0.213107	0.649	0.622	3.54E-05
Pmepa1	1.18E-07	0.212447	0.721	0.72	0.006558
Ndufa2	3.57E-12	0.212288	0.682	0.654	1.98E-07
Uqcr10	2.47E-09	0.211811	0.575	0.551	0.000137
Rps26	6.21E-24	0.211256	0.988	0.985	3.44E-19
Ntn1	1.78E-07	0.195263	0.608	0.586	0.009859
Rbms1	2.90E-09	0.191907	0.725	0.706	0.000161
Palld	1.72E-08	0.19065	0.36	0.371	0.000956
S100a6	6.71E-24	0.189776	0.999	0.998	3.72E-19
Atp5e	3.54E-09	0.189036	0.819	0.79	0.000196
Lum	1.20E-09	0.187121	0.764	0.806	6.65E-05
Iqgap1	7.29E-07	0.183649	0.644	0.635	0.040378
Glud1	6.19E-09	0.182851	0.773	0.752	0.000343
Sem1	1.85E-09	0.182804	0.813	0.778	0.000103
Fgfr1	2.04E-07	0.182755	0.611	0.595	0.011288
Eea1	7.23E-07	0.180559	0.444	0.442	0.040075
Plec	4.11E-07	0.180519	0.655	0.64	0.022784
Rpl23a	2.84E-10	0.170927	0.932	0.927	1.57E-05
Mir6236	6.30E-08	0.166717	0.905	0.879	0.003493

Pdpn	5.71E-07	0.165244	0.527	0.532	0.031638
Bicd2	7.28E-07	0.163524	0.288	0.293	0.040355
Cd9	1.99E-12	0.163346	0.98	0.96	1.10E-07
Rpl22	1.86E-11	0.162751	0.975	0.969	1.03E-06
Rps23	1.38E-12	0.157606	0.989	0.991	7.67E-08
Itgb1	3.05E-09	0.156193	0.787	0.786	0.000169
Slk	7.50E-07	0.155788	0.471	0.47	0.041572
Tmbim1	1.32E-08	0.14964	0.363	0.373	0.000733
Malat1	5.99E-10	0.141552	1	1	3.32E-05
Rps15a	7.25E-08	0.138984	0.989	0.987	0.004018
Sypl	7.53E-07	0.132192	0.336	0.348	0.041748
Rps15	7.54E-09	0.13187	0.987	0.987	0.000418
Fau	7.10E-08	0.129994	0.995	0.993	0.003934
Hba-a1	2.22E-12	0.118142	0.345	0.26	1.23E-07
Rpl32	4.80E-10	0.1115	0.994	0.999	2.66E-05
Ablim1	8.72E-07	0.101036	0.639	0.664	0.0483

Pre-Obs_DOWN					
Gene	p_val	avg_log2FC	pct.1	pct.2	p_val_adj
Angptl4	5.93E-10	-0.825522393	0.174	0.38	3.29E-05
Hspa1a	1.10E-07	-0.820079169	0.773	0.751	0.006115
Tmed3	3.52E-07	-0.751257972	0.398	0.57	0.019511
Serpinh1	9.83E-16	-0.745954175	0.757	0.919	5.45E-11
Ibsp	1.17E-11	-0.698607123	0.941	0.975	6.50E-07
Ssr2	3.19E-07	-0.662602524	0.411	0.609	0.017678
Col5a2	2.42E-08	-0.607115937	0.586	0.767	0.001343
Sparc	6.10E-13	-0.553862884	0.97	1	3.38E-08
Rcn3	4.49E-10	-0.545147091	0.799	0.902	2.49E-05
Pdia3	4.94E-08	-0.543674255	0.707	0.84	0.002736
Lum	1.94E-10	-0.526913299	0.681	0.859	1.08E-05
Col1a1	6.61E-08	-0.492096105	0.901	0.952	0.003664
Col1a2	5.02E-08	-0.402457663	0.964	0.985	0.002784
Cd63	8.92E-08	-0.375704688	0.964	0.988	0.004944
Tnc	9.16E-08	-0.344472587	0.694	0.861	0.005076

OsteoX_DOWN					
**Gene**	**p_val**	**avg_log2FC**	**pct.1**	**pct.2**	**p_val_adj**
Ostn	2.23E-51	-2.963102202	0.242	0.606	1.23E-46
Col13a1	1.74E-13	-1.89713291	0.06	0.208	9.63E-09
Mest	1.28E-08	-1.528707842	0.079	0.194	0.000709
7SK.293	2.25E-18	-1.52426386	0.143	0.363	1.24E-13
Bglap2	2.9E-74	-1.49002491	0.709	0.95	1.61E-69
C1qtnf6	2.81E-13	-1.477677333	0.109	0.278	1.55E-08
Fibin	3.49E-07	-1.473393576	0.058	0.152	0.019316
Adam12	1.35E-21	-1.455535796	0.097	0.317	7.5E-17
Bglap	8.57E-58	-1.365756762	0.554	0.896	4.75E-53
Fam167a	7.6E-08	-1.346384251	0.055	0.155	0.004209
Prr7	1.15E-07	-1.338239387	0.055	0.152	0.006362
Rab27b	4.1E-23	-1.277700919	0.206	0.477	2.27E-18
Chn2	6.36E-13	-1.260603556	0.081	0.229	3.53E-08
Slc36a2	7.91E-09	-1.23956859	0.076	0.194	0.000438
Acan	1.81E-11	-1.200495204	0.127	0.288	1E-06
Ten1	2.94E-08	-1.180054582	0.051	0.143	0.001628
Cilp2	6.07E-07	-1.170614565	0.157	0.278	0.033618
Pitx1	1.02E-10	-1.163316279	0.081	0.213	5.64E-06
Cthrc1	3.38E-14	-1.15386072	0.24	0.446	1.87E-09
Bmp5	1.63E-09	-1.145874035	0.088	0.214	9.02E-05
Sox4	5.41E-23	-1.143531989	0.316	0.591	3E-18
Psmb5	5.22E-17	-1.10483193	0.247	0.475	2.89E-12
Mgll	1.74E-17	-1.103602974	0.252	0.486	9.62E-13
Tspan18	1.27E-08	-1.092838698	0.12	0.254	0.000706
Col3a1	1.81E-60	-1.058206134	0.933	0.985	1E-55
Cuta	1.01E-10	-1.053381726	0.139	0.298	5.59E-06
Mmp14	6.43E-07	-1.044059453	0.125	0.241	0.035645
Intu	5.79E-07	-1.036750835	0.102	0.213	0.032091
Hmox2	1.11E-09	-1.035310892	0.189	0.348	6.15E-05
Pdzrn4	4.06E-10	-1.01824354	0.182	0.346	2.25E-05
Trnp1	5E-08	-0.965446732	0.065	0.159	0.002769
Ufm1	1.11E-07	-0.905912465	0.125	0.25	0.006142
Fat3	8.34E-11	-0.904493933	0.27	0.452	4.62E-06
Smco4	5.8E-09	-0.898027248	0.148	0.291	0.000321
Bccip	3.1E-07	-0.891551302	0.159	0.289	0.017175
Col1a1	1.97E-28	-0.879889076	0.977	0.993	1.09E-23
Podnl1	6.57E-08	-0.874665893	0.18	0.322	0.003643
Mdk	1.36E-13	-0.873076725	0.425	0.618	7.54E-09
Col11a1	3.72E-11	-0.867272632	0.37	0.551	2.06E-06
Sec22b	4.29E-07	-0.861702637	0.185	0.317	0.023752
Serpinh1	1.39E-48	-0.861538164	0.866	0.973	7.69E-44
Sparc	1.51E-47	-0.842497765	0.995	1	8.39E-43
Mfap2	7.48E-11	-0.842214022	0.266	0.449	4.15E-06
Mras	8.82E-07	-0.82513411	0.099	0.201	0.048893
P4ha3	3.78E-07	-0.821598144	0.132	0.25	0.02096

S100a1	7.82E-10	-0.784388764	0.231	0.401	4.33E-05
Kctd12	2.8E-14	-0.773278136	0.573	0.75	1.55E-09
Taldo1	1.52E-08	-0.770988811	0.222	0.377	0.000842
Ppp1r14b	3.31E-09	-0.769009138	0.339	0.491	0.000183
Sytl2	4.34E-07	-0.761073703	0.141	0.258	0.024042
Cbfb	2.37E-09	-0.752011545	0.312	0.481	0.000131
Snrpd3	1.56E-07	-0.739435548	0.224	0.369	0.008627
Col16a1	9.63E-18	-0.720344455	0.721	0.836	5.34E-13
Epha3	6.74E-07	-0.711265303	0.155	0.274	0.037352
Igf1	1.87E-08	-0.708386541	0.284	0.444	0.001038
Tmed3	1.9E-15	-0.692854755	0.536	0.714	1.05E-10
Cope	6.02E-11	-0.691220572	0.404	0.584	3.34E-06
Ccdc50	3.03E-08	-0.678235547	0.39	0.546	0.001681
Col5a2	1.54E-27	-0.676945447	0.82	0.936	8.51E-23
Postn	3.05E-34	-0.670008817	0.827	0.975	1.69E-29
Col5a1	5.27E-14	-0.668013719	0.626	0.769	2.92E-09
Wnt5b	1.59E-07	-0.666658875	0.349	0.502	0.008834
Dpysl3	5.39E-21	-0.666029142	0.624	0.821	2.99E-16
Col1a2	4.43E-28	-0.660471579	0.993	0.999	2.45E-23
Mrps28	3.14E-08	-0.653835196	0.21	0.357	0.001742
Cd63	2.04E-46	-0.641820427	0.988	1	1.13E-41
Kdelr3	8.35E-09	-0.623486127	0.273	0.437	0.000463
Pdia3	3.76E-19	-0.621574282	0.764	0.874	2.08E-14
Dad1	1.79E-18	-0.620393874	0.617	0.81	9.92E-14
Myo1b	9.59E-08	-0.610183676	0.284	0.438	0.005316
Kdelr2	1.55E-14	-0.609326818	0.58	0.761	8.59E-10
Lamtor4	1.55E-08	-0.605372987	0.247	0.402	0.000856
Sdc2	1.88E-20	-0.602749008	0.882	0.953	1.04E-15
Pja1	1.69E-07	-0.599249049	0.127	0.232	0.009344
Ostc	4.89E-11	-0.594574151	0.425	0.616	2.71E-06
S100a16	2.17E-09	-0.582557604	0.457	0.63	0.00012
Olfml2b	5.39E-11	-0.581826246	0.497	0.679	2.99E-06
Clec11a	6.68E-20	-0.57974305	0.857	0.94	3.7E-15
Psmd6	5.7E-07	-0.573590984	0.15	0.26	0.031574
Mylk	6.62E-15	-0.572720759	0.51	0.727	3.67E-10
Arf5	3.83E-13	-0.569347718	0.633	0.798	2.12E-08
Adamts2	2.91E-08	-0.567232243	0.513	0.654	0.001613
Ufc1	3.84E-08	-0.563469789	0.314	0.474	0.00213
Tle5	3.7E-08	-0.555722099	0.443	0.602	0.002048
Pcolce	5.39E-23	-0.551377839	0.834	0.945	2.99E-18
Gpc1	7.08E-09	-0.549496598	0.42	0.591	0.000392
Psmb3	1.35E-09	-0.54776317	0.441	0.618	7.51E-05
Cdk4	3.22E-07	-0.540560767	0.27	0.413	0.01785
Serpinf1	1.61E-19	-0.537922751	0.97	0.985	8.92E-15
Atp5g1	2.74E-07	-0.532010481	0.425	0.575	0.015186
Ndufb9	3.93E-09	-0.520586302	0.566	0.712	0.000218
Sec61b	5.69E-08	-0.517724211	0.626	0.748	0.003155

Cnpy2	2.77E-08	-0.50902179	0.473	0.633	0.001536
Tnc	1.23E-07	-0.497139161	0.707	0.816	0.006835
Rcn3	1.39E-17	-0.486873159	0.855	0.919	7.72E-13
Copz2	1.84E-07	-0.484777657	0.499	0.64	0.010197
Mmp23	5.46E-07	-0.481036093	0.353	0.503	0.03024
Ssr2	2.57E-07	-0.477876563	0.527	0.666	0.014258
Tmed10	5.67E-07	-0.477516379	0.51	0.653	0.031409
Fkbp7	5.53E-07	-0.445057349	0.538	0.678	0.03066
Enah	4.19E-08	-0.439375953	0.589	0.735	0.002323
Psmb6	6.51E-07	-0.436800231	0.497	0.641	0.0361
Eif5a	1.53E-07	-0.428468092	0.688	0.786	0.008491
Nenf	4.1E-09	-0.421251384	0.771	0.874	0.000227
Psma5	5.81E-07	-0.418796368	0.196	0.31	0.03222
Emp3	3.72E-08	-0.405321403	0.755	0.847	0.002063
Tmed9	4.35E-07	-0.381305293	0.686	0.785	0.024098
Hexa	7.4E-07	-0.377550563	0.635	0.761	0.040996
Npc2	6.15E-09	-0.370022205	0.637	0.79	0.000341
Alpl	1.62E-07	-0.366638675	0.737	0.839	0.008971
Pth1r	5.27E-07	-0.35200988	0.635	0.769	0.0292
Ctsl	2.23E-10	-0.31998042	0.949	0.974	1.23E-05
Ptn	2.13E-08	-0.313546528	0.566	0.723	0.001178
Ctsb	8.53E-08	-0.311457319	0.901	0.943	0.004729
Hsp90b1	2.58E-07	-0.307178793	0.843	0.914	0.014295
Bsg	6.08E-08	-0.302774323	0.79	0.889	0.003368
Gnas	6.8E-11	-0.280862095	0.965	0.988	3.77E-06

Fibro-1_DOWN					
**Gene**	**p_val**	**avg_log2FC**	**pct.1**	**pct.2**	**p_val_adj**
Ostn	1.92E-24	-3.413448406	0.045	0.168	1.07E-19
Nppc	6.29E-34	-2.754609482	0.049	0.226	3.48E-29
Mest	4.51E-20	-2.105797466	0.055	0.183	2.50E-15
7SK.293	1.50E-38	-1.980823204	0.188	0.426	8.32E-34
Wnt16	2.80E-30	-1.974959386	0.099	0.297	1.55E-25
Trp53inp2	9.90E-11	-1.782736015	0.043	0.121	5.49E-06
Lrrn1	8.27E-14	-1.696992502	0.073	0.178	4.58E-09
Cilp2	3.56E-43	-1.623246145	0.26	0.509	1.97E-38
Col8a1	1.67E-34	-1.606566792	0.247	0.465	9.23E-30
Mfap4	2.98E-23	-1.593498582	0.161	0.344	1.65E-18
Tspan18	9.09E-09	-1.552512135	0.037	0.103	0.000504
Fbxo2	3.35E-24	-1.536846255	0.189	0.366	1.86E-19
Postn	1.46E-10	-1.534375192	0.114	0.211	8.09E-06
Pstpip1	3.94E-11	-1.466995898	0.05	0.138	2.18E-06
Megf6	2.11E-15	-1.449330556	0.093	0.219	1.17E-10
Egfl6	6.35E-09	-1.44249972	0.047	0.121	0.000352
Igfbp5	7.37E-51	-1.380405872	0.613	0.822	4.08E-46
Igf1	7.21E-40	-1.323937453	0.387	0.638	4.00E-35
Gamt	6.74E-11	-1.279648894	0.081	0.179	3.74E-06
Dlx6	9.22E-08	-1.244545225	0.062	0.135	0.00511
Ptn	6.49E-20	-1.195504846	0.266	0.447	3.60E-15
Spon1	1.95E-18	-1.15407581	0.222	0.389	1.08E-13
Crabp2	8.06E-14	-1.136134464	0.126	0.259	4.47E-09
Acp5	9.25E-11	-1.105389296	0.1	0.206	5.12E-06
Peg3	1.32E-17	-1.097024369	0.213	0.381	7.30E-13
Mrps34	6.17E-10	-0.969893854	0.132	0.24	3.42E-05
Itm2a	1.39E-31	-0.967920703	0.499	0.701	7.69E-27
Col15a1	2.74E-09	-0.966788825	0.116	0.218	0.000152
Crispld2	2.54E-32	-0.946900787	0.68	0.776	1.41E-27
Mgll	7.51E-08	-0.943574078	0.136	0.224	0.00416
Mfap2	5.74E-20	-0.908852199	0.273	0.465	3.18E-15
Fkbp11	7.63E-09	-0.904645245	0.113	0.211	0.000423
Letm1	6.98E-08	-0.904254643	0.087	0.17	0.003867
Abhd8	5.20E-07	-0.903381549	0.113	0.197	0.028822
Dio2	6.69E-14	-0.897576988	0.278	0.435	3.71E-09
Epha4	3.83E-07	-0.889088282	0.11	0.194	0.021216
Mdk	2.06E-15	-0.882182726	0.346	0.483	1.14E-10
Cntfr	1.71E-09	-0.86159747	0.119	0.224	9.48E-05
Bglap2	1.89E-13	-0.847751926	0.262	0.416	1.04E-08
Atf5	3.72E-08	-0.830092809	0.247	0.35	0.00206
Arl2	1.11E-07	-0.829549972	0.15	0.243	0.006125
Hspa1a	7.70E-11	-0.81168317	0.741	0.761	4.27E-06
Smco4	3.93E-12	-0.806028446	0.144	0.27	2.18E-07
Epha3	4.47E-15	-0.804306559	0.18	0.334	2.48E-10
Gnai1	8.21E-07	-0.801738497	0.121	0.207	0.045506

Ostc	4.26E-17	-0.791949939	0.349	0.522	2.36E-12
Tmem70	3.83E-07	-0.782318255	0.11	0.194	0.021235
Serpinh1	2.08E-49	-0.777331738	0.801	0.927	1.16E-44
Ccdc124	2.94E-11	-0.765777483	0.237	0.373	1.63E-06
1110008P1	3.08E-08	-0.764591764	0.137	0.238	0.001708
Pdzd11	5.13E-07	-0.754415201	0.112	0.196	0.028455
Csrp2	3.06E-12	-0.74357813	0.309	0.453	1.69E-07
Pfn2	5.78E-08	-0.741450559	0.172	0.275	0.003205
Fam171b	5.05E-07	-0.738865162	0.114	0.198	0.028009
Mylk	3.46E-07	-0.729980442	0.213	0.31	0.019176
Ifi30	6.09E-07	-0.727114786	0.179	0.274	0.033771
Pitx1	1.03E-11	-0.72587541	0.222	0.364	5.73E-07
Clec11a	8.14E-28	-0.719054566	0.62	0.776	4.51E-23
Oaf	5.75E-09	-0.718381197	0.186	0.301	0.000319
Fat4	3.23E-10	-0.711707902	0.269	0.398	1.79E-05
Taf10	1.26E-10	-0.709306312	0.259	0.392	6.97E-06
Kctd12	8.84E-11	-0.706768186	0.348	0.486	4.90E-06
Chad	3.50E-14	-0.705035365	0.58	0.691	1.94E-09
Wfdc1	2.29E-09	-0.70315358	0.244	0.364	0.000127
Crlf1	1.89E-07	-0.701535411	0.111	0.196	0.010482
Mettl1	9.81E-08	-0.698466826	0.172	0.275	0.005439
Iscu	4.11E-07	-0.696323409	0.216	0.311	0.022782
Ebp	3.21E-08	-0.685557497	0.243	0.352	0.001778
Pdgfrl	2.36E-13	-0.683755763	0.41	0.557	1.31E-08
Taldo1	2.18E-09	-0.678626832	0.278	0.399	0.000121
Col8a2	7.60E-13	-0.677694239	0.438	0.57	4.21E-08
Smdt1	2.80E-20	-0.671208561	0.487	0.671	1.55E-15
Srpx	1.04E-08	-0.670961195	0.282	0.39	0.000577
Slc25a5	1.84E-10	-0.668135203	0.263	0.398	1.02E-05
Tkt	5.77E-07	-0.667431417	0.224	0.323	0.031981
Angptl4	1.56E-08	-0.662506124	0.182	0.293	0.000862
Mrps15	7.75E-07	-0.661843471	0.163	0.258	0.042945
Hoxa10	4.25E-07	-0.658799332	0.322	0.419	0.023547
Mlec	1.20E-10	-0.656260235	0.278	0.416	6.67E-06
Tenm3	1.59E-10	-0.649946645	0.17	0.29	8.79E-06
Sparc	3.42E-42	-0.645023059	0.987	0.998	1.89E-37
Il11ra1	6.78E-20	-0.641197685	0.668	0.788	3.76E-15
Acaa1a	5.06E-08	-0.6367805	0.232	0.343	0.002805
Daam2	5.45E-07	-0.636102979	0.248	0.344	0.030198
Tmed1	4.38E-10	-0.632907417	0.224	0.354	2.43E-05
H2bc4	1.94E-07	-0.618281457	0.273	0.371	0.010732
Emb	8.11E-07	-0.615342729	0.292	0.395	0.04492
Zcchc17	1.74E-07	-0.610148427	0.255	0.364	0.009641
Matn4	4.54E-07	-0.610072939	0.193	0.291	0.025154
Ccnd2	2.92E-14	-0.609182971	0.535	0.66	1.62E-09
Plpp1	2.80E-12	-0.607971151	0.46	0.572	1.55E-07
Creb3l1	3.46E-08	-0.607673329	0.284	0.398	0.001916

Psmc5	4.70E-08	-0.606208012	0.277	0.394	0.002603
Ufc1	8.85E-15	-0.605507121	0.404	0.572	4.90E-10
Sec61b	3.52E-20	-0.589854442	0.633	0.778	1.95E-15
Mrpl18	2.79E-07	-0.587128417	0.197	0.3	0.01548
Ndufs8	1.04E-08	-0.586203019	0.243	0.364	0.000579
Aldh2	2.72E-16	-0.585685189	0.549	0.697	1.51E-11
Stub1	1.40E-09	-0.58295066	0.345	0.468	7.75E-05
Neo1	1.93E-07	-0.582560341	0.276	0.388	0.010694
Id2	8.93E-15	-0.580798103	0.603	0.749	4.95E-10
Pdia3	1.00E-29	-0.575618568	0.783	0.892	5.56E-25
Atp6ap1	1.68E-09	-0.575380538	0.342	0.474	9.33E-05
Spock2	4.87E-11	-0.571608126	0.243	0.379	2.70E-06
Ly6e	1.08E-07	-0.570865851	0.31	0.428	0.005958
Fzd2	5.55E-07	-0.564575402	0.217	0.32	0.030767
Scn1b	5.27E-07	-0.563746073	0.288	0.394	0.029218
Col11a1	1.53E-10	-0.562143705	0.28	0.42	8.47E-06
Tmed3	5.41E-17	-0.558363772	0.579	0.721	3.00E-12
Srpr	2.80E-09	-0.555503837	0.366	0.495	0.000155
Lrpap1	1.06E-08	-0.554789414	0.397	0.515	0.000589
Olfml2b	4.41E-08	-0.550971431	0.299	0.417	0.002445
Calr	8.87E-26	-0.550265723	0.729	0.863	4.91E-21
Copb2	2.70E-09	-0.550075161	0.388	0.511	0.000149
Prrx2	2.29E-10	-0.545810877	0.51	0.616	1.27E-05
Ckb	7.16E-07	-0.543094443	0.379	0.482	0.039663
Slc50a1	1.82E-09	-0.542627635	0.354	0.489	0.000101
Tceal8	3.37E-08	-0.541787784	0.349	0.469	0.001869
Serpinf1	3.07E-23	-0.536185617	0.926	0.965	1.70E-18
Tuba1b	2.53E-13	-0.53595078	0.518	0.659	1.40E-08
Gpc1	8.87E-10	-0.534956421	0.426	0.56	4.91E-05
Lsm4	4.07E-07	-0.534823279	0.281	0.393	0.022559
Nt5c	4.74E-07	-0.534464057	0.194	0.295	0.026258
Gnas	6.64E-28	-0.530829011	0.973	0.988	3.68E-23
Nucb2	6.81E-07	-0.529319662	0.309	0.414	0.037718
Cpxm1	1.89E-09	-0.528721104	0.445	0.572	0.000105
Copz2	6.01E-11	-0.52602076	0.488	0.611	3.33E-06
Ndufb6	1.96E-07	-0.523472942	0.325	0.437	0.010865
Cdkn1c	4.21E-12	-0.519174937	0.753	0.827	2.33E-07
Psmb3	8.96E-12	-0.51344399	0.484	0.624	4.97E-07
Atp5g1	2.19E-10	-0.513233558	0.453	0.588	1.21E-05
Lamtor4	1.23E-07	-0.511681191	0.373	0.485	0.006822
Cnpy2	7.32E-12	-0.511272946	0.526	0.651	4.06E-07
Col1a2	2.41E-28	-0.507435089	0.971	0.987	1.34E-23
Kcnq1ot1	2.81E-09	-0.504433944	0.405	0.537	0.000156
Map1lc3a	1.03E-11	-0.50085294	0.65	0.745	5.70E-07
Sra1	1.46E-07	-0.4981379	0.362	0.472	0.008111
S100a16	2.62E-07	-0.497265031	0.379	0.491	0.014527
Mrfap1	4.36E-08	-0.486639168	0.361	0.483	0.002414

Pcolce	1.49E-26	-0.484530727	0.871	0.927	8.27E-22
Ifi27	7.54E-09	-0.481835423	0.449	0.575	0.000418
Rex1bd	2.19E-07	-0.479026105	0.427	0.537	0.012127
Edf1	2.53E-10	-0.47679387	0.526	0.648	1.40E-05
Col1a1	3.09E-25	-0.475167905	0.909	0.97	1.71E-20
Rcn3	9.15E-20	-0.474650279	0.788	0.887	5.07E-15
Slc25a4	2.15E-20	-0.470764304	0.787	0.887	1.19E-15
Ckap4	7.73E-08	-0.469073996	0.367	0.488	0.004282
Mdh1	2.06E-08	-0.465739671	0.383	0.51	0.001139
Igfbp4	5.91E-12	-0.46394121	0.576	0.709	3.28E-07
Ift20	2.22E-08	-0.462845671	0.464	0.58	0.001233
Srsf3	6.79E-11	-0.462039902	0.534	0.665	3.76E-06
Fkbp1a	1.66E-08	-0.461445083	0.474	0.593	0.000917
Tle5	1.75E-10	-0.459192331	0.541	0.668	9.72E-06
Psmb6	2.81E-11	-0.458845181	0.539	0.679	1.55E-06
Pdia6	1.15E-12	-0.458157712	0.532	0.674	6.39E-08
Tsc22d1	1.55E-10	-0.456897372	0.58	0.701	8.57E-06
Ndufc2	1.32E-07	-0.456376415	0.387	0.507	0.007334
Cope	2.26E-09	-0.455217176	0.517	0.633	0.000125
Cox5a	7.68E-08	-0.453839373	0.4	0.521	0.004257
Hspe1	5.17E-08	-0.453545535	0.536	0.626	0.002866
Tmem119	5.85E-08	-0.452523088	0.339	0.463	0.003242
Ctsl	7.72E-21	-0.445662905	0.942	0.969	4.28E-16
Calm3	9.76E-08	-0.44549865	0.436	0.555	0.005411
Pomp	1.13E-08	-0.44271564	0.431	0.56	0.000626
Selenom	1.10E-19	-0.441264924	0.843	0.912	6.08E-15
Hsbp1	2.09E-08	-0.440014222	0.42	0.542	0.00116
Atp5o.1	6.04E-07	-0.436940424	0.458	0.565	0.033477
Ctsz	8.80E-12	-0.436937821	0.627	0.744	4.88E-07
Hmgn1	7.15E-10	-0.436273308	0.579	0.697	3.96E-05
Olfml3	5.46E-18	-0.434588037	0.923	0.947	3.03E-13
Bcap31	2.63E-08	-0.432516096	0.522	0.633	0.001459
Rhoc	2.75E-09	-0.427658717	0.537	0.654	0.000152
Psmd7	6.22E-07	-0.424347317	0.426	0.54	0.034489
Swi5	1.16E-10	-0.423132519	0.656	0.75	6.41E-06
Fkbp2	1.21E-07	-0.422351527	0.443	0.562	0.006716
Acat1	1.71E-07	-0.414312803	0.315	0.432	0.00945
Dad1	1.42E-13	-0.413034648	0.693	0.796	7.87E-09
Pfn1	5.84E-13	-0.412814951	0.702	0.813	3.24E-08
Prdx2	9.36E-10	-0.40648826	0.613	0.72	5.19E-05
Prrx1	7.20E-15	-0.406435014	0.818	0.859	3.99E-10
Gpx4	1.23E-07	-0.40523063	0.48	0.597	0.006793
Cfl1	4.22E-10	-0.404760077	0.639	0.74	2.34E-05
Tmed9	1.62E-10	-0.401183588	0.68	0.753	9.00E-06
Reep5	8.47E-13	-0.401156516	0.716	0.821	4.69E-08
Ndufb5	1.84E-07	-0.396957742	0.433	0.552	0.01017
Mpc2	4.71E-07	-0.392437927	0.47	0.581	0.026092

Ftl1	3.83E-07	-0.390896765	0.605	0.679	0.021226
Bglap	3.61E-08	-0.385371276	0.243	0.359	0.002
Psma7	2.09E-07	-0.385269853	0.526	0.636	0.011563
Calm1	2.52E-13	-0.383547013	0.809	0.892	1.40E-08
Fkbp7	1.51E-07	-0.382022558	0.557	0.656	0.008354
Cd81	2.81E-18	-0.380012691	0.917	0.95	1.56E-13
Spcs1	2.47E-08	-0.379211977	0.602	0.706	0.001368
Gpx8	6.76E-07	-0.379082935	0.472	0.579	0.037455
Hsp90b1	1.23E-15	-0.375004709	0.869	0.92	6.81E-11
Eif5a	2.80E-10	-0.366247307	0.716	0.806	1.55E-05
Sar1a	3.92E-07	-0.361682137	0.485	0.601	0.021708
Ppib	9.51E-19	-0.360196487	0.88	0.946	5.27E-14
Ndufb9	7.28E-09	-0.356651063	0.638	0.735	0.000403
Akr1a1	2.16E-09	-0.356012576	0.677	0.777	0.00012
Psma3	5.54E-07	-0.352563084	0.492	0.606	0.030689
Atp5d	4.31E-08	-0.352513155	0.656	0.742	0.002388
Snx3	2.69E-07	-0.350451609	0.419	0.539	0.014887
Prdx1	1.20E-12	-0.350405172	0.756	0.846	6.63E-08
Dynll1	7.90E-13	-0.349604321	0.792	0.885	4.38E-08
Lman1	7.23E-07	-0.348180286	0.456	0.572	0.040063
Tceal9	6.63E-07	-0.348150041	0.565	0.655	0.036747
Marcks	5.35E-09	-0.344230019	0.828	0.863	0.000296
Atp5j	1.86E-09	-0.327761358	0.735	0.817	0.000103
Ogn	9.71E-09	-0.322649204	0.808	0.886	0.000538
Tmed10	5.27E-07	-0.322634683	0.625	0.722	0.029203
Gpx3	5.18E-09	-0.318338705	0.973	0.985	0.000287
Nenf	8.78E-11	-0.317184428	0.814	0.871	4.87E-06
Serpinb6a	1.00E-07	-0.313819102	0.766	0.837	0.005564
Krtcap2	1.40E-07	-0.310395766	0.624	0.731	0.007768
Ssr4	5.81E-08	-0.306670743	0.605	0.722	0.003222
Chchd2	3.95E-07	-0.304478374	0.689	0.778	0.021878
Emp3	1.15E-08	-0.300270191	0.832	0.904	0.000636
Actb	3.54E-11	-0.293838452	0.97	0.987	1.96E-06
Myl6	9.05E-11	-0.29142975	0.857	0.922	5.01E-06
Ndufb10	5.10E-07	-0.281832982	0.599	0.711	0.028237
Cd63	8.50E-11	-0.278512464	0.965	0.977	4.71E-06
Zbtb20	3.01E-07	-0.273033977	0.922	0.929	0.016697
Atp5h	1.24E-08	-0.271903883	0.81	0.866	0.000688
Bsg	1.18E-09	-0.269586206	0.825	0.855	6.55E-05
Laptm4a	1.06E-09	-0.236323019	0.945	0.958	5.89E-05
Col3a1	3.47E-10	-0.124023691	0.702	0.824	1.92E-05

Fibro-2_DOWN					
**Gene**	**p_val**	**avg_log2FC**	**pct.1**	**pct.2**	**p_val_adj**
Dkk2	1.49E-27	-1.247062346	0.126	0.225	8.28E-23
Engase	1.07E-13	-1.177806983	0.05	0.102	5.94E-09
7SK.293	1.68E-41	-1.16382619	0.18	0.326	9.32E-37
Rbp4	3.97E-17	-1.147646141	0.098	0.168	2.2E-12
Sfrp1	1.35E-23	-1.059216221	0.206	0.301	7.46E-19
Lsamp	4.01E-13	-1.052212899	0.054	0.105	2.22E-08
Col15a1	1.41E-19	-1.022087616	0.145	0.23	7.83E-15
Hsph1	9.76E-23	-0.954453758	0.185	0.285	5.41E-18
Itm2a	1.49E-30	-0.774659277	0.244	0.38	8.26E-26
Cxcl14	9.39E-11	-0.770833261	0.278	0.341	5.21E-06
Hspa1a	2.41E-29	-0.765511312	0.682	0.759	1.34E-24
Stmn4	7.26E-09	-0.759096647	0.064	0.107	0.000402
Tmeff2	4.25E-17	-0.751025789	0.116	0.196	2.35E-12
Glt8d2	5.13E-10	-0.734087904	0.074	0.123	2.84E-05
Gamt	2.36E-18	-0.73384181	0.113	0.194	1.31E-13
Ms4a4d	1.14E-18	-0.701431397	0.119	0.202	6.34E-14
Psmb9	3.44E-08	-0.677487756	0.066	0.108	0.001905
Acvr2a	3.33E-11	-0.667582446	0.131	0.195	1.85E-06
Pcdh7	2.02E-13	-0.665298939	0.12	0.191	1.12E-08
Prdm8	1.4E-08	-0.664210021	0.065	0.107	0.000778
Hebp2	4.65E-09	-0.652068412	0.091	0.141	0.000258
Ppdpf	3.02E-15	-0.639686211	0.162	0.246	1.68E-10
Eef1akmt1	2.55E-11	-0.63949066	0.104	0.164	1.41E-06
Lgi2	2.17E-07	-0.632832304	0.071	0.112	0.012029
Bmper	1.29E-10	-0.631950869	0.102	0.16	7.16E-06
Psmb10	1.32E-13	-0.629083986	0.157	0.235	7.3E-09
Lpar4	9.32E-08	-0.625641805	0.086	0.131	0.005162
Srpx	2.02E-12	-0.624870385	0.126	0.194	1.12E-07
Nrep	5.72E-07	-0.623653572	0.091	0.134	0.031717
Fbln1	2.34E-27	-0.623544095	0.339	0.475	1.3E-22
Angptl4	7.35E-18	-0.613755742	0.14	0.227	4.07E-13
Sigmar1	2.89E-17	-0.6122752	0.196	0.29	1.6E-12
Csrp2	3.15E-17	-0.604875295	0.249	0.349	1.74E-12
Cmtm7	8.37E-15	-0.600547914	0.138	0.215	4.64E-10
Pax9	5.13E-08	-0.597318504	0.064	0.104	0.00284
Hspb1	8.83E-13	-0.594454899	0.565	0.634	4.89E-08
Slfn5	1.05E-18	-0.593103263	0.214	0.316	5.84E-14
Clec11a	8.03E-11	-0.587263135	0.184	0.251	4.45E-06
Pi16	2.89E-12	-0.584284642	0.304	0.391	1.6E-07
Ten1	5.7E-10	-0.582577541	0.101	0.155	3.16E-05
Il11ra1	1.86E-34	-0.58019811	0.609	0.736	1.03E-29
Atf5	6.27E-22	-0.579851968	0.426	0.525	3.47E-17
Igf1	4.88E-32	-0.577116795	0.522	0.656	2.71E-27
Fmo1	2.02E-11	-0.574823772	0.139	0.207	1.12E-06
C7	8.78E-12	-0.56476888	0.274	0.352	4.87E-07

Kitl	5.71E-08	-0.564718722	0.09	0.136	0.003162
Ifi27	2.79E-46	-0.562775323	0.573	0.713	1.55E-41
Rab30	7.62E-07	-0.555147768	0.084	0.125	0.042246
Nrp1	2.67E-16	-0.539870243	0.309	0.408	1.48E-11
Tmem126a	5.1E-12	-0.532578549	0.167	0.24	2.83E-07
Lynx1	1.38E-10	-0.52996291	0.117	0.177	7.65E-06
Mrps25	1.6E-08	-0.528896562	0.147	0.204	0.000888
Pla2g4a	6.81E-08	-0.526439937	0.091	0.135	0.003776
Serpinh1	8.37E-60	-0.525809849	0.77	0.878	4.64E-55
Spats2l	1.57E-09	-0.525682592	0.098	0.15	8.71E-05
Pcdh18	1.61E-12	-0.522106198	0.123	0.188	8.94E-08
Rgs10	1.2E-09	-0.518068287	0.154	0.217	6.65E-05
Maged2	5.82E-12	-0.515139157	0.213	0.291	3.22E-07
Podn	6.7E-10	-0.507305148	0.133	0.195	3.71E-05
Pcolce	4.95E-63	-0.504140291	0.862	0.926	2.74E-58
Tmem205	1.16E-13	-0.503005495	0.193	0.276	6.45E-09
Angptl1	4.25E-12	-0.499857393	0.392	0.477	2.36E-07
Tuba1b	2.34E-33	-0.493802706	0.528	0.666	1.3E-28
Josd2	5.54E-08	-0.485753601	0.129	0.181	0.003071
Tmem107	3.98E-13	-0.477937491	0.167	0.244	2.21E-08
Hspd1	3.01E-12	-0.477300264	0.428	0.5	1.67E-07
Jam3	3.33E-09	-0.474999211	0.087	0.132	0.000184
Meox2	1.97E-14	-0.474582654	0.306	0.401	1.09E-09
Gga2	4.27E-07	-0.473532465	0.111	0.157	0.023636
Psme2	1.06E-18	-0.465395708	0.358	0.47	5.86E-14
Acvrl1	2.14E-13	-0.463440349	0.199	0.281	1.19E-08
Etfb	9.3E-10	-0.45675139	0.16	0.223	5.15E-05
Ccdc80	2.05E-22	-0.455776646	0.767	0.819	1.13E-17
Ndn	4.36E-24	-0.45566382	0.339	0.468	2.42E-19
Tmem223	5.32E-11	-0.455411064	0.143	0.206	2.95E-06
Pdia3	5.33E-50	-0.449333878	0.766	0.868	2.95E-45
Banp	2.59E-09	-0.449276096	0.083	0.127	0.000144
Ech1	9.12E-17	-0.44855927	0.365	0.469	5.05E-12
Lefty1	8.73E-07	-0.447692027	0.076	0.113	0.048386
Qpct	1.01E-09	-0.446526622	0.183	0.25	5.57E-05
Fzd4	6.45E-07	-0.441941639	0.159	0.212	0.035749
Gstt1	9.33E-10	-0.43592258	0.221	0.293	5.17E-05
Creld1	4.35E-07	-0.434293514	0.08	0.119	0.024094
Sertad3	3.72E-08	-0.431773731	0.089	0.131	0.002059
Idnk	5.35E-07	-0.431419334	0.116	0.162	0.029636
Gas1	1.28E-20	-0.426051083	0.795	0.844	7.09E-16
Sparc	1.84E-38	-0.425934542	0.975	0.992	1.02E-33
Gpc3	3.79E-16	-0.423424267	0.141	0.217	2.1E-11
Mrpl27	2.36E-07	-0.42263348	0.193	0.252	0.013094
Crlf1	5.57E-07	-0.420207894	0.166	0.22	0.030881
Stub1	1.03E-15	-0.419482803	0.358	0.46	5.71E-11
Thy1	7.39E-08	-0.418575282	0.214	0.277	0.004096

Fus	4.6E-16	-0.417094022	0.34	0.444	2.55E-11
1110065P2	3.1E-10	-0.417006044	0.166	0.232	1.72E-05
Aimp2	3.59E-08	-0.416105068	0.093	0.137	0.001989
Gpx7	3.12E-13	-0.414781754	0.255	0.343	1.73E-08
Nfkbia	5.22E-13	-0.414142732	0.703	0.747	2.89E-08
Stk16	5.71E-09	-0.412780424	0.174	0.237	0.000317
Taf10	1.02E-17	-0.412706445	0.319	0.428	5.63E-13
Acsl5	3.34E-07	-0.411652342	0.126	0.175	0.018525
Cd34	3.15E-29	-0.410073281	0.724	0.81	1.75E-24
Calml4	2.5E-08	-0.404735152	0.154	0.211	0.001383
Gmppa	5.14E-07	-0.403924172	0.088	0.126	0.0285
Suclg1	3.28E-09	-0.403023014	0.213	0.282	0.000182
Jpt1	7.14E-16	-0.402831678	0.358	0.463	3.96E-11
Bmp1	1.91E-12	-0.402740868	0.312	0.401	1.06E-07
Mrps7	2.73E-08	-0.400804358	0.156	0.213	0.001514
Mrpl36	1.49E-08	-0.400729803	0.181	0.242	0.000825
Coa3	3.85E-13	-0.399404522	0.27	0.359	2.13E-08
Ly6a	3.86E-46	-0.397760065	0.941	0.971	2.14E-41
Mmp23	1.63E-07	-0.393894541	0.202	0.262	0.009058
Ebpl	4.53E-11	-0.390844795	0.171	0.238	2.51E-06
Tmem119	3.22E-10	-0.389367627	0.167	0.233	1.78E-05
Rev3l	1.99E-09	-0.388811466	0.226	0.297	0.00011
Eef1g	9.38E-20	-0.387955776	0.524	0.625	5.2E-15
Saysd1	4.99E-07	-0.38751732	0.114	0.158	0.027631
Nqo2	5.19E-07	-0.386950848	0.118	0.163	0.028785
Olfml3	1.39E-12	-0.38552362	0.46	0.539	7.73E-08
Cd248	5.39E-17	-0.384725156	0.482	0.591	2.99E-12
Ncln	3.41E-07	-0.384550885	0.136	0.185	0.018881
Ostc	3.92E-18	-0.382634767	0.414	0.526	2.17E-13
Eri3	1.93E-09	-0.380306722	0.168	0.23	0.000107
Cadm3	5.28E-09	-0.37984602	0.299	0.373	0.000292
Taldo1	1.05E-16	-0.379624138	0.334	0.44	5.83E-12
Mrpl17	1.58E-08	-0.379542894	0.275	0.345	0.000877
Gpx8	3.69E-17	-0.379182271	0.498	0.592	2.04E-12
Hspa1b	6.59E-07	-0.378820783	0.667	0.684	0.036539
Lysmd2	1.06E-13	-0.378472977	0.405	0.499	5.9E-09
Insyn1	4.95E-07	-0.378036479	0.098	0.139	0.02744
Tmed1	7.84E-10	-0.375752186	0.233	0.306	4.34E-05
Psmb8	6.23E-09	-0.375487875	0.187	0.25	0.000345
Abca8a	4.51E-13	-0.374568829	0.5	0.589	2.5E-08
Acaa1a	3.76E-07	-0.374343361	0.229	0.29	0.020837
Mydgf	1.25E-12	-0.37384211	0.301	0.391	6.94E-08
Babam1	5.77E-07	-0.373429207	0.147	0.197	0.031981
Dap	3.85E-17	-0.373352256	0.522	0.61	2.13E-12
Sh3bgrl3	3.56E-11	-0.372965184	0.505	0.57	1.97E-06
Sfxn1	1.57E-10	-0.372083085	0.167	0.231	8.69E-06
Prrx2	6.01E-13	-0.370604006	0.301	0.392	3.33E-08

Bbip1	8.8E-07	-0.370155261	0.089	0.126	0.04878
Htra2	5.11E-07	-0.369021683	0.116	0.16	0.028296
Ly6e	4.24E-17	-0.368706063	0.516	0.618	2.35E-12
Ifitm3	2.37E-56	-0.364490223	0.966	0.987	1.31E-51
Rcn3	3.44E-19	-0.363530203	0.558	0.661	1.91E-14
Selenow	2.52E-46	-0.361878184	0.884	0.942	1.39E-41
Mrpl4	8.2E-08	-0.36168513	0.183	0.241	0.004543
Chordc1	1.32E-07	-0.361315989	0.17	0.225	0.007342
Phf5a	2.95E-13	-0.36046504	0.2	0.277	1.63E-08
Rab8a	1.14E-09	-0.35960416	0.185	0.249	6.3E-05
Nt5c	1.03E-10	-0.35889669	0.261	0.34	5.7E-06
S100a16	1.73E-18	-0.358439712	0.551	0.656	9.59E-14
Ahsa1	9.05E-09	-0.357592668	0.189	0.25	0.000502
Uqcrc1	1.27E-07	-0.35756969	0.269	0.334	0.007049
Abca9	1.62E-07	-0.356357411	0.26	0.324	0.009002
Arpc1b	4.51E-13	-0.356214941	0.426	0.519	2.5E-08
Yif1a	1.19E-08	-0.351416408	0.221	0.287	0.000659
Rras	5.71E-16	-0.351079513	0.536	0.634	3.17E-11
Rab1b	4.13E-07	-0.349635106	0.194	0.25	0.022884
Lsm4	3.83E-10	-0.34815785	0.341	0.422	2.12E-05
Rap2a	8.24E-09	-0.346404285	0.231	0.299	0.000457
Ccl11	2.02E-08	-0.346304633	0.14	0.197	0.00112
Zmat5	5.58E-07	-0.345758147	0.179	0.233	0.030901
Selenon	5.79E-07	-0.345320999	0.201	0.258	0.032093
Copz2	4.57E-14	-0.34518275	0.375	0.473	2.53E-09
Mrpl51	6.73E-08	-0.344874958	0.208	0.269	0.00373
Tmed3	2.99E-19	-0.344769111	0.608	0.706	1.66E-14
Aldh2	3.23E-21	-0.344249237	0.672	0.753	1.79E-16
Ndufs8	7.19E-08	-0.341259687	0.25	0.315	0.003984
Bst2	5.91E-11	-0.341087313	0.216	0.289	3.28E-06
Atp5o.1	3.46E-13	-0.338752678	0.495	0.577	1.92E-08
Med28	3.57E-10	-0.338556539	0.362	0.444	1.98E-05
Gstm2	2.45E-07	-0.336673784	0.181	0.236	0.013586
Tubb4b	9.46E-11	-0.334786167	0.422	0.503	5.24E-06
Arpc1a	7.14E-12	-0.334712403	0.285	0.369	3.96E-07
Lama4	2.55E-10	-0.334385476	0.384	0.466	1.41E-05
Eva1b	8.66E-07	-0.333897116	0.25	0.31	0.047988
Rbck1	1.06E-08	-0.333783336	0.164	0.219	0.000585
Clec3b	6.76E-24	-0.333415809	0.851	0.91	3.75E-19
Ssr2	1.91E-08	-0.333107275	0.396	0.464	0.001056
Dguok	4.71E-07	-0.331719362	0.19	0.245	0.026074
Map2k2	3.13E-07	-0.331367584	0.162	0.214	0.017372
H1f0	1.05E-09	-0.327884333	0.245	0.318	5.8E-05
Puf60	1.97E-07	-0.326597837	0.21	0.27	0.010932
C3	8E-17	-0.326248606	0.502	0.611	4.43E-12
Akr7a5	5.51E-07	-0.320987881	0.184	0.237	0.030558
Rps2	4.04E-76	-0.319906734	0.995	0.998	2.24E-71

Pfdn6	4.18E-09	-0.319698169	0.233	0.301	0.000232
Fkbp1a	2.78E-15	-0.317983222	0.595	0.681	1.54E-10
Bcl7c	3.77E-10	-0.316751952	0.302	0.382	2.09E-05
Gsn	4.85E-29	-0.316433084	1	0.999	2.69E-24
Cyb561	1.28E-07	-0.316407682	0.095	0.133	0.00707
Tsen34	1.01E-10	-0.31626549	0.319	0.402	5.58E-06
Ift20	2.3E-10	-0.315382233	0.421	0.504	1.27E-05
Rbms2	2.98E-07	-0.314722827	0.167	0.218	0.016517
Hikeshi	2.04E-08	-0.314572418	0.205	0.266	0.001131
Tpi1	1.61E-09	-0.314144558	0.405	0.483	8.92E-05
Edf1	8.86E-17	-0.313324245	0.587	0.678	4.91E-12
Ctsz	9.27E-18	-0.312572203	0.581	0.686	5.14E-13
Nfib	1.06E-15	-0.312570372	0.655	0.742	5.87E-11
Ift27	7.18E-09	-0.311758984	0.28	0.353	0.000398
Cbr3	3.68E-07	-0.311154644	0.256	0.318	0.020394
Rarres2	9.35E-15	-0.31075805	0.793	0.843	5.18E-10
Sf3b5	9.71E-11	-0.3097945	0.436	0.519	5.38E-06
Tshz2	3.39E-14	-0.308509921	0.431	0.531	1.88E-09
Emc10	1.34E-11	-0.308504713	0.422	0.511	7.43E-07
Ndufs4	1.12E-07	-0.308301998	0.27	0.336	0.006192
Dpysl3	1.28E-09	-0.307725147	0.528	0.584	7.11E-05
Mrpl58	1.36E-07	-0.307314232	0.187	0.242	0.007524
Anxa6	9.24E-11	-0.307194054	0.298	0.379	5.12E-06
Mien1	1.74E-09	-0.306879705	0.288	0.362	9.65E-05
Atp6ap1	2.44E-10	-0.304951266	0.39	0.474	1.35E-05
Gabpb2	2.49E-07	-0.304259381	0.18	0.234	0.013811
Bin1	9.26E-09	-0.303887476	0.276	0.347	0.000513
Rplp0	6.32E-60	-0.30379704	0.988	0.993	3.5E-55
Snrpd3	4.65E-09	-0.303247314	0.293	0.367	0.000258
Cuta	3.02E-08	-0.302195253	0.266	0.334	0.001675
Blvrb	2.57E-12	-0.301677585	0.464	0.557	1.42E-07
Slc50a1	8.53E-08	-0.301254264	0.327	0.397	0.004728
Gstm5	2.84E-08	-0.300095063	0.211	0.272	0.001574
Sra1	1.37E-09	-0.299482682	0.344	0.423	7.6E-05
Cdk4	5.76E-08	-0.297500954	0.292	0.361	0.00319
Ccnl1	4.31E-07	-0.297434615	0.455	0.517	0.023907
Emg1	6.62E-10	-0.295005635	0.308	0.385	3.67E-05
Stip1	5.45E-07	-0.294231323	0.197	0.252	0.030193
Gadd45gip	5.72E-07	-0.293654235	0.199	0.253	0.031673
Ddost	1.5E-12	-0.293550304	0.563	0.649	8.34E-08
Yars	7.17E-07	-0.292566321	0.089	0.123	0.039728
Ebf2	5.57E-10	-0.290729458	0.349	0.43	3.09E-05
Tubb6	4.5E-08	-0.290518057	0.271	0.338	0.002495
Rnaseh2c	5.05E-11	-0.290483072	0.307	0.388	2.8E-06
Sec13	5.92E-09	-0.290270731	0.231	0.298	0.000328
Fkbp7	1.43E-09	-0.290133254	0.445	0.524	7.93E-05
Atxn10	1.69E-09	-0.290027729	0.361	0.44	9.34E-05

Rcn1	6.39E-07	-0.289683485	0.243	0.303	0.035417
Serpinf1	7.1E-27	-0.289180406	0.895	0.948	3.93E-22
Rpsa	4.03E-57	-0.288918793	0.99	0.996	2.23E-52
Calm3	2E-08	-0.28851234	0.366	0.44	0.001109
Ptbp1	1.35E-07	-0.288364557	0.24	0.301	0.007465
Marcks	3.9E-13	-0.28745267	0.687	0.763	2.16E-08
Rpl8	3.64E-60	-0.286673957	0.992	0.999	2.02E-55
Leo1	3.89E-07	-0.286669541	0.128	0.17	0.021535
Psmb5	1E-07	-0.28553619	0.329	0.399	0.005565
Pgls	2.63E-09	-0.285287847	0.316	0.392	0.000146
Pigyl	3.28E-07	-0.285240779	0.147	0.192	0.018171
Rpl13a	4.03E-08	-0.285225581	0.193	0.25	0.002231
Ehd2	2.23E-07	-0.285220071	0.333	0.4	0.012346
Acta2	4.39E-08	-0.284476225	0.093	0.132	0.002432
Selenom	6.03E-26	-0.283968847	0.825	0.892	3.34E-21
Ccdc124	2.01E-08	-0.283029534	0.3	0.37	0.001115
Tubb2a	2.45E-07	-0.282542682	0.441	0.509	0.013592
Pomp	7.47E-10	-0.282393955	0.473	0.552	4.14E-05
Glrx3	2.5E-08	-0.280821189	0.306	0.377	0.001387
Hmgn3	1.34E-07	-0.279842085	0.35	0.419	0.00745
Rab3d	4.47E-08	-0.278572593	0.082	0.115	0.002475
Ebp	4.2E-07	-0.278009462	0.261	0.322	0.023283
Atp5d	1.35E-16	-0.277498541	0.702	0.78	7.49E-12
Dynll1	3.69E-20	-0.276550392	0.79	0.862	2.05E-15
Arhgap29	4.62E-07	-0.276254656	0.192	0.246	0.025594
Sgta	4.05E-07	-0.276024092	0.242	0.301	0.022433
Hsd17b10	3.04E-08	-0.275983564	0.293	0.361	0.001685
Eif5a	1.3E-18	-0.275372323	0.737	0.808	7.21E-14
Nfix	3.32E-18	-0.274523772	0.729	0.813	1.84E-13
Anapc11	3.78E-07	-0.274336084	0.397	0.464	0.020946
Tle5	8.27E-15	-0.273879444	0.703	0.765	4.58E-10
Smdt1	7.48E-12	-0.273790846	0.552	0.638	4.15E-07
Wdr83os	1.92E-09	-0.270144651	0.373	0.452	0.000106
Ccdc91	2.01E-07	-0.269458533	0.1	0.137	0.011111
Cryab	3.71E-09	-0.268872625	0.533	0.61	0.000206
Cnpy2	2.18E-11	-0.267281853	0.462	0.551	1.21E-06
Ndufv2	1.35E-08	-0.267051569	0.392	0.468	0.000749
Capns1	2.31E-13	-0.266229115	0.671	0.731	1.28E-08
Grb10	8.44E-07	-0.265538257	0.267	0.328	0.046795
Cope	1.85E-08	-0.264388559	0.498	0.57	0.001025
Snapc5	2.42E-08	-0.264273695	0.272	0.339	0.001342
Il1r1	7.47E-07	-0.263476784	0.35	0.416	0.041406
Mbd3	6.1E-08	-0.262776282	0.314	0.383	0.003378
Eid1	4.21E-12	-0.261667769	0.561	0.649	2.33E-07
Dctn3	7.69E-07	-0.261610595	0.305	0.369	0.042627
Mrpl18	2.89E-08	-0.26020984	0.229	0.289	0.0016
Grpel1	1.95E-08	-0.259092607	0.235	0.296	0.001082

Psme1	6.24E-08	-0.25867974	0.324	0.393	0.003457
Psma4	2.56E-08	-0.257547495	0.444	0.518	0.00142
Mfap5	9.3E-11	-0.257422529	0.812	0.861	5.15E-06
Igfbp6	9.62E-09	-0.257402618	0.977	0.973	0.000533
Dpt	2.07E-19	-0.256554692	0.795	0.865	1.15E-14
Yif1b	9.41E-09	-0.251181331	0.305	0.376	0.000521
2310011J0	3.69E-07	-0.251040715	0.163	0.211	0.020432
Cct5	1.44E-08	-0.249174393	0.467	0.543	0.0008
Ggh	1.41E-07	-0.248197001	0.368	0.438	0.007797
Ociad1	8.48E-07	-0.248077812	0.253	0.311	0.047012
Setd5	4.48E-07	-0.247311673	0.176	0.224	0.024801
Htra3	3.35E-08	-0.245993492	0.575	0.648	0.001857
Mrps34	3.21E-07	-0.245318465	0.172	0.22	0.017792
Tex264	1.01E-07	-0.245244217	0.284	0.347	0.005613
Col5a1	4.52E-07	-0.244324142	0.535	0.592	0.025029
Mrps24	6.42E-11	-0.243883765	0.427	0.513	3.56E-06
Ltbp4	1.81E-10	-0.24382972	0.695	0.756	1E-05
Ftl1	2.57E-11	-0.243702092	0.652	0.721	1.43E-06
Ppib	1.24E-26	-0.243118946	0.895	0.945	6.88E-22
Chchd2	7.53E-12	-0.24232763	0.715	0.778	4.18E-07
Lman1	9.84E-08	-0.242086653	0.443	0.515	0.005453
Shisa5	2.63E-09	-0.241172263	0.448	0.528	0.000146
Arf5	1.01E-12	-0.240444708	0.704	0.774	5.62E-08
Col3a1	1.63E-22	-0.239903014	0.881	0.94	9.02E-18
Ssr4	1.44E-10	-0.239250396	0.657	0.727	8E-06
Eif3i	5.24E-10	-0.238953242	0.541	0.622	2.9E-05
Smim7	1.65E-07	-0.238313598	0.356	0.424	0.009134
Hspe1	6.36E-07	-0.238104573	0.588	0.647	0.03526
Pfn1	2.31E-14	-0.237974705	0.768	0.829	1.28E-09
Psmb2	1.3E-09	-0.236965386	0.439	0.52	7.22E-05
Ddah2	2.51E-10	-0.235912303	0.641	0.71	1.39E-05
Prrx1	1.06E-10	-0.235098165	0.779	0.82	5.88E-06
Antxr2	4.66E-08	-0.234681967	0.413	0.486	0.00258
Bcap31	6.5E-08	-0.234022494	0.494	0.566	0.003602
Mdh1	4.15E-08	-0.23309859	0.398	0.471	0.002302
Acadvl	7.32E-08	-0.233071124	0.257	0.319	0.004056
Cavin3	2.14E-13	-0.232997994	0.774	0.826	1.19E-08
Mpc2	6.32E-09	-0.229246678	0.558	0.631	0.00035
Calr	8E-15	-0.228443388	0.799	0.859	4.44E-10
Psmb6	1.26E-08	-0.228097863	0.568	0.64	0.000695
Sdhb	3.32E-08	-0.227484733	0.353	0.423	0.001839
Tomm5	9.8E-08	-0.227407602	0.318	0.384	0.005432
Ndufb8	1.2E-07	-0.227036041	0.368	0.438	0.006633
Ndufb10	6.47E-10	-0.226606544	0.623	0.695	3.59E-05
Mxra7	5.88E-08	-0.225543839	0.46	0.534	0.003259
Ssna1	2.99E-07	-0.224568149	0.262	0.32	0.016586
Psip1	9.14E-09	-0.22205242	0.284	0.351	0.000507

Pdia6	2.27E-08	-0.22170409	0.569	0.642	0.001256
Rpl7a	3.23E-28	-0.220400689	0.957	0.978	1.79E-23
Zcchc17	5.68E-07	-0.219660276	0.235	0.29	0.031487
Slc25a4	1.26E-12	-0.218938921	0.785	0.833	7.01E-08
Agpat3	6.34E-07	-0.21828852	0.192	0.241	0.035138
Eif2s1	7.08E-07	-0.218197068	0.166	0.21	0.039251
Hadh	5.34E-07	-0.217763817	0.178	0.224	0.029575
Rpl10	2.01E-31	-0.217173628	0.994	0.997	1.11E-26
Rpl10a	7.79E-22	-0.216514794	0.94	0.961	4.32E-17
Scand1	8.16E-07	-0.216430785	0.5	0.568	0.045212
Mrps15	2.25E-09	-0.214591143	0.21	0.268	0.000125
Rps9	3.79E-36	-0.213649514	0.994	0.998	2.1E-31
Ubb	2.9E-12	-0.213553845	0.866	0.898	1.61E-07
Fkbp2	7.16E-07	-0.213388399	0.495	0.562	0.039664
Hsp90b1	9.52E-17	-0.213295644	0.884	0.917	5.27E-12
Tspo	1.16E-08	-0.213107452	0.768	0.806	0.000644
Mrpl34	1.56E-07	-0.210392133	0.191	0.24	0.008633
Chchd1	2.41E-07	-0.20985851	0.322	0.385	0.01335
Rps3a1	2.12E-29	-0.208889497	0.996	0.998	1.18E-24
Rps4x	1.26E-30	-0.208640334	0.998	0.999	6.97E-26
Cfl1	2.7E-10	-0.208307017	0.726	0.782	1.5E-05
Rpl6	1.96E-18	-0.207644177	0.928	0.951	1.08E-13
Rpl9	1.78E-31	-0.206819847	0.995	0.998	9.86E-27
Tex261	9.86E-10	-0.205798974	0.338	0.412	5.47E-05
Serping1	1.16E-10	-0.201914822	0.958	0.97	6.43E-06
F3	3.91E-07	-0.201613542	0.099	0.138	0.021665
Bsg	3.8E-13	-0.201064774	0.79	0.852	2.11E-08
Fstl1	1.61E-07	-0.200318624	0.896	0.915	0.008909
Lrpap1	4.34E-07	-0.199161886	0.345	0.409	0.024067
Mlf2	3.48E-07	-0.197341992	0.343	0.407	0.019258
Rsrp1	9.65E-11	-0.196012208	0.742	0.809	5.35E-06
Cd81	3.8E-15	-0.195050641	0.918	0.937	2.1E-10
Rala	7.21E-07	-0.194847006	0.406	0.472	0.039958
Prdx1	1E-09	-0.194439458	0.791	0.834	5.55E-05
Tmed9	2.98E-07	-0.193715576	0.601	0.662	0.016495
Gpx4	1.51E-07	-0.193680074	0.594	0.66	0.008382
Rexo2	5.14E-07	-0.192719386	0.429	0.497	0.028488
Anxa2	1.1E-11	-0.191298664	0.895	0.926	6.09E-07
Ppp1ca	9.2E-08	-0.187670337	0.521	0.593	0.005101
Rps5	1.01E-28	-0.187236971	0.999	0.999	5.59E-24
Dad1	2.44E-10	-0.187090034	0.685	0.759	1.35E-05
Prdx2	3.35E-07	-0.18666444	0.676	0.724	0.018554
Ndufb5	3.78E-07	-0.186188112	0.489	0.558	0.020928
Mzt2	6.86E-07	-0.185281394	0.132	0.169	0.038008
Txnl4a	6.77E-07	-0.185007889	0.184	0.228	0.037492
Tek	2.94E-07	-0.183971084	0.101	0.133	0.016296
Tmsb10	7.33E-09	-0.182227972	0.681	0.746	0.000406

Npdc1	5.25E-07	-0.182149334	0.56	0.626	0.029115
Rps3	1.48E-07	-0.180360481	0.716	0.77	0.008181
Cald1	1.97E-09	-0.179621961	0.68	0.75	0.000109
Rack1	1.99E-18	-0.179080274	0.979	0.984	1.1E-13
Mrfap1	1.22E-07	-0.178318142	0.4	0.468	0.006782
Mgst1	7.04E-08	-0.178282517	0.685	0.749	0.0039
Cct3	8.35E-07	-0.176791013	0.287	0.342	0.04626
Ndufs7	9.23E-08	-0.17580036	0.508	0.579	0.005112
Cyba	3.84E-08	-0.175703189	0.666	0.734	0.002127
Zbtb20	1.66E-12	-0.174693396	0.943	0.957	9.18E-08
Psmb1	3.56E-08	-0.1743253	0.654	0.718	0.001973
Nenf	4.4E-11	-0.174076339	0.856	0.893	2.44E-06
Emp3	1.27E-12	-0.17403987	0.87	0.914	7.03E-08
Hsp90ab1	1.12E-12	-0.173757985	0.971	0.974	6.21E-08
Snrpb	9.36E-08	-0.173719085	0.571	0.642	0.005185
Ssrp1	4.88E-08	-0.170321881	0.271	0.329	0.002705
Akr1a1	5.11E-07	-0.165739834	0.716	0.764	0.028342
Gapdh	3.07E-07	-0.163802762	0.622	0.688	0.016996
Rps7	5.07E-16	-0.163763108	0.962	0.98	2.81E-11
Fbn1	5.8E-12	-0.162687148	0.662	0.746	3.21E-07
Gabarap	1.03E-12	-0.161974887	0.907	0.944	5.71E-08
Rpl13	2.93E-22	-0.160065128	0.996	0.998	1.62E-17
Rpl19	7.11E-18	-0.160035592	0.993	0.997	3.94E-13
Hbb-bt	1.14E-12	-0.158676053	0.279	0.199	6.3E-08
Calm1	9.53E-08	-0.153244361	0.884	0.909	0.005281
Rpl18	8.71E-13	-0.150080392	0.908	0.943	4.83E-08
Elof1	3.08E-08	-0.149886366	0.259	0.313	0.001707
Trappc2l	5.91E-07	-0.147776114	0.311	0.366	0.032741
Selenof	3.41E-09	-0.147006954	0.833	0.876	0.000189
Zfp871	9.95E-08	-0.145041508	0.233	0.282	0.005514
Rpl5	2.35E-09	-0.144964449	0.934	0.945	0.00013
Col1a2	1.57E-25	-0.144803585	0.935	0.979	8.71E-21
Mgat2	2.08E-07	-0.144522743	0.319	0.375	0.0115
Atp6v0b	2.98E-07	-0.143958995	0.388	0.451	0.016503
Mia2	2.86E-07	-0.142617865	0.258	0.306	0.015842
Tmed10	2.65E-09	-0.140135467	0.654	0.728	0.000147
Tmem126b	8.24E-07	-0.139333088	0.078	0.104	0.04564
Ybx1	4.67E-10	-0.136865377	0.926	0.94	2.59E-05
Ifngr2	1.8E-08	-0.136083471	0.17	0.211	0.000996
Myl6	3.96E-07	-0.135141902	0.854	0.891	0.02196
Eef1d	3.76E-07	-0.134863758	0.807	0.854	0.020819
Rnf187	1.71E-07	-0.133545952	0.397	0.46	0.009474
Lamb2	2.33E-07	-0.133057153	0.578	0.644	0.012887
Rbp1	1.37E-07	-0.131950204	0.192	0.24	0.007593
Eef1b2	5.54E-09	-0.130903658	0.913	0.923	0.000307
Lamtor4	3.08E-07	-0.13085328	0.401	0.462	0.017049
Itm2b	4.94E-16	-0.128425772	0.998	0.998	2.74E-11

Naca	3.77E-07	-0.127001394	0.951	0.962	0.02087
Eif1b	1.22E-07	-0.125660439	0.358	0.416	0.006753
Rpl7	3.75E-07	-0.125584907	0.906	0.929	0.020768
Psmb4	7.78E-08	-0.125480238	0.56	0.629	0.004313
Copb2	3.12E-07	-0.121019573	0.422	0.483	0.017291
Rnase4	8.17E-07	-0.119645693	0.929	0.947	0.045266
Rpl18a	1.4E-11	-0.117512411	0.993	0.996	7.78E-07
Rpl14	5.68E-10	-0.116773971	0.974	0.985	3.15E-05
Pten	2.86E-07	-0.115926941	0.343	0.399	0.015866
Actb	6.74E-13	-0.115167259	0.99	0.995	3.74E-08
Tmsb4x	2.25E-08	-0.112577524	0.987	0.995	0.001247
